# One-Electron Approach for Trans-Selective Alkyne Semi-Reduction via Cobalt Catalysis

**DOI:** 10.1021/jacs.5c07630

**Published:** 2025-10-28

**Authors:** Rakesh Mondal, Lior Galmidi, Avra Tzaguy, Tal Sason, Moran Feller, Mark A. Iron, Liat Avram, Ronny Neumann, Samer Gnaim

**Affiliations:** † Department of Molecular Chemistry and Materials Science, 34976 Weizmann Institute of Science, Rehovot 7610001, Israel; ‡ Department of Chemical Research Support, Weizmann Institute of Science, Rehovot 7610001, Israel

## Abstract

The diastereoselective semireduction of alkynes to alkenes is a powerful transformation in synthetic chemistry, yet catalytic methods for trans-selective (E) alkyne reduction remain limited. Herein, we introduce a fundamentally new approach for the highly selective trans-semireduction of internal alkynes, enabled by a cobalt-catalyzed electrochemical radical pathway. This method offers a broad substrate scope, accommodating alkynes with diverse electronic and steric profiles, and displays exceptional chemoselectivity and functional group tolerance. The methodology was extended to isotopically labeled trans-deuteration and demonstrated excellent chemoselectivity in substrates containing multiple alkyne motifs. Mechanistic studies, including cyclic voltammetry, UV–vis spectroelectrochemistry, and DFT calculations, support a dual catalytic cycle involving electrochemical Co–H formation and a subsequent organometallic radical pathway. Insights from this mechanism guided the development of a complementary chemical oxidative protocol, enabling access to *E*-alkenes from substrates that are otherwise unreactive under electroreductive conditions. This work introduces a fundamentally new and general strategy for accessing *trans*-alkenes from alkynes via cobalt catalysis while opening a new avenue for radical-based alkyne functionalization.

## Graphical abstract

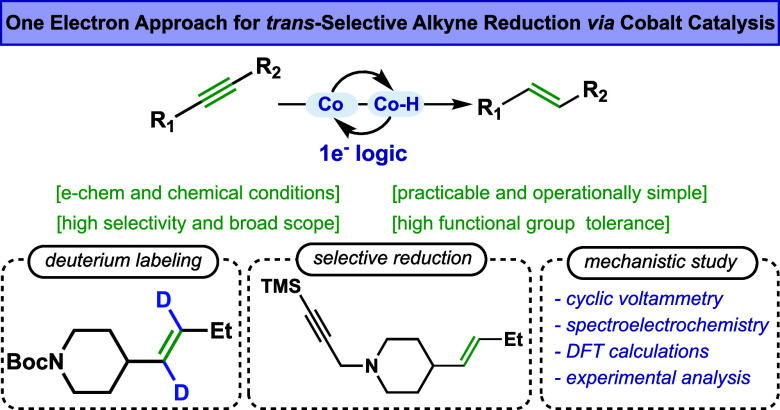

## Introduction

From the building blocks of life to transformative medicines, compounds featuring alkenyl double bonds have significantly influenced human health and society. These stereochemically defined functionalities, *E* or *Z*, find applications across numerous areas of chemical science, including pharmaceuticals, agrochemicals, and intermediates in organic synthesis.^1–4^ Among the ways to forge these bonds, the selective reduction of alkynes emerges as a particularly elegant solution, transforming rigid triple bonds into precisely tailored olefins.^5–7^ In this regard, achieving a diastereoselective reduction of triple bonds is vital for the practical utility of these reactions (Figure 1).

**Figure 1:**
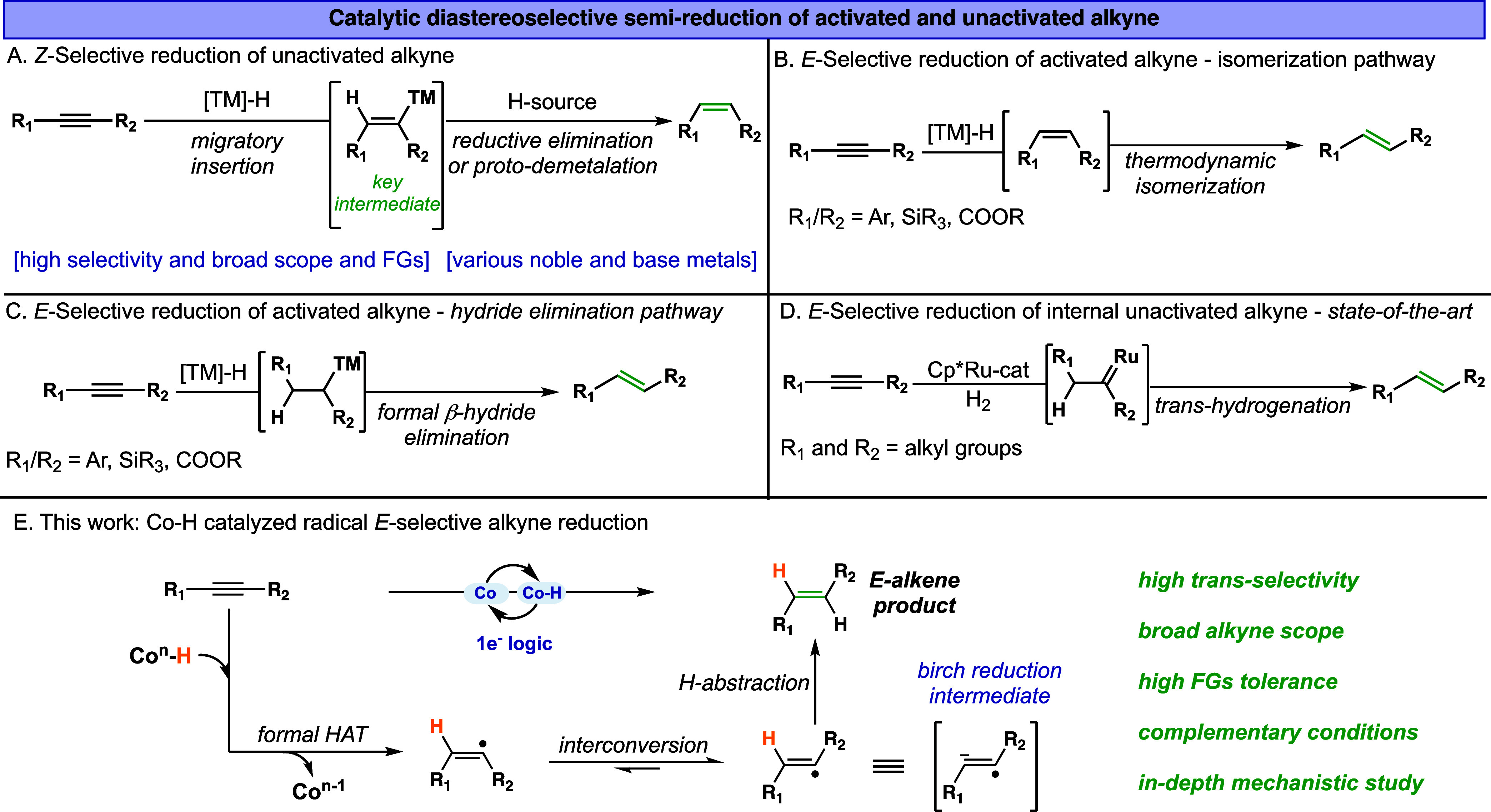
(A) Z-selective semireduction of unactivated internal alkynes. (B) E-selective semireduction of activated internal alkynes via an isomerization pathway. (C) E-selective semireduction of activated internal alkynes through β-hydride elimination pathway. (D) E-selective semireduction of unactivated internal alkynes via ruthenium catalysis. (E) This work and the envisioned mechanism. FGs—functional groups.

The catalytic Z-selective semireduction of alkynes to *cis*-alkenes is a well-established area in synthetic chemistry, with numerous methodologies developed over the years (Figure 1A).^8^ These advancements span both homogeneous and heterogeneous catalytic systems, utilizing a wide range of transition metals, from noble metals such as Au, Pt, Rh, and Pd to more earth-abundant base metals like Cr, Co, Ni, Fe, and Cu. Collectively, these efforts have led to over 20 distinct protocols for the selective Z-reduction of alkynes, offering broad substrate scopes and excellent tolerance to diverse functional groups.^9–24^ The rapid progress in this field is largely driven by a well-understood mechanistic foundation centered on metal hydride or dihydride species, which act as active catalysts.^25,26^ The main key step underpinning Z-selectivity is the syn-insertion of a transition metal hydride into the alkyne, a hydrometalation process, typically followed by a formal reductive elimination or protodemetalation to yield the Z-alkene product (Figure 1A).

In contrast, a concerted anti-insertion of a metal hydride into a π-system is geometrically unfeasible, making the selective formation of *E*-alkenes particularly difficult. As a result, since the novel work of Milstein and co-workers,^27^ the majority of trans-selective semireduction methods developed over the past decade have been effective primarily for conjugated alkynes, such as those bearing aryl, silyl, or carbonyl substituents, where inherent electronic effects facilitate this transformation (Figure 1B,C). Mechanistically, these strategies often proceed via the in situ generation of a *Z*-alkene intermediate, which subsequently undergoes metal-mediated isomerization to yield the *E*-alkene (Figure 1B).^17,28–35^ An alternative proposed mechanism is the full reduction of the alkyne to a hydrometalated alkane intermediate, which is followed by a concerted β-hydride elimination to generate the *E*-alkene (Figure 1C).^16,36–42^ In both cases, the presence of an electron-withdrawing conjugating group is crucial, as it thermodynamically drives the formation of the trans isomer. Despite significant progress, there is currently one main catalytic approach that has shown practical utility for unactivated internal alkynes.

Fürstner and colleagues developed a ruthenium-catalyzed trans-selective hydrogenation, marking the first broadly applicable method for a wide range of unactivated internal alkynes with a good functional-group tolerance (Figure 1D). This system employs the precious metal complexes [Cp*Ru­(cod)­Cl] or [Cp*RuCl]_4_ under hydrogen pressure to reduce alkynes to the corresponding *trans*-alkenes.^43,44^ This highly valuable methodology has been employed in several total syntheses of natural products.^45,46^ Mechanistic investigations suggest that a labile ruthenium­(II) intermediate likely governs the stereochemical outcome. The alkyne binds to a [Cp*Ru]-based catalyst and forms a metallacyclopropene intermediate. From this point, two competing pathways are possible: one leads directly to the *E*-alkene product via concerted trans-delivery of both hydrogen atoms from H_2_, while the other forms a ruthenium carbene intermediate through a rare gem-hydrogenation step.^43,47–49^ In the realm of noncatalytic strategies for trans-selective alkyne reduction, the traditional toolkit primarily revolves around dissolving metal conditions.^50–52^ While less common, certain chromium-based reagents have also been employed for electron transfer to triple bonds to achieve similar outcomes.^53,54^ Additionally, metal hydrides like LiAlH_4_ (LAH) have proven effective for selectively reducing propargyl alcohols to their corresponding *E*-allylic alcohol derivatives.^55,56^

Building on state-of-the-art methods, there is a growing recognition within the synthetic community of the urgent need to develop fundamentally new mechanistic pathways for trans-selective alkyne reduction that can match the selectivity, substrate scope, and generality achieved by established Z-selective strategies.^57,58^ Drawing inspiration from the dissolving metal reduction of alkynes using sodium or lithium^50,51^ - where radical chemistry drives the trans-selective outcome,^52^ we envisioned that a similar principle could be applied through a one-electron catalytic platform utilizing hydrogen-atom transfer (HAT) paradigms. While metal hydride HAT catalysis has revolutionized the field of alkene reduction, isomerization, and functionalization, allowing the construction of new chemical bonds with unmatched selectivity and efficiency,^59–72^ its reactivity with alkynes is rarely discussed in the literature.^20,73^ This work presents a conceptually new approach for the trans-selective alkyne reduction, leveraging a cobalt-hydride (Co–H) radical-mediated pathway. This methodology offers excellent chemoselectivity and significantly expands the range of compatible substrates while preserving the trans-stereochemical preference observed in classical dissolving metal reductions. As illustrated in Figure 1E, our envisioned mechanism begins with a formal HAT, generating a vinyl radical, akin to the intermediate formed in dissolving metal systems, which subsequently equilibrates to the thermodynamically favored trans-isomer. The reaction is then completed through a termination step involving hydrogen abstraction, yielding the *trans*-alkene product.

## Results and Discussion

With the recent advances in Co-HAT catalysis with alkenes, where cobalt-hydride can be formed oxidatively or reductively, our primary focus was on the reductive process due to the suggested improved chemoselectivity.^20,74,76^ In this instance, the active Co–H intermediate requires a single electron reductant and a proton source to facilitate its mild formation. Accordingly, compound **1** was selected for this initial study (Figure 2). Such a simple-looking substrate **1** was challenging to reduce with known dissolving metals conditions (see Supporting Information—additional data section). In our study, we found that electrochemistry, as the terminal reductant, provided the most favorable conditions for both cobalt hydride generation and its subsequent reactivity (Figure 2). When **Co-1** was used as a catalyst, hexafluoroisopropanol (HFIP) as a proton source, and zinc sacrificial anode conditions in acetone were applied, product **2** was obtained in 69% yield and a 36:64 *E*/*Z* ratio (entry 1). Next, we turned our study to test the catalyst’s impact on the yield and *E*/*Z* ratio. Notably, cobalt complexes supported with salen-based ligands showed hydrogenation reactivity (**Co-1–Co-5**, Figure 2), while the other Co complexes gave no product due to suspected decompositions of these catalysts under the electro-reductive conditions (**Co-6**, **Co-7**, and **Co-8**, Figure 2). While several catalysts were tested (entries 2–6), **Co-4** gave the best results with a 40% yield and 84:16 *E*/*Z* ratio. An interesting correlation between the ligand steric environment and E-selectivity can be noticed here. Next, a solvent screen revealed that alcoholic solvents perform the best, with dry *iso*-BuOH yielding 67% and a 94:6 *E*/*Z* ratio (entry 11). It is worth noting that while entry 10 with *tert*-BuOH as a solvent gave a good reaction outcome, we observed an immediate increase in the reaction voltage due to the high resistance of the reaction, leading to side products and decompositions. Next, the proton source screen study revealed that HFIP (20 equiv) proved optimal (entries 12–14). Finally, the additive screen showed that adding 2 equiv of water (see Supporting Information, additional experiments section for further discussion) and electrolysis for 16F/mol gave the best result, 88% isolated yield and 96:4 *E*/*Z* ratio (entry 15). It is worth noting that the over-reduction alkane side product was not detected, and no isomerization product was observed. While several cathodic materials (e.g., Ni, Ni-foam, and graphite) were applicable to the reaction, we found that the use of a tin (Sn) cathode was important to obtain a reproducible reaction outcome. Notably, chemical reductants, such as magnesium (Mg), iron (Fe), manganese (Mn), and zinc (Zn), instead of electricity, failed to give the expected product (entry 16), highlighting the need for electrochemistry in this aspect (see Supporting Information, additional data section for further discussion). Reactions without electricity, a catalyst, or HFIP showed no product formation (entries 17–19). The final set of reaction conditions is operationally simple and does not require inert atmosphere techniques such as a glovebox or Schlenk line. The reaction can be set up within minutes using a basic, undivided electrochemical cell and a commercially available potentiostat. This makes the procedure highly accessible and user-friendly for standard laboratory settings.

**Figure 2:**
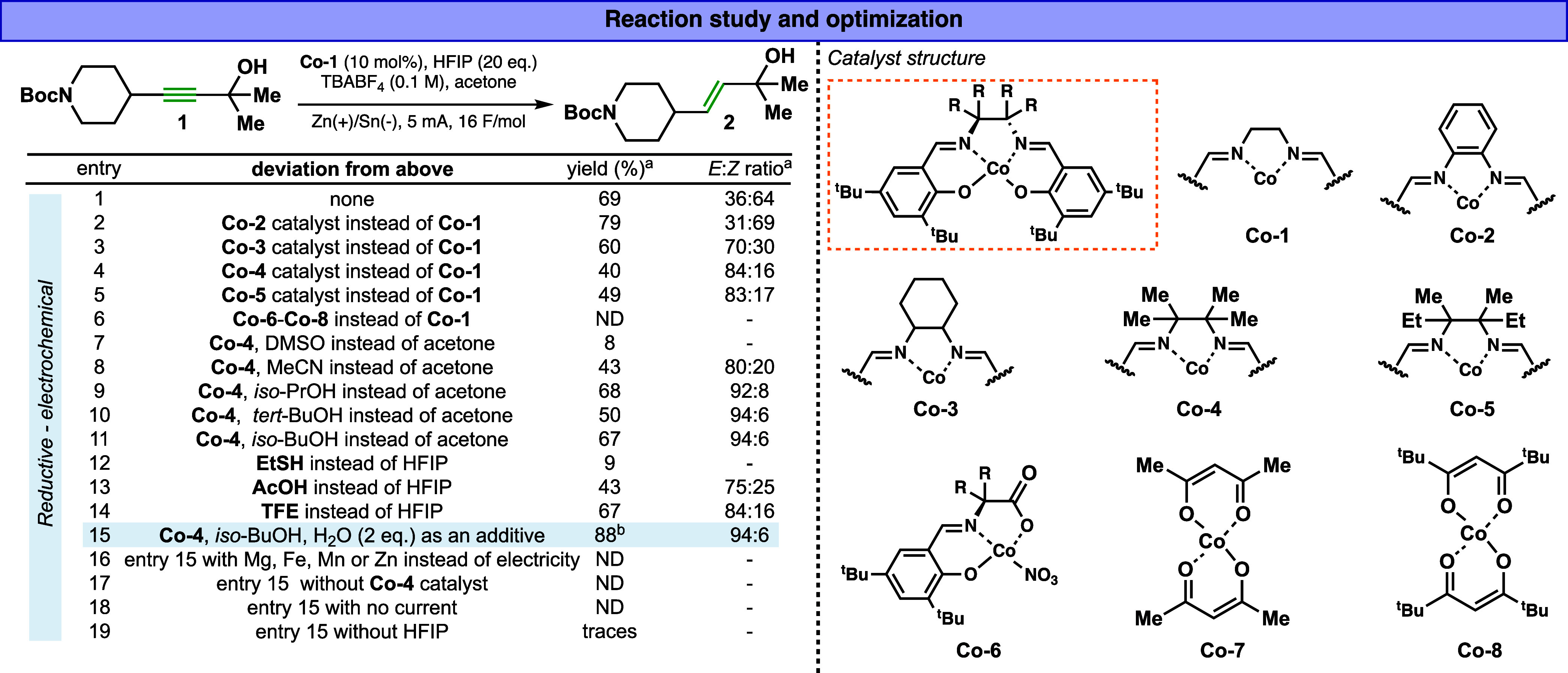
(left) Optimization study of the electrochemical trans-reduction of alkynes to alkene. (right) Structure of the cobalt catalysts. ^a^ Yields and ratios were determined using ^1^H NMR. ^b^ Isolated yield.

With the optimized reaction conditions established, we investigated the reaction scope using alkynes with varying electronic and steric properties, as shown in Figure 3. Our study focused on three key parameters: the electronic characteristics of the alkyne, the steric influence of aliphatic substituents, and the reaction’s functional group tolerance. The optimized conditions were applied across different substrates, with the charge passage adjusted between 3 and 24 F/mol depending on the alkyne type. The *E*/*Z* isomeric ratios were primarily determined using ^1^H NMR analysis. In cases where the proton peaks of *E*/*Z* isomers overlapped (compounds **10**, **12**, **14**, **15**, and **17**), GC–MS analysis was employed, with authentic Z isomers synthesized using established protocols. In some instances (compounds **10**, **11**, **16**, and **17**), alkane formation was observed, as noted in Figure 3. In certain cases (compounds **19**, **24**–**29**, **31**, and **32**), increasing the catalyst loading to 15 mol % proved beneficial, resulting in improved yields.

**Figure 3:**
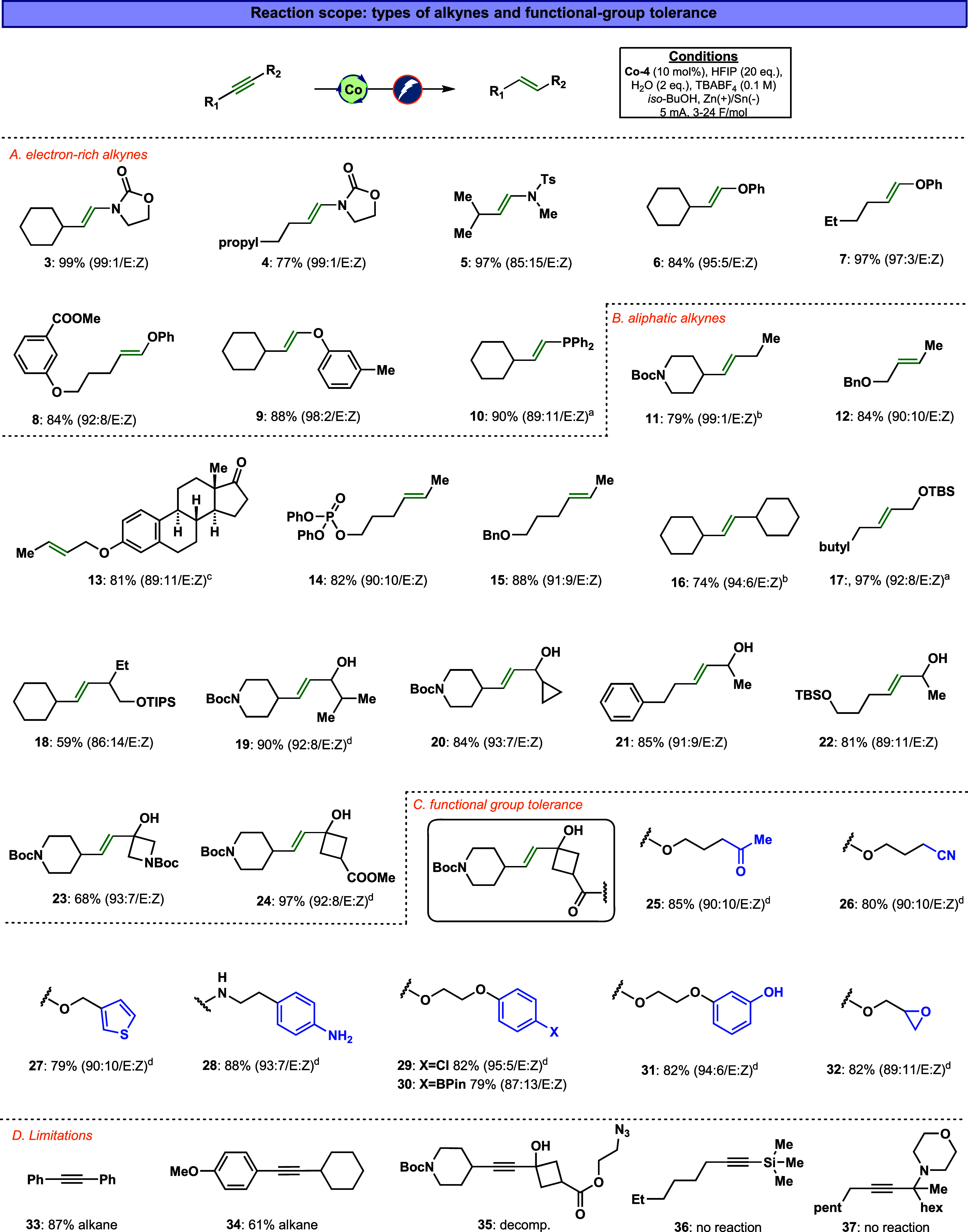
Co-catalyzed trans-reduction of alkynes to trans-alkenes—scope and functional group tolerance. If not noted otherwise, all reactions were performed with the optimized conditions. ^a^ 20% alkane, ^b^ 12% alkane, ^c^ 4% alkane, ^d^ Co-4 (15 mol %).

We first applied the optimized conditions to the reduction of electron-rich alkynes. The reaction performed efficiently with ynamides, yielding *E*-enamine products with high selectivity and yield. This was demonstrated using oxazolidinone-substituted alkynes and tosyl-protected secondary amines (Figure 3, compounds **3**–**5**). The reaction is effective across a variety of secondary and tertiary aliphatic-substituted ynamides. While catalytic Z-selective reduction of ynamides to enamines is established in the literature,^77,78^ to our knowledge, this is the first reported catalytic example of their trans-selective reduction. Similarly, the electrochemical trans-reduction was effective for generating *trans*-enol ethers. A range of aliphatic-substituted ynol ether precursors underwent selective reduction to the corresponding enol ethers with high E-selectivity (compounds **6**–**9**). In some cases, LAH was used to achieve a similar reduction;^79^ however, its nonselective nature presents challenges when dealing with substrates containing redox-sensitive functionalities. For instance, LAH failed to produce enol ether **8** (see Supporting Information—additional data section for further details). Additionally, this method proved effective for reducing phosphine-alkynes, as obtained with compound **10**.

Next, we investigated the reaction with unactivated internal alkynes. Under the same conditions, a variety of aliphatic alkynes were successfully reduced, including secondary–tertiary (**11**), primary–secondary (**12**–**15**), secondary–secondary (**17**), and tertiary–tertiary **16** and **18** (TIPS deprotected products were observed during the reaction progress), substituted alkynes. In addition, aliphatic propargylic alcohol substrates underwent efficient reduction, delivering the desired products with several secondary (**19**–**22**) and tertiary alcohols (**2**, **23**, and **24**) in high yields and selectivity.

During our study, we observed that the developed methodology exhibits an exceptionally broad functional group tolerance. The reaction successfully accommodated protic functional groups such as free alcohols (**19**–**24**), phenols (**31**), and anilines (**28**). Additionally, the electrocatalytic reduction is compatible with redox-sensitive groups, including esters (**8** and **24**), epoxides (**32**), nitriles (**26**), Boc-protected carbamates (**11**, **19**, and **20**), and ketones (**25**). The hydrogenation reaction also tolerated electron-rich arenes (**31**), aryl chlorides (**29**), aryl boronic esters (**30**), and heterocycles (**27**). On the other hand, aliphatic azide (**35**) was found to be incompatible, as it underwent decomposition under the reaction conditions. Moreover, aryl conjugated alkynes exhibit high over-reduction alkane product, presumably due to direct cathodic reduction (**33** and **34**). Silyl-substituted alkynes (**36**), and propargylic amines (**37**) are inactive under the reaction conditions, leading to complete recovery of the starting materials, presumably due to the high steric environment around the triple bonds.

To further expand the utility of our electrocatalytic system and address key challenges in the selective reduction of alkynes, we turned our attention to the development of an E-selective deuteration protocol. While Z-selective deuterium labeling of alkynes is well established in the literature,^80–82^ a general and practical approach for the E-selective deuteration of alkynes has not yet been reported to the best of our knowledge. Our electrocatalytic system offers a straightforward and practical opportunity to fill this gap. By simply replacing the proton source with its commercially available deuterium-labeled analogue, we translated our optimized hydrogenation conditions to a deuterium-labeled version without further modifications. Specifically, the use of HFIP-*d*_1_ (HFIP-OD) as the deuterium source, in combination with *iso*-PrOD-*d*_1_ as the solvent and **Co-4** as the catalyst, and maintaining the same electrolyte and electrochemical parameters, enabled efficient and selective trans-deuteration of alkynes under mild conditions. This modified protocol was applied to a set of five alkyne substrates bearing diverse electronic and steric features. In all cases, the transformation proceeded with excellent E-selectivity (*E*/*Z* > 90:10) and high levels of deuterium incorporation (80–90%), affording the corresponding deuterium-labeled *trans*-alkenes (compounds **38**–**42**, Figure 4A). This achievement not only showcases the versatility of our system but also provides a powerful and user-friendly platform for accessing stereodefined, deuterium-labeled alkenes, valuable compounds in medicinal chemistry, mechanistic studies, and isotope tracing applications.

**Figure 4:**
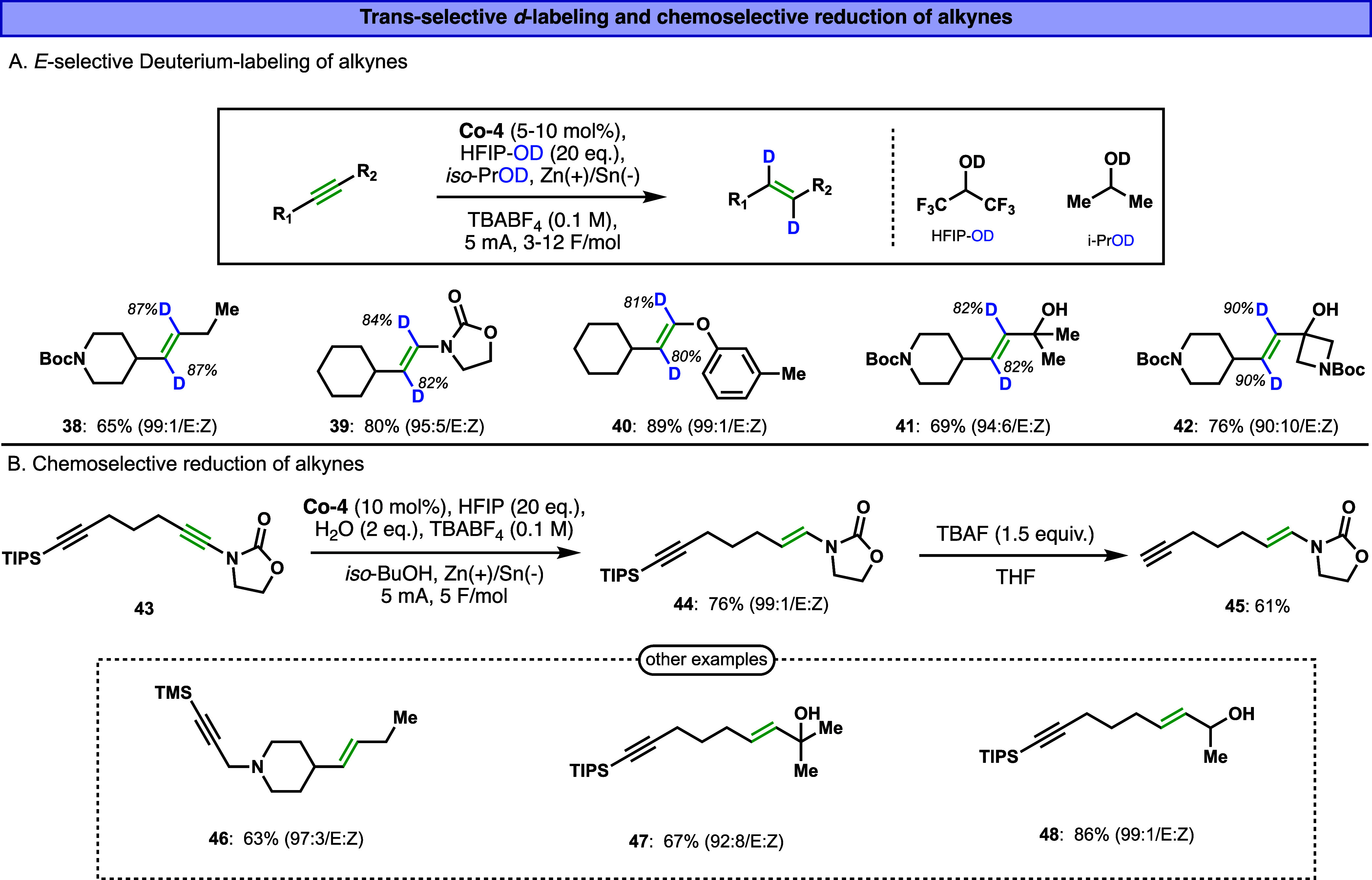
(A) Trans-selective deuteration of alkynes. Ratios and yields determined using NMR. (B) Chemoselective reduction of alkynes in diyne substrates.

During the exploration of substrate scope, we noticed that our catalytic system exhibited a remarkable ability to discriminate between internal alkynes based on their electronic properties and steric environments. We wondered whether this intrinsic selectivity could be harnessed to achieve site-selective reductions within more complex molecules containing multiple alkyne units. Specifically, we examined substrates bearing two alkyne groups, one of which was substituted with a silyl moiety. This chemoselectivity opens a valuable synthetic strategy, where the silyl group can serve as a temporary protecting group for terminal alkynes, allowing for downstream transformations or late-stage functionalization. Remarkably, the catalytic system consistently favored the reduction of the electronically activated alkyne, leaving the silyl-protected alkyne untouched. For example, substrate **43**, containing both a ynamide and a silyl-substituted alkyne, undergoes selective reduction at the ynamide position to yield the corresponding *trans*-enamide in 76% isolated yield (Figure 4B). Subsequent deprotection of the silyl group quantitatively furnished the terminal alkyne product **45**. Encouraged by this result, we applied the same logic to substrates bearing alkynes with varying electronic and steric profiles, successfully obtaining the corresponding *E*-alkenes **46**–**48** with moderate/high yields and high selectivities (Figure 4B).

To gain a deeper mechanistic understanding of the trans-selective semihydrogenation of alkynes, we conducted a series of electroanalytical experiments, spectroscopic analyses, and DFT calculations. Our data indicate that the transformation operates via two interconnected catalytic cycles: an electrochemical cycle generating the active cobalt-hydride species and a subsequent chemical hydrogenation cycle (Figure 5A).

**Figure 5:**
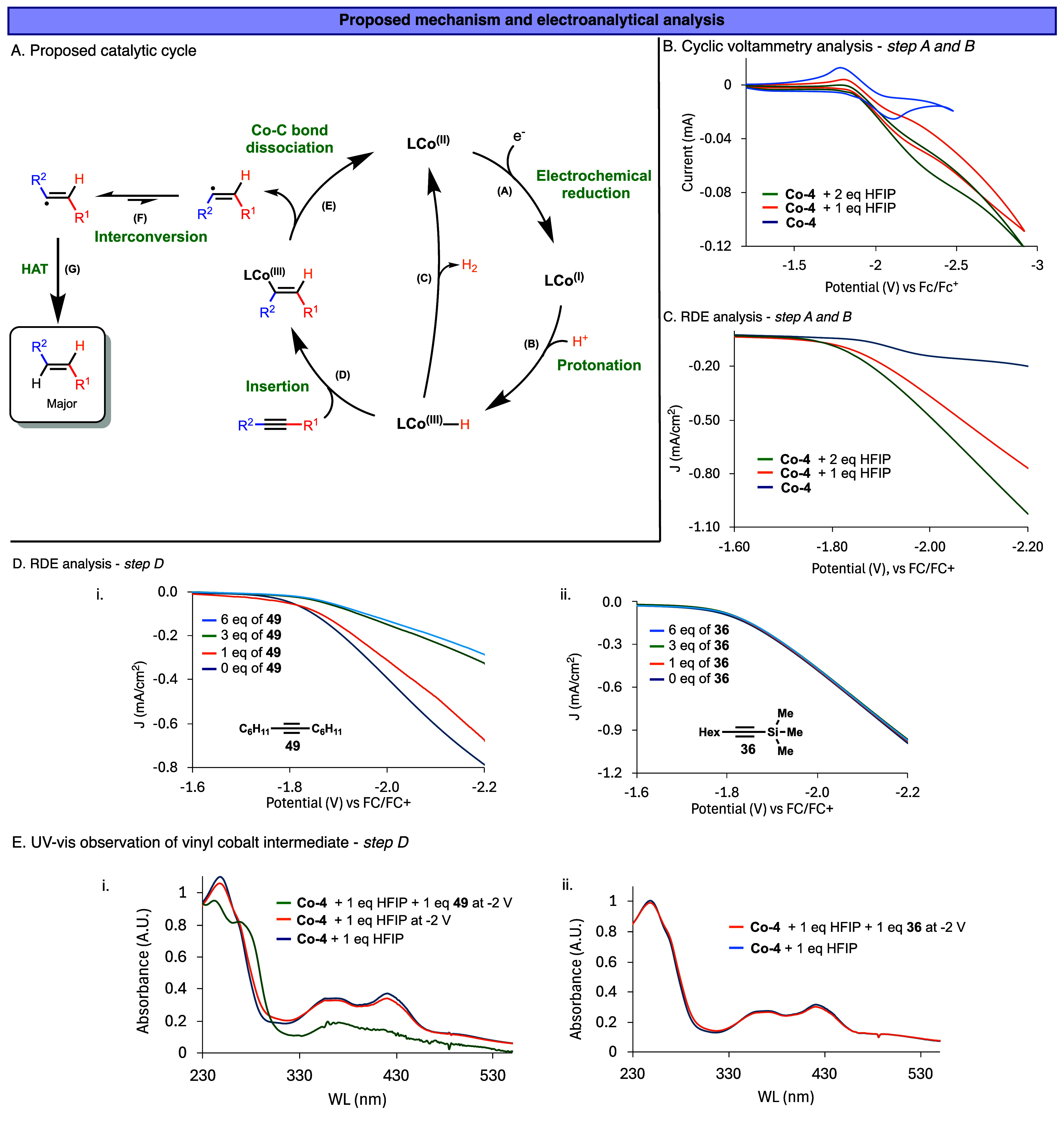
(A) Proposed mechanism. (B) Cyclic voltammetry of **Co-4** catalyst with and without HFIP. (C) RDE analysis of **Co-4** catalyst with and without HFIP. (D) RDE analysis of **Co-4** catalyst with HFIP in the presence of alkynes **36** and **49**. (E) In situ UV–vis analysis of the **Co-4** electrolysis reaction with alkynes **36** and **49**. CV and RDE conditions: 1 mM of Co-4, 1–2 mM HFIP, 1–6 mM alkynes, and 0.1 M TBABF4 in *iso*-BuOH, 100 mV/s (for CV) and 100–250 rpm (for RDE).

In the electrochemical cycle, the cobalt catalyst is reduced at the cathode from Co­(II) to Co­(I) (step A, Figure 5A), which is then protonated by a Brønsted acid, specifically HFIP, to form a transient cobalt-hydride intermediate (step B, Figure 5A). In the absence of HFIP, cyclic voltammetry (CV) measurement of **Co-4**, in *iso*-BuOH as a solvent, displays a reversible redox couple at −1.9 V vs Fc/Fc^+^, corresponding to the Co­(II)/Co­(I) system (Figure 5B). Upon the addition of one or two equivalents of HFIP, in *iso*-BuOH as a solvent, an over 3-fold increase in catalytic current is observed (−0.03 to −0.09 mA), indicative of protonation and subsequent catalytic hydrogen evolution, consistent with literature reports on cobalt-mediated electrocatalysis.^74,83^ In addition, this result suggests that the acidic proton in HFIP is the hydride source in the Co–H intermediate. Further validation was obtained via rotating-disk electrode (RDE) experiments. Increasing HFIP concentrations led to higher current densities (Figure 5C), again supporting the formation of a reactive Co–H intermediate followed by hydrogen evolution.

Notably, upon titration of alkyne substrate **49** (1–6 equiv) into a solution containing **Co-4** and two equivalents of HFIP in *iso*-BuOH (Figure 5D­(i)), the current density steadily decreases from −0.80 to −0.28 mA/cm^2^, approaching the baseline of **Co-4** alone without HFIP. This suggests that the in situ-generated Co–H species is being consumed in a chemical reaction with the alkyne (Figure 5A, step D). In contrast, when a nonworking alkyne (compound **36**) was used, no change in current was observed (Figure 5D­(ii)), reinforcing the notion that the current intensity decrease is observed when the Co–H reaction occurs. As a control, the titration of substrate **49** in the absence of HFIP yielded no significant RDE changes (see Figures S1 and S2). These findings support a mechanism where the Co–H intermediate reacts with the alkyne via an insertion step to form a vinyl-cobalt­(III) complex (Figure 5A, step D). While HAT is often invoked in cobalt catalysis, particularly with alkenes, previous studies have shown that under certain conditions, cobalt-salen complexes can facilitate insertion.^61,65^

This was further supported using in situ UV–vis spectro-electrochemical analysis.^84–86^ The UV–vis spectrum of **Co-4** in *iso*-BuOH with HFIP, without an applied potential, exhibits characteristic absorption peaks at 249, 367, and 422 nm (Figure 5E­(i)). These features are generally consistent with a combination of electronic transitions typical for cobalt coordination complexes, including metal-to-ligand charge transfer (MLCT), ligand-to-metal charge transfer (LMCT), and intraligand (π–π*) excitations.^87–90^ Upon applying a potential of −2.0 V, a slight decrease in intensity is observed, attributed to the formation of Co–H and partial consumption of **Co-4**. Upon addition of 1 equiv of alkyne **49** under the same electrochemical conditions, a new absorption peak emerges at 275 nm, accompanied by attenuation of the peaks at higher wavelengths. This spectral shift is attributed to the formation of the vinyl-Co­(III) intermediate, where the π-accepting vinyl ligand alters the electronic environment of cobalt and leading to the formation of a low-spin Co­(III) species from the high-spin Co­(II) precursors.

The mechanism of the formation of the Co­(III)-vinyl intermediate was further supported by DFT calculations (Figure 6A, see the Computational Methods section of the Supporting Information for more details). Initially, the Co­(III)-hydride forms a long-range complex with the alkyne, intermediate **I**, with an associated reaction energy of Δ*G*_298_ = 3.6 kcal/mol. This subsequently undergoes an outer-sphere insertion of the alkyne into the Co–H bond. The transition state, **II**, for this reaction shows a concerted formation of Co–C and C–H bonds, leading directly to the vinyl complex **III**. This leads to a reasonable barrier height of Δ*G*_298_^⧧^ = 16.3 kcal/mol and a reaction energy of Δ*G*_298_ = −11.3 kcal/mol. In this instance, the *Z*-vinyl-cobalt intermediate **III** is 4.9 kcal/mol more stable than the *E*-isomer (see Supporting Information—Computational Methods section).

**Figure 6:**
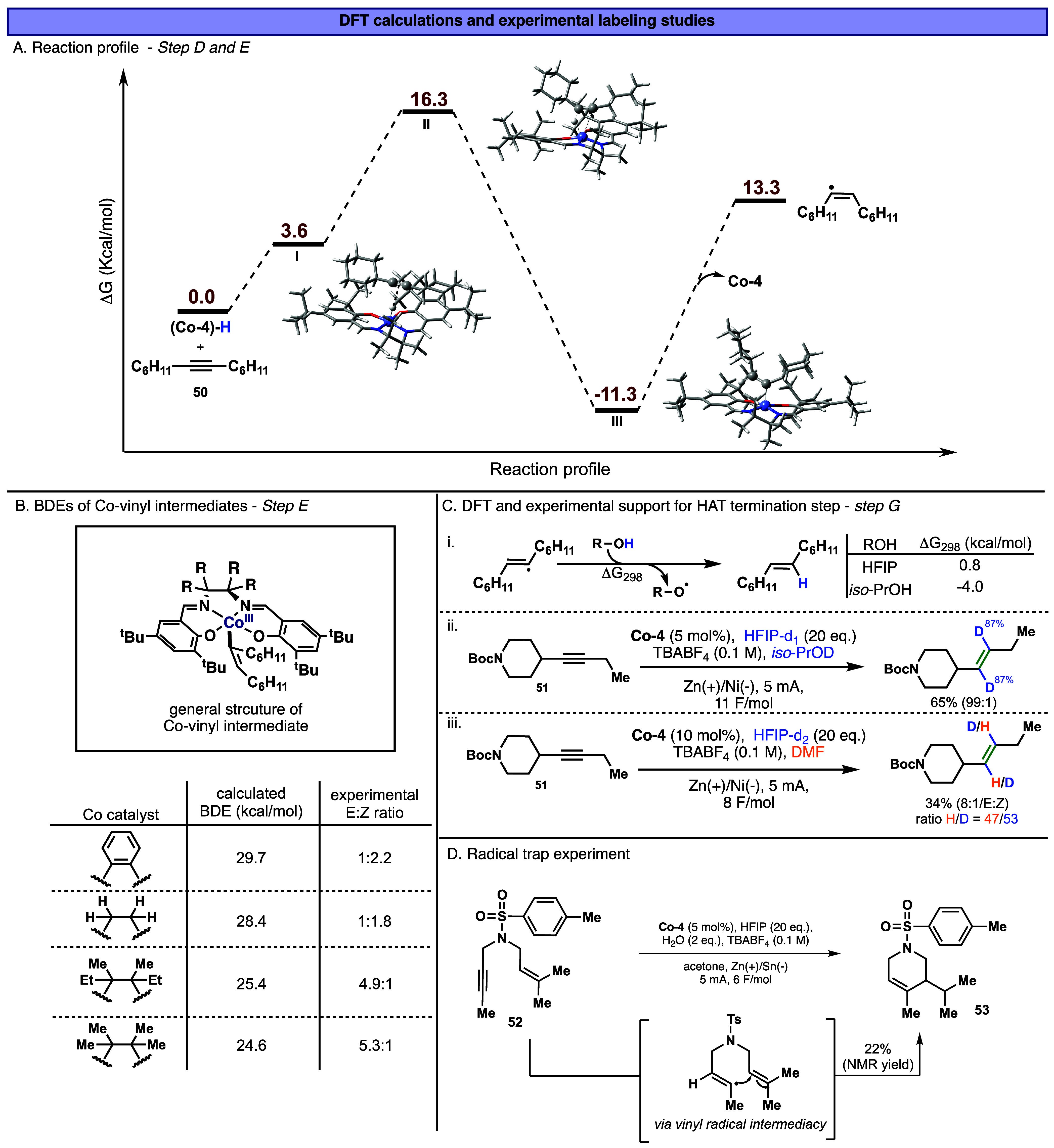
(A) DFT computation of Co-vinyl intermediate **III** formation. Method: SMD­(^*i*^PrOH)-revDSD-PBEP86-D4/CBS//PBE_D3BJ_/def2-SVP level of theory. (B) BDE analysis of vinyl-cobalt intermediates of varied cobalt complexes **Co-1, Co-2, Co-4, and** **Co-5**. (C) DFT calculations and deuterium labeling studies for the hydrogen abstraction as the terminating step G. (D) Radical trap experiment with enyne.

Next, the vinyl-Co­(III) intermediate **III** is proposed to undergo homolytic Co–C bond cleavage to form a vinyl radical (Figure 5A, step E) with a reaction energy of Δ*G*_298_ = 13.3 kcal/mol relative to the Co­(III)–H and alkyne and regenerate the **Co-4**. To understand this step, we evaluated the Co–C bond dissociation energies (BDEs) of various vinyl-Co intermediates using DFT analysis (Figure 6B) and correlated them with the observed *E*/*Z* selectivity taken from Figure 2 (entries 1–5). A clear trend is observed; catalysts with lower Co–C BDEs (e.g., **Co-4** and **Co-5**) favored *E*-alkene formation, while stronger Co–C bonds (e.g., **Co-1** and **Co-2**) are associated with increased formation of the Z-isomer. This suggests that stronger Co–C bonds may allow for a competitive protodemetalation pathway that does not involve vinyl dissociation and subsequent isomerization to the more stable E-isomer.

The generated *Z*-vinyl radical is then free to isomerize to the thermodynamically preferred E-geometry, similar to known vinyl radical behavior in Birch reductions and related systems.^58^ This *E*-vinyl radical subsequently undergoes hydrogen atom abstraction to afford the final alkene product. Vinyl radicals are highly reactive intermediates with a strong propensity for H atom abstraction. In our system, we determined that the two hydrogen atoms originate from different sources: one from HFIP (via the Co–H species) and the other from the solvent (*iso*-BuOH or *iso*-PrOH). This conclusion is supported by DFT calculations, which show that H atom abstraction from *iso*-PrOH is thermodynamically favorable (Δ*G*_298_ = −4.0 kcal/mol, Figure 6C­(i)). Furthermore, isotopic labeling studies revealed that when using deuterated *iso*-PrOH and deuterated HFIP (Figure 6C­(ii)), over 87% deuterium incorporation was observed. In contrast, replacing the deuterated *iso*-PrOH with dry, nondeuterated DMF while employing deuterated HFIP resulted in a roughly 1:1 ratio of H/D incorporation in the final product (Figure 6C­(iii)). The HMBC-NMR experiment (see Supporting Information, additional data section) confirms that hydrogen and deuterium are located on the same alkene molecule. This observation supports that one hydrogen is delivered via a cobalt-mediated pathway from HFIP, while the second is abstracted from the solvent itself, completing the semihydrogenation cycle. In the analogous alkene reduction reactions involving TM-HAT systems, similar radical abstraction steps, where a carbon-centered radical abstracts a hydrogen atom from a protic species, have also been proposed.^71,72^ It is worth noting that the Co­(III)–H species could, in principle, serve as the source of the second hydrogen incorporated into the alkene.^74^ This pathway is thermodynamically favorable (Δ*G* ≈ −50.3 kcal/mol, see Computations section in Supporting Information); however, the isotopic labeling results do not support its involvement in the hydrogen abstraction step G (Figure 6C). In contrast, changes in solvent environment, particularly switching from DMF to alcoholic solvents, can influence this specific radical step in the mechanism.

To further substantiate the involvement of vinyl radical intermediates, we conducted an intramolecular radical trapping experiment (Figure 6D). In this study, enyne **52** was subjected to the reaction conditions, affording the six-membered ring product **53**. This transformation is consistent with a pathway involving initial formation of a vinyl radical intermediate, followed by intramolecular C–C bond formation and subsequent hydrogen atom abstraction.

Building on these promising results, we envisioned a complementary strategy whereby the trans-selective reduction of alkynes could be achieved through an oxidative pathway using traditional Co-HAT chemistry.^60–64^ In contrast to the electrochemical reduction, which likely proceeds via low-valent cobalt intermediates (as discussed in Figure 5), this oxidative variant is presumed to operate through high-valent cobalt species, highlighting a mechanistic divergence rooted in the distinct modes of Co–H formation. Such dual reactivity, where the same catalyst system can be employed in both reductive and oxidative regimes, is exceptionally rare and underscores the versatility of cobalt-hydride-based catalysis. Guided by the same selectivity principles as in our electrochemical system, we discovered that the employment of the **Co-5** catalyst in combination with phenylsilane (PhSiH_3_) as the hydride source and *tert*-butyl hydroperoxide (TBHP) as the terminal oxidant (Figure 7) leads to trans-selective reduction of alkynes. Under these conditions, the alkene product **2** was obtained in excellent yield (93%) and high trans-selectivity (*E*/*Z* = 97:3). This result demonstrates that oxidative trans-selective reduction is not only feasible but also efficient. Importantly, this oxidative Co–H-based method exhibits a complementary substrate scope compared to the electrochemical approach. Several substrates that failed under electrochemical conditions were successfully converted under the chemical protocol with good yields and selectivity. For example, azide-containing alkyne **35**, which is challenging to reduce under electro-reductive conditions, was well tolerated under the oxidative chemical conditions to reveal compound **54** in 56% isolated yield with a 90:10 *E*/*Z* ratio (Figure 7). Likewise, propargylic amine-containing alkyne, which did not perform well under electrochemical conditions, underwent smooth conversion under the oxidative protocol, affording the corresponding *E*-alkene **55** with good yield and selectivity, 61% (*E*/*Z* = 97:3). On the other hand, the oxidative method does exhibit certain limitations. It showed lower efficiency with electron-rich alkynes (suspected decomposition, or oxidation, of the electron-rich alkene products **3**, **7** and **10**) and displayed moderate trans-selectivity when applied to internal aliphatic alkynes **11** and **15**. These observations suggest that subtle electronic and steric factors significantly influence the outcome depending on the mechanistic pathway. Ongoing studies in our group are focused on unraveling the mechanistic basis for these differences in reactivity and selectivity between the reductive and oxidative systems.

**Figure 7:**
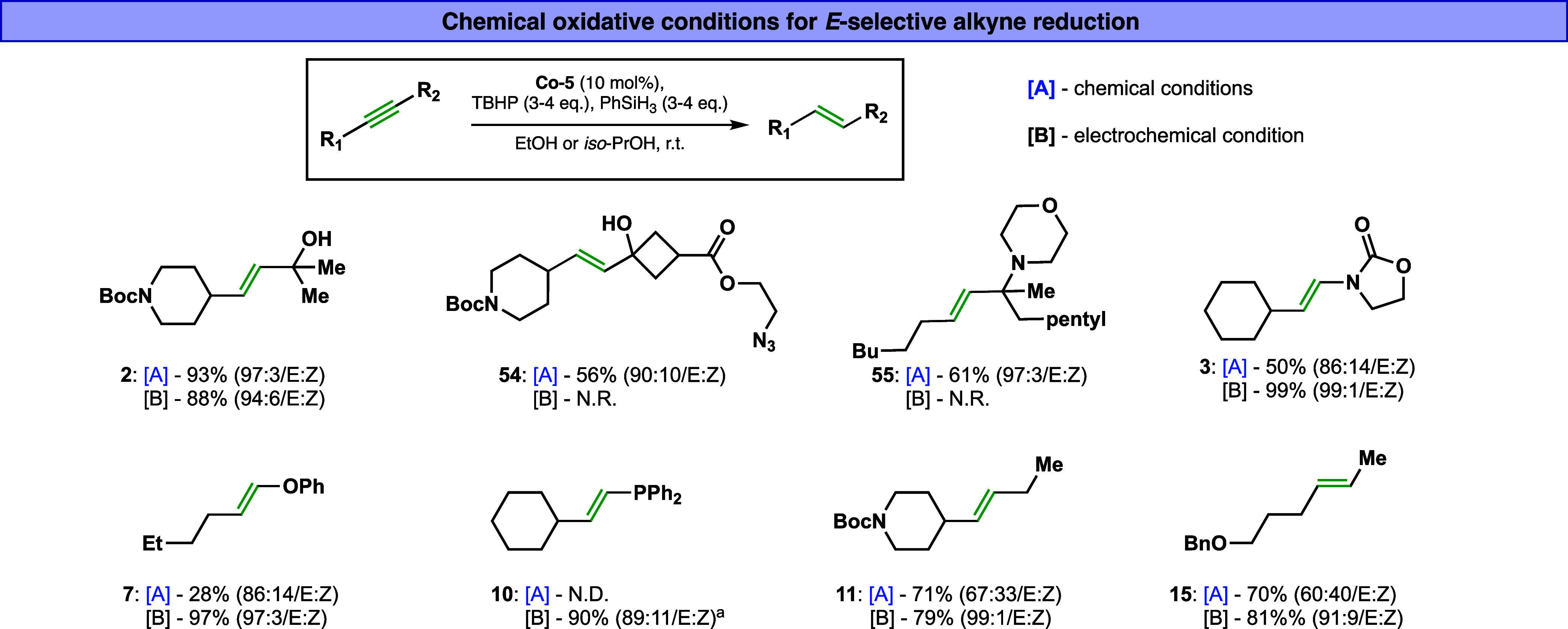
Chemical conditions for the cobalt-hydride mediated *trans*-reduction of alkyne. N.R.—no reaction. N.D.—not detected. ^a^ 20% alkane.

## Conclusion

In conclusion, we have established a fundamentally distinct cobalt-catalyzed strategy for the E-selective semireduction of internal alkynes, leveraging a one-electron radical mechanism. This first-row transition metal system exhibits a broad substrate scope and exceptional functional group tolerance, enabling efficient synthesis of *E*-alkenes from a wide array of sterically and electronically diverse alkynes. The synthetic utility is further underscored by the facile incorporation of isotopic labels (e.g., trans-deuteration of alkynes) and by remarkable chemoselectivity in diyne systems, selectively reducing one alkyne moiety in the presence of another. Moreover, a complementary oxidation-driven protocol was developed to address unreactive substrates under electrochemical conditions, thus expanding the range of alkynes amenable to this trans-selective reduction. Mechanistic studies, based on cyclic voltammetry, UV–vis spectroelectrochemistry, and DFT calculations, support dual catalytic pathways involving electrochemical generation of a Co–H species and a subsequent organometallic insertion sequence, thereby validating the one-electron logic and distinguishing this mechanism from traditional Z-selective routes. Collectively, these findings define a new paradigm for accessing *E*-alkenes via first-row transition-metal catalysis, and we anticipate that the mechanistic insights and one-electron approach demonstrated here will inspire further innovations in sustainable hydrogenation catalysis.

## Supplementary Material





## Notes

Provided are full details of the experimental procedures for the synthesis of all new substances, together with characterization data, including computational details and Cartesian coordinates of the optimized structures (PDF)
Geometry of complexes (TXT)
