# Supplementary material for: One-Electron Approach for Trans-Selective Alkyne Semi-Reduction via Cobalt Catalysis
Source: J Am Chem Soc. 2025 Oct 28;147(45):41272–83. doi: 10.1021/jacs.5c07630 (PMC12616701; doi:10.1021/jacs.5c07630)
Supplement: Supplementary file 1 [file ja5c07630_si_001.pdf]

SUPPORTING INFORMATION

One-Electron Approach for *Trans*-Selective Alkyne Semi-Reduction via Cobalt Catalysis

Rakesh Mondal[#a], Lior Galmidi[#a], Avra Tzaguy[a], Tal Sason[a], Moran Feller[a], Mark A. Iron[b], Liat Avram[b], Ronny Neumann[a], Samer Gnaim[*a]

[a] Department of Molecular Chemistry and Materials Science, Weizmann Institute of Science, Rehovot 7610001, Israel.

[b] Department of Chemical Research Support, Weizmann Institute of Science, Rehovot 7610001, Israel.

# These authors contributed equally to this study.

TABLE OF CONTENTS

**1.**	**Experimental details**		**S6 – S88**
	1.1.	Methods and materials	S6
	1.2.	Electrochemical equipment	S7
	1.3.	Synthetic Procedures	
	Synthetic procedure and chemical analysis data of starting material (Compound **1**)		S8
	Synthetic procedure and chemical analysis data of starting material (Compound **51**)		S9
	Synthetic procedure and chemical analysis data of starting material (Compound **S1**)		S10
	Synthetic procedure and chemical analysis data of starting material (Compound **S2**)		S11
	Synthetic procedure and chemical analysis data of starting material (Compound **S3**)		S12
	Synthetic procedure and chemical analysis data of starting material (Compound **S4**)		S13
	Synthetic procedure and chemical analysis data of starting material (Compound **S5**)		S14
	Synthetic procedure and chemical analysis data of starting material (Compound **S6**)		S15
	Synthetic procedure and chemical analysis data of starting material (Compound **S7**)		S16
	Synthetic procedure and chemical analysis data of starting material (Compound **S8**)		S17
	Synthetic procedure and chemical analysis data of starting material (Compound **S9**)		S18
	Synthetic procedure and chemical analysis data of starting material (Compound **S10**)		S19
	Synthetic procedure and chemical analysis data of starting material (Compound **S11**)		S20
	Synthetic procedure and chemical analysis data of starting material (Compound **S12**)		S21
	Synthetic procedure and chemical analysis data of starting material (Compound **S13**)		S22
	Synthetic procedure and chemical analysis data of starting material (Compound **S14**)		S23
	Synthetic procedure and chemical analysis data of starting material (Compound **S15**)		S24
	Synthetic procedure and chemical analysis data of starting material (Compound **S16**)		S25
	Synthetic procedure and chemical analysis data of starting material (Compound **S17**)		S26

	Synthetic procedure and chemical analysis data of starting material (Compound **35**)	S27
	Synthetic procedure and chemical analysis data of starting material (Compound **S18**)	S28
	Synthetic procedure and chemical analysis data of starting material (Compound **49**)	S29
	Synthetic procedure and chemical analysis data of starting material (Compound **37**)	S30
	Synthetic procedure and chemical analysis data of starting material (Compound **43**)	S31
	Synthetic procedure and chemical analysis data of starting material (Compound **S19**)	S32
	Synthetic procedure and chemical analysis data of starting material (Compound **S20**)	S33
	Synthetic procedure and chemical analysis data of starting material (Compound **S21**)	S34
	Synthetic procedure and chemical analysis data of Salen ligand (Compound **S22**)	S35
	Synthetic procedure and chemical analysis data of Salen ligand (Compound **S23**)	S36
	Synthetic procedure and chemical analysis data of Salen ligand (Compound **S24**)	S37
	Synthetic procedure of catalyst **Co-5**	S38
	**General procedure A**: cobalt electro-catalyzed *trans*-selective hydrogenation of alkynes	S39
	**General procedure B**: General procedures for *trans*-selective deuteration of alkynes	S40
	Graphical guide for electrochemical reaction set-up	S41 – S43
	**General procedure C**: Chemical conditions for *trans*-selective hydrogenation of alkynes	S44
	Catalytic procedure and chemical analysis data of *E*-alkene (Compound **2**)	S45
	Catalytic procedure and chemical analysis data of *E*-alkene (Compound **3**)	S46
	Catalytic procedure and chemical analysis data of *E*-alkene (Compound **4**)	S47
	Catalytic procedure and chemical analysis data of *E*-alkene (Compound **5**)	S48
	Catalytic procedure and chemical analysis data of *E*-alkene (Compound **6**)	S49
	Catalytic procedure and chemical analysis data of *E*-alkene (Compound **7**)	S50
	Catalytic procedure and chemical analysis data of *E*-alkene (Compound **8**)	S51

	Catalytic procedure and chemical analysis data of *E*-alkene (Compound **9**)	S52
	Catalytic procedure and chemical analysis data of *E*-alkene (Compound **10**)	S53
	Catalytic procedure and chemical analysis data of *E*-alkene (Compound **11**)	S54
	Catalytic procedure and chemical analysis data of *E*-alkene (Compound **12**)	S55
	Catalytic procedure and chemical analysis data of *E*-alkene (Compound **13**)	S56
	Catalytic procedure and chemical analysis data of *E*-alkene (Compound **14**)	S57
	Catalytic procedure and chemical analysis data of *E*-alkene (Compound **15**)	S58
	Catalytic procedure and chemical analysis data of *E*-alkene (Compound **16**)	S59
	Catalytic procedure and chemical analysis data of *E*-alkene (Compound **17**)	S60
	Catalytic procedure and chemical analysis data of *E*-alkene (Compound **18**)	S61
	Catalytic procedure and chemical analysis data of *E*-alkene (Compound **19**)	S62
	Catalytic procedure and chemical analysis data of *E*-alkene (Compound **20**)	S63
	Catalytic procedure and chemical analysis data of *E*-alkene (Compound **21**)	S64
	Catalytic procedure and chemical analysis data of *E*-alkene (Compound **22**)	S65
	Catalytic procedure and chemical analysis data of *E*-alkene (Compound **23**)	S66
	Catalytic procedure and chemical analysis data of *E*-alkene (Compound **24**)	S67
	Catalytic procedure and chemical analysis data of *E*-alkene (Compound **25**)	S68
	Catalytic procedure and chemical analysis data of *E*-alkene (Compound **26**)	S69
	Catalytic procedure and chemical analysis data of *E*-alkene (Compound **27**)	S70
	Catalytic procedure and chemical analysis data of *E*-alkene (Compound **28**)	S71
	Catalytic procedure and chemical analysis data of *E*-alkene (Compound **29**)	S72
	Catalytic procedure and chemical analysis data of *E*-alkene (Compound **30**)	S73

	Catalytic procedure and chemical analysis data of *E*-alkene (Compound **31**)	S74
	Catalytic procedure and chemical analysis data of *E*-alkene (Compound **32**)	S75
	Catalytic procedure and chemical analysis data of deuterated *E*-alkene (Compound **38**)	S76
	Catalytic procedure and chemical analysis data of deuterated *E*-alkene (Compound **39**)	S77
	Catalytic procedure and chemical analysis data of deuterated *E*-alkene (Compound **40**)	S78
	Catalytic procedure and chemical analysis data of deuterated *E*-alkene (Compound **41**)	S79
	Catalytic procedure and chemical analysis data of deuterated *E*-alkene (Compound **42**)	S80
	Chemoselective hydrogenation and chemical analysis data of *E*-alkene (Compound **44**)	S81
	Procedure for the TIPS deprotection and chemical analysis data of Compound **45**	S82
	Chemoselective hydrogenation and chemical analysis data of *E*-alkene (Compound **46**)	S83
	Chemoselective hydrogenation and chemical analysis data of *E*-alkene (Compound **47**)	S84
	Chemoselective hydrogenation and chemical analysis data of *E*-alkene (Compound **48**)	S85
	Catalytic procedure and chemical analysis data of *E*-alkene (Compound **53**)	S86
	Catalytic procedure and chemical analysis data of *E*-alkene (Compound **54**)	S87
	Catalytic procedure and chemical analysis data of *E*-alkene (Compound **55**)	S88
**2.**	**Additional data**	**S89 – S103**
**3.**	**Computational study details**	**S104 – S110**
**4.**	**NMR spectra**	**S111 – S272**
**5.**	**References**	**S273 – S276**

1. Experimental details

1.1. Methods and materials

Reagents were purchased at the highest commercial quality and used without further purification unless otherwise stated. Isolated yields refer to chromatographically and spectroscopically (^{1}H-NMR) homogeneous material unless otherwise stated. Acetonitrile (MeCN), chloroform ($CHCl_3$), dichloromethane (DCM), *N,N*-dimethylformamide (DMF), *n*-pentane, *n*-hexane, 1,2-dimethoxyethane (DME), benzene, toluene, dimethylsulfoxide (DMSO), diethyl ether, and tetrahydrofuran (THF) were obtained by passing the previously degassed solvents through an activated alumina column. For determination of ^{1}H-NMR yields, cyclohexane carboxaldehyde, nitromethane, 1,4-bis(trifluoromethyl)benzene, or 1,3,5-trimethoxybenzene were used as internal standards (automatic baseline correction was applied). Reactions were monitored by thin-layer chromatography (TLC) carried out on 0.25 mm E. Merck silica plates (60 F_{254}), using short-wave UV light (254 nm) for visualization, and *p*-anisaldehyde or potassium permanganate as developing agents. Flash column chromatography was performed using E. Merck silica gel (60, particle size 0.043–0.063 mm) or basic Al_2O_3. NMR spectra were recorded on Bruker Avance NEO-300, Avance NEO-400, Avance III HD-500, and Avance III-800, and chemical shifts for ^{1}H- and ^{13}C-NMR are reported relative to the solvent peaks (7.26 ppm for ^{1}H-NMR in $CDCl_3$, 77.16 ppm for ^{13}C-NMR in $CDCl_3$). The following abbreviations were used to explain NMR peak multiplicities: s = singlet, d = doublet, t = triplet, q = quartet, p = pentet, m = multiplet, br = broad. High–resolution mass spectra (HRMS) were recorded on an Agilent LC/MSD TOF mass spectrometer using an ESI ion source, a Waters LC-TOF (I-Class and G2-XS) mass spectrometer using ESI or APCI ion sources, and a Thermo Fisher Scientific LTQ Orbitrap XL mass spectrometer using an ESI ion source. GC-MS (EI) was recorded on Agilent 7820A GC systems and 5975 Series MSD using *n*-decane as an internal standard. Optical rotation data were recorded on an Anton Paar 100 Modular Circular Polarimeter.

1.2. Electrochemistry equipment

The tin (catalog number: 0040002857), nickel-foam (catalog number: 0040002861), zinc (catalog number: 0040002850), and magnesium (catalog number: 0040002848) electrodes used in this work were bought from IKA (for 0.2−0.5 mmol scale). Other electrodes used or tested in this work were obtained from IKA (https://www.ika.com/en). For experiments using an ElectraSyn vial, the dimensions of the electrodes were approximately W7 × D1.5 × H55 mm (with the submerged exterior surface of the electrode approximately W7 × D1.5 × H20 mm) unless otherwise stated.

1.3. Synthetic procedures

Starting materials synthesis

<u>**Compound 1**</u>

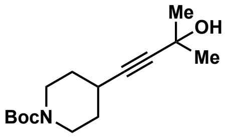

A solution of *tert*-butyl 4-ethynylpiperidine-1-carboxylate (1.046 mg, 5.0 mmol) in THF (10 mL) in a flame-dried flask was cooled to −78 °C, *n*-BuLi (1.5 eq., 1.6M hexane solution, 4.6 mL, 7.5 mmol) was added, and the mixture was stirred at the same temperature for 30 min. Dry acetone (2.0 eq., 740 μL, 10.0 mmol) was added, and the mixture was warmed to room temperature. After 12 h of stirring, the reaction was quenched by the addition of 10 mL of distilled water, and the aqueous solution was extracted with EtOAc (3 × 10 mL), followed by washing with saturated NH_4Cl and brine solution and dried over Na_2SO_4. After filtration and evaporation of the solvent, a residue was obtained, which was purified by column chromatography (Hexane/EtOAc,100:0 - 70:30) in 67% yield (895.0 mg, 3.35 mmol) as a white solid.

^{1}H-NMR (300 MHz, $CDCl_3$) δ (ppm): 3.67 (ddd, J = 13.3, 6.7, 3.7 Hz, 2H), 3.19 (ddd, J = 13.4, 8.4, 3.4 Hz, 2H), 2.58 (dt, J = 12.1, 4.0 Hz, 1H), 2.02 (s, 1H), 1.79 – 1.71 (m, 2H), 1.59 – 1.53 (m, 2H), 1.51 (s, 6H), 1.4 6 (s, 9H).

^{13}C-NMR (75 MHz, $CDCl_3$) δ (ppm): 154.78, 86.67, 84.13, 79.48, 65.19, 42.13, 31.78, 31.37, 28.45, 26.79.

Physical State: white solid.

HRMS (ESI-TOF): calc'd for $C_{15}H_{25}NNaO_3$ $[M+Na]^+$: 290.1732; found 290.1734.

TLC: R_f = 0.5 (7:3 Hexane: EtOAc).

__**Compound 51**__

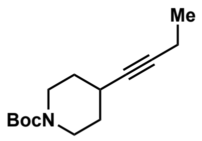

The preparation of the following starting material was carried out according to the literature procedure.[1]

^{1}H-NMR (400 MHz, $CDCl_3$) δ (ppm): 3.50 – 3.46 (m, 2H), 3.02 – 2.99 (m, 2H), 2.35 (dd, *J* = 3.7, 1.9 Hz, 1H), 1.99 – 1.97 (m, 2H), 1.54 (dd, *J* = 12.7, 3.2 Hz, 2H), 1.37 – 1.32 (m, 2H), 1.27 (s, 9H), 0.95 – 0.91 (m, 3H).

^{13}C-NMR (101 MHz, $CDCl_3$) δ (ppm): 154.49, 82.87, 81.18, 78.98, 42.00, 31.64, 28.24, 26.82, 14.18, 12.18.

Physical State: colorless oil.

GC\MS (EI): 57 (100%), 108 (26.3%), 152.1 (21.2%), 181 (22.3%), 237.2 (1 %).

TLC: R_f = 0.5 (49:1 Hexane: EtOAc).

Compound S1

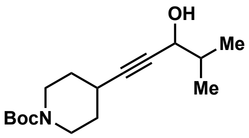

A solution of *tert*-butyl 4-ethynylpiperidine-1-carboxylate (1.046 g, 5.0 mmol) in THF (10 mL) in a flame-dried flask was cooled to −78 °C, *n*-BuLi (1.5 eq., 1.6M hexane solution, 4.6 mL, 7.5 mmol) was added, and the mixture was stirred at the same temperature for 30 min. *iso*-butyraldehyde (1.2 eq., 547 µL, 6.0 mmol) was added, and the mixture was warmed to room temperature. After 12 h of stirring, the reaction was quenched by the addition of 10 mL of distilled water, and the aqueous solution was extracted with EtOAc (3 × 10 mL), followed by washing with saturated NH_4Cl and brine solution and dried over Na_2SO_4. After filtration, evaporation of the solvent gave a residue, which was purified by column chromatography (Hexane/EtOAc,100:0 - 70:30) in 78% yield (1.1 g, 3.9 mmol) as a colorless oil.

^{1}H-NMR (300 MHz, $CDCl_3$) δ (ppm): 4.19 (td, *J* = 5.6, 1.8 Hz, 1H), 3.68 (ddd, *J* = 13.1, 6.7, 3.6 Hz, 2H), 3.23 (ddd, *J* = 13.4, 8.3, 3.4 Hz, 2H), 2.64 (dtd, *J* = 10.2, 4.0, 2.0 Hz, 1H), 1.92 – 1.74 (m, 4H), 1.68 – 1.52 (m, 2H), 1.47 (s, 9H), 1.00 (dd, *J* = 6.7, 5.3 Hz, 6H).
^{13}C-NMR (75 MHz, $CDCl_3$) δ (ppm): 154.77, 87.86, 81.31, 79.50, 68.04, 34.65, 31.40, 28.45, 26.93, 18.18, 17.41.
Physical State: colorless oil.
HRMS (ESI-TOF): calc'd for $C_{16}H_{27}NNaO_3$ $[M+Na]^+$: 304.1889; found 304.1885.
TLC: R_f = 0.5 (7:3 Hexane: EtOAc).

<u>Compound S2</u>

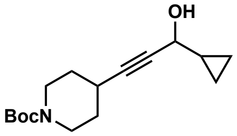

A solution of *tert*-butyl 4-ethynylpiperidine-1-carboxylate (1.046 g, 5.0 mmol) in THF (10 mL) in a flame-dried flask was cooled to −78 °C, *n*-BuLi (1.5 eq., 1.6M hexane solution, 4.6 mL, 7.5 mmol) was added, and the mixture was stirred at the same temperature for 30 min. cyclopropanecarbaldehyde (1.2 eq., 448 µL, 6.0 mmol) was added, and the mixture was warmed to room temperature. After 12 h of stirring, the reaction was quenched by the addition of 10 mL of distilled water, and the aqueous solution was extracted with EtOAc (3 × 10 mL), followed by washing with saturated NH_4Cl and brine solution and dried over Na_2SO_4. After filtration, evaporation of the solvent gave a residue, which was purified by column chromatography (Hexane/EtOAc,100:0 - 70:30) in 72% yield (1.0 g, 3.6 mmol) as a colorless oil.

^{1}H-NMR (300 MHz, $CDCl_3$) δ (ppm): 4.23 (s, 1H), 3.65 – 3.59 (m, 2H), 3.21 – 3.12 (m, 2H), 2.57 – 2.44 (m, 2H), 1.76 – 1.70 (m, 2H), 1.56 – 1.46 (m, 2H), 1.43 (s, 9H), 1.23 – 1.16 (m,1H), 0.52 – 0.36 (m, 4H).
^{13}C-NMR (75 MHz, $CDCl_3$) δ (ppm): 154.76, 87.39, 80.20, 79.53, 65.71, 31.33, 28.44, 26.90, 17.18, 3.18, 1.35.
Physical State: colorless oil.
HRMS (ESI-TOF): calc'd for $C_{16}H_{25}NNaO_3$ $[M+Na]^+$: 302.1732; found 302.1729.
TLC: R_f = 0.6 (3:7 EtOAc: Hexane).

Compound S3

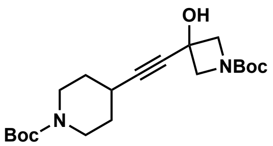

A solution of *tert*-butyl 4-ethynylpiperidine-1-carboxylate (1.046 g, 5.0 mmol) in THF (10 mL) in a flame-dried flask was cooled to −78 °C, *n*-BuLi (1.5 eq., 1.6M hexane solution, 4.68 mL, 7.5 mmol) was added, and the mixture was stirred at the same temperature for 30 min. *tert*-butyl 3-oxoazetidine-1-carboxylate (1.2 eq., 1.02 g, 6.0 mmol) was added, and the mixture was heated to 50 ºC temperature. After 12 h of stirring, the reaction was quenched by the addition of 10 mL of distilled water, and the aqueous solution was extracted with EtOAc (3 x 10 mL), followed by washing with saturated NH_4Cl and brine solution and dried over Na_2SO_4. After filtration, evaporation of the solvent gave a residue, which was purified by column chromatography (Hexane:EtOAc,100:0 – 70:30) in 56% yield (1.074 g, 2.82 mmol) as a white solid.

^{1}H-NMR (400 MHz, $CDCl_3$) δ (ppm): 4.12 (d, *J* = 9.0 Hz, 2H), 4.00 (d, *J* = 9.0 Hz, 2H), 3.70 – 3.66 (m, 2H), 3.27 (s, 1H), 3.18 – 3.12 (m, 2H), 2.61 (dt, *J* = 12.4, 4.0 Hz, 1H), 1.78 – 1.74 (m, 3H), 1.44 (s, 9H), 1.43 (s, 9H).
^{13}C-NMR (101 MHz, $CDCl_3$) δ (ppm): 156.2, 154.8, 88.3, 81.9, 79.9, 79.7, 64.5, 62.2, 31.11, 28.4, 28.4, 27.0.
Physical State: white solid.
HRMS (ESI-TOF): calc'd for $C_{20}H_{32}N_2O_5Na$ $[M+Na]^+$: 403.2209; found 403.2206.
TLC: R_f = 0.5 (3:7 EtOAc:Hexane).

<u>Compound S4</u>

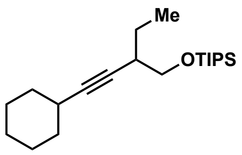

An oven-dried 30 mL re-sealable screwcap test tube equipped with a Teflon-coated magnetic stirrer bar was charged with anhydrous $FeBr_2$ (98% purity, 44 mg, 0.20 mmol, 0.10 equiv), (2-iodobutoxy)tri-*iso*-propylsilane (2.0 mmol, 1.0 equiv), and NMP solvent (8.0 mL), followed by the addition of freshly prepared (cyclohexylethynyl)magnesium bromide solution[2] (0.5M in THF, 6.0 mL, 1.5 equiv). The reaction mixture was stirred at room temperature for 16 h to form a deep brown or black solution. After 16 h of stirring, the reaction was quenched by adding 10 mL of distilled water, and the aqueous solution was extracted with EtOAc (3 × 10 mL), followed by washing with a brine solution and drying over Na_2SO_4. After filtration, evaporation of the solvent gave a residue, which was purified by column chromatography (Hex/EtOAc,100:0 – 90:10) in 65% yield (451 mg, 1.34 mmol) as a colorless oil.

^{1}H-NMR (300 MHz, $CDCl_3$) δ (ppm): 3.79 – 3.74 (m, 1H), 3.61 – 3.56 (m, 1H), 2.51 – 2.34 (m, 2H), 1.80 – 1.69 (m, 5H), 1.48 – 1.30 (m, 7H), 1.15 – 1.00 (m, 24H).
^{13}C-NMR (75 MHz, $CDCl_3$) δ (ppm): 86.49, 80.76, 66.22, 36.65, 33.15, 29.13, 26.02, 24.87, 24.50, 18.00, 12.01, 11.35.
Physical State: colorless oil.
HRMS (ESI-TOF): calc'd for $C_{21}H_{41}OSi$ $[M+H]^+$: 337.2927; found 337.2929.
TLC: R_f = 0.5 (100% Hexane).

Compound S5

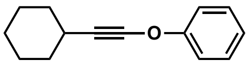

The preparation of the following starting material was carried out according to the literature procedure.[2]

^{1}H-NMR (300 MHz, $CDCl_3$) δ (ppm): 7.37 (tt, *J* = 7.3, 2.0 Hz, 2H), 7.30 – 7.27 (m, 2H), 7.17 – 7.10 (m, 1H), 2.56 – 2.47 (m, 1H), 1.90 – 1.72 (m, 4H), 1.58 – 1.43 (m, 3H), 1.40 – 1.28 (m, 3H).
^{13}C-NMR (101 MHz, $CDCl_3$) δ (ppm): 156.47, 129.53, 123.85, 114.81, 83.68, 49.00, 33.48, 27.96, 25.99, 24.98.
Physical State: colorless oil.
GC\MS (EI): 32.0 (100%), 40.0 (6.3%), 200.1 (1.8%).
TLC: R_f = 0.4 (100% Hexane).

Compound S6

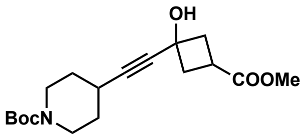

A solution of *tert*-butyl 4-ethynylpiperidine-1-carboxylate (1.046 g, 5.0 mmol) in THF (10 mL) in a flame-dried flask was cooled to −78 °C, *n*-BuLi (1.5 eq., 1.6M hexane solution, 4.6 mL, 7.5 mmol) was added, and the mixture was stirred at the same temperature for 30 min. Methyl-3-oxocyclobutane-1-carboxylate (1.2 eq., 0.769 g, 6.0 mmol) was added, and the mixture was warmed to room temperature. After 12 h of stirring, the reaction was quenched by the addition of 10 mL of distilled water, and the aqueous solution was extracted with EtOAc (3 × 10 mL), followed by washing with saturated NH_4Cl and brine solution and dried over Na_2SO_4. After filtration, evaporation of the solvent gave a residue, which was purified by column chromatography (Hexane:EtOAc,100:0 – 70:30) in 72% yield (1.2 g, 3.6 mmol) as a colorless oil.

^{1}H-NMR (300 MHz, $CDCl_3$) δ (ppm): 3.72 – 3.68 (m, 4H), 3.21 (ddd, *J* = 13.3, 8.5, 3.4 Hz, 2H), 2.96 – 2.89 (m, 1H), 2.70 – 2.63 (m, 3H), 2.54 – 2.50 (m, 2H), 1.81 – 1.77 (m, 2H), 1.66 – 1.57 (m, 3H), 1.48 (s, 9H).

^{13}C-NMR (101 MHz, $CDCl_3$) δ (ppm): 175.31, 154.77, 86.27, 84.24, 79.59, 64.29, 52.01, 42.49, 31.28, 29.41, 28.44, 26.91.

Physical State: white solid.

HRMS (ESI-TOF): calc'd for $C_{18}H_{27}NNaO_5$ $[M+Na]^+$: 360.1787; found 360.1776.

TLC: R_f = 0.5 (30% EtOAc, 70% Hexane).

Compound S7

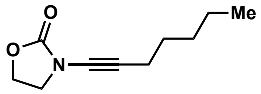

The preparation of the following starting material was carried out according to the literature procedure.[3]

^{1}H-NMR (300 MHz, $CDCl_3$) δ (ppm): 4.41 – 4.35 (m, 2H), 3.87 – 3.81 (m, 2H), 2.25 (t, J = 7.1 Hz, 2H), 1.54 – 1.44 (m, 2H), 1.37 – 1.21 (m, 4H), 0.86 (t, J = 7.1 Hz, 3H).
^{13}C-NMR (101 MHz, $CDCl_3$) δ (ppm): 156.73, 71.09, 70.05, 62.91, 47.06, 30.98, 28.46, 22.14, 18.32, 13.91.
Physical State: colourless oil.
GC\MS (EI): 32.0 (100%), 126.0 (9.1 %), 166.0 (2.3%), 181.0 (1.0%).
TLC: R_f = 0.5 (10% EtOAc in Hexane).

Compound S8

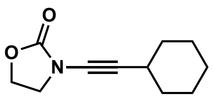

The preparation of the following starting material was carried out according to the literature procedure.[4]

^{1}H-NMR (300 MHz, $CDCl_3$) δ (ppm): 4.42 (dd, J = 8.7, 7.3 Hz, 2H), 3.88 (dd, J = 8.7, 7.3 Hz, 2H), 2.54 – 2.47 (m, 1H), 1.84 – 1.81 (m, 2H), 1.73 – 1.66 (m, 2H), 1.54 – 1.41 (m, 3H), 1.36 – 1.27 (m, 3H).

^{13}C-NMR (101 MHz, $CDCl_3$) δ (ppm): 156.54, 75.09, 70.26, 62.72, 47.18, 32.78, 28.83, 25.83, 24.92.

Physical State: light yellow oil.

GC\MS (EI): 32.0 (100), 164.1 (15.4%), 193.1 (1.3%).

TLC: R_f = 0.5 (20% EtOAc in Hexane).

Compound S9

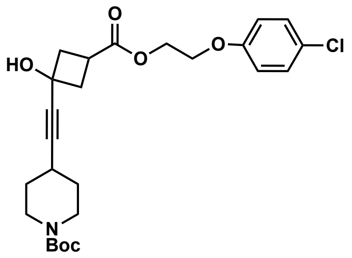

3-((1-(*tert*-butoxycarbonyl)piperidin-4-yl)ethynyl)-3-hydroxycyclobutane-1-carboxylic acid (277mg, 0.85 mmol), 1-(2-bromoethoxy)-4-chlorobenzene (1.0 eq., 200 mg, 0.85 mmol), sodium iodide (1.0 eq., 127 mg, 0.85 mmol) and K_2CO_3 (1.5 eq., 176 mg, 1.27 mmol) were taken in a flame-dried round-bottom flask. 5.0 mL of dry and degassed DMF was added and stirred for 18 h at 80 ºC under a nitrogen atmosphere. Afterward, the reaction was quenched by adding 10 mL of distilled water, and the aqueous solution was extracted with diethyl ether (3 × 20 mL). The combined organic phases were washed with brine solution and dried over Na_2SO_4. After filtration, evaporation of the solvent gave a brown-coloured residue, which was purified by column chromatography (Hexane:EtOAc,100:0 - 60:40) in 40% yield (162 mg, 0.34 mmol) as a light-yellow oil.

^{1}H-NMR (300 MHz, $CDCl_3$) δ (ppm): 7.27 – 7.24 (m, 2H), 6.88 – 6.84 (m, 2H), 4.47 – 4.44 (m, 2H), 4.18 – 4.14 (m, 2H), 4.09 – 3.96 (m, 1H), 3.73 – 3.65 (m, 2H), 3.24 – 3.16 (m, 2H), 3.03 – 2.91 (m, 1H), 2.72 – 2.49 (m, 5H), 1.82 – 1.74 (m, 2H), 1.63 – 1.51 (m, 2H), 1.47 (s, 9H).

^{13}C-NMR (101 MHz, $CDCl_3$) δ (ppm): 174.70, 157.06, 154.76, 129.42, 126.17, 115.93, 86.45, 84.10, 79.59, 69.52, 66.19, 64.33, 63.03, 61.37, 42.46, 31.27, 29.47, 28.45, 26.92.

Physical State: light yellow oil.

HRMS (ESI-TOF): calc'd for $C_{25}H_{32}ClNNaO_6$ $[M+Na]^+$: 500.1816; found 500.1813.

TLC: R_f = 0.5 (40% EtOAc in Hexane).

Compound S10

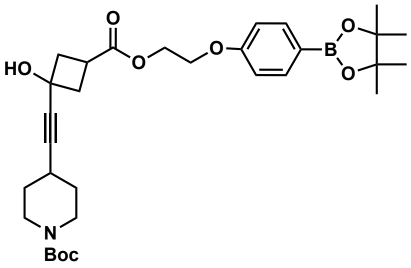

3-((1-(*tert*-butoxycarbonyl)piperidin-4-yl)ethynyl)-3-hydroxycyclobutane-1-carboxylic acid (270 mg, 0.80 mmol), 2-(4-(2-bromoethoxy)phenyl)-4,4,5,5-tetramethyl-1,3,2-dioxaborolane (1.0 eq., 260 mg, 0.80 mmol), sodium iodide (1.0 eq., 120 mg, 0.80 mmol) and K_2CO_3 (1.5 eq., 166 mg, 1.20 mmol) were taken in a flame-dried round-bottom flask. 5.0 mL of dry and degassed DMF was added and stirred for 24 h at 70 ºC under a nitrogen atmosphere. Afterward, the reaction was quenched by adding 10 mL of distilled water, and the aqueous solution was extracted with diethyl ether (3 × 20 mL). The combined organic phases were washed with brine solution and dried over Na_2SO_4. After filtration, evaporation of the solvent gave a brown-colored residue, which was purified by column chromatography (Hex/EtOAc,100:0 - 60:40) in 75% yield (340 mg, 0.59 mmol) as a white solid.

^{1}H-NMR (300 MHz, $CDCl_3$) δ (ppm): 7.75 (d, *J* = 8.6 Hz, 2H), 6.90 (d, *J* = 8.6 Hz, 2H), 4.47 – 4.44 (m, 2H), 4.21 – 4.18 (m, 2H), 3.71 – 3.63 (m, 2H), 3.23 – 3.14 (m, 2H), 2.99 – 2.93 (m, 1H), 2.70 – 2.48 (m, 5H), 1.80 – 1.73 (m, 2H), 1.61 – 1.50 (m, 2H), 1.46 (s, 9H), 1.34 (s, 12H).
^{13}C-NMR (101 MHz, $CDCl_3$) δ (ppm): 174.75, 160.99, 154.76, 136.58, 113.91, 86.19, 84.24, 83.63, 79.58, 65.62, 64.20, 63.09, 42.52, 31.26, 29.45, 28.45, 26.90, 24.86.
^{11}B NMR (96 MHz, $CDCl_3$) δ (ppm): 32.28.
Physical State: white solid.
HRMS (ESI-TOF): calc'd for $C_{31}H_{44}BNNaO_8$ $[M+Na]^+$: 592.3059; found 592.3058.
TLC: R_f = 0.5 (40% EtOAc in Hexane).

Compound S11

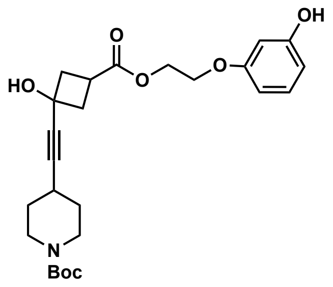

3-((1-(*tert*-butoxycarbonyl)piperidin-4-yl)ethynyl)-3-hydroxycyclobutane-1-carboxylic acid (300 mg, 0.90 mmol), 3-(2-bromoethoxy)phenol (1.0 eq., 195 mg, 0.90 mmol), sodium iodide (1.0 eq., 135 mg, 0.90 mmol) and K_2CO_3 (1.5 eq., 186 mg, 1.35 mmol) were taken in a flame-dried round-bottom flask. 5.0 mL of dry and degassed DMF was added and stirred for 18 h at 80 ºC under a nitrogen atmosphere. Afterward, the reaction was quenched by adding 10 mL of distilled water, and the aqueous solution was extracted with diethyl ether (3 × 20 mL). The combined organic phases were washed with brine solution and dried over Na_2SO_4. After filtration, evaporation of the solvent gave a brown-colored residue, which was purified by column chromatography (Hexane/EtOAc,100:0 - 50:50) in 73% yield (303 mg, 0.6 mmol) as a light-yellow oil.

^{1}H-NMR (300 MHz, $CDCl_3$) δ (ppm): 7.13 – 7.08 (m, 1H), 6.51 – 6.43 (m, 3H), 4.44 – 4.41 (m, 2H), 4.16 – 4.13 (m, 2H), 3.69 – 3.61 (m, 2H), 3.26 – 3.17 (m, 2H), 2.98 – 2.93 (m, 1H), 2.70 – 2.48 (m, 5H), 1.79 – 1.72 (m, 2H), 1.60 – 1.51 (m, 2H), 1.47 (s, 9H).
^{13}C-NMR (101 MHz, $CDCl_3$) δ (ppm): 174.97, 159.70, 157.59, 155.01, 130.10, 108.64, 106.52, 102.45, 86.19, 84.19, 80.02, 65.86, 64.25, 63.39, 42.54, 31.17, 29.52, 28.47, 26.78.
Physical State: Light yellow oil.
HRMS (ESI-TOF): calc'd for $C_{25}H_{33}NNaO_7$ $[M+Na]^+$: 482.2155; found 482.2148.
TLC: R_f = 0.5 (50% EtOAc and Hexane).

Compound S12

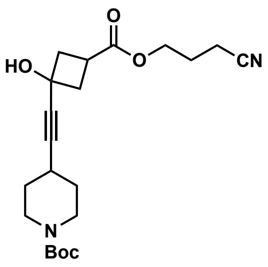

3-((1-(*tert*-butoxycarbonyl)piperidin-4-yl)ethynyl)-3-hydroxycyclobutane-1-carboxylic acid (300 mg, 0.90 mmol), 4-iodobutanenitrile (1.0 eq., 175 mg, 0.90 mmol), and K_2CO_3 (1.5 eq., 186 mg, 1.35 mmol) were taken in a flame-dried round-bottom flask. 5 mL of dry and degassed DMF was added and stirred for 18 h at 80 ºC under a nitrogen atmosphere. Afterward, the reaction was quenched by adding 10 mL of distilled water, and the aqueous solution was extracted with diethyl ether (3 × 20 mL). The combined organic phases were washed with brine solution and dried over Na_2SO_4. After filtration, evaporation of the solvent gave a brown-colored residue, which was purified by column chromatography (Hexane/EtOAc,100:0 - 60:40) in 58% yield (203 mg, 0.52 mmol) as a light-yellow oil.

^{1}H-NMR (300 MHz, $CDCl_3$) δ (ppm): 5.30 (s, 1H), 4.20 (t, J = 5.9 Hz, 2H), 3.70 – 3.62 (m, 2H), 3.23 – 3.15 (m, 2H), 2.97 – 2.85 (m, 1H), 2.69 – 2.57 (m, 3H), 2.55 – 2.44 (m, 4H), 2.06 – 1.97 (m, 2H), 1.80 – 1.72 (m, 2H), 1.61 – 1.50 (m, 2H), 1.45 (s, 9H).
^{13}C-NMR (75 MHz, $CDCl_3$) δ (ppm): 174.41, 154.76, 118.90, 86.20, 84.18, 79.60, 64.12, 62.61, 53.46, 42.53, 31.25, 29.39, 28.43, 26.88, 24.76, 14.35.
Physical State: light-yellow oil.
HRMS (ESI-TOF): calc'd for $C_{21}H_{30}NNaO_5$ $[M+Na]^+$: 413.2052; found 413.2038.
TLC: R_f = 0.5 (40% EtOAc in Hexane).

Compound S13

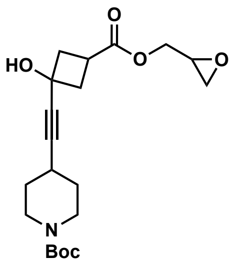

3-((1-(*tert*-butoxycarbonyl)piperidin-4-yl)ethynyl)-3-hydroxycyclobutane-1-carboxylic acid (100 mg, 0.31 mmol), DMAP (0.5 eq., 19.0 mg, 0.15 mmol), and EDC-HCl (2.5 eq., 148 mg, 0.77 mmol) were added in a flame-dried round-bottom flask. 3.0 mL of dry and degassed DCM was added and stirred at 0 ºC in an ice bath for 10 minutes. A solution of oxiran-2-ylmethanol (1.0 eq., 23 mg, 0.31 mmol) in 2 mL DCM was added dropwise at 0 ºC. The reaction mixture was stirred at the same temperature for 15 minutes, then warmed to room temperature and stirred for 2 h. Afterward, the reaction was quenched by adding 10 mL of distilled water, and the aqueous solution was extracted with diethyl ether (3 × 20 mL). The combined organic phases were washed with brine solution and dried over Na_2SO_4. After filtration, evaporation of the solvent gave a brown-colored residue, which was purified by column chromatography (Hexane/EtOAc,100:0 - 40:60) in 34% yield (40 mg, 0.1 mmol) as a colorless oil.

^{1}H-NMR (300 MHz, $CDCl_3$) δ (ppm): 4.46 (dd, J = 12.3, 3.0 Hz, 1H), 3.95 (dd, J = 12.3, 6.3 Hz, 1H), 3.74 – 3.66 (m, 2H), 3.25 – 3.16 (m, 3H), 3.01 – 2.86 (m, 2H), 3.01 – 2.86 (m, 2H), 2.73 – 2.50 (m, 7H), 1.83 – 1.75 (m, 2H), 1.64 – 1.52 (m, 2H), 1.48 (s, 9H).

^{13}C-NMR (101 MHz, $CDCl_3$) δ (ppm): 174.46, 154.76, 86.47, 84.09, 79.59, 65.30, 64.31, 49.27, 44.62, 42.48, 31.28, 29.39, 28.45, 26.93.

Physical State: colorless oil.

HRMS (ESI-TOF): calc'd for $C_{20}H_{29}NNaO_6$ $[M+Na]^+$: 402.1893; found 402.1881.

TLC: R_f = 0.5 (60% EtOAc in Hexane).

Compound S14

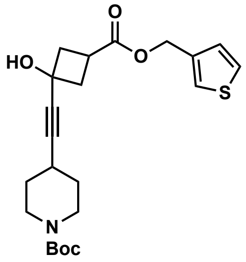

3-((1-(*tert*-butoxycarbonyl)piperidin-4-yl)ethynyl)-3-hydroxycyclobutane-1-carboxylic acid (239 mg, 0.74 mmol), 3-(bromomethyl)thiophene (1.0 eq., 131 mg, 0.74 mmol), sodium iodide (1.0 eq., 111 mg, 0.74 mmol) and K_2CO_3 (1.5 eq., 152 mg, 1.1 mmol) were taken in a flame-dried round-bottom flask. 5 mL of dry and degassed DMF was added and stirred for 18 h at 70 ºC under a nitrogen atmosphere. Afterward, the reaction was quenched by adding 10 mL of distilled water, and the aqueous solution was extracted with diethyl ether (3 × 20 mL). The combined organic phases were washed with brine solution and dried over Na_2SO_4. After filtration, evaporation of the solvent gave a brown-colored residue, which was purified by column chromatography (Hexane/EtOAc,100:0 - 50:50) in 80% yield (248 mg, 0.59 mmol) as a light-yellow oil.

^{1}H-NMR (300 MHz, $CDCl_3$) δ (ppm): 7.31 – 7.29 (m, *J* = 4.7, 2.9 Hz, 2H), 7.07 (dd, J = 4.6, 1.5 Hz, 1H), 5.12 (s, 2H), 3.68 – 3.61 (m, 2H), 3.23 – 3.14 (m, 2H), 2.99 – 2.87 (m, 1H), 2.68 – 2.47 (m, 5H), 1.78 – 1.72 (m, 2H), 1.57 – 1.53 (m, 2H), 1.45 (s, 9H), 1.25 – 1.18 (m, 1H).
^{13}C-NMR (101 MHz, $CDCl_3$) δ (ppm): 174.56, 154.75, 136.58, 127.50, 126.31, 124.39, 85.98, 84.39, 79.61, 64.08, 61.65, 60.54, 42.54, 31.26, 29.53, 28.45, 26.86.
Physical State: light-yellow oil.
HRMS (ESI-TOF): calc'd for $C_{22}H_{29}NNaO_5S$ $[M+Na]^+$: 442.1664; found 442.1655.
TLC: R_f = 0.5 (50% EtOAc in Hexane).

Compound S15

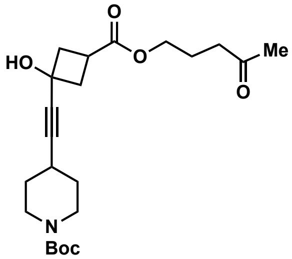

3-((1-(*tert*-butoxycarbonyl)piperidin-4-yl)ethynyl)-3-hydroxycyclobutane-1-carboxylic acid (160 mg, 0.69 mmol), 5-chloropentan-2-one (1.2 eq., 100 mg, 0.83 mmol), sodium iodide (1.0 eq., 103 mg, 0.69 mmol), and K_2CO_3 (1.5 eq., 143 mg, 1.05 mmol) were taken in a flame-dried round-bottom flask. 5 mL of dry and degassed DMF was added and stirred for 18 h at 50 ºC under a nitrogen atmosphere. Afterward, the reaction was quenched by adding 10 mL of distilled water, and the aqueous solution was extracted with diethyl ether (3 × 20 mL). The combined organic phases were washed with brine solution and dried over Na_2SO_4. After filtration, evaporation of the solvent gave a brown-colored residue, which was purified by column chromatography (Hexane/EtOAc,100:0 - 60:40) in 23% yield (65 mg, 0.16 mmol) as a light-yellow oil.

^{1}H-NMR (300 MHz, $CDCl_3$) δ (ppm): 4.10 (t, *J* = 6.4 Hz, 2H), 3.77 – 3.63 (m, 2H), 3.24 – 3.15 (m, 2H), 3.02 (s, 1H), 2.87 (dd, *J* = 17.1, 8.4 Hz, 1H), 2.68 – 2.45 (m, 7H), 2.16 (s, 3H), 1.94 – 1.87 (m, 2H), 1.80 – 1.74 (m, 2H), 1.62 – 1.51 (m, 2H), 1.45 (s, 9H).
^{13}C-NMR (101 MHz, $CDCl_3$) δ (ppm): 207.71, 174.75, 154.76, 86.20, 84.27, 79.58, 64.22, 64.01, 42.49, 41.23, 39.84, 31.27, 29.97, 29.52, 28.44, 26.89, 22.73.
Physical State: light-yellow oil.
HRMS (ESI-TOF): calc'd for $C_{22}H_{33}NNaO_6$ $[M+Na]^+$: 430.2206; found 430.2191.
TLC: R_f = 0.5 (40% EtOAc in Hexane).

Compound S16

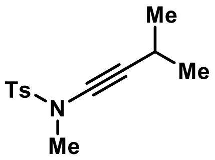

The preparation of the following starting material was carried out according to the literature procedure.[5]

^{1}H-NMR (300 MHz, $CDCl_3$) δ (ppm): 7.80 (d, *J* = 8.3 Hz, 1H), 7.37 (d, *J* = 8.0 Hz, 1H), 3.02 (s, 2H), 2.67 – 2.54 (m, 1H), 2.47 (s, 2H), 1.16 (s, 3H), 1.14 (s, 3H).

^{13}C-NMR (101 MHz, $CDCl_3$) δ (ppm): 144.45, 133.07, 129.56, 127.91, 74.51, 73.93, 39.42, 23.15, 21.64, 20.29.

GC\MS (EI): 91.0 (100%), 139.0 (48.9%), 236.0 (61.3%), 251.1 (88.2%).

Physical State: light yellow oil.

TLC: R_f = 0.5 (20% EtOAc in Hexane).

Compound S17

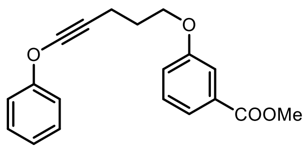

((5-bromopent-1-yn-1-yl)oxy)benzene (119 mg, 0.5 mmol), 3-hydroxymethylbenzoate (1.5 eq., 114 mg, 0.75 mmol), sodium iodide (1.0 eq., 75 mg, 0.5 mmol), and K_2CO_3 (1.5 eq., 104 mg, 0.75 mmol) were taken in a flame-dried round-bottom flask. To it, 5 mL dry and degassed DMF was added, and the reaction mixture was stirred for 6 h at 80 ºC under a nitrogen atmosphere. Afterward, the reaction was quenched by the addition of 10 mL of distilled water, and the aqueous solution was extracted with diethyl ether (3 × 20 mL). The combined organic phases were washed with brine solution and dried over Na_2SO_4. After filtration, evaporation of the solvent gave a dark brown colored residue, which was purified by column chromatography (Hex/EtOAc,100:0 - 90:10) in 82% yield (127 mg, 0.41 mmol) as a colorless oil.

^{1}H NMR (400 MHz, $CDCl_3$) δ (ppm): 7.66 (d, J = 7.7 Hz, 1H), 7.62 – 7.61 (m, 1H), 7.39 – 7.32 (m, 3H), 7.28 – 7.24 (m, 2H), 7.16 – 7.12 (m, 2H), 4.18 (t, J = 6.1 Hz, 2H), 3.93 (s, 3H), 2.54 (t, J = 6.9 Hz, 2H), 2.10 – 2.04 (m, 2H).
^{13}C NMR (101 MHz, $CDCl_3$) δ (ppm): 166.99, 158.98, 156.26, 131.46, 129.59, 129.42, 124.03, 122.01, 119.93, 114.82, 114.78, 83.82, 66.64, 52.17, 43.40, 28.94, 14.07.
Physical State: Colorless oil.
HRMS (ESI-TOF): calc'd for $C_{19}H_{18}NaO_4$ $[M+Na]^+$: 333.1103; found 333.1102.
TLC: R_f = 0.3 (10% Ethyl acetate in hexane)

<u>Compound 35</u>

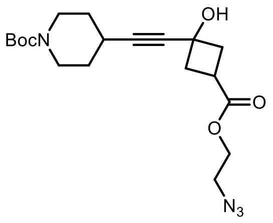

3-((1-(*tert*-butoxycarbonyl)piperidin-4-yl)ethynyl)-3-hydroxycyclobutane-1-carboxylic acid (277mg, 0.85 mmol), 1-azido-2-iodoethane (1.0 eq., 167.4 mg, 0.85 mmol), and K_2CO_3 (1.5 eq., 176 mg, 1.27 mmol) were taken in a flame-dried round-bottom flask. To it, 5.0 mL dry and degassed DMF was added, and the reaction mixture was stirred for 18 h at 80 ºC under a nitrogen atmosphere. Afterward, the reaction was quenched by the addition of 10 mL of distilled water, and the aqueous solution was extracted with diethyl ether (3 × 20 mL). The combined organic phases were washed with brine solution and dried over Na_2SO_4. After filtration, evaporation of the solvent gave a brown-colored residue, which was purified by column chromatography (Hex/EtOAc,100:0 - 60:40) in 61% yield (202 mg, 0.51 mmol) as a light-yellow oil.

^{1}H NMR (400 MHz, $CDCl_3$) δ (ppm): 4.26 – 4.23 (m, 2H), 3.67 – 3.63 (m, 2H), 3.47 – 3.45 (m, 2H), 3.19 – 3.14 (m, 2H), 2.97 – 2.88 (m, 2H), 2.68 – 2.56 (m, 3H), 2.50 (td, *J* = 9.4, 2.4 Hz, 2H), 1.77 – 1.72 (m, 2H), 1.58 – 1.49 (m, 2H), 1.43 (s, 9H).
^{13}C NMR (75 MHz, $CDCl_3$) δ (ppm): 174.37, 154.76, 86.42, 84.09, 79.59, 64.20, 63.42, 42.43, 42.18, 31.27, 29.36, 28.44, 26.93.
Physical State: Light yellow oil.
HRMS (ESI-TOF): calc'd for $C_{19}H_{28}N_4NaO_5$ $[M+Na]^+$: 415.1957; found 415.1951.
TLC: R_f = 0.5 (40% Ethyl acetate in hexane).

Compound S18

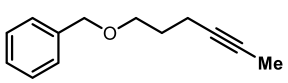

The preparation of the following starting material was carried out according to the literature procedure.[6]

^{1}H NMR (400 MHz, CDCl$_3$) δ (ppm): 7.40 – 7.29 (m, 5H), 4.57 (s, 2H), 3.62 (t, J = 6.2 Hz, 2H), 2.32 (tq, J = 7.1, 2.5 Hz, 2H), 1.88 – 1.81 (m, 5H).
^{13}C NMR (101 MHz, CDCl$_3$) δ (ppm): 138.66, 128.37, 127.62, 127.52, 78.60, 75.74, 72.94, 69.01, 29.25, 15.63, 3.48.
Physical State: Light yellow oil.
GC\MS (EI): 91.0 (100%), 173.0 (23.4%), 187.1 (25.8%), 188.1 (3.9%).
TLC: R_f = 0.7 (50:1 Hexane: Ethyl acetate).

Compound 49

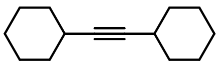

The preparation of the following starting material was carried out according to the literature procedure.[7]

^{1}H NMR (400 MHz, $CDCl_3$) δ (ppm): 2.34 (t, J = 8.2 Hz, 2H), 1.77 – 1.67 (m, 8H), 1.48 – 1.36 (m, 6H), 1.32 – 1.26 (m, 6H).
^{13}C NMR (101 MHz, $CDCl_3$) δ (ppm): 84.53, 33.25, 29.07, 26.03, 24.87.
Physical State: Colorless oil.
GC\MS (EI): 79.0 (100%), 122.0 (50.3%), 147.0 (28.2%), 190.1 (23.7%).
TLC: R_f = 0.2 (100% hexane).

Compound 37

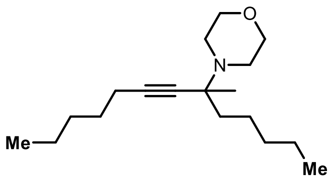

The preparation of the following starting material was carried out according to the literature procedure.[8]

^{1}H NMR (300 MHz, CDCl$_3$) δ (ppm): 3.62 (t, *J* = 4.7 Hz, 4H), 2.56 – 2.44 (m, 4H), 2.10 (t, *J* = 7.0 Hz, 2H), 1.49 – 1.16 (m, 17H), 0.81 (td, *J* = 7.1, 2.4 Hz, 6H).

^{13}C NMR (101 MHz, CDCl$_3$) δ (ppm): 84.46, 81.54, 67.46, 57.45, 47.02, 39.31, 32.25, 31.05, 28.81, 23.76, 23.64, 22.60, 22.14, 18.55, 14.05, 13.99.

Physical State: Yellow oil.

HRMS (ESI-TOF): calc'd for $C_{18}H_{34}NO$ $[M+H]^+$: 280.2640; found 280.2640.

TLC: R_f = 0.7 (10:90 Ethyl acetate: Hexane).

Compound 43

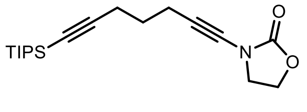

In an oven-dried round-bottom flask containing a stir bar, 2-oxazolidinone (295 mg, 3.39 mmol, 2.0 equiv.), K_3PO_4 (719 mg, 3.39 mmol, 2.0 equiv.), $CuSO_4$. $5H_2O$ (27 mg, 0.17 mmol, 0.1 equiv.) and 1,10-phenanthroline (61 mg, 0.338 mmol, 0.2 equiv.) were added. To this mixture, a solution of (7-bromohepta-1,6-diyn-1-yl)tri-*iso*-propylsilane (555 mg, 1.69 mmol, 1.0 equiv.) in DMF/toluene (1/10) was added dropwise under N_2 atmosphere. The reaction mixture was heated to 100 °C for 24 h while being monitored with TLC analysis. Upon completion, the reaction mixture was cooled to room temperature, diluted with EtOAc, filtered through Celite, and the filtrate was concentrated under vacuum. The crude products were purified by column chromatography (Hex/EtOAc,100:0 - 60:40) in 67% yield (381 mg, 1.13 mmol) as a colorless oil.

^{1}H NMR (800 MHz, $CDCl_3$) δ (ppm): 4.36 – 4.34 (m, 2H), 3.82 – 3.80 (m, 2H), 2.38 (t, J = 7.1 Hz, 2H), 2.29 (t, J = 7.0 Hz, 2H), 1.68 (p, J = 7.0 Hz, 2H), 0.99 – 0.93 (m, 21H).
^{13}C NMR (201 MHz, $CDCl_3$) δ (ppm): 159.28, 110.37, 83.54, 73.22, 72.64, 65.59, 49.64, 30.66, 21.64, 21.22, 20.09, 13.84.
Physical State: Colorless oil.
HRMS (ESI-TOF): calc'd for $C_{19}H_{31}NaNO_2Si$ $[M+Na]^+$: 356.2022; found 356.2019.
TLC: R_f = 0.4 (60:40 Ethyl acetate: hexane).

<u>Compound S19</u>

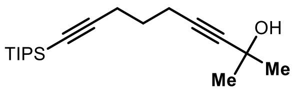

EtMgBr in THF (1.0 M, 2.5 mmol) was added to a solution of hepta-1,6-diyn-1-yltri-*iso*-propylsilane (162 mg, 0.65 mmol) in dry and degassed THF (5 mL) at 0 ºC under an inert atmosphere. The mixture was stirred for 1 h at 50 ºC and then cooled again at 0 ºC. At this temperature, dry and degassed acetone (167 µL, 2.25 mmol) was added rapidly. The reaction mixture was further stirred for 2 hours at that temperature and quenched with water. The organic layer was separated, and the aqueous layer was extracted with Et_2O (2 × 20 mL). The combined organic layers were dried over Na_2SO_4 and purified by column chromatography (Hex/EtOAc, 100:0 - 80:20) in a 21% yield (42 mg, 0.13 mmol) as a colorless oil.

^{1}H NMR (400 MHz, $CDCl_3$) δ (ppm): 2.34 (dt, *J* = 10.8, 7.0 Hz, 4H), 1.89 (s, 1H), 1.71 (p, *J* = 7.0 Hz, 2H), 1.49 (s, 6H), 1.08 – 0.99 (m, 21H).
^{13}C NMR (101 MHz, $CDCl_3$) δ (ppm): 107.84, 85.67, 81.48, 80.93, 65.29, 31.73, 27.89, 19.01, 18.62, 17.63, 11.27.
Physical State: colorless oil.
HRMS (ESI-TOF): calc'd for $C_{19}H_{34}NaOSi$ $[M+Na]^+$: 329.2277; found 329.2277.
TLC: R_f = 0.5 (20% Ethyl acetate in hexane).

Compound S20

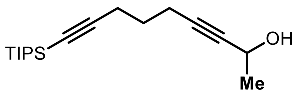

EtMgBr in THF (1.0 M, 2.0 mmol) was added to a solution of hepta-1,6-diyn-1-yltri-*iso*-propylsilane (150 mg, 0.60 mmol) in dry and degassed THF (5 mL) at 0 ºC under an inert atmosphere. The mixture was stirred for 1h at 50 ºC and then cooled again at 0 ºC. At this temperature, dry and degassed acetaldehyde (72 µL, 2.1 mmol) was added rapidly. The reaction mixture was further stirred for 2 hours at that temperature and quenched with water. The organic layer was separated, and the aqueous layer was extracted with Et_2O (2 × 20 mL). The combined organic layers were dried over Na_2SO_4 and purified by column chromatography (Hex/EtOAc,100:0 - 80:20) in 50% yield (87 mg, 0.3 mmol) as a colorless oil.

^{1}H NMR (300 MHz, $CDCl_3$) δ (ppm): 4.52 – 4.49 (m, 1H), 2.40 – 2.33 (m, 4H), 1.83 (s, 1H), 1.74 (p, J = 7.0 Hz, 2H), 1.44 (d, J = 6.5 Hz, 3H), 1.10 – 1.05 (m, 21H).
^{13}C NMR (75 MHz, $CDCl_3$) δ (ppm): 107.79, 83.58, 82.78, 80.99, 58.57, 27.89, 24.71, 19.04, 18.62, 17.70, 11.27.
Physical State: colorless oil.
HRMS (ESI-TOF): calc'd for $C_{18}H_{33}OSi$ $[M+H]^+$: 293.2301; found 293.2302.
TLC: R_f = 0.5 (20% Ethyl Acetate in Hexane).

Compound S21

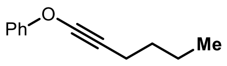

The preparation of the following starting material was carried out according to the literature procedure.[9]

^{1}H NMR (300 MHz, $CDCl_3$) δ (ppm): 7.41 – 7.11 (m, 5H), 2.32 (t, *J* = 6.9 Hz, 2H), 1.58 – 1.51 (m, 4H), 0.98 (t, *J* = 7.2 Hz, 3H).
^{13}C NMR (75 MHz, $CDCl_3$) δ (ppm): 156.46, 129.54, 123.88, 114.82, 83.29, 44.67, 31.50, 21.98, 16.96, 13.65.
Physical State: Colorless oil.
GC\MS (EI): 77.0 (100%), 103.0 (32%), 131.0 (17.4%), 159.0 (8.2%), 174.0 (22.2%).
TLC: R_f = 0.4 (100% Hexane).

Compound S22

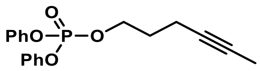

A flame-dried 20 mL glass vial was charged with hept-5-yn-1-ol (190 mg, 2.0 mmol), Et_3N (0.418 mL, 3.0 mmol), DMAP (61 mg, 0.5 mmol), and dichloromethane (10 mL). The reaction mixture was cooled to 0 °C, and diphenyl chlorophosphate (0.621 mL, 3.0 mmol) was added dropwise to the solution. The resulting suspension was allowed to warm to room temperature. After completion (TLC monitoring), the reaction was carefully quenched with saturated aqueous NH_4Cl solution, and the aqueous phase was extracted with dichloromethane (3 × 50 mL). The combined organic phases were washed with water and dried over anhydrous Na_2SO_4, filtered, and the volatiles were evaporated under reduced pressure. The crude was purified by column chromatography (Hexane: ethyl acetate 100:0 to 1:1) to yield 90% (594 mg, 1.8 mmol) of the desired compound as a light brown liquid.

^{1}H NMR (300 MHz, $CDCl_3$) δ (ppm): 7.35 (t, *J* = 7.8 Hz, 4H), 7.26 – 7.17 (m, 6H), 4.38 (dt, *J* = 12.5, 6.2 Hz, 2H), 2.27 – 2.22 (m, 2H), 1.89 – 1.85 (m, 2H), 1.76 (t, *J* = 2.5 Hz, 3H).
^{13}C NMR (75 MHz, $CDCl_3$) δ (ppm): 150.59 (d, *J* = 7.1 Hz), 129.78, 125.33 (d, *J* = 1.1 Hz), 120.05 (d, *J* = 4.9 Hz), 67.95 (d, *J* = 6.4 Hz), 31.94, 29.69 (d, *J* = 3.3 Hz), 29.45 (t, *J* = 6.0 Hz), 22.70, 14.90, 3.40.
^{31}P NMR (121 MHz, $CDCl_3$) δ (ppm): -10.57 (s).
Physical State: light brown oil.
HRMS (ESI-TOF): calc'd for $C_{18}H_{19}NaO_4P$ $[M+Na]^+$: 353.0919; found 353.0919.
TLC: R_f = 0.3 (1:1 EtOAc and Hexane).

Compound S23

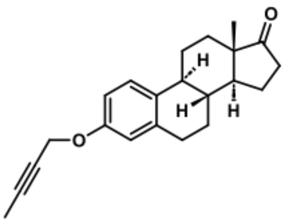

A flame-dried 10 mL glass vial was charged with estrone (270 mg, 1.0 mmol) and dry DMF (2.0 mL). To it, Cs2CO3 (450 mg, 1.3 mmol) was added, followed by the addition of 1-bromobut-2-yne (0.114 mL, 1.3 mmol). The reaction mixture was stirred at room temperature for 12 h. After completion (TLC monitoring), the reaction was carefully quenched with brine solution, and the aqueous phase was extracted with diethyl ether (3 × 50 mL). The combined organic phases were washed with water and dried over anhydrous Na_2SO_4, filtered, and the volatiles were evaporated under reduced pressure. The crude was purified by column chromatography (Diethyl ether: hexane 1:9) to yield 98% (315 mg, 1.8 mmol) of the desired compound as a white solid.

^{1}H NMR (300 MHz, $CDCl_3$) δ (ppm): 7.22 (d, *J* = 8.6 Hz, 1H), 6.79 (dd, *J* = 8.6, 2.8 Hz, 1H), 6.71 (d, *J* = 2.7 Hz, 1H), 4.63 (q, *J* = 2.3 Hz, 2H), 2.92 (dd, *J* = 9.4, 4.7 Hz, 2H), 2.52 (dd, *J* = 18.3, 8.4 Hz, 1H), 2.45 – 2.40 (m, 1H), 2.30 – 2.22 (m, 1H), 2.19 – 1.99 (m, 4H), 1.89 – 1.87 (m, 3H), 1.68 – 1.41 (m, 6H), 0.93 (s, 3H).
^{13}C NMR (75 MHz, $CDCl_3$) δ (ppm): 220.85, 155.88, 137.77, 132.61, 126.31, 114.87, 112.30, 83.51, 74.28, 56.37 (d, *J* = 6.9 Hz), 50.43, 48.00, 44.01, 38.34, 35.88, 31.61, 29.68, 26.56, 25.91, 21.61, 13.87, 3.76.
Physical State: White solid.
HRMS (ESI-TOF): calc'd for $C_{22}H_{26}NaO_2$ $[M+Na]^+$: 345.1830; found 345.1828.
TLC: R_f = 0.3 (10% diethyl ether in hexane).

Compound S24

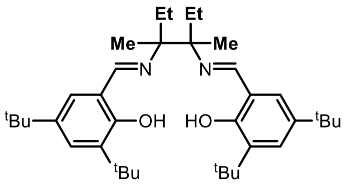

In an oven-dried 20-mL vial equipped with a magnetic stirring bar, 3,5-di-*tert*-butylsalicylic acid (0.468 g, 2.0 mmol), 3,4-dimethylhexane-3,4-diamine dihydrochloride[10] (0.217 g, 1.0 mmol), triethylamine (0.348 mL, 2.5 mmol), and ethanol (5.0 mL) were added. The vial was sealed with a cap and placed in a pre-heated oil bath at 70 ºC, stirred for 6 h under N_2 atmosphere. The reaction mixture was cooled to ambient temperature, concentrated under vacuum, and diluted with 2 mL of water. The mixture was filtered, washed with cold ethanol, and dried under vacuum to afford the ligand as a yellow solid (0.520 g, 91% yield).

^{1}H NMR (300 MHz, $CDCl_3$) δ (ppm): 8.29 (s, 1H), 8.19 (s, 1H), 7.38 (dd, *J* = 10.2, 2.4 Hz, 2H), 7.14 (d, *J* = 2.4 Hz, 1H), 7.03 (d, *J* = 2.3 Hz, 1H), 2.04 (dq, *J* = 14.4, 7.2 Hz, 2H), 1.90 – 1.81 (m, 1H), 1.67 (dd, *J* = 13.6, 7.3 Hz, 1H), 1.47 (d, *J* = 4.9 Hz, 18H), 1.34 (s, 10H), 1.29 (s, 26H), 0.77 (dt, *J* = 14.5, 7.4 Hz, 6H).
^{13}C NMR (201 MHz, $CDCl_3$) δ (ppm): 166.07, 161.44, 142.23, 139.33, 129.43, 128.95, 120.44, 70.99, 37.71, 36.79, 34.18, 32.13, 18.78, 11.01.
Physical State: Yellow solid.
HRMS (ESI-TOF): calc'd for $C_{38}H_{61}N_2O_2$ $[M+H]^+$: 577.4733; found 577.4742.

Catalyst Co-5

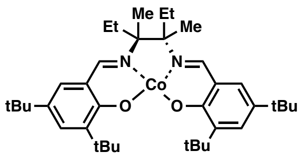

Ligand **S24** (288 mg, 0.5 mmol, 1.0 equiv.) and anhydrous $Co(OAc)_2$ (88 mg, 0.5 mmol, 1.0 equiv.) were dissolved in EtOH (10.0 mL) and heated to reflux in an oil bath. The color of the reaction mixture immediately changed from yellow to red, indicating the formation of the complex. After 4 h, the reaction mixture was cooled to room temperature, filtered through a filter paper, washed with ethanol, and dried under vacuum to obtain the dark red Co-5 complex (200.2 mg, 63% yield).[11]

General procedure A: cobalt electrocatalyzed *trans*-selective hydrogenation

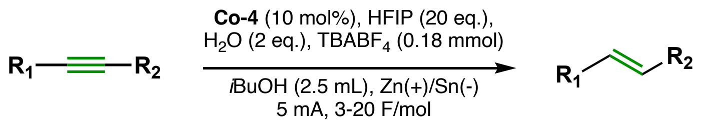

In an oven-dried 5.0 mL Electrasyn-vial containing a magnetic stirring bar and wrapped with a single layer of Teflon tape on the screw thread, **Co-4** (6.05 mg, 0.01 mmol), TBABF4 (60.0 mg, 0.18 mmol), and the alkyne substrate (0.1 mmol) were charged. Next, H_2O (3.6 µL, 0.2 mmol), HFIP (210 µL, 2.0 mmol), and 2.5 mL of dry and degassed *i*so-BuOH were added. The vial was then sealed with the cap bearing an IKA zinc anode and an IKA tin cathode. The reaction mixture was bubbled with nitrogen gas for 5 minutes. Finally, the Electrasyn-vial was fitted in an Electrasyn and electrolyzed under a constant current of 5.0 mA for 3-24 F/mol. The reaction was monitored by TLC, GC-MS, or NMR to confirm near-full consumption of alkyne starting material. After reaction completion, the crude mixture was diluted with diethyl ether (25 mL) and then washed with water (25 mL). After phase separation, the collected aqueous phase was extracted with diethyl ether (2 × 25 mL). The combined organic phase was washed with water (20 mL) and then brine (20 mL). The organic phase was dried with Na_2SO_4 and concentrated under vacuum. The product was obtained using chromatography or P-TLC.

General procedure B: General procedures for deuterium labeling hydrogenation

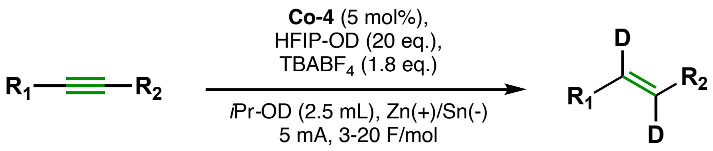

In an oven-dried 5.0 mL Electrasyn-vial containing a magnetic stirring bar and wrapped with a single layer of Teflon tape on the screw thread was charged with Co-4 (3.0 mg, 0.005 mmol), $TBABF_4$ (60.0 mg, 0.18 mmol), and alkyne substrate (0.1 mmol). HFIP-OD (210 μL) and 2.5 mL of degassed *iso*-propanol-OD were added. The vial was then sealed with the cap bearing an IKA zinc anode and an IKA tin cathode. The reaction mixture was then bubbled with N_2 gas for 5 minutes. Finally, the Electrasyn vial was fitted in an Electrasyn and electrolyzed under a constant current of 5.0 mA. After reaction completion, the crude mixture was diluted with diethyl ether (25 mL) and washed with water (25 mL). After phase separation, the collected aqueous phase was extracted with diethyl ether (2 × 25 mL). The combined organic phase was washed with water (20 mL) and then brine (20 mL). The organic phase was dried with Na_2SO_4 and then concentrated under vacuum. The product was obtained using chromatography or P-TLC.

Graphical Guide

Photos were taken from the reduction of compound **S46**.

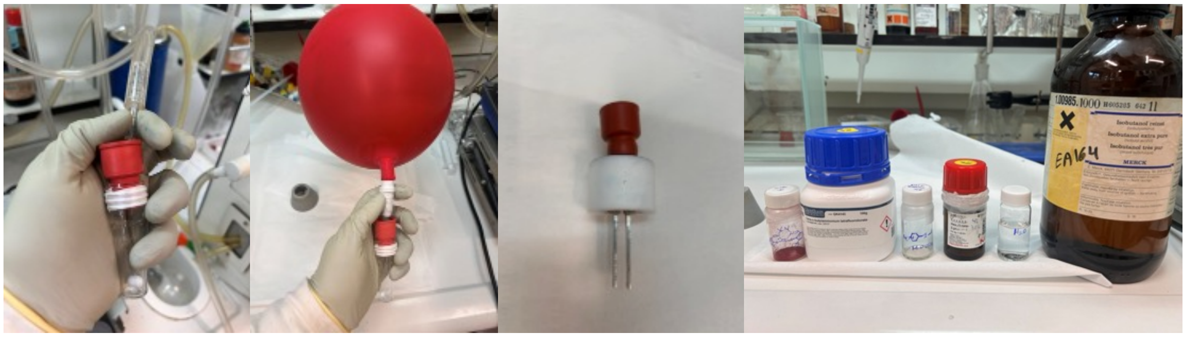

Picture 1. ElectraSyn 2.0 vial (5.0 mL, wrapped with Teflon tape) with a stir bar; electrodes (Zn as anode and Sn as cathode) and all reagents for this reaction.

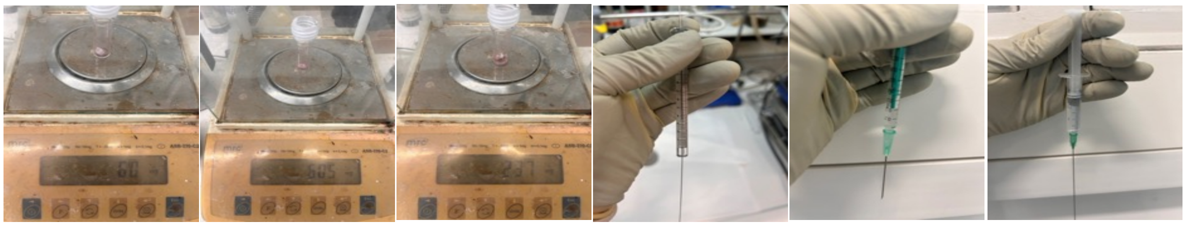

Picture 2. Weighing and measuring catalysts and reagents (From left to right: Catalyst, Electrolyte, Water, HFIP, and *iso*-BuOH).

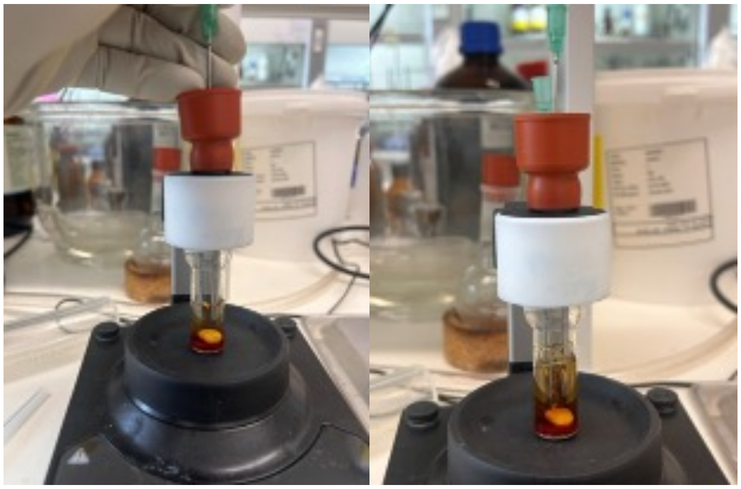

Picture 3. Purging of the reaction mixture with nitrogen.

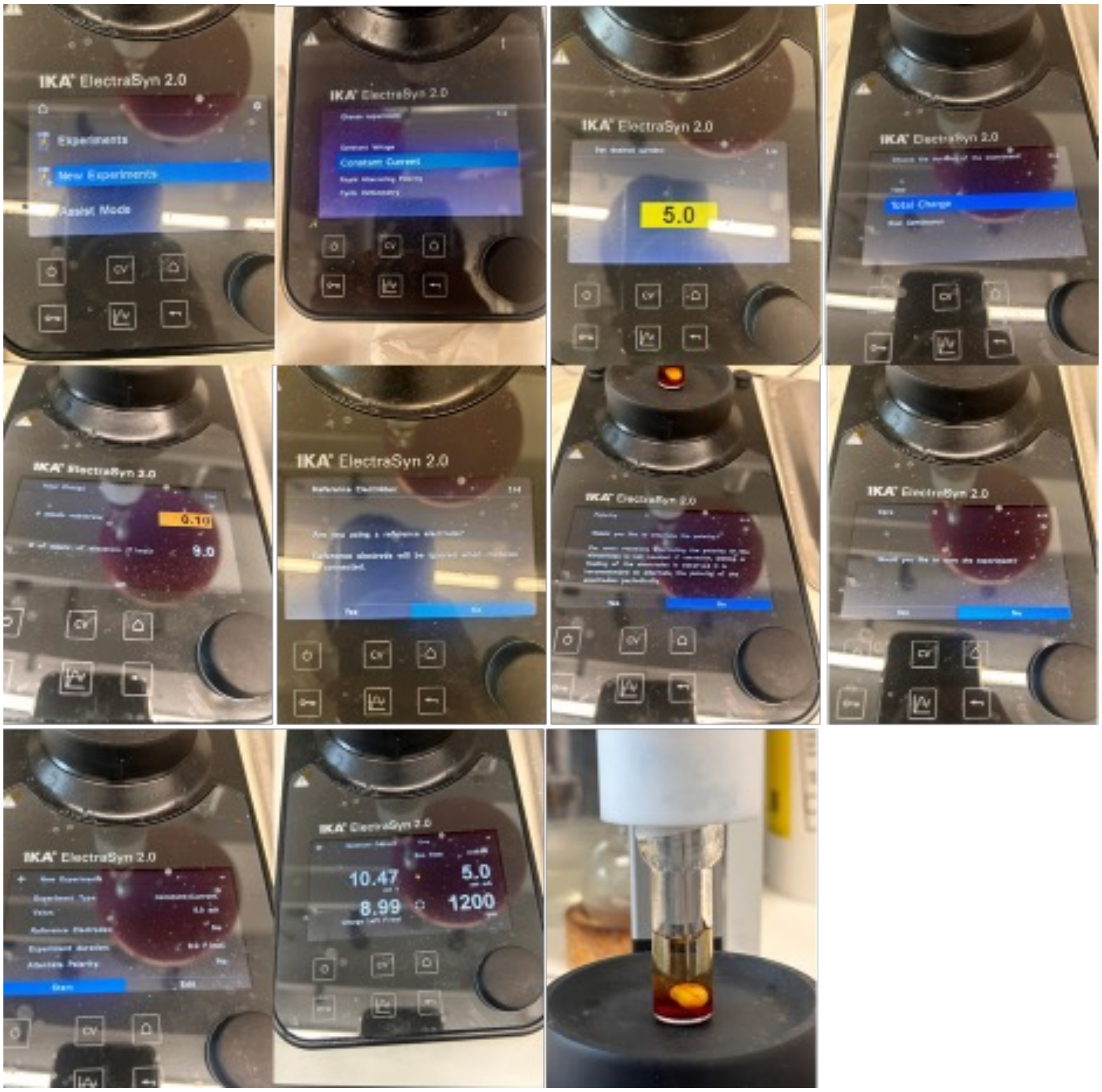

Picture 4. Electrasyn setup.

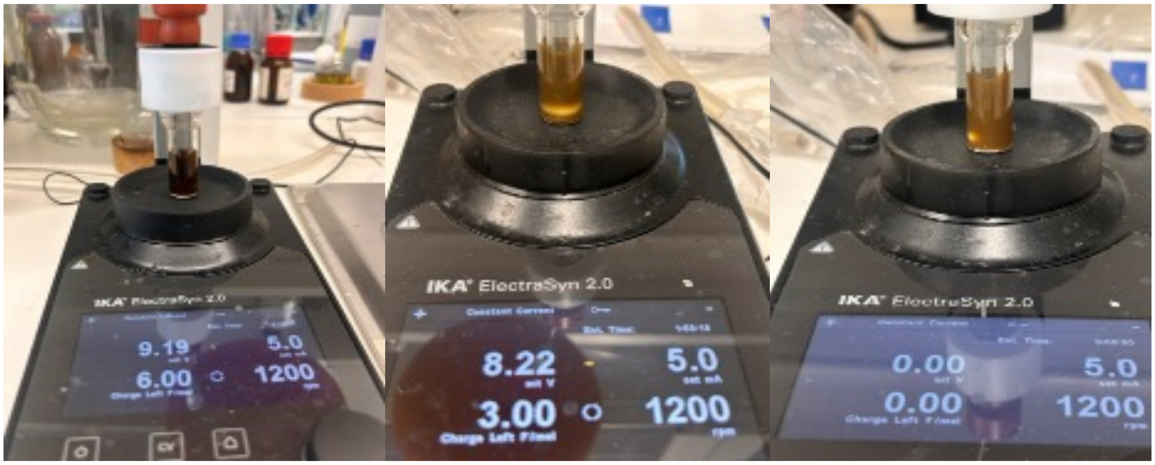

Picture 5. Color changes with the progress of the reaction (from left to right after 3F, 6F, and 9F, respectively).

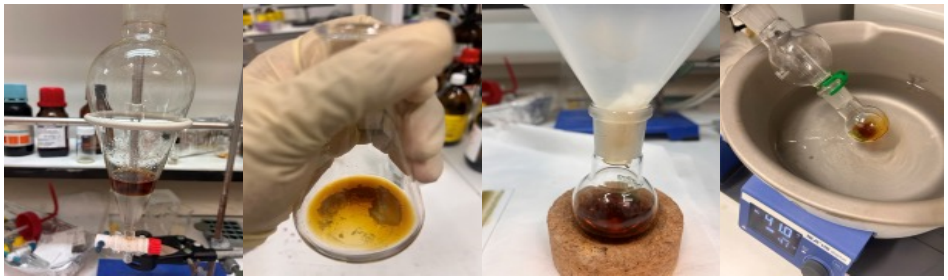

Picture 6. Workup process after completion of the reaction (from left to right: Extractions of the reaction mixture with diethyl ether and water, followed by washing with brine solution; dried over sodium sulfate, concentrated the crude using rotavap.).

General procedure C: Chemical conditions for alkyne *trans*-hydrogenation

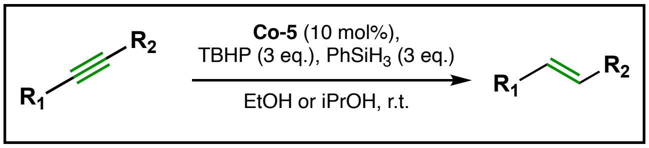

In an oven-dried 10.0 mL vial, a magnetic stirring bar was charged with Co (Salen)-catalyst **Co-5** (6.0 mg, 10.0 µmol) and alkyne substrate (0.1 mmol). To it, TBHP (3-4.5 equiv., 0.3-0.45 mmol, 33-50 µl), $PhSiH_3$ (3-4.5 equiv., 0.3-0.45 mmol, 37-55 µl), and 2.5 mL of dry and degassed alcoholic solvent (Ethanol or *iso*-PrOH) were added. The vial was then sealed with the septum and allowed to stir at room temperature for 12 h. After completion of the reaction, the mixture was quenched with 5 mL of water, followed by the extraction of ethyl acetate (3 × 20 mL); finally, the combined organic layers were washed with brine solution and concentrated under vacuum. The product was obtained using chromatography or P-TLC.

Compound 2

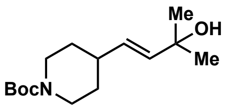

Following the general procedure A on 0.1 mmol scale: Co(salen)-**4** (6.0 mg, 10 μmol), $TBABF_4$ (60 mg), HFIP (210 μL, 2.0 mmol), H_2O (3.6 μL, 0.2 mmol) and *iso*-BuOH (2.5 mL), with zinc as anode and tin as cathode under the electrolysis of 5 mA for 18 F/mol. Compound **2** was purified by P-TLC (silica, 7:3 Hexane:EtOAc) to afford 23.6 mg (88%) as a pale yellow oil. E:Z = 94:6 (0% Alkane).

Following the general procedure C on a 0.1 mmol scale, using Co(salen)-**5** (6.0 mg, 10 μmol), TBHP (33.4 μl), $PhSiH_3$ (37 μl), and 2.5 mL of dry and degassed *iso*-PrOH, the mixture was stirred at room temperature for 12 h. Compound **2** was purified by P-TLC (silica, 7:3 Hexane:EtOAc) to afford 25 mg (93%) as a pale yellow oil. E:Z = 97:3 (0% Alkane).

^{1}H-NMR (300 MHz, $CDCl_3$) δ (ppm): 5.64 – 5.51 (m, 2H), 4.11– 4.06 (m, 2 H), 2.72 (t, J = 12.0, 2H), 2.13 – 2.04 (m, 1H), 1.68 – 1.60 (m, 4H), 1.45 (s, 9H), 1.30 (s, 6H), 1.29 – 1.23 (m, 2H).
^{13}C-NMR (125 MHz, $CDCl_3$) δ (ppm): 154.87, 136.65, 130.90, 79.32, 70.63, 43.7, 38.44, 31.85, 29.89, 28.48.
Physical State: pale yellow oil.
HRMS (ESI-TOF): calc'd for $C_{15}H_{27}NNaO_3$ $[M+Na]^+$: 292.1884; found 292.1890.
TLC: R_f = 0.4 (7:3 Hexane: EtOAc).

Compound 3

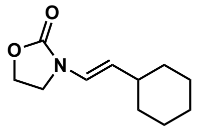

Following the general procedure A on a 0.1 mmol scale: Co(salen)-**4** (6.0 mg, 10 µmol), $TBABF_4$ (60 mg), HFIP (210 µL, 2.0 mmol), H_2O (3.6 µL, 0.2 mmol) and *iso*-BuOH (2.5 mL), with zinc as anode and tin as cathode under the electrolysis of 5 mA for 6F/mol. Compound **3** was purified by P-TLC (silica, 20% EtOAc in Hexane) to afford 19.0 mg (99%) as a colorless oil. E:Z = 99:1 (0% Alkane).

Following the general procedure C on a 0.1 mmol scale, using Co(salen)-**5** (6.0 mg, 10 µmol), TBHP (33.4 µl), $PhSiH_3$ (37 µl), and 2.5 mL of dry and degassed *iso*-PrOH, the mixture was stirred at room temperature for 12 h. Compound **3** was purified by P-TLC (silica, 8:2 Hexane:EtOAc) to afford 9.7 mg (50%) as a pale yellow oil. E:Z = 86:14 (8% alkene).

^{1}H-NMR (300 MHz, $CDCl_3$) δ (ppm): 6.62 (d, J = 14.5 Hz, 1H), 4.76 (dd, J = 14.4, 7.1 Hz, 1H), 4.48 – 4.34 (m, 2H), 3.67 (dd, J = 8.9, 7.2 Hz, 2H), 2.03 – 1.96 (m, 1H), 1.79 – 1.55 (m, 5H), 1.30 –1.22 (m, 3H), 1.20 – 1.06 (m, 2H).
^{13}C-NMR (101 MHz, $CDCl_3$) δ (ppm): 155.54, 122.29, 117.50, 62.09, 42.60, 38.50, 33.70, 26.04, 26.02.
Physical State: light yellow oil.
HRMS (ESI-TOF): calc'd for $C_{11}H_{17}NNaO_2$ $[M+Na]^+$: 218.1157; found 218.1161.
TLC: R_f = 0.5 (20% EtOAc in Hexane).

Compound 4

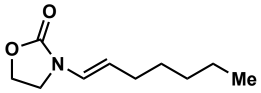

Following the general procedure A on a 0.1 mmol scale: Co(salen)-**4** (6.0 mg, 10 µmol), $TBABF_4$ (60 mg), HFIP (210 µL, 2.0 mmol), H_2O (3.6 µL, 0.2 mmol) and *iso*-BuOH (2.5 mL), with zinc as anode and tin as cathode under the electrolysis of 5 mA for 12F/mol. Compound **4** was purified by P-TLC (silica, 10% EtOAc in Hexane) to afford 14.0 mg (77%) as a colorless oil. E:Z = 99:1 (0% Alkane).

^{1}H-NMR (300 MHz, $CDCl_3$) δ (ppm): 6.67 (d, *J* = 14.3 Hz, 1H), 4.83 (dt, *J* = 14.3, 7.1 Hz, 1H), 4.43 (ddd, *J* = 12.4, 8.6, 5.6 Hz, 2H), 3.71 (dd, *J* = 8.9, 7.2 Hz, 2H), 2.17 – 2.00 (m, 2H), 1.47 – 1.17 (m, 6H), 0.91 (t, 3H).

^{13}C-NMR (101 MHz, $CDCl_3$) δ (ppm): 155.45, 123.81, 111.43, 62.08, 42.63, 31.24, 29.75, 22.49, 14.04.

Physical State: light yellow oil.

HRMS (ESI-TOF): calc'd for $C_{10}H_{17}NO_2Na$ [M+Na]+ 206.1157, found 206.1155.

TLC: R_f = 0.5 (10% EtOAc in Hexane).

Compound 5

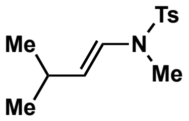

Following the general procedure A on a 0.1 mmol scale: Co(salen)-**4** (6.0 mg, 10 μmol), $TBABF_4$ (60 mg), HFIP (210 μL, 2.0 mmol), H_2O (3.6 μL, 0.2 mmol) and *iso*-BuOH (2.5 mL), with zinc as anode and tin as cathode under the electrolysis of 5 mA for 12F/mol. Compound **5** was purified by P-TLC (silica, 20% EtOAc and Hexane) to afford 24.5 mg (97%) as a colorless oil. E:Z = 85:15 (0% Alkane).

^{1}H-NMR (300 MHz, $CDCl_3$) δ (ppm): 7.66 – 7.62 (m, 1H), 7.32 (dd, *J* = 13.8, 5.8 Hz, 2H), 6.71 (dd, *J* = 14.2, 1.1 Hz, 1H), 4.70 (dd, *J* = 14.2, 7.2 Hz, 1H), 2.82 (s, 2H), 2.44 (s, 2H), 2.38 – 2.28 (m, 1H), 1.01 (s, 3H), 0.99 (s, 2H). (*E* and *Z* isomers are inseparable in P-TLC).
^{13}C-NMR (75 MHz, $CDCl_3$) δ (ppm): 143.53, 139.58, 134.51, 129.60, 127.73, 127.07, 125.87, 124.50, 119.38, 38.14, 32.24, 29.33, 26.26, 23.34, 22.54, 21.53.
Physical State: colorless oil.
HRMS (ESI-TOF): calc'd for $C_{13}H_{20}NO_2S$ $[M+H]^+$: 254.1215; found 254.1215.
TLC: R_f = 0.5 (20% EtOAc in Hexane).

Compound 6

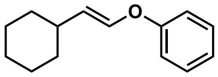

Following the general procedure A on a 0.1 mmol scale: Co(salen)-**4** (6.0 mg, 10 µmol), $TBABF_4$ (60 mg), HFIP (210 µL, 2.0 mmol), H_2O (3.6 µL, 0.2 mmol) and *iso*-BuOH (2.5 mL), with zinc as anode and tin as cathode under the electrolysis of 5 mA for 4F/mol. Compound **6** was purified by P-TLC (silica, 100% Hexane) to afford 12.3 mg (84%) as a colorless oil. E:Z = 95:5 (0% Alkane).

^{1}H-NMR (300 MHz, $CDCl_3$) δ (ppm): 7.36 – 7.23 (m, 2H), 7.08 – 6.93 (m, 3H), 6.41 (dd, *J* = 12.2, 1.0 Hz, 1H), 5.36 (dd, *J* = 12.2, 7.7 Hz, 1H), 2.09 – 1.97 (m, 1H), 1.78 – 1.64 (m, 4H), 1.38 – 1.32 (m, 1H), 1.29 – 1.24 (m, 3H), 1.21 – 1.08 (m, 3H).
^{13}C-NMR (101 MHz, $CDCl_3$) δ (ppm): 157.54, 140.23, 129.54, 122.33, 120.02, 116.28, 36.66, 33.73, 26.07, 26.04.
Physical State: colorless oil.
HRMS (ESI-TOF): calc'd for $C_{14}H_{19}O$ $[M+H]^+$: 203.1436; found 203.1436.
TLC: R_f = 0.4 (100% Hexane).

Compound 7

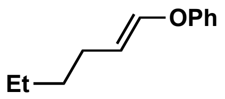

Following the general procedure A on a 0.1 mmol scale, using Co(salen)-**4** (6.0 mg, 10 μmol), $TBABF_4$ (60 mg), HFIP (210 μL, 2.0 mmol), H_2O (3.6 μL, 0.2 mmol) and *iso*-BuOH (2.5 mL), with zinc as anode and tin as cathode under the electrolysis of 5 mA for 4F/mol. Compound **7** was purified by P-TLC (Hexane) to afford 17 mg (97%) as a colorless oil. E:Z = 97:3 (0% Alkane).

Following the general procedure C on a 0.1 mmol scale, using Co(salen)-**5** (6.0 mg, 10 μmol), TBHP (33.4 μl), $PhSiH_3$ (37 μl), and 2.5 mL of dry and degassed *iso*-PrOH, the mixture was stirred at room temperature for 12 h. Compound **7** was purified by P-TLC (Hexane) to afford 17 mg (28%) as a colorless oil. E:Z = 86:14 (0% Alkane).

^{1}H NMR (300 MHz, $CDCl_3$) δ (ppm): 7.36 – 7.26 (m, 2H), 7.08 – 6.96 (m, 3H), 6.44 (dt, *J* = 12.1, 1.3 Hz, 1H), 5.40 (dt, *J* = 12.1, 7.5 Hz, 1H), 2.06 (qd, *J* = 7.2, 1.3 Hz, 2H), 1.41 – 1.40 (m, 4H), 0.97 – 0.92 (m, 3H).
^{13}C NMR (75 MHz, $CDCl_3$) δ (ppm): 157.46, 141.38, 129.55, 122.36, 116.30, 113.87, 32.14, 26.96, 22.11, 13.90.
Physical State: Colorless oil.
HRMS (ESI-TOF): calc'd for $C_{12}H_{15}O$ $[M-H]^-$: 175.1122; found 175.1123.
TLC: R_f = 0.4 (100% Hexane).

Compound 8

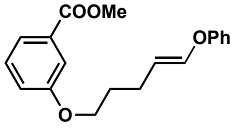

Following the general procedure A on a 0.1 mmol scale, using Co(salen)-**4** (6.0 mg, 10 μmol), $TBABF_4$ (60 mg), HFIP (210 μL, 2.0 mmol), H_2O (3.6 μL, 0.2 mmol) and *iso*-BuOH (2.5 mL), with zinc as anode and tin as cathode under the electrolysis of 5 mA for 3F/mol. Compound **8** was purified by P-TLC (silica, 1:9 Ethyl acetate: Hexane) to afford 26.3 mg (84%) as a colorless oil. E:Z = 92:8 (0% Alkane).

^{1}H NMR (400 MHz, $CDCl_3$) δ (ppm): 7.64 (d, *J* = 7.7 Hz, 1H), 7.58 – 7.57 (m, 1H), 7.36 – 7.24 (m, 2H), 7.12 – 7.10 (m, 1H), 7.03 (t, *J* = 7.4 Hz, 1H), 6.94 (d, *J* = 7.8 Hz, 1H), 6.47 (d, *J* = 12.1 Hz, 1H), 5.39 (dt, *J* = 12.1, 7.6 Hz, 1H), 4.06 (t, *J* = 6.2 Hz, 2H), 3.91 (s, 3H), 2.25 (q, *J* = 7.0 Hz, 2H), 1.95 – 1.86 (m, 2H).
^{13}C NMR (101 MHz, $CDCl_3$) δ (ppm): 167.00, 159.04, 157.26, 142.40, 131.46, 129.59, 129.42, 122.56, 121.96, 119.93, 116.37, 114.74, 112.10, 67.07, 52.18, 29.42, 23.78.
Physical State: colorless oil.
HRMS (ESI-TOF): calc'd for $C_{19}H_{20}NaO_4$ $[M+Na]^+$: 335.1259; found 335.1260.
TLC: R_f = 0.6 (1:9 Ethyl acetate: Hexane).

Compound 9

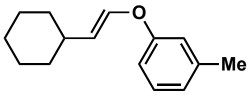

Following the general procedure A on a 0.1 mmol scale, using Co(salen)-**4** (6.0 mg, 10 µmol), $TBABF_4$ (60 mg), HFIP (210 µL, 2.0 mmol), H_2O (3.6 µL, 0.2 mmol) and *iso*-BuOH (2.5 mL), with zinc as anode and tin as cathode under the electrolysis of 5 mA for 3 F/mol. Compound **9** was purified by P-TLC (silica, 100% Hexane) to afford 19.0 mg (88%) as a colorless oil. E:Z = 98:2.

^{1}H-NMR (300 MHz, $CDCl_3$) δ (ppm): ^{1}H NMR (300 MHz, $CDCl_3$) δ 7.26 – 7.14 (m, 1H), 6.95 – 6.68 (m, 3H), 5.36 (dd, *J* = 12.2, 7.7 Hz, 1H), 2.36 (s, 3H), 2.04 (m, 1H), 1.88 – 1.63 (m, 5H), 1.45 – 1.04 (m, 6H).
^{13}C-NMR (101 MHz, $CDCl_3$) δ (ppm): 157.54, 140.3, 139.7, 129.3, 123.1, 119.7, 116.9, 113.3, 36.7, 33.7, 26.1, 26.1, 21.45.
Physical State: colorless oil.
HRMS (ESI-TOF): calc'd for $C_{15}H_{21}O$ $[M+H]^+$: 217.1592; found 217.1588.
TLC: R_f = 0.4 (100% Hexane).

Compound 10

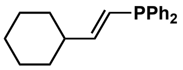

Following the general procedure A on a 0.1 mmol scale, using Co(salen)-**4** (6.0 mg, 10 µmol), $TBABF_4$ (60 mg), HFIP (210 µL, 2.0 mmol), H_2O (3.6 µL, 0.2 mmol) and *iso*-BuOH (2.5 mL), with zinc as anode and tin as cathode under the electrolysis of 5 mA for 9F/mol. Compound **10** was purified by P-TLC (90:10 Hexane/Ethyl acetate) to afford 26 mg (90%) as a pale yellow oil. E:Z = 89:11 (20% alkane).

^{1}H NM (300 MHz, $CDCl_3$) δ (ppm): 7.50 – 7.31 (m, 10H), 6.40 – 6.04 (m, 2H), 2.25 – 2.14 (m, 1H), 1.94 – 1.62 (m, 5H), 1.25 (m, 5H). (mixed with alkane product)

^{13}C NMR (75 MHz, $CDCl_3$) δ (ppm): 154.6, 132.8, 132.5, 128.5, 128.4, 124.2, 33.0, 32.3, 26.1, 25.9.

^{31}P NMR (121 MHz, $CDCl_3$) δ (ppm): -12.4.

Physical State: colorless oil.

HRMS (ESI-TOF): calc'd for $C_{20}H_{24}P$ $[M+H]^+$: 295.1616; found 295.1602.

TLC: R_f = 0.5 (Hexane).

Compound 11

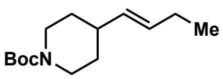

Following the general procedure A on a 0.1 mmol scale: Co(salen)-**4** (6.0 mg, 10 μmol), $TBABF_4$ (60 mg), HFIP (210 μL, 2.0 mmol), H_2O (3.6 μL, 0.2 mmol) and *iso*-BuOH (2.5 mL), with zinc as anode and tin as cathode under the electrolysis of 5 mA for 9 F/mol. Compound **11** was purified by P-TLC (silica, 8:1 Hexane: EtOAc) to afford 19 mg (79%) as a pale yellow oil. E:Z = 99:1 (and 12% Alkane).

Following the general procedure C on a 0.1 mmol scale, using Co(salen)-**5** (6.0 mg, 10 μmol), TBHP (33.4 μl), $PhSiH_3$ (37 μl), and 2.5 mL of dry and degassed *iso*-PrOH, the mixture was stirred at room temperature for 12 h. Compound **11** was purified by P-TLC (silica, 8:1 Hexane: EtOAc) to afford 16.9 mg (71%) as a pale yellow oil. E:Z = 67:33 (7% Alkane).

^{1}H-NMR (300 MHz, $CDCl_3$) δ (ppm): 5.52 – 5.45 (m, 1H), 5.39 – 5.31 (m, 1H), 4.10 – 4.06 (m, 2H), 2.74 (t, J = 12.8 Hz, 2H), 2.12 – 1.93 (m, 3H), 1.68 – 1.64 (m, 2H), 1.49 (s, 9H), 1.25 – 1.19 (m, 2H), 0.99 (t, J = 7.4 Hz, 3H).
^{13}C-NMR (101 MHz, $CDCl_3$) δ (ppm): 154.90, 133.20, 130.71, 79.22, 38.82, 32.08, 28.86, 28.49, 25.60, 13.90.
Physical State: pale yellow oil.
HRMS (ESI-TOF): calc'd for $C_{14}H_{25}NNaO_2$ $[M+Na]^+$: 262.1777; found 262.1794.
TLC: R_f = 0.5 (20:1 Hexane: EtOAc).

Compound 12

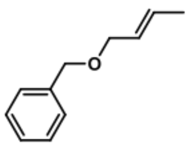

Following the general procedure A on 0.1 mmol scale: Co(salen)-**4** (6.0 mg, 10 μmol), $TBABF_4$ (60 mg), HFIP (210 μL, 2.0 mmol), H_2O (3.6 μL, 0.2 mmol) and *iso*-BuOH (2.5 mL), with zinc as anode and tin as cathode under the electrolysis of 5 mA for 12 F/mol. Compound **12** was purified by P-TLC (silica, 1:1 Hexane: Dichloromethane) to afford 13.6 mg (84%) as a pale yellow oil. E:Z = 99:1 (0% Alkane).[12]

^{1}H NMR (800 MHz, $CDCl_3$) δ (ppm): 7.36 – 7.31 (m, 4H), 7.28 (tt, *J* = 4.9, 3.4 Hz, 1H), 5.75 – 5.71 (m, 1H), 5.65 – 5.61 (m, 1H), 4.50 (s, 2H), 3.96 – 3.95 (m, 2H), 1.72 (dd, *J* = 6.5, 1.4 Hz, 3H).
^{13}C NMR (201 MHz, $CDCl_3$) δ (ppm): 141.11, 132.41, 131.02, 130.46, 130.20, 130.18, 74.59, 73.58, 20.48.
TLC: R_f = 0.4 (1:1 Hexane: Dichloromethane).

Compound 13

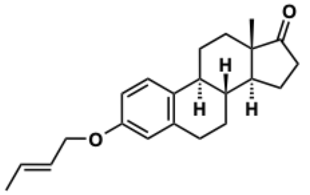

Following the general procedure A on 0.1 mmol scale: Co(salen)-**4** (6.0 mg, 10 μmol), $TBABF_4$ (60 mg), HFIP (210 μL, 2.0 mmol), H_2O (3.6 μL, 0.2 mmol) and *iso*-BuOH (2.5 mL), with zinc as anode and tin as cathode under the electrolysis of 5 mA for 12 F/mol. Compound **13** was purified by P-TLC (silica, 1:9 Diethyl ether: Hexane) to afford 26.2 mg (81%) as a colourless oil. E:Z = 99:1 (0% Alkane).

^{1}H NMR (300 MHz, $CDCl_3$) δ (ppm): 7.21 (d, *J* = 8.5 Hz, 1H), 6.75 (dd, *J* = 8.6, 2.8 Hz, 1H), 6.68 – 6.67 (m, 1H), 5.88 (ddd, *J* = 15.3, 12.4, 6.2 Hz, 1H), 5.82 – 5.69 (m, 1H), 4.51 – 4.40 (m, 2H), 2.99 – 2.85 (m, 2H), 2.53 (dd, *J* = 18.3, 8.3 Hz, 1H), 2.45 – 2.37 (m, 1H), 2.36 – 2.19 (m, 1H), 2.20 – 1.96 (m, 4H), 1.78 (dd, *J* = 6.2, 1.1 Hz, 3H), 1.67 – 1.40 (m, 6H), 0.93 (s, 3H).

^{13}C NMR (75 MHz, $CDCl_3$) δ (ppm): 220.97, 156.76, 137.71, 132.06, 130.38, 126.29, 114.75, 112.31, 68.67, 50.44, 48.03, 44.01, 38.39, 35.89, 31.61, 29.68, 26.58, 25.92, 21.60, 17.88, 13.87.

Physical State: colorless oil.

HRMS (ESI-TOF): calc'd for $C_{22}H_{29}O_2$ $[M+H]^+$: 325.2168; found 325.2159.

TLC: R_f = 0.3 (10% Diethyl ether in Hexane).

<u>Compound 14</u>

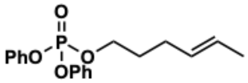

Following the general procedure A on 0.1 mmol scale: Co(salen)-**4** (6.0 mg, 10 µmol), $TBABF_4$ (60 mg), HFIP (210 µL, 2.0 mmol), H_2O (3.6 µL, 0.2 mmol) and *iso*-BuOH (2.5 mL), with zinc as anode and tin as cathode under the electrolysis of 5 mA for 12 F/mol. Compound **14** was purified by P-TLC (silica, 1:1 Ethyl acetate: Hexane) to afford 27.2 mg (82%) as a pale yellow oil. E:Z = 90:10 (0% Alkane).

^{1}H NMR (300 MHz, $CDCl_3$) δ (ppm): 7.37 (t, *J* = 7.8 Hz, 4H), 7.24 (tt, *J* = 13.3, 6.6 Hz, 6H), 5.56 – 5.32 (m, 2H), 4.30 – 4.24 (m, 2H), 2.07 (dd, *J* = 13.1, 6.5 Hz, 2H), 1.78 (p, *J* = 7.1 Hz, 2H), 1.65 (dd, *J* = 5.8, 0.9 Hz, 3H).

^{13}C NMR (75 MHz, $CDCl_3$) δ (ppm): 150.63 (d, *J* = 7.2 Hz), 129.77, 129.40, 126.31, 125.29 (d, *J* = 1.1 Hz), 120.07 (d, *J* = 4.9 Hz), 68.75 (d, *J* = 6.5 Hz), 29.95 (d, *J* = 6.9 Hz), 28.22, 17.89.

^{31}P NMR (121 MHz, $CDCl_3$) δ (ppm): -10.51 (s).

Physical State: light yellow oil.

HRMS (ESI-TOF): calc'd for $C_{18}H_{21}NaO_4$ $[M+Na]^+$: 355.1075; found 355.1078.

TLC: R_f = 0.3 (1:1 Ethyl acetate and Hexane).

Compound 15

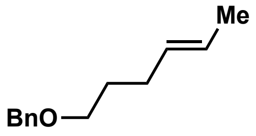

Following the general procedure A on a 0.1 mmol scale, using Co(salen)-**4** (12.0 mg, 20 μmol), $TBABF_4$ (60 mg), HFIP (210 μL, 2.0 mmol), H_2O (3.6 μL, 0.2 mmol) and *iso*-BuOH (2.5 mL), with zinc as anode and tin as cathode under the electrolysis of 5 mA for 17F/mol. Compound **15** was purified by P-TLC (40:1 Hexane/Ethyl acetate) to afford 15.3 mg (88%) as a pale yellow oil. E:Z = 91:9 (0% Alkane).

Following the general procedure C on a 0.1 mmol scale, using Co(salen)-**5** (6.0 mg, 10 μmol), TBHP (33.4 μl), $PhSiH_3$ (37 μl), and 2.5 mL of dry and degassed *iso*-PrOH, the mixture was stirred at room temperature for 12 h. Compound **15** was purified by P-TLC (40:1 Hexane/Ethyl acetate) to afford 13.3 mg (70%) as a pale yellow oil. E:Z = 60:40 (0% Alkane).

^{1}H NMR (400 MHz, $CDCl_3$) δ (ppm): 7.35 – 7.26 (m, 5H), 5.53 – 5.35 (m, 2H), 4.50 (s, 2H), 3.47 (t, *J* = 6.5 Hz, 2H), 2.09 – 2.05 (m, 2H), 1.67 – 1.63 (m, 5H).
^{13}C NMR (75 MHz, $CDCl_3$) δ (ppm): 130.74, 129.77, 127.48, 125.25, 72.87, 69.85, 29.61, 29.15, 17.92.
Physical State: Colorless oil.
HRMS (ESI-TOF): calc'd for $C_{13}H_{19}O$ $[M+H]^+$: 191.1436; found 191.1436.
TLC: R_f = 0.7 (40:1 Hexane: Ethyl acetate).

Compound 16

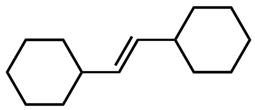

Following the general procedure A on a 0.1 mmol scale, using Co(salen)-**4** (6.0 mg, 10 µmol), $TBABF_4$ (60 mg), HFIP (210 µL, 2.0 mmol), H_2O (3.6 µL, 0.2 mmol) and *iso*-BuOH (2.5 mL), with zinc as anode and tin as cathode under the electrolysis of 5 mA for 6F/mol. Compound **16** was purified by P-TLC (100% Hexane) to afford 14.2 mg (74%) as a pale yellow oil. E:Z = 94:6 (12% Alkane).

^{1}H NMR (500 MHz, $CDCl_3$) (ppm): 5.40 – 5.28 (m, 2H), 1.96 – 1.86 (m, 2H), 1.80 – 1.62 (m, 10H), 1.31 – 1.12 (m, 10H).
^{13}C NMR (75 MHz, $CDCl_3$) δ (ppm): 133.78, 40.68, 33.36, 26.17, 24.88.
Physical State: Pale yellow oil.
HRMS (ESI-TOF): calc'd for $C_{14}H_{25}$ $[M+H]^+$: 193.1956; found 193.1957.
TLC: R_f = 0.2 (100% Hexane).

Compound 17

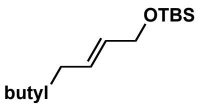

Following the general procedure A on a 0.1 mmol scale, using Co(salen)-**4** (6.0 mg, 10 μmol), $TBABF_4$ (60 mg), HFIP (210 μL, 2.0 mmol), H_2O (3.6 μL, 0.2 mmol) and *iso*-BuOH (2.5 mL), with zinc as anode and tin as cathode under the electrolysis of 5 mA for 12F/mol. Due to the highly volatile nature of Compound **17**, we failed to isolate the compound. GC-MS yield 97%, E:Z = 92:9 (20% Alkane).

^{1}H NMR (400 MHz, $CDCl_3$) δ (ppm): 5.77 – 5.46 (m, 2H), 4.13 – 4.11 (m, 2H), 1.43 – 1.28 (m, 8H), 0.91 (m, 12H), 0.07 (s, 6H).
^{13}C NMR (101 MHz, $CDCl_3$) δ (ppm):131.6, 129.1, 64.2, 32.2, 31.4, 29.4, 28.9, 26.0, 22.5, 14.1, -5.1.
Physical State: Colorless oil
HRMS (ESI-TOF): calc'd for $C_{14}H_{30}OSiNa$ $[M+Na]^+$: 265.1963; found 265.1966.
TLC: R_f = 0.3 (10% Ethyl acetate in hexane).

Compound 18

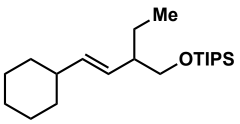

Following the general procedure A on a 0.1 mmol scale: Co(salen)-**4** (6.0 mg, 10 µmol), $TBABF_4$ (60 mg), HFIP (210 µL, 2.0 mmol), H_2O (3.6 µL, 0.2 mmol) and *iso*-BuOH (2.5 mL), with zinc as anode and tin as cathode under the electrolysis of 5 mA for 18 F/mol. Compound **18** was purified by P-TLC (100% Hexane) to afford 19.9 mg (59%) as a colorless oil. E:Z = 86:14 (0% Alkane).

^{1}H-NMR (300 MHz, $CDCl_3$) δ (ppm): 5.40 – 5.35 (m, 1H), 5.20 – 5.12 (m, 1H), 3.61 – 3.49 (m, 2H), 2.05 – 1.82 (m, 2H), 1.71 – 1.68 (m, 5H), 1.63 – 1.53 (m, 2H), 1.28 – 1.24 (m, 2H), 1.22 – 1.19 (m, 1H), 1.17 – 1.14 (m, 1H), 1.12 – 1.10 (m, 1H), 1.06 (s, 21 H), 0.89 – 0.82 (m, 3H).

^{13}C-NMR (75 MHz, $CDCl_3$) δ (ppm): 137.6, 128.9, 67.2, 47.4, 40.8, 33.2, 26.3, 26.1, 24.0, 18.1, 12.0, 11.6.

Physical State: colorless oil.

HRMS (ESI-TOF): calc'd for $C_{21}H_{43}OSi$ $[M+H]^+$: 339.3083; found 339.3033.

TLC: R_f = 0.5 (100% Hexane).

Compound 19

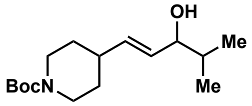

Following the general procedure A on a 0.1 mmol scale: Co(salen)-**4** (9.0 mg, 15 μmol), $TBABF_4$ (60 mg), HFIP (210 μL, 2.0 mmol), H_2O (3.6 μL, 0.2 mmol) and *iso*-BuOH (2.5 mL), with zinc as anode and tin as cathode under the electrolysis of 5 mA for 12 F/mol. Compound **19** was purified by P-TLC (silica, 7:3 Hexane: EtOAc) to afford 25.5 mg (90%) as a pale yellow oil. E:Z = 92:8 (0% Alkane).

^{1}H-NMR (300 MHz, $CDCl_3$) δ (ppm): 5.60 – 5.53 (m, 1H), 5.47 – 5.40 (m, 1H), 4.12 – 4.03 (m, 2H), 3.76 (t, J = 6.0 Hz, 1H), 2.71 (t, J = 12.0 Hz, 2H), 2.16 – 2.04 (m, 1H), 1.73 – 1.62 (m, 2H), 1.43 (s, 9H), 1.33 – 1.18 (m, 3H), 0.96 (t, J = 6.0 Hz, 1H), 0.90 – 0.83 (m, 6H).
^{13}C-NMR (75 MHz, $CDCl_3$) δ (ppm): 154.8, 136.4, 129.8, 79.3, 78.0, 38.6, 33.9, 31.8, 28.5, 18.2, 18.1.
Physical State: pale yellow oil.
HRMS (ESI-TOF): calc'd for $C_{16}H_{29}NNaO_3$ $[M+Na]^+$: 306.2045; found 306.2042.
TLC: R_f = 0.6 (7:3 Hexane: EtOAc).

Compound 20

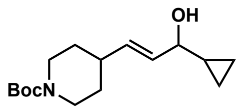

Following the general procedure A on a 0.1 mmol scale: Co(salen)-**4** (6.0 mg, 10 μmol), $TBABF_4$ (60 mg), HFIP (210 μL, 2.0 mmol), H_2O (3.6 μL, 0.2 mmol) and *iso*-BuOH (2.5 mL), with zinc as anode and tin as cathode under the electrolysis of 5 mA for 12 F/mol. Compound **20** was purified by P-TLC (silica, 3:7 EtOAc: Hexane) to afford 23.8 mg (84%) as a pale yellow oil. E:Z = 93:7 (0% Alkane).

^{1}H-NMR (300 MHz, $CDCl_3$) δ (ppm): 5.67 – 5.52 (m, 2H), 4.09 (d, *J* = 9.9 Hz, 2H), 3.44 (dd, *J* = 7.7, 5.6 Hz, 1H), 2.74 (t, *J* = 12.4 Hz, 2H), 2.14 – 2.11 (m, 1H), 1.71 – 1.68 (m, 3H), 1.47 (s, 9H), 1.36 – 1.24 (m, 2H), 1.00 – 0.96 (m, 1H), 0.57 – 0.47 (m, 2H), 0.37 – 0.32 (m, 1H), 0.27 – 0.22 (m, 1H).

^{13}C-NMR (126 MHz, $CDCl_3$) δ (ppm): 154.8, 135.3, 130.0, 79.3, 76.9, 43.7, 38.5, 31.7, 28.5, 17.6, 3.1, 2.0.

Physical State: pale yellow oil.

HRMS (ESI-TOF): calc'd for $C_{16}H_{27}NNaO_3$ $[M+Na]^+$: 304.1889; found 304.1897.

TLC: R$_f$ = 0.5 (3:7 EtOAc: Hexane).

Compound 21

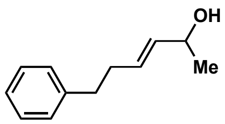

Following the general procedure A on a 0.1 mmol scale: Co(salen)-**4** (6.0 mg, 10 μmol), $TBABF_4$ (60 mg), HFIP (210 μL, 2.0 mmol), H_2O (3.6 μL, 0.2 mmol) and *iso*-BuOH (2.5 mL), with zinc as anode and tin as cathode under the electrolysis of 5 mA for 12 F/mol. Compound **21** was purified by P-TLC (silica, 3:7 EtOAc: Hexane) to afford 15.0 mg (85%) as a pale yellow oil. E:Z = 91:9 (0% Alkane).[13]

^{1}H-NMR (400 MHz, $CDCl_3$) δ (ppm): 7.33 – 7.27 (m, 2H), 7.18 (td, *J* = 7.0, 1.6 Hz, 3H), 5.68 (dtd, *J* = 14.1, 6.5, 0.9 Hz, 1H), 5.53 (ddt, *J* = 15.4, 6.6, 1.3 Hz, 1H), 4.30 – 4.22 (m, 1H), 2.73 – 2.66 (m, 2H), 2.39 – 2.29 (m, 2H), 1.24 (d, *J* = 6.3 Hz, 3H). (mixed with residue starting material).

^{13}C-NMR (126 MHz, $CDCl_3$) δ (ppm): 141.7, 134.8, 130.0, 128.5, 128.3, 125.9, 68.9, 35.6, 33.9, 23.4.

Physical State: pale yellow oil.

TLC: R_f = 0.5 (1:9 EtOAc: Hexane).

Compound 22

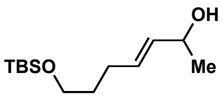

Following the general procedure A on a 0.1 mmol scale: Co(salen)-**4** (6.0 mg, 10 µmol), $TBABF_4$ (60 mg), HFIP (210 µL, 2.0 mmol), H_2O (3.6 µL, 0.2 mmol) and *iso*-BuOH (2.5 mL), with zinc as anode and tin as cathode under the electrolysis of 5 mA for 12 F/mol. Compound **22** was purified by P-TLC (silica, 3:7 EtOAc: Hexane) to afford 19.7 mg (81%) as a pale yellow oil. E:Z = 89:11 (0% Alkane).

^{1}H-NMR (400 MHz, $CDCl_3$) δ (ppm): 5.75 – 5.63 (m, 1H), 5.55 (ddt, *J* = 15.4, 6.6, 1.3 Hz, 1H), 4.35 – 4.25 (m, 1H), 3.63 (t, *J* = 6.4 Hz, 3H), 2.38 – 1.87 (m, 3H), 1.86 – 1.56 (m, 4H), 1.28 (d, *J* = 6.3 Hz, 5H), 0.92 (s, 16H), 0.07 (s, 9H).
^{13}C-NMR (201 MHz, $CDCl_3$) δ (ppm): 137.0, 133.3, 71.6, 65.1, 34.9, 31.0, 28.6, 28.6, 26.08, 21.0.
Physical State: pale yellow oil.
HRMS (ESI-TOF): calc'd for $C_{13}H_{28}O_2NaSi$ $[M+Na]^+$: 267.1756; found 267.1757.
TLC: R_f = 0.4 (2:8 EtOAc: Hexane).

Compound 23

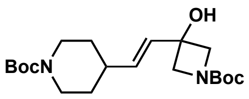

Following the general procedure A on a 0.1 mmol scale: Co(salen)-**4** (6.0 mg, 10 μmol), $TBABF_4$ (60 mg), HFIP (210 μL, 2.0 mmol), H_2O (3.6 μL, 0.2 mmol) and *iso*-BuOH (2.5 mL), with zinc as anode and tin as cathode under the electrolysis of 5 mA for 18 F/mol. Compound **23** was purified by P-TLC (silica, 3:7 EtOAc: Hexane) to afford 25.9 mg (68%) as a pale yellow oil. E:Z = 93:7 (0% Alkane).

^{1}H-NMR (400 MHz, $CDCl_3$) δ 5.76 – 5.3 (m, *J* = 1.5 Hz, 2H), 4.12 (br s, *J* = 15.9 Hz, 2H), 3.98 – 3.78 (m, 4H), 2.75 – 2.51 (m, *J* = 12.9 Hz, 3H), 2.23 – 2.12 (m, 1H), 1.77 – 1.62 (m, 2H), 1.47 (s, 9H), 1.46 (s, 9H), 1.38 – 1.27 (m, 2H).

^{13}C-NMR (126 MHz, $CDCl_3$) δ 156.5, 154.8, 134.1, 130.5, 79.8, 79.5, 70.6, 69.8, 62.6, 43.7, 38.4, 36.1, 31.6, 29.9, 28.5, 28.4.

Physical State: pale yellow oil.

HRMS (ESI-TOF): calc'd for $C_{20}H_{34}N_2O_5Na$ $[M+Na]^+$: 405.2365; found 405.2357.

TLC: R_f = 0.6 (3:7 EtOAc: Hexane).

Compound 24

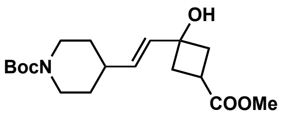

Following the general procedure A on a 0.1 mmol scale: Co(salen)-**4** (9.0 mg, 15 μmol), $TBABF_4$ (60 mg), HFIP (210 μL, 2.0 mmol), H_2O (3.6 μL, 0.2 mmol) and *iso*-BuOH (2.5 mL), with zinc as anode and tin as cathode under the electrolysis of 5 mA for 12 F/mol. Compound **24** was purified by P-TLC (silica, 3:7 EtOAc: Hexane) to afford 32.8 mg (97%) as a colorless oil. E:Z = 92:8 (0% Alkane).

^{1}H-NMR (400 MHz, $CDCl_3$) δ (ppm): 5.79 – 5.53 (m, 2H), 4.18 – 4.00 (m, 2H), 3.70 (s, 3H), 2.74 – 2.65 (m, 3H), 2.52 – 2.44 (m, 2H), 2.42 – 2.35 (m, 2H), 2.17 – 2.09 (m, 1H), 1.72 – 1.61 (m, 2H), 1.45 (s, 9H), 1.34 – 1.25 (m, 2H).
^{13}C-NMR (101 MHz, $CDCl_3$) δ (ppm): 176.0, 154.8, 132.8, 131.9, 79.4, 71.1, 52.0, 43.7, 39.9, 38.5, 29.0, 28.5.
Physical State: colorless oil.
HRMS (ESI-TOF): calc'd for $C_{18}H_{29}NNaO_5$ $[M+Na]^+$: 362.1943; found 362.1937.
TLC: R_f = 0.5 (3:7 EtOAc: Hexane).

Compound 25

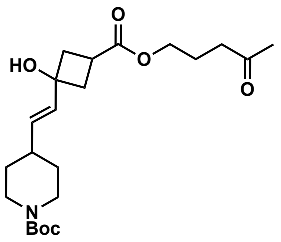

Following the general procedure A on a 0.1 mmol scale: Co(salen)-**4** (9.0 mg, 15 μmol), $TBABF_4$ (60 mg), HFIP (210 μL, 2.0 mmol), H_2O (3.6 μL, 0.2 mmol) and *iso*-BuOH (2.5 mL), with zinc as anode and tin as cathode under the electrolysis of 5 mA for 24F/mol. Compound **25** was purified by P-TLC (silica, 50% EtOAc and Hexane) to afford 34.0 mg (85%) as a colorless oil. E:Z = 90:10 (0% Alkane).

^{1}H-NMR (300 MHz, $CDCl_3$) δ (ppm): 5.79 – 5.63 (m, 2H), 4.12 (q, *J* = 5.2 Hz, 4H), 2.73 (ddd, *J* = 25.0, 13.2, 5.4 Hz, 3H), 2.53 (q, *J* = 5.9 Hz, 4H), 2.45 – 2.31 (m, 2H), 2.18 (m, 4H), 2.05 – 1.90 (m, 2H), 1.69 (d, *J* = 12.5 Hz, 2H), 1.48 (s, 9H), 1.37 – 1.23 (m, 2H).
^{13}C-NMR (126 MHz, $CDCl_3$) δ (ppm): 207.7, 175.5, 154.8, 132.8, 131.9, 79.4, 71.1, 64.0, 42.5, 39.9, 39.9, 38.5, 31.7, 29.9, 29.2, 28.5, 22.8.
Physical State: colorless oil.
HRMS (ESI-TOF): calc'd for $C_{22}H_{35}NNaO_6$ $[M+Na]^+$: 432.2362; found 432.2355.
TLC: R_f = 0.5 (50% EtOAc in Hexane).

Compound 26

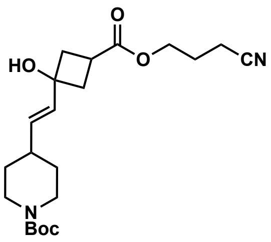

Following the general procedure A on a 0.1 mmol scale: Co(salen)-**4** (9.0 mg, 15 µmol), $TBABF_4$ (60 mg), HFIP (210 µL, 2.0 mmol), H_2O (3.6 µL, 0.2 mmol) and *iso*-BuOH (2.5 mL), with zinc as anode and tin as cathode under the electrolysis of 5 mA for 24F/mol. Compound **26** was purified by P-TLC (silica, 50% EtOAc in Hexane) to afford 31.4 mg (80%) as a colorless oil. E:Z = 90:10 (0% Alkane).

^{1}H-NMR (400 MHz, $CDCl_3$) δ (ppm): 5.82 – 5.59 (m, 2H), 4.25 (t, J = 5.9 Hz, 2H), 4.12 (s, 2H), 2.89 – 2.65 (m, 3H), 2.61 – 2.37 (m, 6H), 2.23 – 2.13 (m, 1H), 2.13 – 2.00 (m, 2H), 1.72 (m, 2H), 1.48 (s, 9H), 1.39 – 1.28 (m, 2H).
^{13}C-NMR (101 MHz, $CDCl_3$) δ (ppm): 175.1, 154.8, 132.9, 131.8, 79.4, 71.0, 62.7, 42.5, 40.0, 38.5, 31.7, 29.1, 28.5, 26.9, 24.8, 14.5.
Physical State: colorless oil.
HRMS (ESI-TOF): calc'd for $C_{21}H_{32}NNaO_5$ $[M+Na]^+$: 415.2209; found 415.2197.
TLC: R_f = 0.5 (50% EtOAc in Hexane).

Compound 27

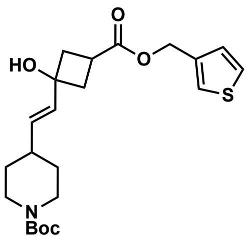

Following the general procedure A on a 0.1 mmol scale: Co(salen)-**4** (6.0 mg, 10 μmol), $TBABF_4$ (60 mg), HFIP (210 μL, 2.0 mmol), H_2O (3.6 μL, 0.2 mmol) and *iso*-BuOH (2.5 mL), with zinc as anode and tin as cathode under the electrolysis of 5 mA for 24F/mol. Compound **27** was purified by P-TLC (silica, 50% EtOAc and Hexane) to afford 33.0 mg (79%) as a colorless oil. E:Z = 90:10 (0% Alkane).

^{1}H-NMR (300 MHz, $CDCl_3$) δ (ppm): 7.38 – 7.31 (m, 2H), 7.10 (dd, *J* = 4.8, 1.5 Hz, 1H), 5.73 – 5.64 (m, 2H), 5.16 (s, 2H), 4.11 (d, *J* = 13.3 Hz, 2H), 2.75 (m, 3H), 2.58 – 2.47 (m, 2H), 2.46 – 2.35 (m, 2H), 2.16 (d, *J* = 3.3 Hz, 1H), 1.76 – 1.63 (m, 2H), 1.48 (s, 9H), 1.35 – 1.23 (m, 2H).

^{13}C-NMR (75 MHz, $CDCl_3$) δ (ppm): 175.3, 154.8, 136.6, 132.9, 131.8, 127.5, 126.3, 124.4, 79.4, 71.1, 61.7, 43.7, 39.9, 38.5, 31.7, 29.2, 28.5.

Physical State: colorless oil.

HRMS (ESI-TOF): calc'd for $C_{22}H_{31}NNaO_5S$ $[M+Na]^+$: 444.1821; found 444.1812.

TLC: R_f = 0.5 (50% EtOAc in Hexane).

Compound 28

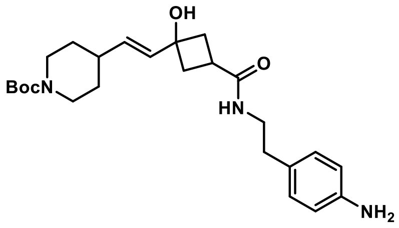

Following the general procedure A on a 0.1 mmol scale: Co(salen)-**4** (9.0 mg, 15 μmol), $TBABF_4$ (60 mg), HFIP (210 μL, 2.0 mmol), H_2O (3.6 μL, 0.2 mmol) and *iso*-butanol (2.5 mL), with zinc as anode and tin as cathode under the electrolysis of 5 mA for 12 F/mol. Compound **28** was purified by P-TLC (silica, 1:19 Methanol: Dichloromethane) to afford 40.2 mg (88%) as a pale yellow oil. E:Z = 93:7 (0% Alkane).

^{1}H-NMR (300 MHz, $CDCl_3$) δ (ppm): 7.03 – 6.92 (m, 2H), 6.65 (d, J = 8.3 Hz, 2H), 5.74 – 5.52 (m, 2H), 4.10 (s, 2H), 3.69 (m, 1H), 3.49 (dd, J = 6.2, 2.9 Hz, 2H), 3.18 (m, 1H), 2.81 – 2.58 (m, 4H), 2.50 – 2.40 (m, 2H), 2.35 – 2.22 (m, 1H), 1.86 – 1.58 (m, 2H), 1.47 (s, 9H), 1.32 (t, J = 8.3 Hz, 2H).

^{13}C-NMR (101 MHz, $CDCl_3$) δ (ppm): 176.0, 175.2, 154.9, 144.9, 132.6, 129.6, 128.4, 115.4, 86.2, 84.4, 79.4, 72.2, 65.4, 42.4, 40.9, 39.9, 38.4, 34.6, 32.9, 32.4, 28.5, 26.9.

Physical State: pale yellow oil.

HRMS (ESI-TOF): calc'd for $C_{25}H_{37}N_3NaO_4$ $[M+Na]^+$: 466.2682; found 466.2651.

TLC: R_f = 0.6 (1:19 Methanol: Dichloromethane).

<u>Compound 29</u>

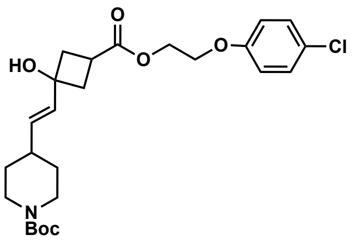

Following the general procedure A on a 0.1 mmol scale: Co(salen)-**4** (9.0 mg, 15 μmol), $TBABF_4$ (60 mg), HFIP (210 μL, 2.0 mmol), H_2O (3.6 μL, 0.2 mmol) and *iso*-BuOH (2.5 mL), with zinc as anode and tin as cathode under the electrolysis of 5 mA for 24F/mol. Compound **29** was purified by P-TLC (silica, 20% EtOAc in Hexane) to afford 30.0 mg (82%) as a colorless oil. E:Z = 95:5 (0% Alkane).

^{1}H-NMR (300 MHz, $CDCl_3$) δ (ppm): 7.32 – 7.20 (m, 2H), 6.92 – 6.80 (m, 2H), 5.75 – 5.64 (m, 2H), 4.46 (m, 2H), 4.21 – 3.92 (m, 6H), 2.75 (t, *J* = 7.9 Hz, 3H), 2.51 (t, *J* = 10.1 Hz, 2H), 2.46 – 2.36 (m, 2H), 2.19 – 2.07 (m, 1H), 1.71 (s, 2H), 1.47 (s, 9H), 1.34 (m, 2H).
^{13}C-NMR (75 MHz, $CDCl_3$) δ (ppm): 175.4, 157.1, 154.8, 132.9, 131.8, 129.4, 126.2, 115.9, 79.4, 71.2, 66.2, 63.0, 39.9, 38.5, 31.7, 29.1, 28.5.
Physical State: light yellow oil.
HRMS (ESI-TOF): calc'd for $C_{25}H_{34}ClNNaO_6$ $[M+Na]^+$: 502.1972; found 502.1964.
TLC: R_f = 0.5 (40% EtOAc in Hexane).

Compound 30

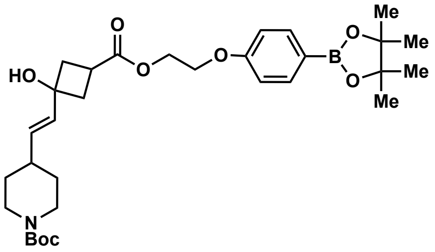

Following the general procedure A on a 0.1 mmol scale: Co(salen)-**4** (6.0 mg, 10 µmol), $TBABF_4$ (60 mg), HFIP (210 µL, 2.0 mmol), H_2O (3.6 µL, 0.2 mmol) and *iso*-BuOH (2.5 mL), with zinc as anode and tin as cathode under the electrolysis of 5 mA for 24F/mol. Compound **30** was purified by P-TLC (silica, 50% EtOAc in Hexane) to afford 45.0 mg (79%) as a colorless oil. E:Z = 87:13 (0% Alkane).

^{1}H-NMR (300 MHz, $CDCl_3$) δ (ppm): 7.77 (d, *J* = 8.6 Hz, 2H), 6.92 (d, *J* = 8.6 Hz, 2H), 5.69 (d, *J* = 3.5 Hz, 2H), 4.52 – 4.40 (m, 2H), 4.25 – 4.20 (m, 2H), 4.11 (d, *J* = 13.7 Hz, 2H), 2.76 (t, *J* = 8.4 Hz, 3H), 2.58 – 2.46 (m, 2H), 2.44 – 2.33 (m, 2H), 2.20 – 2.12 (m, 1H), 1.74 – 1.64 (m, 2H), 1.48 (s, 9H), 1.35 (s, 11H).

^{13}C-NMR (101 MHz, $CDCl_3$) δ (ppm): 175.5, 161.0, 154.8, 136.6, 132.9, 131.8, 113.9, 83.6, 79.4, 71.1, 65.7, 63.1, 42.5, 40.0, 38.5, 31.7, 29.1, 28.5, 26.9, 24.9.

^{11}B NMR (96 MHz, $CDCl_3$) δ (ppm): 32.08.

Physical State: light yellow oil.

HRMS (ESI-TOF): calc'd for $C_{31}H_{46}BNNaO_8$ $[M+Na]^+$: 594.3214; found 594.3224.

TLC: R_f = 0.5 (50% EtOAc in Hexane).

Compound 31

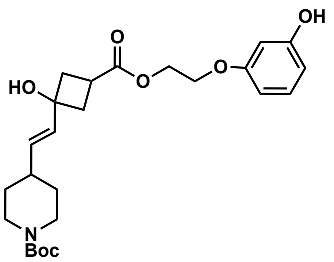

Following the general procedure A on a 0.1 mmol scale: Co(salen)-**4** (9.0 mg, 15 µmol), $TBABF_4$ (60 mg), HFIP (210 µL, 2.0 mmol), H_2O (3.6 µL, 0.2 mmol) and *iso*-BuOH (2.5 mL), with zinc as anode and tin as cathode under the electrolysis of 5 mA for 24F/mol. Compound **31** was purified by P-TLC (silica, 50% EtOAc in Hexane) to afford 37.8 mg (82%) as a colorless oil. E:Z = 96:4 (0% Alkane).

^{1}H-NMR (300 MHz, $CDCl_3$) δ (ppm): 7.15 – 7.12 (m, 1H), 6.53 – 6.46 (m, 3H), 5.70 – 5.59 (m, 2H), 4.48 – 4.45 (m, 2H), 4.21 – 4.13 (m, 4H), 2.79 – 2.52 (m, 3H), 2.52 – 2.41 (m, 4H), 2.20 – 2.16 (m, 1H), 1.72 – 1.61 (m, 2H), 1.48 (s, 9H), 1.36 – 1.32 (m, 2H).
^{13}C-NMR (101 MHz, $CDCl_3$) δ (ppm): 175.52, 159.80, 157.29, 154.98, 132.89, 131.73, 130.17, 108.54, 106.83, 102.41, 79.66, 71.21, 65.97, 63.37, 42.52, 39.94, 38.41, 31.68, 29.16, 28.49.
Physical State: colorless oil.
HRMS (ESI-TOF): calc'd for $C_{25}H_{35}NNaO_7$ $[M+Na]^+$: 484.2311; found 484.2302.
TLC: R_f = 0.5 (50% EtOAc in Hexane).

Compound 32

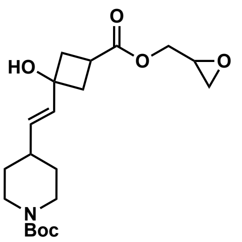

Following the general procedure A on a 0.1 mmol scale: Co(salen)-**4** (9.0 mg, 15 µmol), $TBABF_4$ (60 mg), HFIP (210 µL, 2.0 mmol), H_2O (3.6 µL, 0.2 mmol) and *iso*-BuOH (2.5 mL), with zinc as anode and tin as cathode under the electrolysis of 5 mA for 24F/mol. Compound **32** was purified by P-TLC (silica, 50% EtOAc and Hexane) to afford 31.2 mg (82%) as a colorless oil. E:Z = 89:11 (0% Alkane).

^{1}H-NMR (300 MHz, $CDCl_3$) δ (ppm): 5.74 – 5.54 (m, 2H), 4.45 (dd, *J* = 12.3, 2.9 Hz, 1H), 4.12 – 4.08 (m, 2H), 3.96 – 3.90 (m, 1H), 3.70 – 3.60 (m, 1H), 3.23 – 3.19 (m, 1H), 2.85 (t, *J* = 4.5 Hz, 1H), 2.77 – 2.64 (m, 4H), 2.54 – 2.36 (m, 5H), 1.69 – 1.63 (m, 4H), 1.46 (s, 9H).
^{13}C-NMR (75 MHz, $CDCl_3$) δ (ppm): 175.19, 154.86, 132.96, 131.77, 79.40, 77.45, 77.02, 76.60, 71.12, 65.30, 49.31, 44.63, 42.46, 39.97, 38.49, 31.72, 29.01, 28.47.
Physical State: colorless oil.
HRMS (ESI-TOF): calc'd for $C_{20}H_{31}NNaO_6$ $[M+Na]^+$: 404.2049; found 404.2038.
TLC: R_f = 0.5 (50% EtOAc in Hexane).

Compound 38

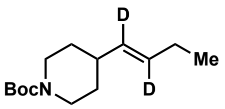

Following the general procedure B on a 0.1 mmol scale: Co(salen)-**4** (3.0 mg, 5 μmol), $TBABF_4$ (60 mg), HFIP-OD (210 μL, 2.0 mmol), and 2-propanol-OD (2.5 mL), with zinc as anode and tin as cathode under the electrolysis of 5 mA for 11 F. Compound **38** purified by P-TLC (silica, 7:3 Hexane: EtOAc) to afford the product as pale yellow oil. 65% NMR Yield, E:Z = 99:1.

^{1}H-NMR (400 MHz, CDCl3) δ (ppm): 5.48 – 5.29 (m, 2D/2H), 4.05 (s, 2H), 2.72 (t, J = 12.1 Hz, 2H), 2.09 – 1.94 (m, 3H), 1.63 (d, J = 13.2 Hz, 2H), 1.45 (s, 9H), 1.21 (dd, J = 12.5, 4.2 Hz, 2H), 0.96 (t, J = 7.4 Hz, 3H).
^{13}C-NMR (101 MHz, $CDCl_3$) δ (ppm): 154.91, 133.08, 130.60, 79.23, 38.66, 32.04, 29.71, 28.48, 25.43, 13.86.
^{2}H-NMR (61 MHz, $CHCl_3/CDCl_3$) δ (ppm): 5.49, 5.38.
Physical State: pale yellow oil.
HRMS (ESI-TOF): calc'd for $C_{14}H_{23}D_2NO_2$ $[M+Na]^+$: 264.1909; found 264.1840.
TLC: R_f = 0.6 (4:1 Hexane: EtOAc).

Compound 39

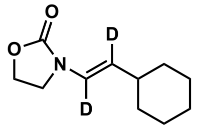

Following the general procedure B on a 0.1 mmol scale: Co(salen)-**4** (3.0 mg, 5 μmol), $TBABF_4$ (60 mg), HFIP-OD (210 μL, 2.0 mmol), and 2-propanol-OD (2.5 mL), with zinc as anode and tin as cathode under the electrolysis of 5 mA for 8 F. Compound **39** was purified by P-TLC (silica, 8:2 Hexane: EtOAc). 80% NMR yield, E:Z = 95:5.

^{1}H-NMR (400 MHz, $CDCl_3$) δ (ppm): 6.63 (d, J = 11.4 Hz, 1D/1H), 4.75 (d, J = 7.1 Hz, 1D/1H), 4.41 (dd, J = 8.9, 7.3 Hz, 2H), 3.67 (dd, J = 8.8, 7.3 Hz, 2H), 2.01 (dd, J = 15.5, 7.0 Hz, 1H), 1.78 – 1.61 (m, 5H), 1.26 (ddd, J = 11.9, 7.7, 2.9 Hz, 2H), 1.12 (ddd, J = 33.5, 13.7, 7.4 Hz, 3H).
^{13}C-NMR (101 MHz, $CDCl_3$) δ (ppm): 155.52, 122.22, 117.34, 62.08, 42.57, 38.34, 33.67, 26.05, 26.03.
^{2}H-NMR (61 MHz, $CHCl_3$/$CDCl_3$) δ (ppm): 6.64, 4.80.
Physical State: light yellow oil.
HRMS (ESI-TOF): calc'd for $C_{11}H_{15}H_2NO_2Na$ $[M+Na]^+$: 220.1283; found 220.1284.
TLC: R_f = 0.5 (20% EtOAc in Hexane).

Compound 40

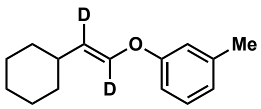

Following the general procedure B on a 0.1 mmol scale: Co(salen)-**4** (6.0 mg, 10 μmol), $TBABF_4$ (60 mg), HFIP-OD (210 μL, 0.33 mmol), and 2-propanol-OD (2.5 mL), with zinc as anode and tin as cathode under the electrolysis of 5 mA for 10F/mol. Compound Compound **40** was purified by P-TLC (silica, 100% Hexane). 89% NMR yield, E:Z = 99:1.

^{1}H-NMR (400 MHz, $CDCl_3$) δ (ppm): 7.18 (t, J = 8.0 Hz, 1H), 6.84 (d, J = 7.5 Hz, 1H), 6.78 (d, J = 7.2 Hz, 2H), 6.40 (d, J = 9.5 Hz, 1D/1H (80:20)), 5.34 (m, 1D/1H (80:20)), 2.34 (s, 3H), 2.10 – 1.94 (m, 1H), 1.79 – 1.63 (m, 4H), 1.34 – 1.25 (m, 4H), 1.20 – 1.12 (m, 2H).
^{13}C-NMR (101 MHz, $CDCl_3$) δ (ppm): 157.53, 139.67, 129.28, 129.23, 123.11, 116.99, 116.91, 113.26, 36.54, 33.72, 26.07, 21.47, 21.43.
^{2}H-NMR (61 MHz, $CHCl_3$/$CDCl_3$) δ (ppm): 6.43, 5.38.
Physical State: colorless oil.
HRMS (ESI-TOF): calc'd for $C_{15}H_{19}D_2O$ $[M+H]^+$: 219.1718; found 219.1733.
TLC: R_f = 0.7 (100% Hexane).

Compound 41

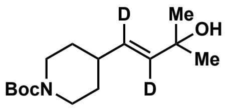

Following the general procedure B on a 0.1 mmol scale: Co(salen)-**4** (3.0 mg, 5 μmol), $TBABF_4$ (60 mg), HFIP-OD (210 μL, 2.0 mmol), and 2-propanol-OD (2.5 mL), with zinc as anode and tin as cathode under the electrolysis of 5 mA for 20 F. Compound **41** was obtained in 69% NMR yield with 82% deuterium labeling was purified by P-TLC (silica, 8:2 Hexane:EtOAc). 69% NMR yield, E:Z = 94:6.

^{1}H-NMR (400 MHz, $CDCl_3$) δ (ppm): 5.65 – 5.50 (m, 2D/2H), 4.08 (s, 2H), 2.72 (t, *J* = 12.2 Hz, 2H), 2.14 – 2.02 (m, 1H), 1.65 (d, *J* = 13.2 Hz, 2H), 1.46 (s, 9H), 1.30 (s, 6H), 1.25 (d, *J* = 11.6 Hz, 2H).
^{13}C-NMR (126 MHz, $CDCl_3$) δ (ppm): 154.87, 136.54, 130.81, 79.32, 70.57, 38.27, 31.81, 29.89, 29.87, 28.48.
^{2}H-NMR (61 MHz, $CHCl_3/CDCl_3$) δ (ppm): 5.63.
Physical State: pale yellow oil.
HRMS (ESI-TOF): calc'd for $C_{15}H_{25}D_2NO_3Na$ $[M+Na]^+$: 294.2014; found 294.1841.
TLC: R_f = 0.4 (7:3 Hexane: EtOAc).

Compound 42

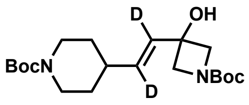

Following the general procedure B on a 0.1 mmol scale: Co(salen)-**4** (6.0 mg, 10 µmol), $TBABF_4$ (60 mg), HFIP-OD (210 µL, 2.0 mmol), and 2-propanol-OD (2.5 mL), with zinc as anode and tin as cathode under the electrolysis of 5 mA for 18 F. Compound **42** purified by P-TLC (silica, 7:3 Hexane: EtOAc) to afford a pale yellow oil, 76 % NMR yield, E:Z = 90:10.

^{1}H-NMR (300 MHz, $CDCl_3$) δ 5.76 (d, J = 2.7 Hz, 0.2H), 4.17 – 4.08 (m, 2H), 4.05 – 3.89 (m, 4H), 2.83 – 2.68 (m, 2H), 1.69 (d, J = 12.2 Hz, 1H), 1.48 (s, 9H), 1.46 (s, 9H), 1.34 (dd, J = 12.4, 4.2 Hz, 2H).

^{13}C-NMR ^{13}C NMR (201 MHz, $CDCl_3$) δ 159.1, 157.5, 136.7, 133.0, 82.4, 82.1, 72.4, 65.26, 46.3, 40.9, 34.2, 31.1, 31.0.

^{2}H-NMR (61 MHz, $CHCl_3$/$CDCl_3$): δ (ppm): 5.77.

Physical State: colorless oil.

HRMS (ESI-TOF): calc'd for $C_{20}H_{32}D_2N_2O_5Na$ $[M+Na]^+$: 407.2491; found 407.2473

TLC: R_f = 0.6 (3:7 EtOAc: Hexane).

Compound 44

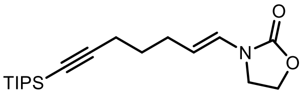

Following the general procedure A on a 0.1 mmol scale, using Co(salen)-**4** (6.0 mg, 10 μmol), $TBABF_4$ (60 mg), HFIP (210 μL, 2.0 mmol), H_2O (3.6 μL, 0.2 mmol) and *iso*-BuOH (2.5 mL), with zinc as anode and tin as cathode under the electrolysis of 5 mA for 3F/mol. Compound **44** was purified by P-TLC (30:70 Ethyl acetate: Hexane) to afford 25.4 mg (76%) as a yellow oil. E:Z = 99:1 (0% Alkane).

^{1}H NMR (800 MHz, $CDCl_3$) δ (ppm): 6.67 (d, J = 14.3 Hz, 1H), 4.78 (dt, J = 14.3, 7.2 Hz, 1H), 4.43 – 4.41 (m, 2H), 3.69 – 3.68 (m, 2H), 2.27 – 2.25 (m, 2H), 2.21 – 2.19 (m, 2H), 1.63 – 1.60 (m, 2H), 1.05 (s, 18H), 1.03 – 1.00 (m, 3H).
^{13}C NMR (201 MHz, $CDCl_3$) δ (ppm): 158.01 (s), 127.16, 112.73, 111.06, 83.33, 64.75, 45.25, 31.95, 31.41, 21.87, 21.30, 13.93.
Physical State: Yellow oil.
HRMS (ESI-TOF): calc'd for $C_{19}H_{34}NO_2Si$ $[M+H]^+$: 336.2359; found 336.2357.
TLC: R_f = 0.5 (30% Ethyl acetate in Hexane).

<u>Compound 45</u>

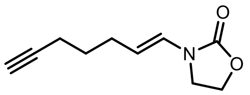

To a solution of **44** (28.0 mg,0.29 mmol) in dry THF (4 mL) was added TBAF in THF (1.0M, 0.44 mL, 0.44 mmol) at room temperature and stirred for 4 h, being monitored by TLC. After full consumption of the starting material, the reaction was quenched by the addition of water, and the mixture was extracted with Et_2O (3 × 20 mL). The combined organic layer was washed with water and brine, dried, and concentrated under vacuum. Compound **45** was purified by P-TLC (30:70 Ethyl acetate: Hexane) to afford 11 mg (61%) as a colorless oil.

^{1}H-NMR (400 MHz, $CDCl_3$) δ (ppm): 6.71 (d, *J* = 14.3 Hz, 1H), 4.80 (dt, *J* = 14.3, 7.2 Hz, 1H), 4.47 – 4.43 (m, 2H), 3.73 – 3.69 (m, 2H), 2.25 – 2.19 (m, 4H), 1.98 (t, *J* = 2.6 Hz, 1H), 1.68 – 1.61 (m, 2H).
^{13}C NMR (101 MHz, $CDCl_3$) δ (ppm): 155.38, 124.69, 109.81, 84.01, 68.68, 62.11, 42.59, 28.75, 28.72,17.70.
Physical State: colorless oil.
HRMS (ESI-TOF): calc’d for $C_{10}H_{14}NO_2$ $[M+H]^+$: 180.1025; found 180.1026.
TLC: R_f = 0.5 (7:3 Hexane: EtOAc).

Compound 46

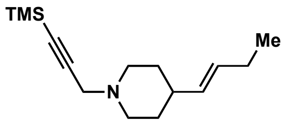

Following the general procedure A on a 0.1 mmol scale, using Co(salen)-**4** (6.0 mg, 10 μmol), $TBABF_4$ (60 mg), HFIP (210 μL, 2.0 mmol), H_2O (3.6 μL, 0.2 mmol) and *iso*-BuOH (2.5 mL), with zinc as anode and tin as cathode under the electrolysis of 5 mA for 9 F/mol. Compound **46** was purified by P-TLC (10:90 Ethyl acetate: Hexane) to afford 15.6 mg (63%) as a yellow oil. E:Z = 97:3 (0% Alkane).

^{1}H NMR (400 MHz, $CDCl_3$) δ (ppm): 5.45 (dt, *J* = 15.4, 6.0 Hz, 1H), 5.34 (dd, *J* = 15.5, 6.5 Hz, 1H), 3.28 (s, 2H), 2.88 (d, *J* = 11.5 Hz, 2H), 2.16 (td, *J* = 11.7, 2.2 Hz, 2H), 1.99 (p, *J* = 7.2 Hz, 2H), 1.69 (d, *J* = 12.9 Hz, 2H), 1.43 (ddd, *J* = 24.8, 12.3, 3.7 Hz, 2H), 0.96 (t, *J* = 7.4 Hz, 3H), 0.17 (s, 9H). (Fraction of inseparable *Z* isomer mixed with the pure *E* isomer)
^{13}C NMR (101 MHz, $CDCl_3$) δ (ppm): 133.67, 130.46, 101.45, 89.57, 52.49, 48.37, 38.34, 32.31, 25.63, 13.96, 0.07.
Physical State: Yellow oil.
HRMS (ESI-TOF): calc'd for $C_{15}H_{28}NSi$ $[M+H]^+$: 250.1991; found 250.1998.
TLC: R_f = 0.6 (10% Ethyl Acetate in Hexane).

Compound 47

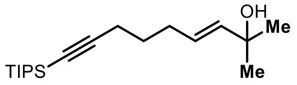

Following the general procedure A on a 0.1 mmol scale, using Co(salen)-**4** (6.0 mg, 10 µmol), $TBABF_4$ (60 mg), HFIP (210 µL, 2.0 mmol), H_2O (3.6 µL, 0.2 mmol) and *iso*-BuOH (2.5 mL), with zinc as anode and tin as cathode under the electrolysis of 5 mA for 24F/mol. Compound **47** was purified by P-TLC (10:90 Ethyl acetate: Hexane) to afford 18 mg (67%) as a colorless oil. E:Z = 92:8 (0% Alkane).

^{1}H NMR (300 MHz, $CDCl_3$) δ (ppm): 5.68 – 5.54 (m, 2H), 2.25 (t, *J* = 7.0 Hz, 2H), 2.19 – 2.13 (m, 2H), 1.65 – 1.55 (m, 2H), 1.31 (s, 6H), 1.25 (s, 1H), 1.10 – 1.04 (m, 21H).
^{13}C NMR (126 MHz, $CDCl_3$) δ (ppm): 138.86, 126.10, 108.72, 80.44, 70.65, 31.05, 29.85, 28.48, 19.23, 18.64, 11.30.
Physical State: Colorless oil.
HRMS (ESI-TOF): calc'd for $C_{19}H_{36}OSiNa$ $[M+Na]^+$: 331.2433; found 331.2433.
TLC: R_f = 0.6 (10% Ethyl Acetate in Hexane).

Compound 48

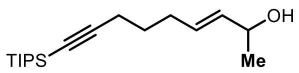

Following the general procedure A on a 0.1 mmol scale, using Co(salen)-**4** (6.0 mg, 10 μmol), $TBABF_4$ (60 mg), HFIP (210 μL, 2.0 mmol), H_2O (3.6 μL, 0.2 mmol) and *iso*-BuOH (2.5 mL), with zinc as anode and tin as cathode under the electrolysis of 5 mA for 12F/mol. Compound **48** was purified by P-TLC (10:90 Ethyl acetate: Hexane) to afford 25 mg (86%) as a colorless oil. E:Z = 99:1 (0% Alkane).

^{1}H NMR (300 MHz, $CDCl_3$) δ (ppm): 5.70 – 5.53 (m, 2H), 4.31 – 4.27 (m, 1H), 2.28 (t, J = 7.0 Hz, 2H), 2.19 (dd, J = 14.1, 6.6 Hz, 2H), 1.68 – 1.61 (m, 2H), 1.42 (s, 1H), 1.28 (d, J = 6.3 Hz, 3H), 1.08 (s, 21H).

^{13}C NMR (75 MHz, $CDCl_3$) δ (ppm): 135.04, 129.87, 108.63, 80.51, 68.88, 31.02, 28.34, 23.44, 19.27, 18.64, 11.30.

Physical State: Colorless oil.

HRMS (ESI-TOF): calc'd for $C_{18}H_{34}OSiNa$ $[M+Na]^+$: 317.2276; found 317.2278.

TLC: R_f = 0.6 (10% Ethyl Acetate in Hexane).

Compound 53

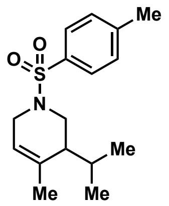

Following the general procedure A on a 0.1 mmol scale, using Co(salen)-**4** (6.0 mg, 10 μmol), $TBABF_4$ (60 mg), HFIP (210 μL, 2.0 mmol), H_2O (3.6 μL, 0.2 mmol) and acetone (2.5 mL), with zinc as anode and tin as cathode under the electrolysis of 5 mA for 6F/mol. Yield was determined using ^{1}H-NMR (22% yield), and Compound **53** was purified by preparative HPLC.

^{1}H NMR (500 MHz, $CDCl_3$) δ (ppm): 7.68 (d, *J* = 8.3 Hz, 2H), 7.34 (d, *J* = 7.9 Hz, 2H), 5.41 (tt, *J* = 3.4, 1.5 Hz, 1H), 3.63 (ddt, *J* = 15.8, 3.9, 2.1 Hz, 1H), 3.37 (dd, *J* = 11.6, 4.6 Hz, 1H), 3.32 (ddt, *J* = 15.9, 4.0, 2.1 Hz, 1H), 2.81 (dd, *J* = 11.6, 4.7 Hz, 1H), 2.45 (s, 3H), 2.12 – 2.05 (m, 1H), 2.02 (m, *J* = 7.5 Hz, 1H), 1.70 (d, *J* = 1.9 Hz, 3H), 1.07 (d, *J* = 7.0 Hz, 3H), 0.90 (d, *J* = 7.0 Hz, 3H).

^{13}C NMR (126 MHz, $CDCl_3$) δ (ppm): 143.32, 135.55, 133.08, 129.54, 127.75, 118.33, 44.89, 44.71, 43.58, 28.50, 21.94, 21.51, 21.01, 18.04.

Physical State: Colorless oil.

HRMS (ESI-TOF): calc'd for $C_{16}H_{23}NO_2SNa$ $[M+Na]^+$: 316.1350; found 316.1347.

Compound 54

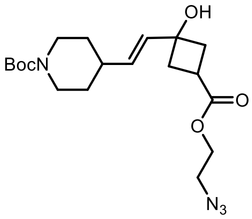

Following the general procedure C on a 0.1 mmol scale, using Co(salen)-**5** (6.0 mg, 10 μmol), TBHP (50 μl), $PhSiH_3$ (55 μl), and 2.5 mL of dry and degassed ethanol, the mixture was stirred at room temperature for 12 h. Compound **54** was purified by P-TLC (Hex/EtOAc,100:0 - 60:40) to afford 22 mg (56%) as a pale yellow oil. E:Z = 90:10 (0% Alkane).

^{1}H NMR (400 MHz, $CDCl_3$) δ (ppm): 5.76 – 5.55 (m, 2H), 4.28 – 4.29 (m, 2H), 4.11 (s, 2H), 3.50 – 3.47 (m, 2H), 2.72 (dd, J = 16.8, 8.2 Hz, 3H), 2.54 – 2.49 (m, 2H), 2.44 – 2.39 (m, 2H), 2.25 (s, 1H), 2.15 (t, J = 12.4 Hz, 1H), 1.68 (d, J = 12.1 Hz, 2H), 1.46 (s, 9H), 1.35 – 1.30 (m, 2H).
^{13}C NMR (101 MHz, $CDCl_3$) δ (ppm): 175.04, 154.84, 132.99, 131.72, 79.40, 71.07, 63.43, 49.77, 39.95, 38.49, 31.72, 29.71, 29.00, 28.48.
Physical State: Pale yellow oil.
HRMS (ESI-TOF): calc'd for $C_{19}H_{30}N_4NaO_5$ $[M+Na]^+$: 417.2114; found 411.2103.
TLC: R_f = 0.5 (40% Ethyl acetate: Hexane).

Compound 55

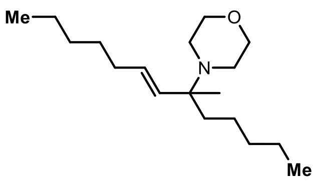

Following the general procedure C on a 0.1 mmol scale, using Co(salen)-**5** (6.0 mg, 10 µmol), TBHP (50 µl), $PhSiH_3$ (55 µl), and 2.5 mL of dry and degassed ethanol, the mixture was stirred at room temperature for 12 h. Compound **55** was purified by P-TLC (10:90 Ethyl acetate:Hexane) to afford 17.5 mg (61%) as a pale yellow oil. E:Z = 97:3 (0% Alkane).

^{1}H NMR (800 MHz, $CDCl_3$) δ (ppm): 5.40 – 5.32 (m, 2H), 3.67 (s, 4H), 2.51 (s, 4H), 2.03 (dd, *J* = 14.2, 7.0 Hz, 2H), 1.42 – 1.34 (m, 4H), 1.32 – 1.21 (m, 10H), 1.01 (s, 3H), 0.88 (dq, *J* = 14.5, 7.2 Hz, 6H). (Inseparable *Z* isomer is mixed with pure *E* isomer)

^{13}C NMR (201 MHz, $CDCl_3$) δ (ppm): 138.15, 132.69, 70.55, 62.60, 49.09, 41.62, 35.32, 35.27, 34.07, 31.96, 26.19, 25.31, 25.16, 19.78, 16.78, 16.76.

Physical State: Pale yellow oil.

HRMS (ESI-TOF): calc'd for $C_{18}H_{36}NO$ $[M+H]^+$: 282.2797; found 282.2796.

TLC: R_f = 0.6 (10% Ethyl Acetate in Hexane).

2. Additional data

Control experiments

Dissolving metal reduction of compound **1**:

Sodium reduction:

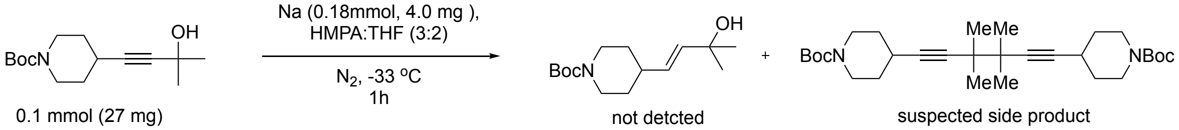

A 10 mL oven-dried drum vial containing a magnetic stirring bar charged with 4.0 mg of metallic sodium, followed by the addition of 0.550 mL of *tert*-BuOH at -33 ºC under an N_2 atmosphere. To it, a solution of Compound **1** (1.0 mmol, 267 mg) in 2.0 mL THF was added, followed by the addition of 3.0 mL of HMPA to maintain the total solvent ratio 3:2 (v/v). Afterwards, the reaction mixture was stirred at the same temperature for 1 h. Then, the reaction mixture was quenched with 5.0 mL of water and extracted with diethyl ether (3 × 10 mL). The combined organic layer was dried over Na_2SO_4 and concentrated under vacuum. Check the crude NMR using a quantitative amount of $CHBr_3$ as the internal standard. Deoxygenative dimerization was obtained in the crude NMR.[14]

Lithium reduction of compound **1**:

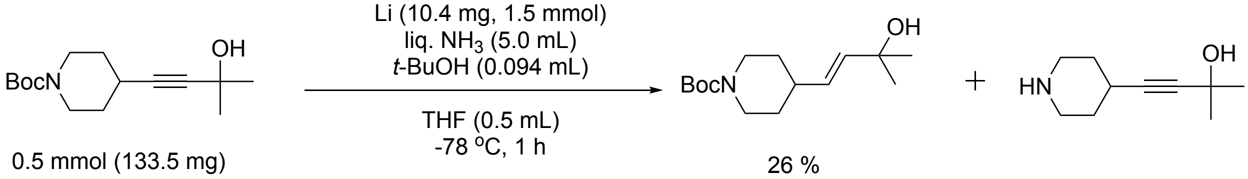

An oven-dried three-necked round-bottom flask fitted with a condenser was placed in a dry-ice-acetone bath (-78 ºC) under an argon atmosphere using the Schlenk line. To it, 5.0 mL of liquid ammonia was added dropwise under the same conditions. Next, 10.4 mg of metallic lithium was added, and immediately the color of the solution turned blue. A solution of 133.5 mg of compound **1** (0.5 mmol) in 0.5 mL dry THF was added to the blue-colored solution

under an argon atmosphere at -78 ºC, and subsequently, 94 µl of *tert*-BuOH was added to the mixture. The reaction mixture was allowed to stir under the same conditions for 1 h. Then, the reaction was warmed to room temperature, and ammonia was evaporated under a flow of argon gas, followed by the quenching of lithium by slow addition of water, and finally extracted with diethyl ether (3 × 10 mL). Combined organic layers were washed with brine and concentrated under vacuum. Checked the NMR of the crude using a quantitative amount of $CHBr_3$ as the internal standard. 26% *E*-alkene was obtained along with the Boc-deprotected alkynes.[15]

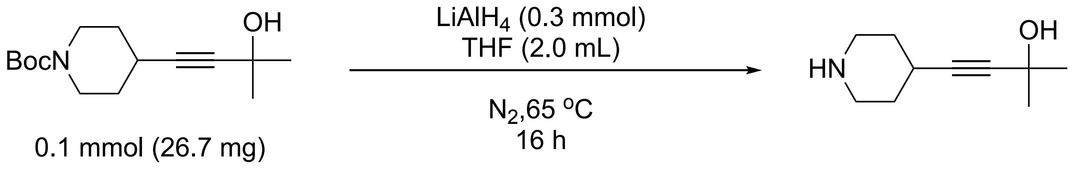

In a 10 mL oven-dried drum vial containing a magnetic stirring bar, Compound **1** (0.1 mmol, 26.7 mg) and $LiAlH_4$ (0.3 mmol, 11.0 mg) were added under an N_2 atmosphere at room temperature. To it, 2.0 mL of dry and degassed THF was added, and the reaction mixture was allowed to stir for 16 h at 65 ºC. The reaction mixture was quenched with water and extracted with diethyl ether (3 x 10 mL). The combined organic layers were washed with brine, dried over Na_2SO_4, and concentrated under vacuum. Check the crude NMR using a quantitative amount of $CHBr_3$ as the internal standard. Boc-deprotected alkyne was observed.[16]

<u>LAH reduction of compound **S17**:</u>

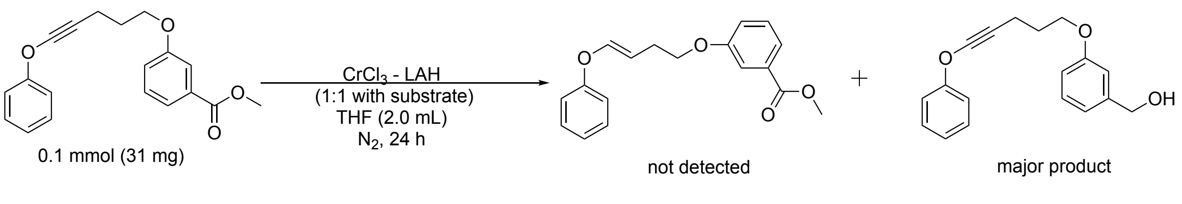

An oven-dried 10 mL vial containing a magnetic stirring bar was charged with anhydrous $CrCl_3$ (0.1 mmol, 16 mg) and $LiAlH_4$ (0.1 mmol, 3.8 mg), and the vial was sealed with a

rubber septum. Then, the vial was evacuated under a high vacuum, backfilled with nitrogen gas for 3 cycles, and placed in a dry ice-acetone bath to cool the mixture at -78 ºC. A solution of compound **S17** (0.1 mmol, 31mg) in 2.0 mL of dry and degassed THF was added dropwise at the same temperature. After 10 min, the reaction mixture was allowed to warm at room temperature. After 24 h, the reaction was quenched with water and extracted with diethyl ether (3 x 10 mL). The combined organic layers were washed with brine, dried over Na_2SO_4, and concentrated under vacuum. Check the crude NMR using a quantitative amount of $CHBr_3$ as the internal standard. Only the reduction of ester was observed in the crude NMR.[17]

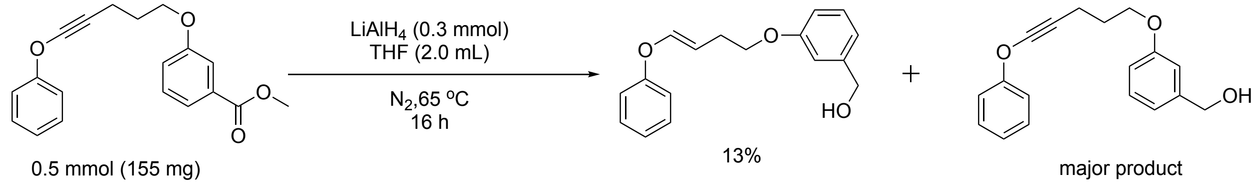

In a 10 mL oven-dried drum vial containing a magnetic stirring bar, Compound **S21** (0.1 mmol, 31 mg) and $LiAlH_4$ (0.3 mmol, 11.0 mg) were added under N_2 atmosphere at room temperature. To it, 2.0 mL of dry and degassed THF was added, and the reaction mixture was allowed to stir for 16 h at 65 ºC. The reaction mixture was quenched with water and extracted with diethyl ether (3 x 10 mL). The combined organic layers were washed with brine, dried over Na_2SO_4, and concentrated under vacuum. Check the crude NMR using a quantitative amount of $CHBr_3$ as the internal standard. Complete reduction of ester was obtained along with 13% alkene corresponding to the alkyne (after reduction of esters).[16]

RDE experiments:

All RDE analyses were carried out using a BioLogic VSP multi-channel potentiostat with a BlueRev rotating disk electrode and an RC-10K system connected to it. A solution of 1 mM **Co-4** in 20 mL *iso*-BuOH, with 0.1 M $TBABF_4$ as a supporting electrolyte, was used for RDE measurements, with increasing equivalents of HFIP and/or substrate. The linear sweep voltammetry (LSV) was performed at a scanning rate of 10 mV/s, with rotating rates of 100 rpm and 250 rpm. The working electrode was glassy carbon, the counter electrode was a Pt wire, and the reference electrode was a Pt wire in Fc/Fc^+ 4 mM solution in acetonitrile. A CV using the same electrode system was conducted first to assess the potential windows needed due to the difference between the reference electrode used here and the CV experiment. Before every LSV, the system was purged using N_2 with a 99.9999% purity grade.

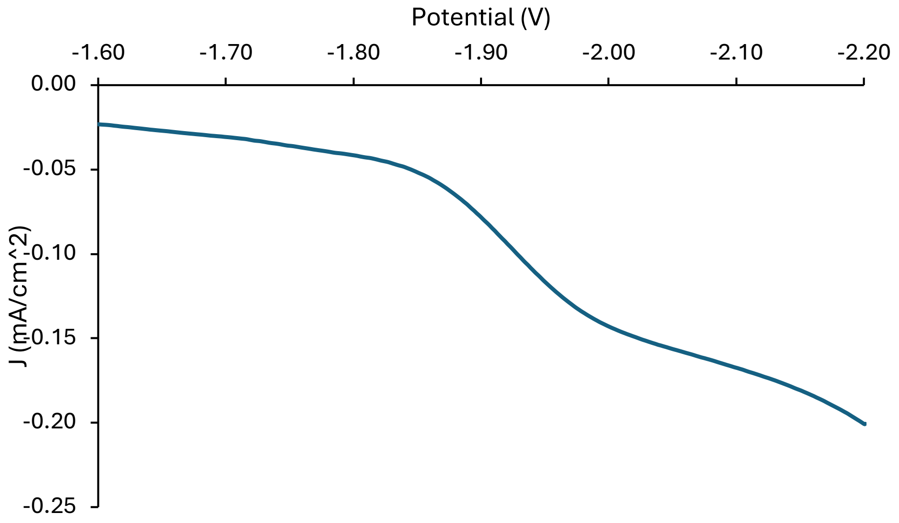

Figure S1: RDE analysis of **Co-4**.

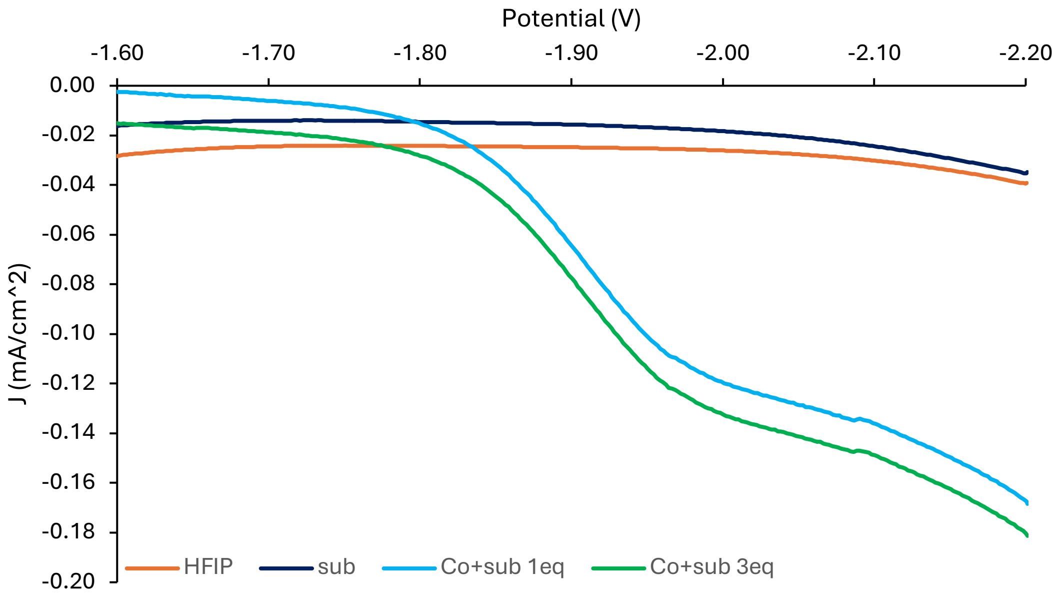

Figure S2: RDE analysis of HFIP (orange), substrate **49** (purple), **Co-4,** and **49** 1 equiv. (blue), and **Co-4** + **49** 3 equiv. (green).

Spectroelectrochemistry experiments:

In situ, spectroelectrochemical measurements were performed in a standard quartz cell with a 1 mm path length, a working electrode prepared from Pt gauze, and a Pt wire counter electrode. An HP Agilent 8453 spectrometer with deuterium and tungsten lamps was used for spectroscopic measurements. A BioLogic VSP multichannel potentiostat was used for the voltammetry. A 4 mL *iso*-BuOH solution was used with 25 µM of the **Co-4**, 0.1 M $TBABF_4$, and varying amounts of HFIP and/or substrate. Absorbance data were baseline corrected by zeroing the absorbance at 700 nm.

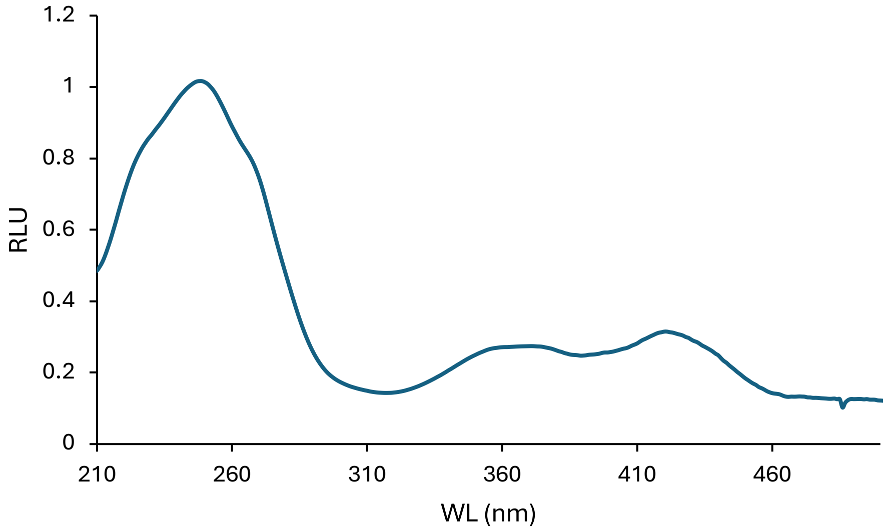

Figure S3: UV-Vis spectrum of **Co-4** (25 µM) in *iso*-BuOH (4.0 mL).

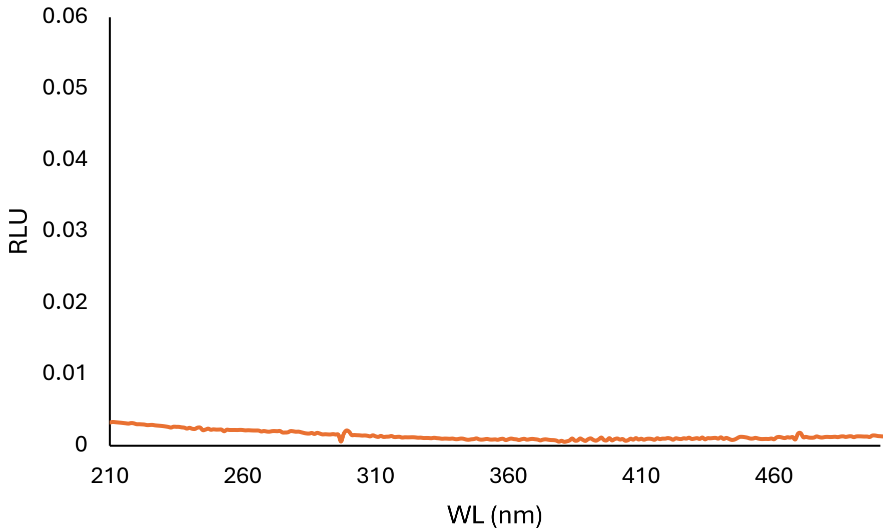

Figure S4: UV-Vis spectrum of alkyne **49** (1 mM) in *iso*-BuOH (4 mL).

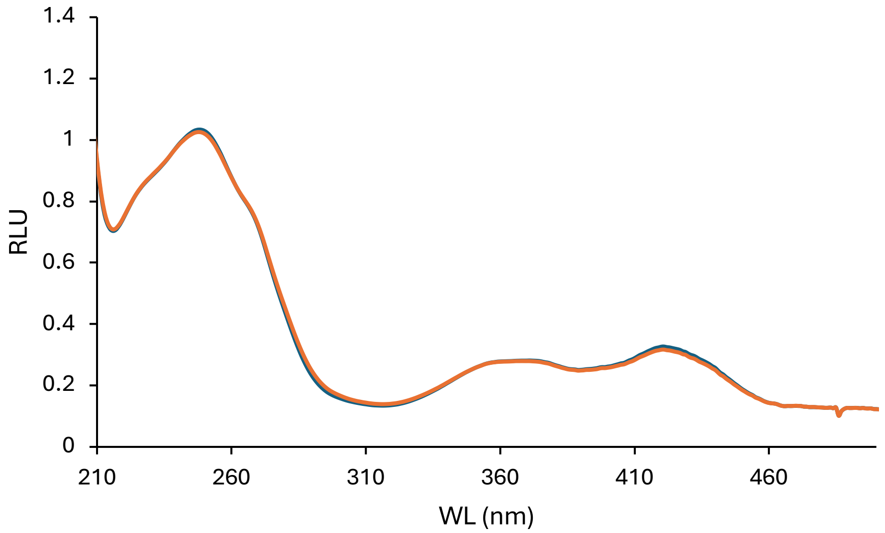

Figure S5: UV-Vis spectra of **Co-4** and alkyne **49** in *iso*-BuOH without potential (blue) and **Co-4** and alkyne **49** in *iso*-BuOH under -2 V potential (orange).

$^{1}H\{^{2}H\}$ HMBC NMR experiment:

Method description

The $^{1}H\{^{2}H\}$ HMBC NMR experiment was recorded on a 9.4T (400.35 MHz) Bruker AVANCE NEO spectrometer, with an iBBFO probe having a ^{2}H pass capability. The pulse sequence was used with 1 ms gradients calculated for ^{2}H (70%, 30% and 55.35%). The spectral width for ^{1}H was 18 ppm and for ^{2}H 10 ppm with TD of 4K and 64, respectively. The number of scans for each row was 48 with D1 of 2s, resulting in approximately 2 hours of experiment time.

Experimental Protocol

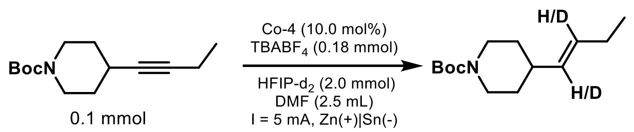

In an oven-dried 5.0 mL Electrasyn-vial containing a magnetic stirring bar and wrapped with a single layer of Teflon tape on the screw thread, the catalyst Co (Salen) (6.0 mg, 0.01 mmol), $TBABF_4$ (60.0 mg, 0.18 mmol), and the alkyne substrate (0.1 mmol) were charged. HFIP-d_2 (210 µL) and 2.5 mL of dry and degassed dimethylformamide were added. The vial was then sealed with the cap bearing a zinc anode and a tin cathode. The reaction mixture was then bubbled with N_2 gas for 5 minutes. Finally, the Electrasyn vial was fitted in an Electrasyn and electrolyzed under a constant current of 5.0 mA. After 8 F/Mol, the crude mixture was diluted with diethyl ether (20-25 mL) and washed with water (20-25 mL). After phase separation, the collected aqueous phase was extracted with diethyl ether (2 × 25 mL). The combined organic phase was washed with brine (20 mL). The organic phase was dried with Na_2SO_4 and then concentrated under a vacuum. The clean product was obtained using chromatography or P-TLC.

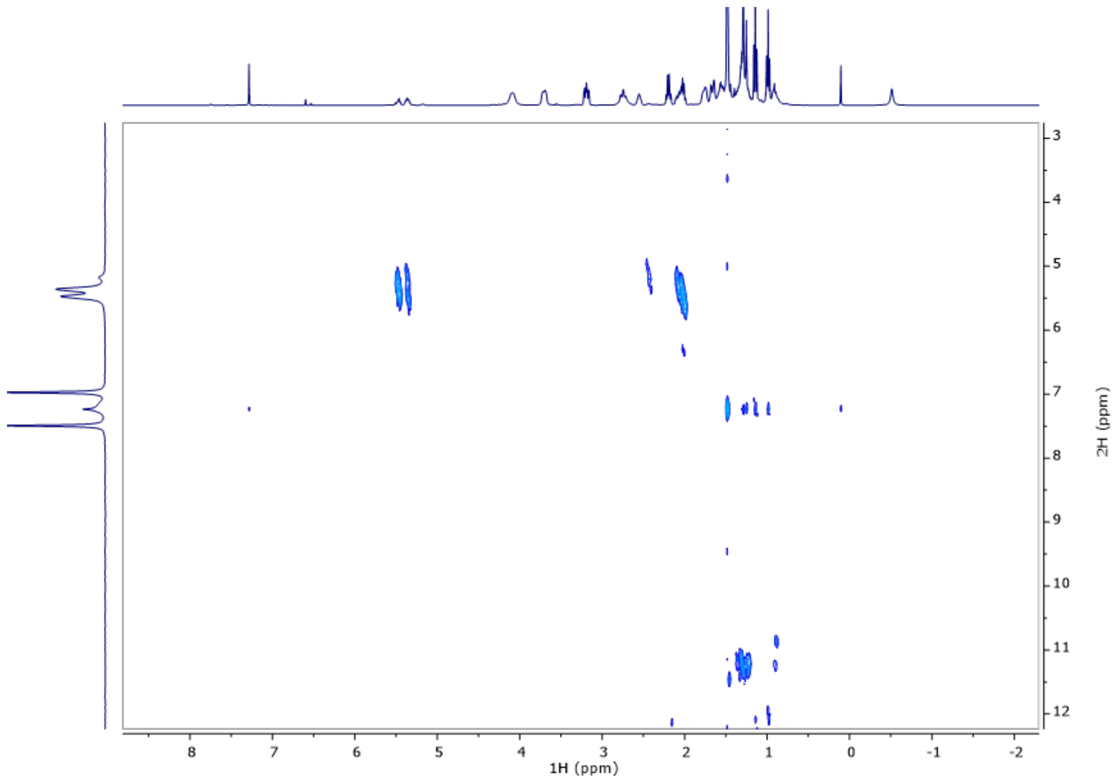

Figure S6: $^{1}H\{^{2}H\}$ HMBC NMR spectrum.

Additional experiments:

Differences between electrochemistry and chemical reductants:

Electrochemistry provides a uniquely suited platform for the controlled generation of low-valent [Co–H] species due to its precise modulation of redox potentials and decoupling of electron and proton transfer events. Specifically, in our system, the electrochemical reduction of Co(II) to Co(I) occurs at a well-defined potential (ca. –2.0 V vs Fc/Fc^{+}), enabling the selective formation of the catalytically active Co–H intermediate via protonation with HFIP under mild conditions (see Figure 5B and 5C).

In contrast, the use of heterogeneous chemical reductants generally leads to uncontrolled or non-specific electron transfer at their respective reduction potentials, which are either too reducing or insufficiently selective for the clean formation of the Co–H species.

In the case of Zn reductant, we assume that its reduction potential (ca. -1.2 V vs Fc/Fc^{+}) is not enough to form the low-valent Co(i) species. Similarly, Fe (–0.9 V vs Fc/Fc^{+}) or Mn (–1.2 V vs Fc/Fc^{+}) chemical reductants, and both cases gave no reduction product 2.

In the case of Mg (ca. –2.3 V vs Fc/Fc^{+}) reductant, we assumed, based on the reduction potentials, that it would work; however, we realized it does not. Several reasons can lead to this outcome: poor interfacial electron transfer kinetics, rapid passivation of the metal surface, competing non-selective redox processes, and lack of evidence for clean Co–H generation. In contrast, electrochemistry enables precise, stepwise control over the redox environment, which is essential for the selective formation and utilization of [Co–H] intermediate formation.

On a general note, although the differences between chemical reducants/oxidants and electrochemistry are not always clear, numerous examples of such differences are reported in the literature for other transformations.

Examples:

1- Nature, 2023, 623, 745-751.
2- Angew. Chem. Int. Ed., 2022, 61, e202208080.
3- Nat. Chem., 2020, 12, 747-754.

The Role of Water Additive in Enhancing Reaction Performance

The incorporation of water (2 equivalents) as an additive was found to significantly improve both the reproducibility and the overall yield of the electrochemical semi-hydrogenation reaction. Taking alkyne **1** as a representative example, we observed notable fluctuations in product yield when the reaction was performed without water. Specifically, five independent runs yielded products in the range of 57% to 82%, although the *E:Z* selectivity remained consistent (from 93:7 to 94:6). In all cases, the unconverted starting material accounted for the rest of the mass balance, indicating that incomplete conversion was the primary cause of reduced yields. In a way, this means that Co-H formation was inconsistent. However, upon inclusion of water as an additive, the reaction became markedly more reproducible and consistently delivered higher yields, as illustrated in Scheme 7. Interestingly, the *E:Z* selectivity remained essentially unchanged regardless of water's presence, suggesting that water does not directly participate in the stereo-determining step or alter the underlying mechanism. Instead, we propose that water modifies the efficiency of Co(II) reduction or protonation, thereby facilitating more effective formation of the active cobalt-hydride (Co–H) catalytic species and improving mass transport near the electrode surface.

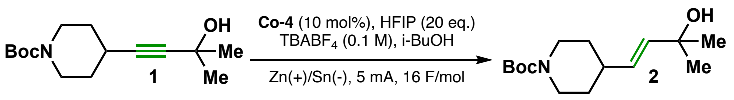

**without H_2O**	**with H_2O**
exp 1: 57% (93:7/E:Z)	exp 1: 88% (94:6/E:Z)
exp 2: 62% (94:6/E:Z)	exp 2: 87% (94:6/E:Z)
exp 3: 63% (94:6/E:Z)	exp 3: 88% (94:6/E:Z)
exp 4: 71% (93:7/E:Z)	exp 4: 88% (94:6/E:Z)
exp 5: 82% (94:6/E:Z)	

Scheme S1: Impact of water on model reaction.

Challenging substrates:

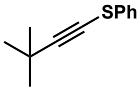

67% (20:80/E:Z)

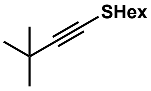

75% (25:75/E:Z)

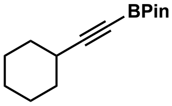

decomp.

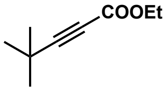

21% (90:10/E:Z)

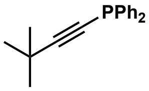

26% (89:11/E:Z)

Optimization campaign:

Catalyst study:

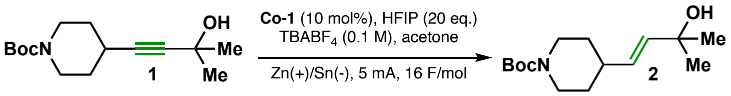

entry	deviation from above	yield (%)[a]	E/Z ratio[a]
1	none	69	36/64
2	**Co-2** catalyst instead of **Co-1**	79	31/69
3	**Co-3** catalyst instead of **Co-1**	60	70/30
4	**Co-4** catalyst instead of **Co-1**	40	84/16
5	**Co-5** catalyst instead of **Co-1**	49	83/17
6	**Co-6** instead of **Co-1**	ND	-
7	**Co-7** instead of **Co-1**	ND	-
8	**Co-8** instead of **Co-1**	ND	-
9	**Co-9** instead of **Co-1**	5	-
10	**$Co(OAc)_2$** instead of **Co-1**	ND	-
11	**$Co(NO_3)_2{\cdot}H_2O$** instead of **Co-1**	ND	-

Catalyst structure

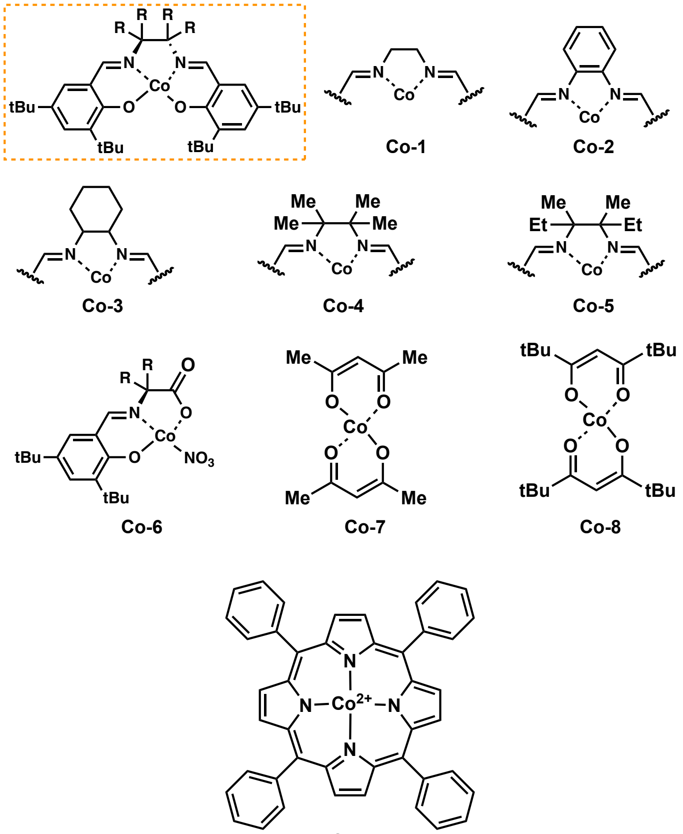

HFIP amount study:

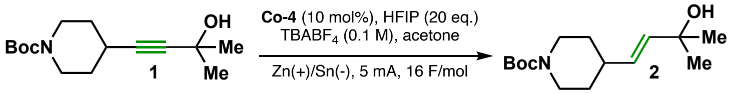

40% (84/16 E/Z)

entry	**deviation from above**	yield (%)[a]	E/Z ratio[a]
12	5 equiv. HFIP	29	85/15
13	10 equiv. HFIP	33	84/16
14	50 equiv. HFIP	37	84/16

Cathode study:

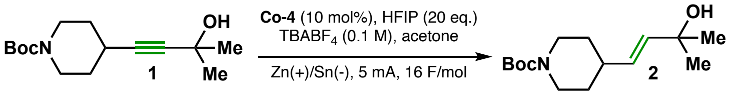

40% (84/16 E/Z)

entry	**deviation from above**	yield (%)[a]	E/Z ratio[a]
15	**Co-4**, **C** cathode instead of Sn	33	82/18
16	**Co-4**, **Ni** cathode instead of Sn	40[a]	84/16
17	**Co-4**, **Ni foam** cathode instead of Sn	35	83/17

[a] inconsistency in the yield.

Solvent study:

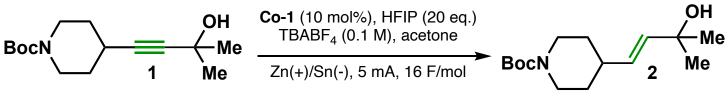

40% (84/16 E/Z)

entry	**deviation from above**	yield (%)[a]	E/Z ratio[a]
18	**Co-4**, DMSO instead of acetone	8	-
19	**Co-4**, MeCN instead of acetone	43	80/20
20	**Co-4**, DCM instead of acetone	ND	-
21	**Co-4**, DMF instead of acetone	59	78/22
22	**Co-4**, THF instead of acetone	12	68/32
23	**Co-4**, MeOH instead of acetone	24	86/16
24	**Co-4**, iso-propanol instead of acetone	68	92/8
25	**Co-4**, tert-butanol instead of acetone	50	94/6
26	**Co-4**, iso-butanol instead of acetone	67	94/6

Proton source study:

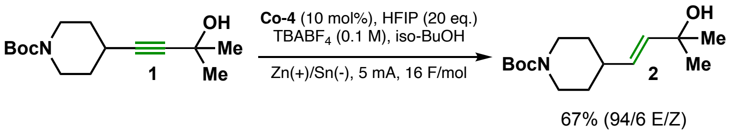

entry	**deviation from above**	yield (%)[a]	E/Z ratio[a]
27	**EtSH** instead of HFIP	9	-
28	**AcOH** instead of HFIP	43	75/25
29	**TFE** instead of HFIP	67	84/16
30	**TFA** instead of HFIP	ND	-
31	**TsOH** instead of HFIP	ND	-
32	**PhOH** instead of HFIP	28	76/24
33	**$BuNH_2$** instead of HFIP	6	-
34	**PhSH** instead of HFIP	21	71/29

Additives study:

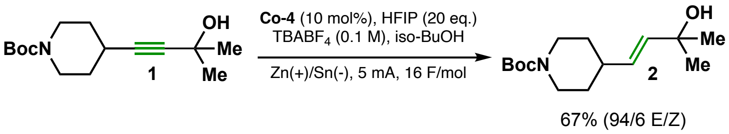

entry	**deviation from above**	yield (%)[a]	E/Z ratio[a]
35	**Co-4**, iso-butanol, H_2O (2 eq.) as an additive	88	94/6
36	**Co-4**, iso-butanol, H_2O (1 eq.) as an additive	84	94/6
37	**Co-4**, iso-butanol, H_2O (5 eq.) as an additive	88	94/6
38	**Co-4**, iso-butanol, Ph_3P (2 eq.) as an additive	72	90/10
39	**Co-4**, iso-butanol, 4-DMAP (2 eq.) as an additive	78	94/6
40	**Co-4**, iso-butanol, Et3N (2 eq.) as an additive	81	91/9

3. Computational details

Method description:

Geometries were optimized using GAUSSIAN16 REVISION C.01[18], while double-hybrid energies were calculated using ORCA versions 6.0.0 and 6.0.1.[19] Geometries were optimized using the Perdew–Burke–Ernzerhof (PBE) DFT exchange-correlation functional[20,21] with the third generation of Grimme and coworkers' empirical dispersion correction with Becke–Johnson dampening (D3BJ).[22,23] Geometry optimizations used def2-SVP basis set[24] and the def2/J (available in Gaussian as "W06") density-fitting basis set.[24,25] Accurate energies were calculated with Santra *et al.*'s revised version of Kozuch *et al.*'s DSD-PBEP86-D4 double-hybrid (DH) DFT functional,[26,27] which includes Grimme and coworkers' fourth-generation empirical dispersion correction (D4).[27,28] Energies were calculated with the def2-SVP, def2-TZVPP, and def2-QZVPP basis sets[24] and were extrapolated to the complete basis set (CBS) limit using a three-point exponential extrapolation $\left(E_{CBS} = \frac{E_n E_{n+2} - E_{n+1}^2}{E_n - 2E_{n+1} + E_{n+2}}\right)$.[29] The RIJK resolution-of-the-identity method[30] was used to speed up the calculations, in conjunction with the def2/JK[31] and def2-*x*/C (where x = SVP, TZVPP or QZVPP)[32] auxiliary basis sets. Solvation effects were modelled using Truhlar and coworkers' SMD implicit solvation model[33] with *iso*-PrOH as the solvent.

By default, thermochemical corrections to the electronic energy are calculated (and often reported) at a standard state of 298.15 K and a pressure of 1 atm; this state will be denoted as ΔG_{298}^{o}. However, this generally does not correspond to the reaction conditions (which will be denoted as $\Delta G'_{298}$). The relation between the two is:

$$\Delta G'_{298} = \Delta G_{298}^{0} + R \cdot T \cdot \ln\left(\frac{Q'}{Q^{o}}\right)$$

Where R is the universal gas constant (0.001987 kcal/mol·K = 0.082057 L·atm/mol·K) and $Q = \frac{\prod_i [product\ i]}{\prod_i [reactant\ i]}$ (where $[a]$ is the concentration of species *a*) is the reaction quotient. If we assume an ideal gas, then $pV = nRT$ or $[a] = \frac{n}{V} = \frac{p}{RT}$ or, at 298.15 K and 1 atm, $[a] = \frac{1}{24.5}$ M.[34] There is discussion in the literature as to the appropriateness for complexation-free energies of the standard assumptions typically used (*i.e.*, the ideal gas–rigid rotor–harmonic

oscillator or IGRRHO). However, Besora *et al.* demonstrated that the use of a dispersion-correction DFT functional (such as the DH used in this study) is the recommended approach.[35]

Calculations for butyne (as a model alkyne):

Energy profile calculations:

Table S1. Relative energies in the hydrogen-atom transfer (HAT) reaction of the $Co^{(III)}$–H complex with **butyne** as a model substrate.

**Reaction Step**	**ΔG_{298} (kcal/mol)**
	**revDSD-PBEP86-D4/CBS**
$Co^{(III)}$–H + **butyne**	0.0
Coordination complex	5.5
TS(HAT)	20.8
Co–Z-vinyl	-13.2

Table S2. Relative Energies of the 2-butenyl Complexes

	**Z**	**E**
**Co-1**	0.0	4.4
**Co-2**	0.0	4.4
**Co-3**	0.0	4.1
**Co-4**	0.0	5.9
**Co-5**	0.0	5.6

The BDEs in Table S seem to follow the overall expected trend that increased steric bulk decreases the BDE, as there are more unfavourable interactions between the ligand bulk and the 2-butenyl radical ligand. The unintuitive result is that the BDE for Co-1 is about the same as for H_4, but one needs to recall that the bridge is still R(H)C–C(H)R, so the steric bulk is reduced.

Table S3. Bond dissociation energies (BDE, ΔG_{298}, kcal/mol) Energies of the 2-butenyl Complexes.

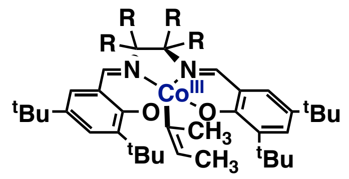

general strcuture of Co-vinyl intermediate

Co catalyst	calculated BDE (kcal/mol)
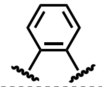	30.9
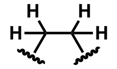	30.3
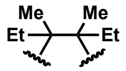	28.8
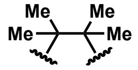	27.9

Reaction of the Free Radical with Alcohols

The final step of the reaction may involve the reaction of the formed *E*-butenyl radical with either the ^{t}BuOH solvent or the hexafluoro-*iso*-propanol (HFIP) added as an acid source for the cobalt catalyst. (*E*-2-butene is more stable than the *Z*-analogue by ΔG_{298} = 0.9 kcal/mol.) The reaction with HFIP is unfavorable with ΔG_{298} = 0.9 kcal/mol, but is (slightly) favorable with *iso*-PrOH by ΔG_{298} = -3.8 kcal/mol (free energies are corrected for concentration).

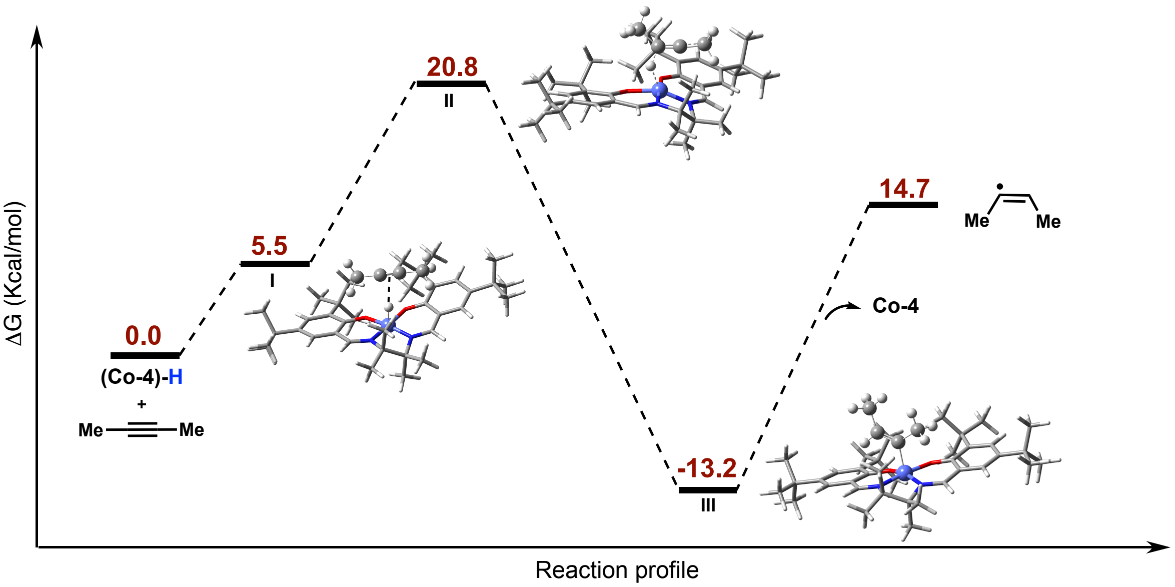

Calculations for alkyne 49:

Chemical structure of **49**:

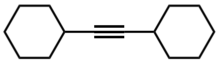

Energy profile calculations:

Table 4. Relative energies in the hydrogen-atom transfer (HAT) reaction of the $Co^{(III)}$–H complex with 1,2-dicyclohexylethyne **49**.

**Reaction Step**	**ΔG298 (kcal/mol)**		
	**PBE$_{D3BJ}$/def2-SVP**	**revDSD-PBEP86-D4/QZVPP**	**revDSD-PBEP86-D4/CBS**
$Co^{(III)}$–H + **49**	0.0	0.0	0.0
Coordination complex	-8.1	3.6	3.8
TS(HAT)	9.9	16.3	16.7
Co–Z-vinyl	-31.9	-11.3	-11.0

Bond Dissociation Energy of various cobalt catalysts with alkyne **49**:

Table 5. Bond dissociation energies (BDE, ΔG_{298}, kcal/mol) Energies of the 2-butenyl Complexes.

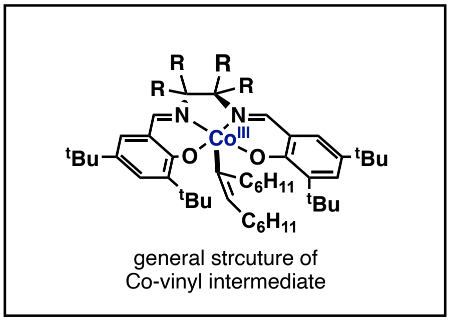

general strcuture of Co-vinyl intermediate

Co catalyst	calculated BDE (kcal/mol)
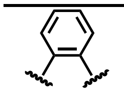	29.7
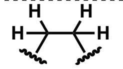	28.4
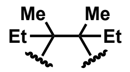	25.4
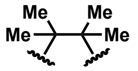	24.6

Table 6. Hydrogen-atom abstraction energies (kcal/mol) under standard state $\left(\boldsymbol{\Delta G_{298}^{0}}\right)$ and reaction conditions $\left(\boldsymbol{\Delta G_{298}^{exp}}\right)$ of 1,2-dicyclohexylvinyl radical with various hydrogen donors.

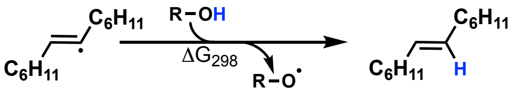

	$\boldsymbol{\Delta G_{298}^{exp}}$
HFIP	0.8
iso-PrOH	-4.0
Co–H	-50.3

Calculations for the other three unsymmetrical alkynes:

Chemical structures of alkynes:

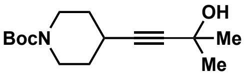

49

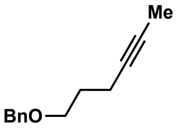

S18

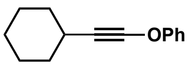

S5

Table 7. Bond dissociation energies (BDE, ΔG_{298}, kcal/mol), energy of the pre-activation complex $\left(\boldsymbol{\Delta G_{298}^{pre}}\right)$, relative energy of the Z-vinyl complex $\left(\boldsymbol{\Delta G_{298}^{(Z)}}\right)$, and HAT barrier height ($\boldsymbol{\Delta G_{298}^{\ddagger}}$, kcal/mol) at the SMD(*iso*-PrOH)-revDSD-PBEP86-D4/CBS//PBE_{D3BJ}/def2-SVP level of theory of various vinyl substrates from the Co-4 complex, where the vinyl radical maintains the parent *E* isomer and radical centre.

	$\boldsymbol{\Delta G_{298}^{pre}}$	$\boldsymbol{\Delta G_{298}^{\ddagger}}$	$\boldsymbol{\Delta G_{298}^{(Z)}}$	**E**
Boc-PP-Ċ=CH-C(Me)$_2$OH (alkyne 1)	8.3	13.2	-3.2	5.8
OH(Me)$_2$C-Ċ=CH-PP-Boc (alkyne 1)	11.0	26.3	-3.5	4.7
BnOCH$_2$CH$_2$CH$_2$-Ċ=CH-Me (alkyne S18)	6.8	20.5	-8.4	21.1
Me-Ċ=CH- CH$_2$CH$_2$CH$_2$OBn (alkyne S18)	6.3	21.2	-7.6	18.2
PhO-Ċ=CH-Cy (alkyne S5)	1.3	29.3[b]	-24.0	22.1
Cy-Ċ=CH-OPh (alkyne S5)	5.3	7.3	-12.5	17.6

[a] For the oxazolidinonyl complexes, the lower BDE of two rotamers is given. [b] Only one of the two transition states – one for each rotamer – was found.

Table 8. Bond dissociation energies (BDE, ΔG_{298}, kcal/mol), energy of the pre-activation complex $\left(\mathbf{\Delta G_{298}^{\mathit{pre}}}\right)$, relative energy of the Z-vinyl complex $\left(\mathbf{\Delta G_{298}^{(Z)}}\right)$, and HAT barrier height ($\mathbf{\Delta G_{298}^{\ddagger}}$, kcal/mol) at the SMD(*iso*-PrOH)-revDSD-PBEP86-D4/def-QZVPP//PBE_{D3BJ}/def2-SVP level of theory of various vinyl substrates from the Co-4 complex, where the vinyl radical maintains the parent *E* isomer and radical centre.

	$\mathbf{\Delta G_{298}^{\mathit{pre}}}$	$\mathbf{\Delta G_{298}^{\ddagger}}$	$\mathbf{\Delta G_{298}^{(Z)}}$	**E**
BocNCy-Ċ=CH-CMe_2OH (alkyne 1)	8.0	13.2	-3.2	6.0
CMe_2OH-Ċ=CH-BocNCy (alkyne 1)	10.6	25.7	-3.9	4.8
$BnOCH_2CH_2CH_2$-Ċ=CH-Me (alkyne S18)	6.6	20.3	-8.7	21.0
Me-Ċ=CH- $CH_2CH_2CH_2OBn$ (alkyne S18)	6.0	20.8	-7.6	18.2
PhO-Ċ=CH-Cy (alkyne S5)	0.9	28.7	-24.3	22.2
Cy-Ċ=CH-OPh (alkyne S5)	5.0	6.8	-12.9	17.8

[a] For the oxazolidinonyl complexes, the lower BDE of two rotamers is given.

Table 9. Bond dissociation energies (BDE, ΔG_{298}, kcal/mol), energy of the pre-activation complex $\left(\mathbf{\Delta G_{298}^{\mathit{pre}}}\right)$, relative energy of the Z-vinyl complex $\left(\mathbf{\Delta G_{298}^{(Z)}}\right)$, and HAT barrier height ($\mathbf{\Delta G_{298}^{\ddagger}}$, kcal/mol) at the PBE_{D3BJ}/def2-SVP level of theory of various vinyl substrates from the Co-4 complex, where the vinyl radical maintains the parent *E* isomer and radical centre.

	$\mathbf{\Delta G_{298}^{\mathit{pre}}}$	$\mathbf{\Delta G_{298}^{\ddagger}}$	$\mathbf{\Delta G_{298}^{(Z)}}$	**E**
BocNCy-Ċ=CH-CMe_2OH (alkyne 1)	-0.9	8.0	-27.1	21.4
CMe_2OH-Ċ=CH-BocNCy (alkyne 1)	-5.8	11.0	-27.4	23.3
$BnOCH_2CH_2CH_2$-Ċ=CH-Me (alkyne S18)	-5.3	18.2	-29.4	29.8
Me-Ċ=CH- $CH_2CH_2CH_2OBn$ (alkyne S18)	-6.0	15.4	-27.1	30.1
PhO-Ċ=CH-Cy (alkyne S5)	-13.0	0.9	-42.5	34.3
Cy-Ċ=CH-OPh (alkyne S5)	-11.5	9.1	-38.9	29.2[b]

[a] For the oxazolidinonyl complexes, the lower BDE of two rotamers is given. [b] The relative energies of the rotamers are flipped at this level of theory; the BDE of the other rotamer is 34.2 kcal/mol.

4. NMR spectra

^{1}H-NMR (300 MHz, $CDCl_3$) of compound **1**

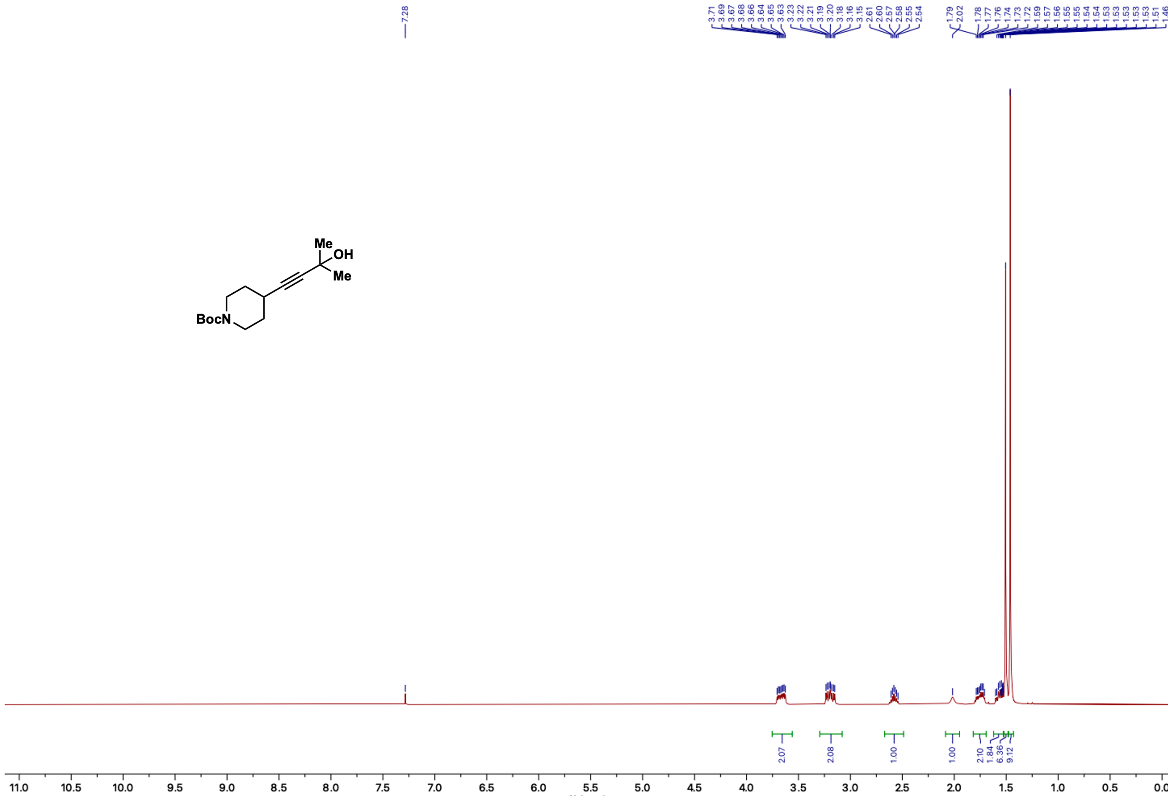

^{13}C-NMR (75 MHz, $CDCl_3$) of compound **1**

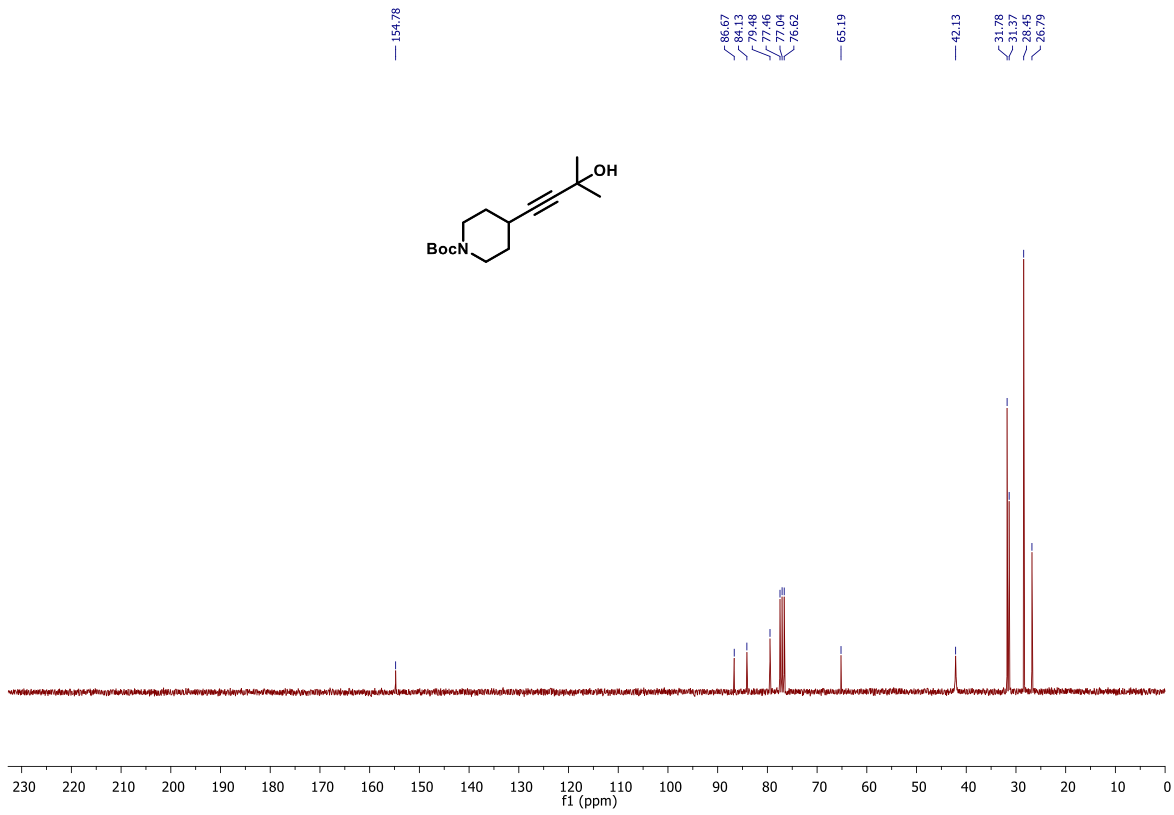

^{1}H-NMR (400 MHz, $CDCl_3$) of compound **51**

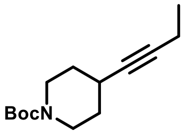

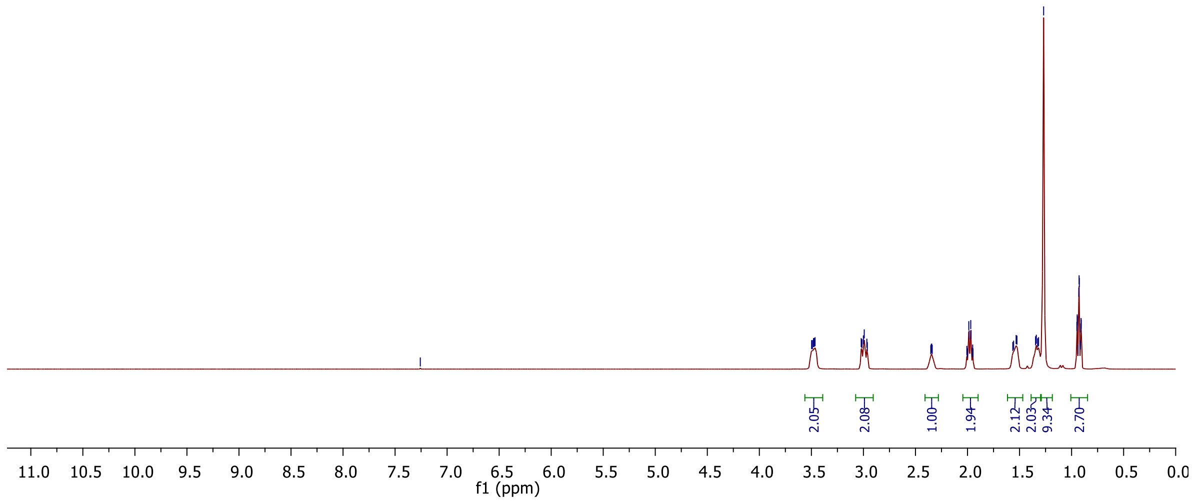

^{13}C-NMR (101 MHz, $CDCl_3$) of compound **51**

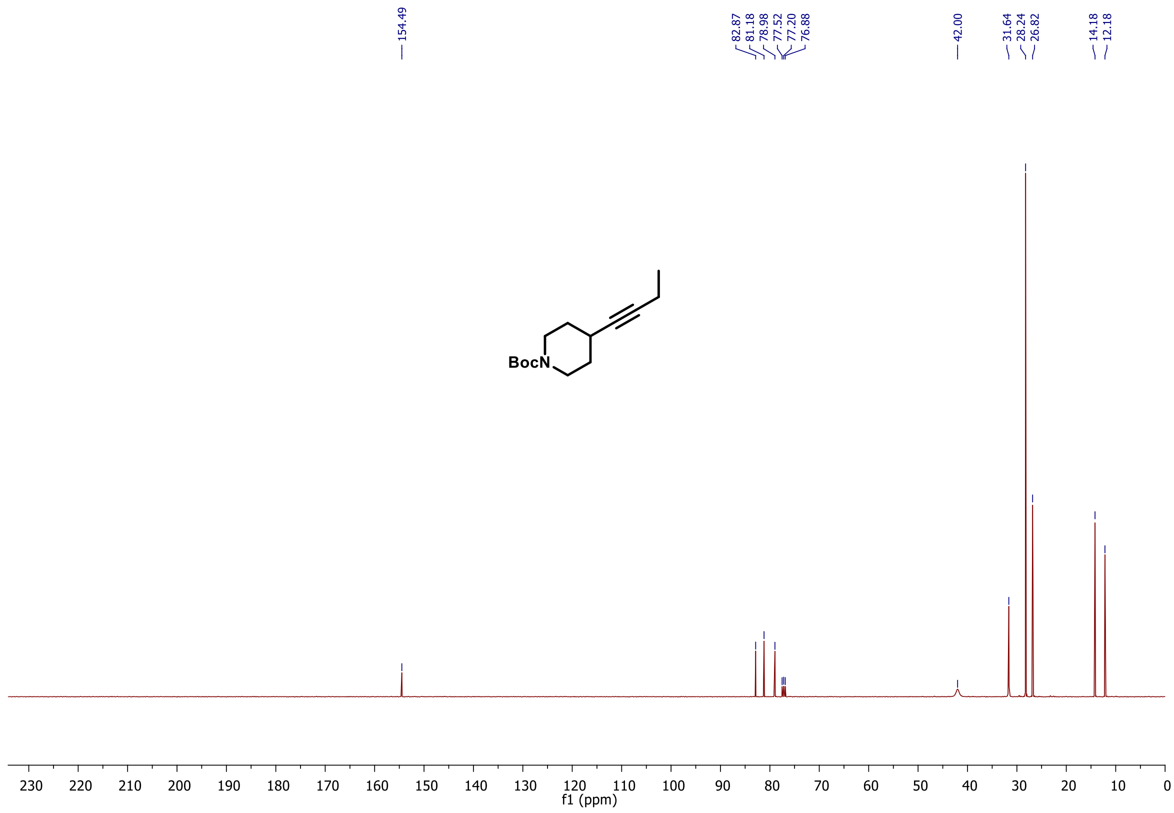

^{1}H-NMR (300 MHz, $CDCl_3$) of compound **S1**

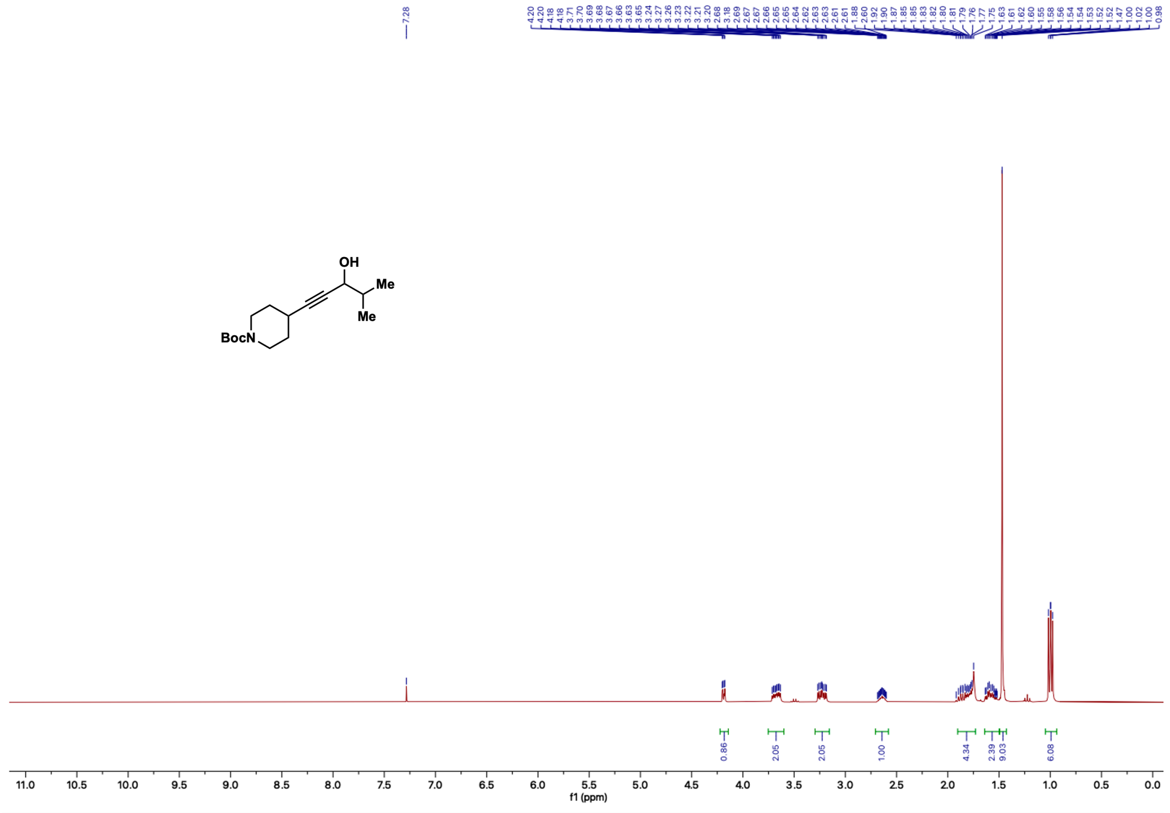

^{13}C-NMR (75 MHz, $CDCl_3$) of compound **S1**

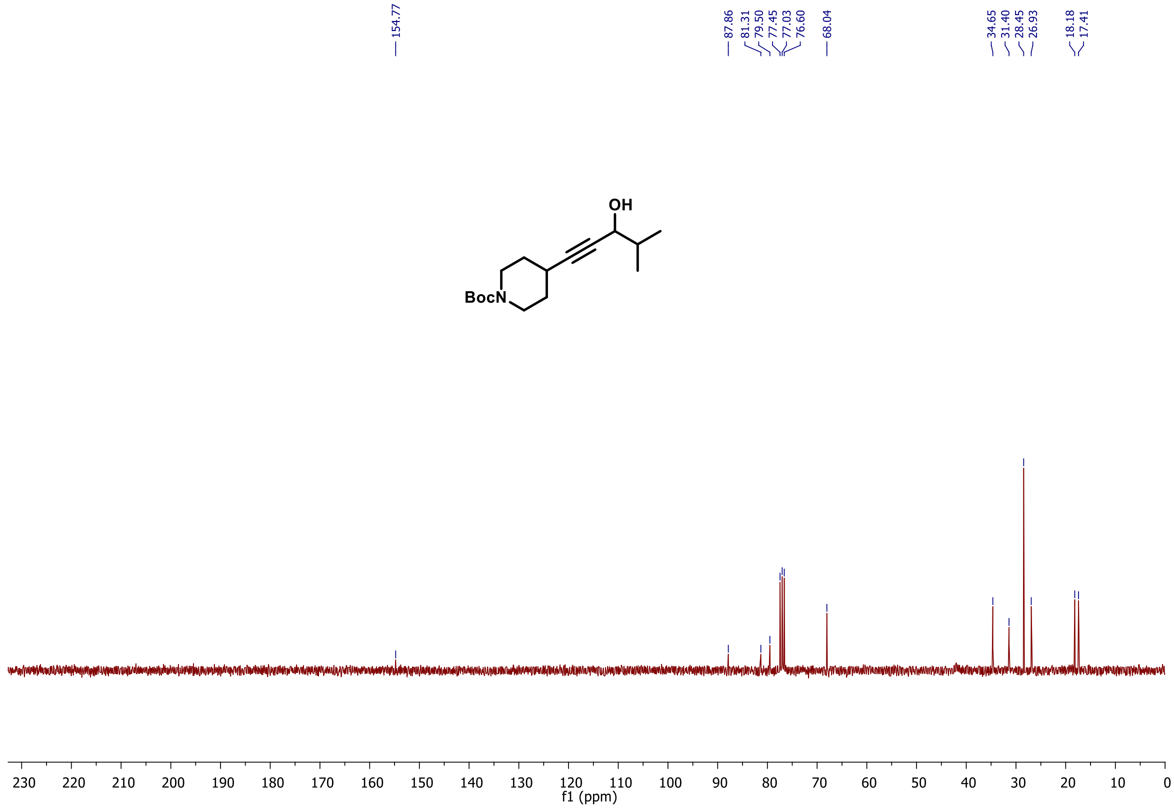

^{1}H-NMR (300 MHz, $CDCl_3$) of compound **S2**

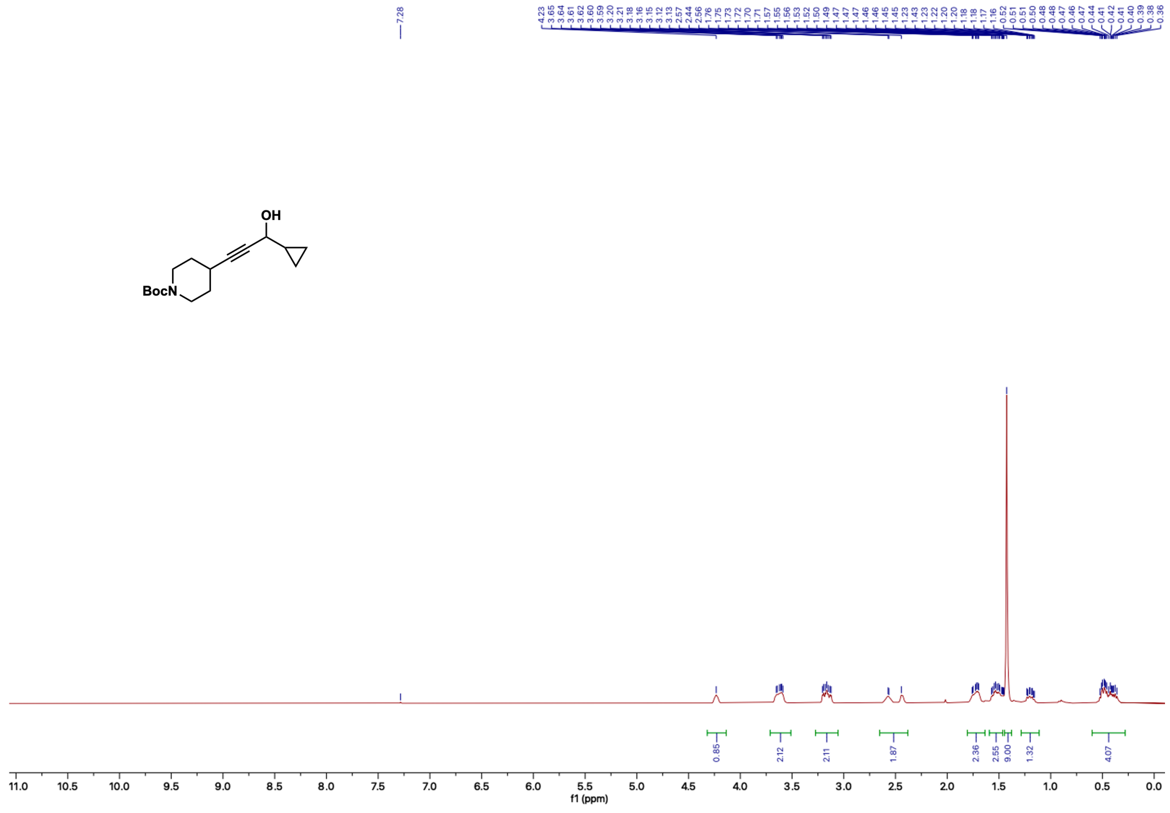

^{13}C-NMR (75 MHz, $CDCl_3$) of compound **S2**

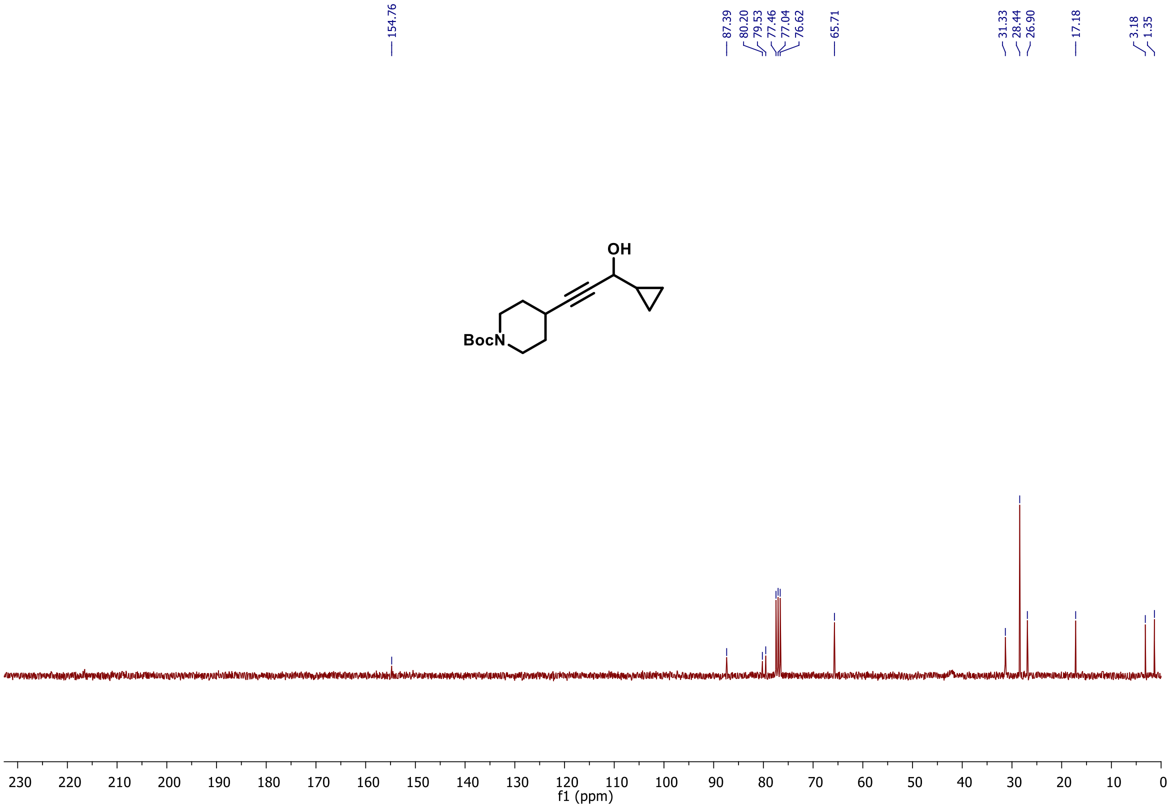

^{1}H-NMR (400 MHz, $CDCl_3$) of compound **S3**

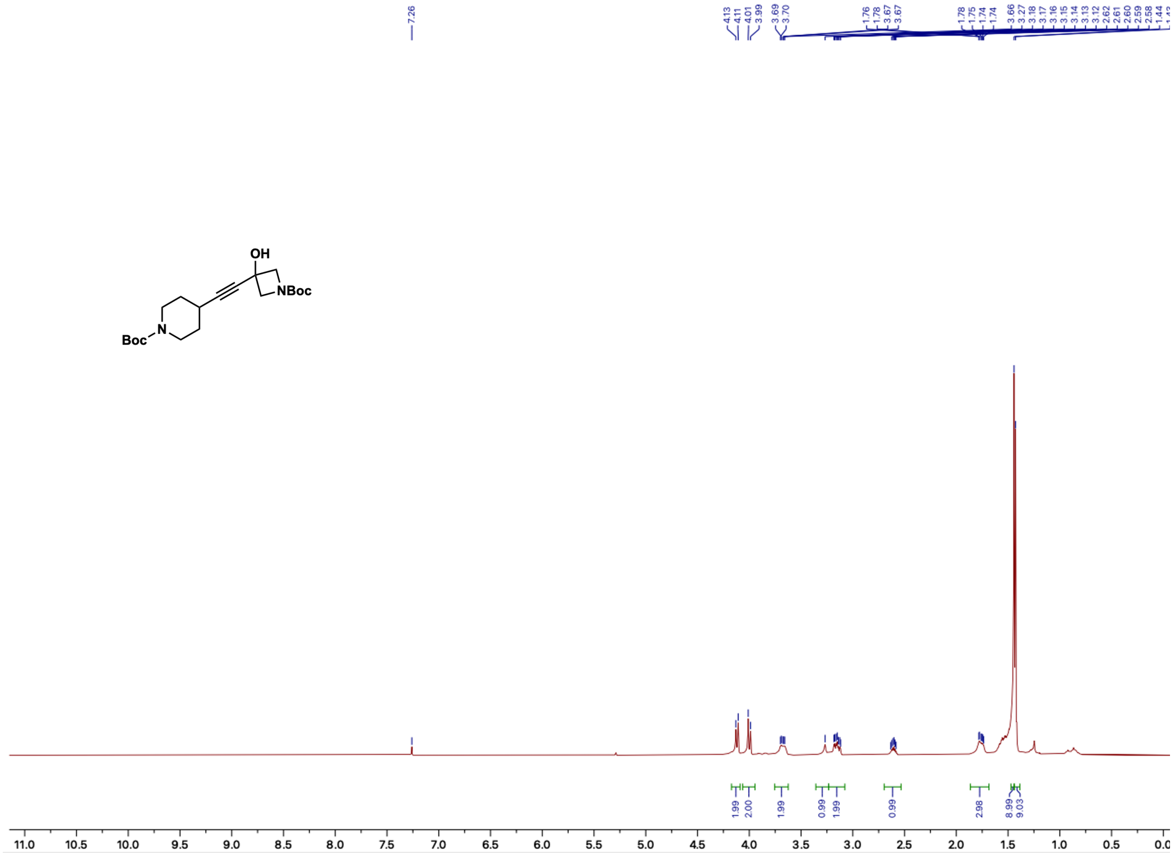

^{13}C-NMR (101 MHz, $CDCl_3$) of compound **S3**

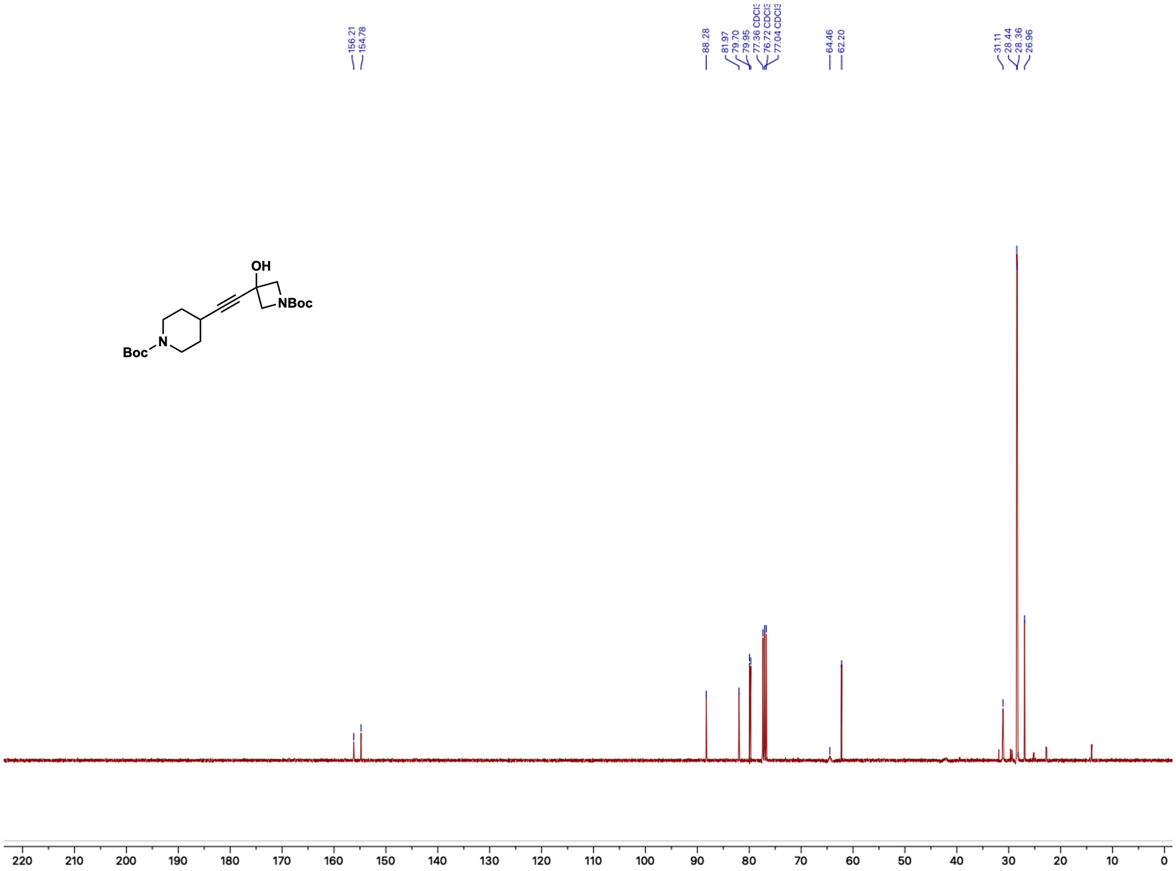

^{1}H-NMR (300 MHz, $CDCl_3$) of compound **S4**

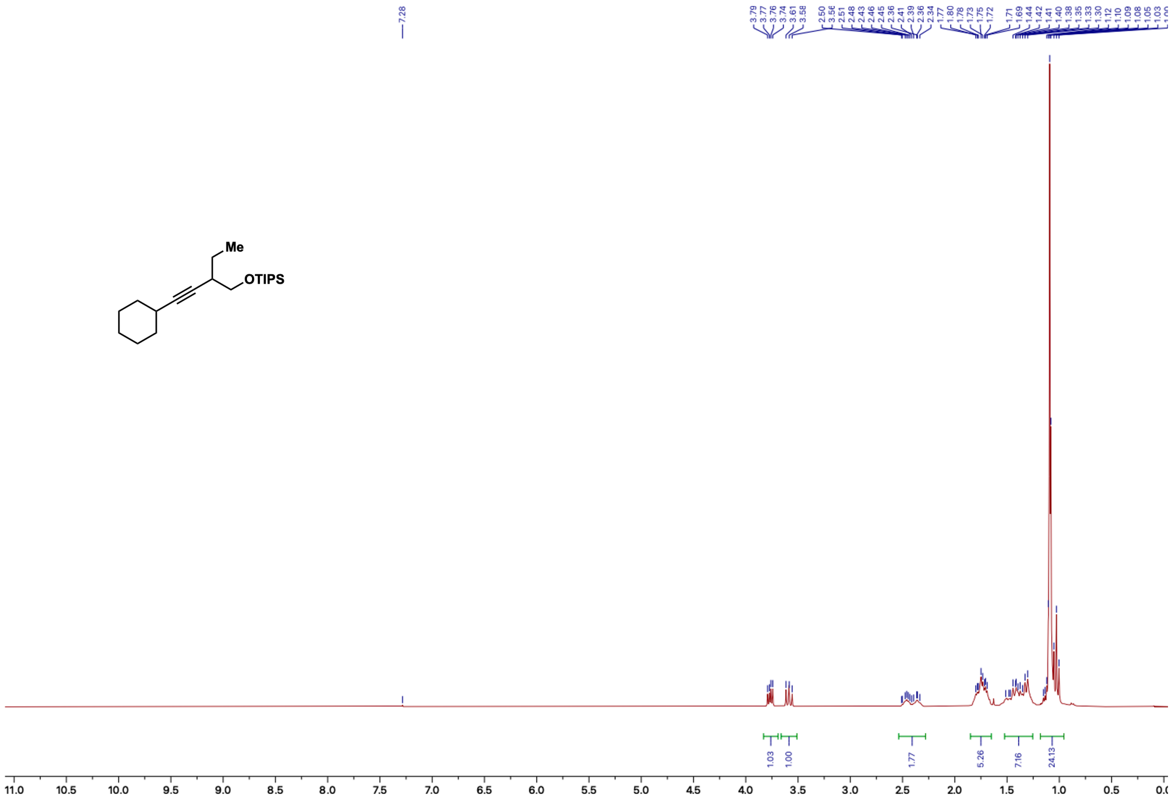

^{13}C-NMR (75 MHz, $CDCl_3$) of compound **S4**

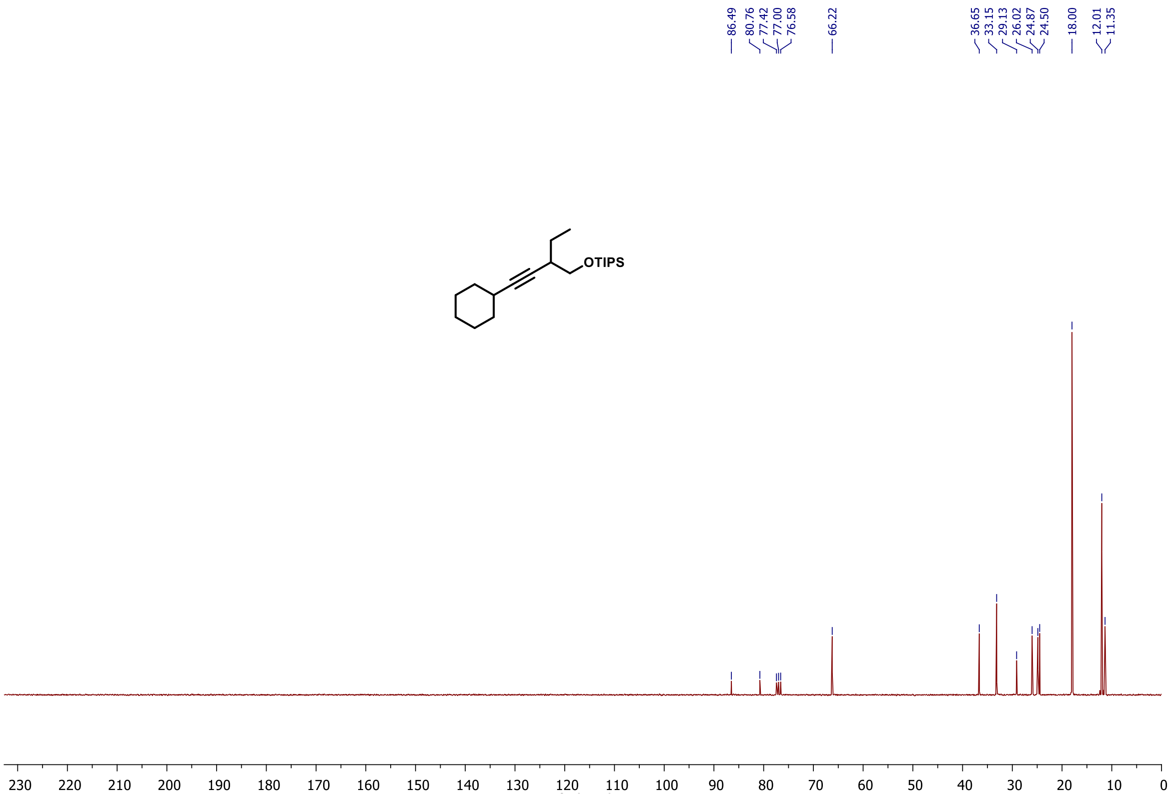

^{1}H-NMR (300 MHz, $CDCl_3$) of compound **S5**

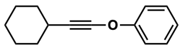

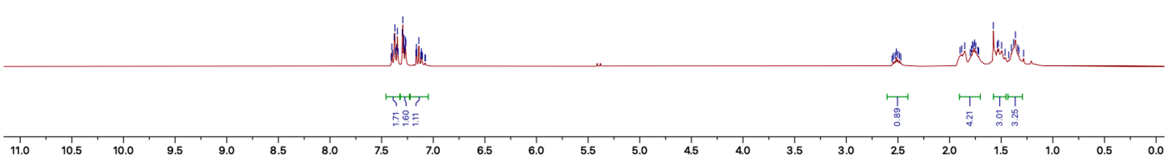

^{13}C-NMR (101 MHz, $CDCl_3$) of compound **S5**

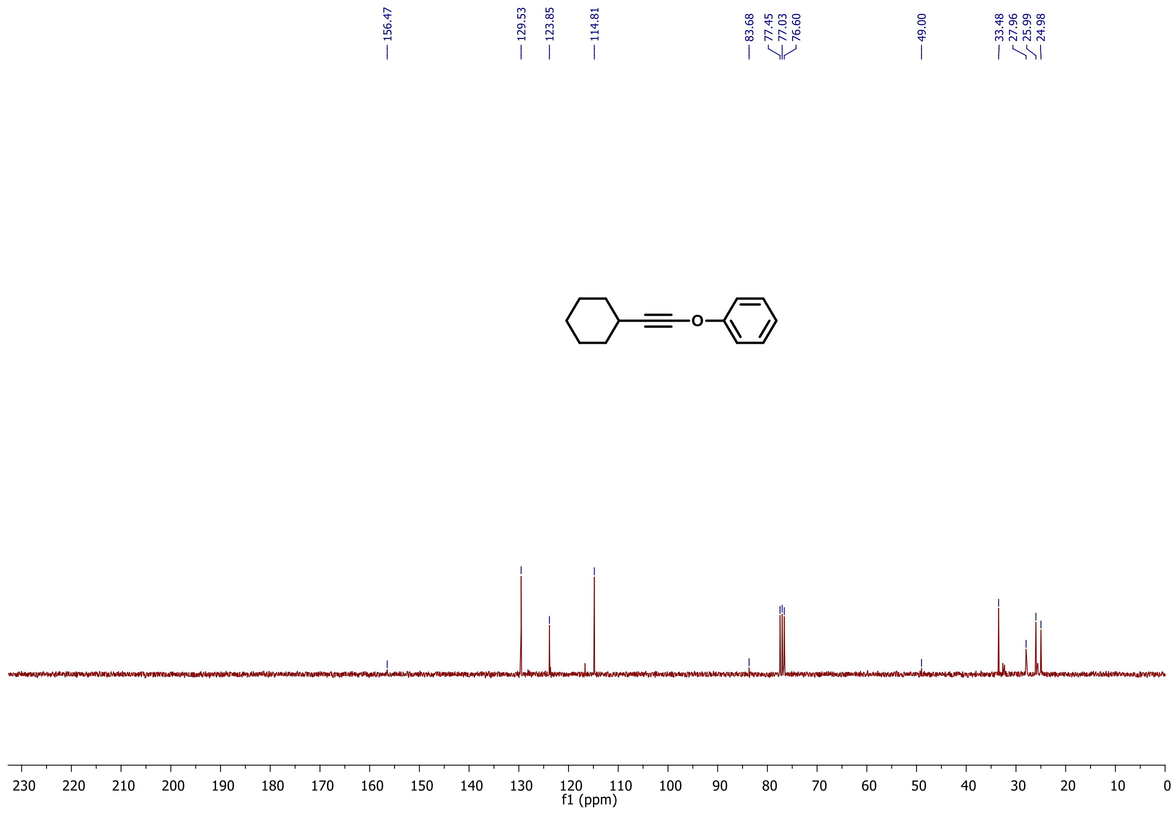

^{1}H-NMR (300 MHz, $CDCl_3$) of compound **S6**

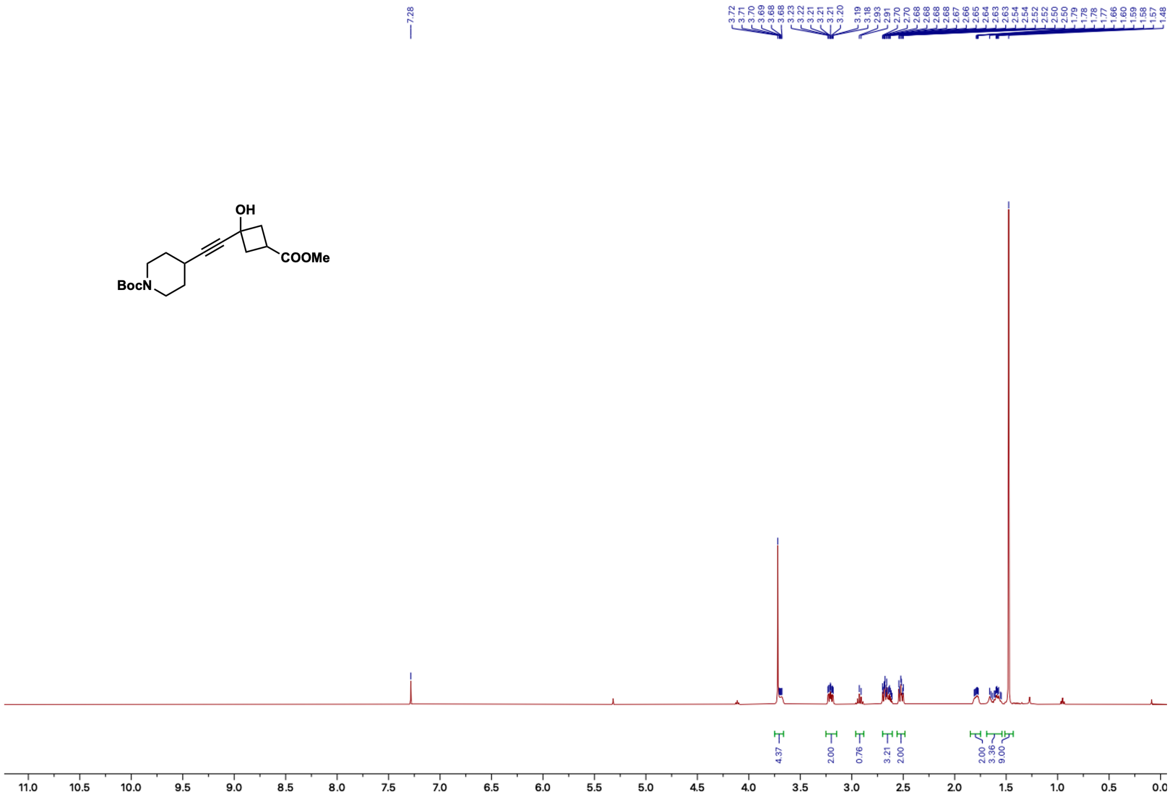

^{13}C-NMR (101 MHz, $CDCl_3$) of compound **S6**

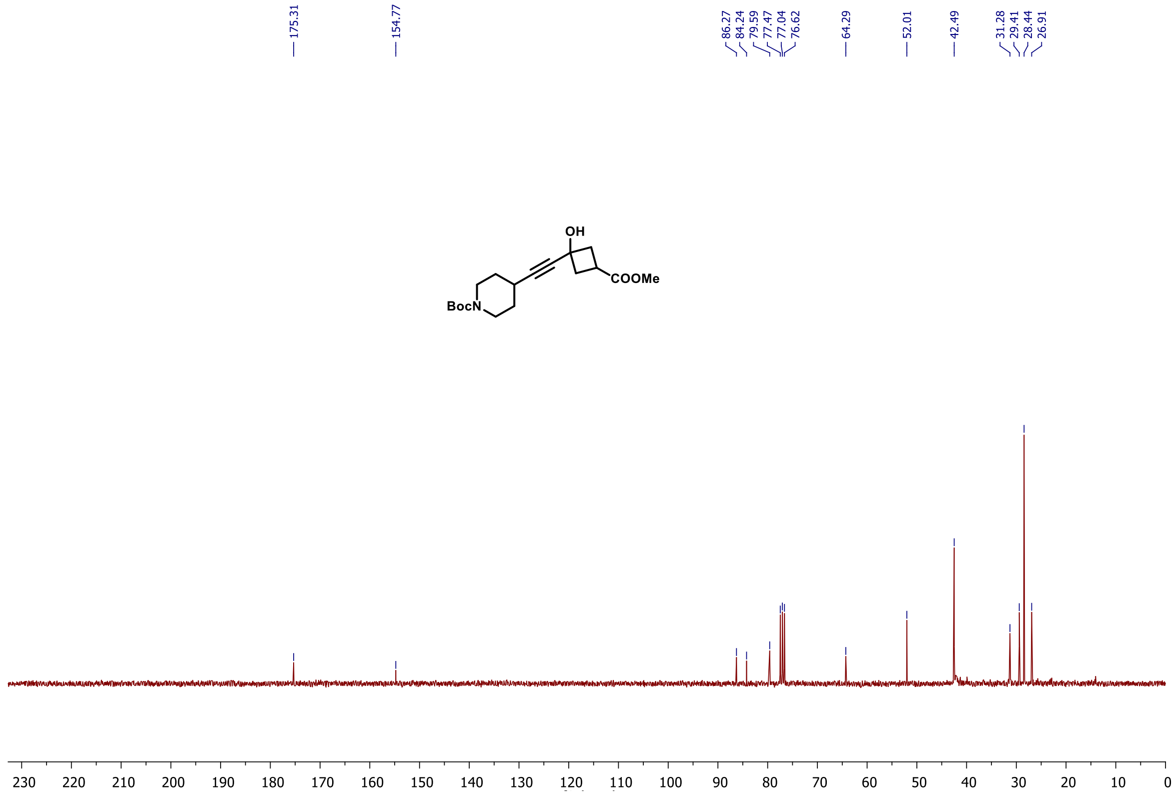

^{1}H-NMR (300 MHz, $CDCl_3$) of compound **S7**

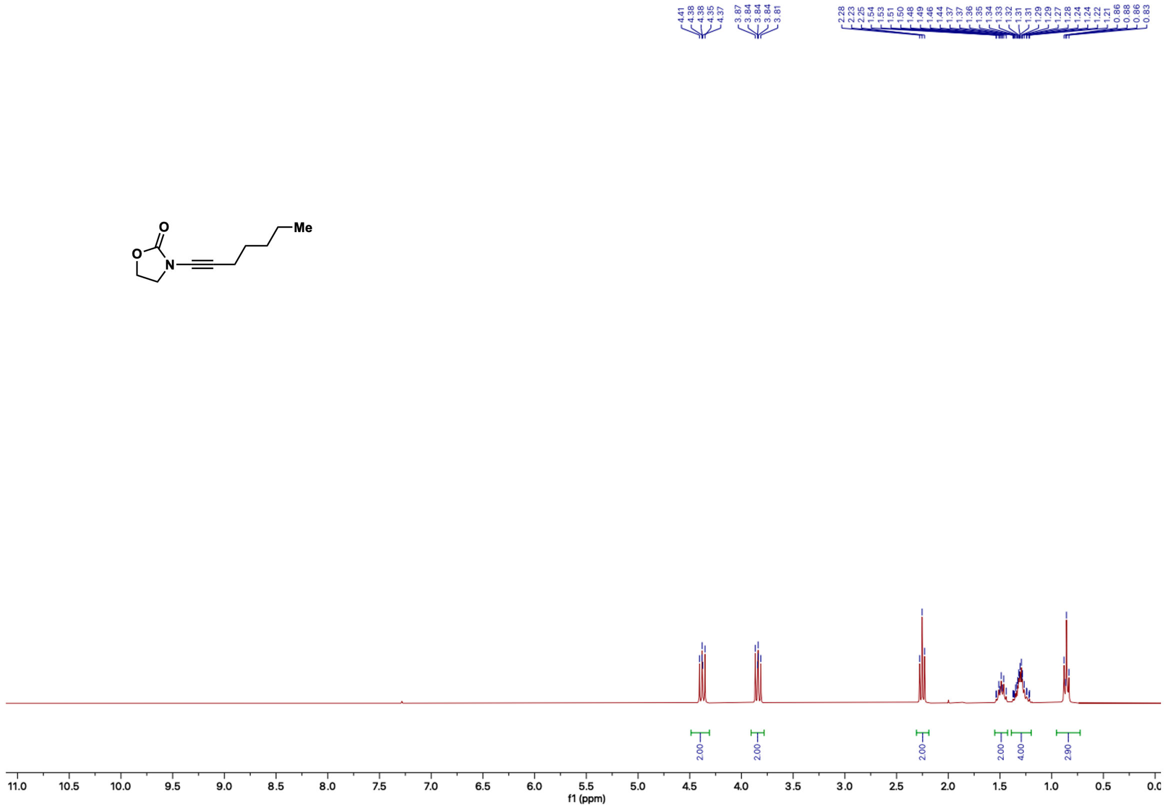

^{13}C-NMR (101 MHz, $CDCl_3$) of compound **S7**

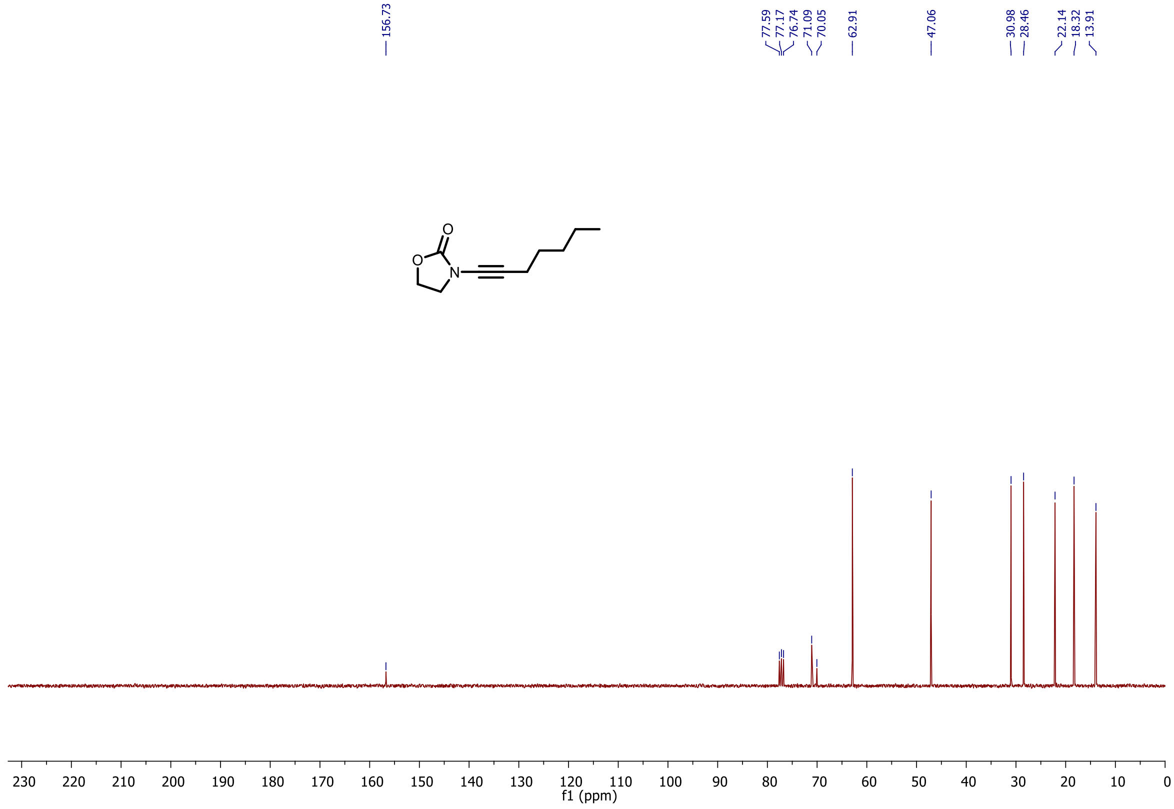

^{1}H-NMR (300 MHz, $CDCl_3$) of compound **S8**

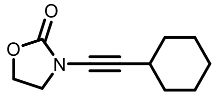

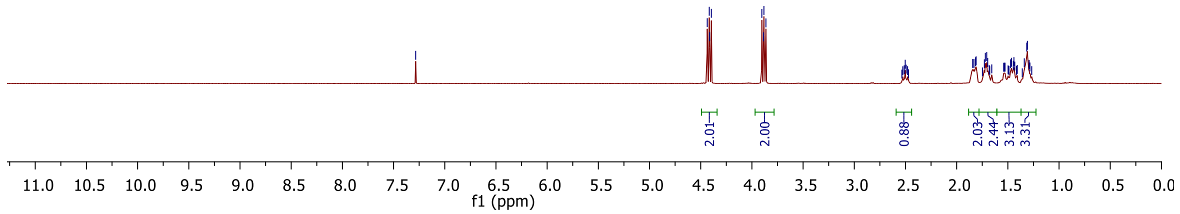

^{13}C-NMR (101 MHz, CDCl$_3$) of compound **S8**

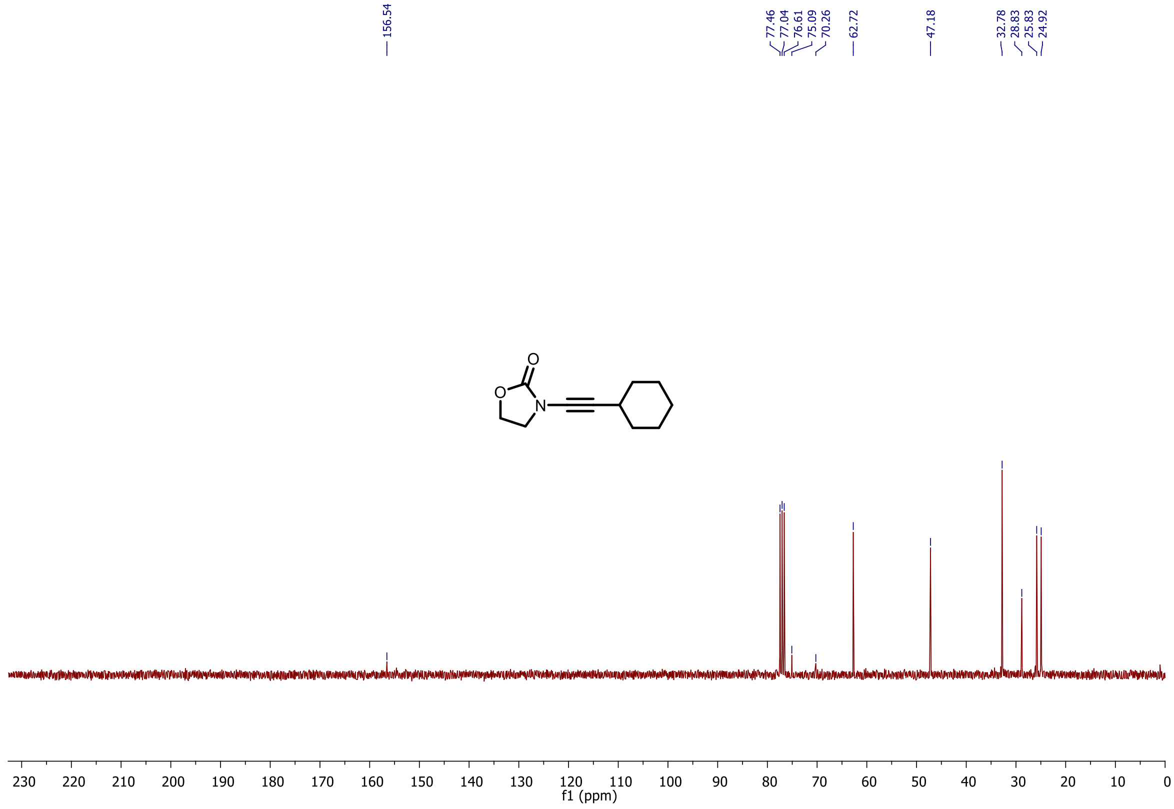

^{1}H-NMR (300 MHz, $CDCl_3$) of compound **S9**

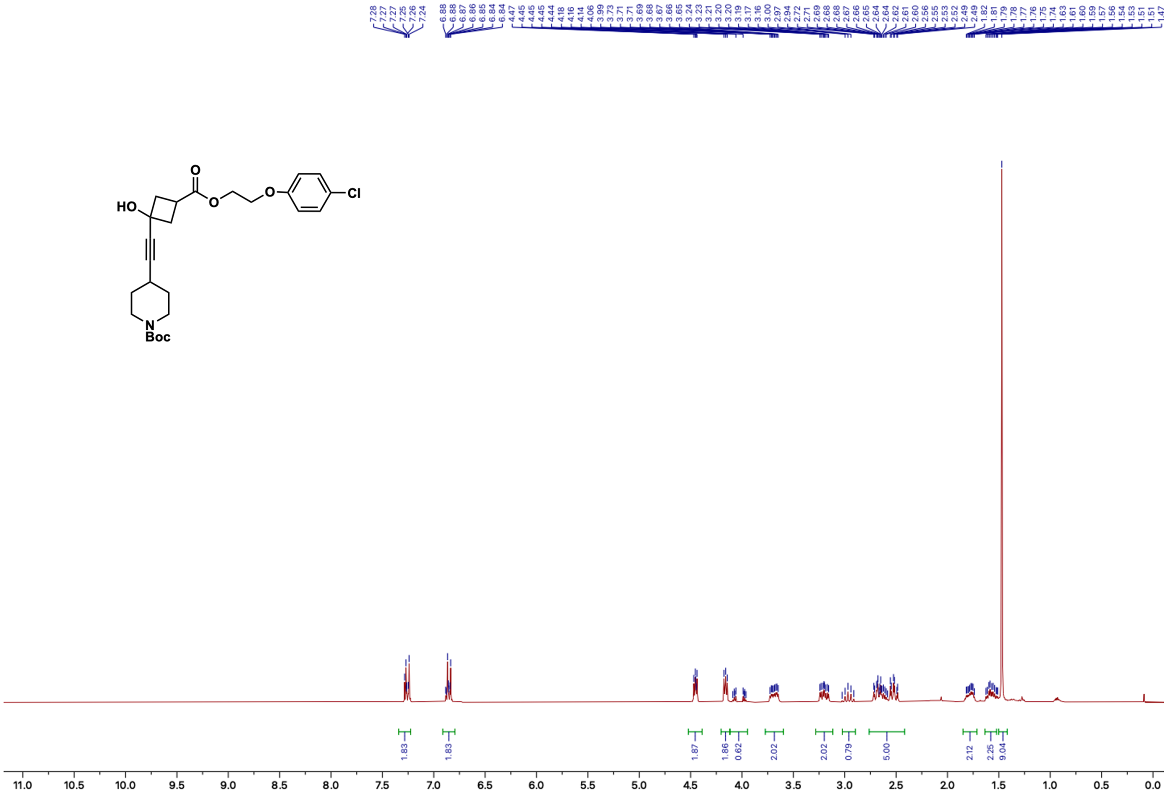

^{13}C-NMR (101 MHz, $CDCl_3$) of compound **S9**

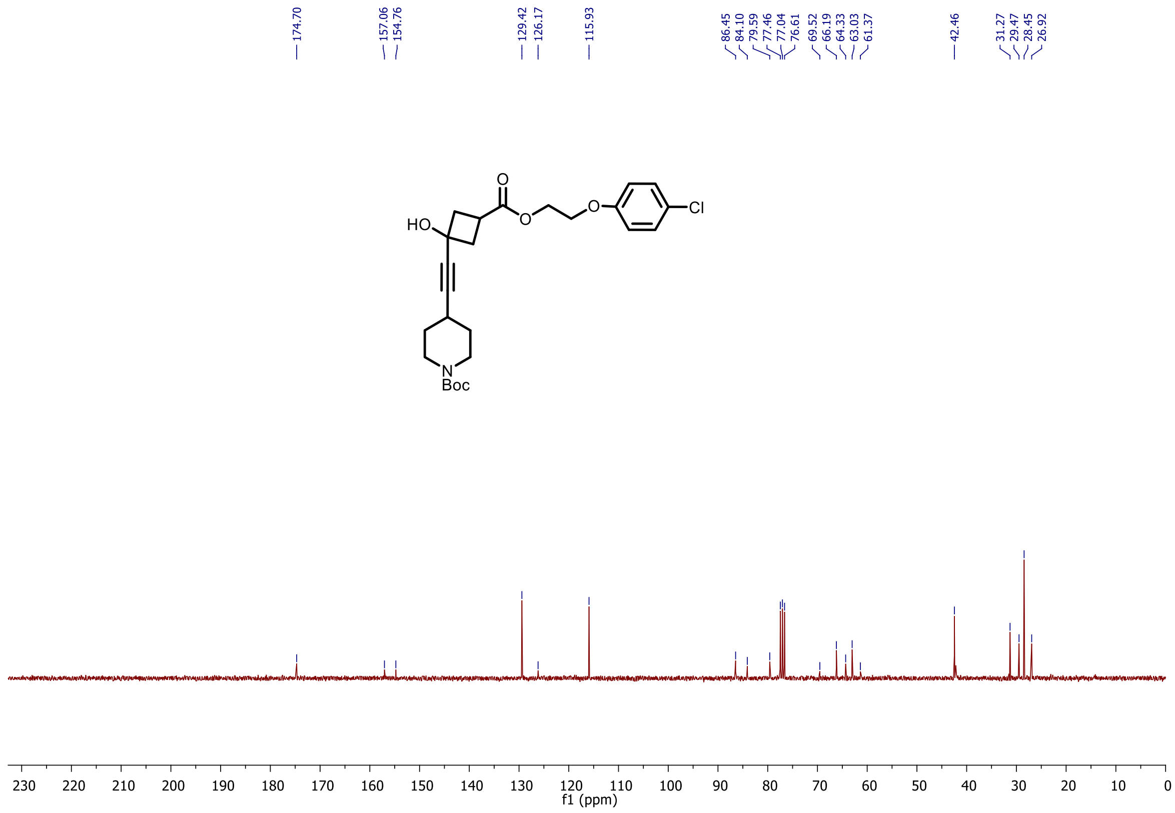

^{1}H-NMR (300 MHz, $CDCl_3$) of compound **S10**

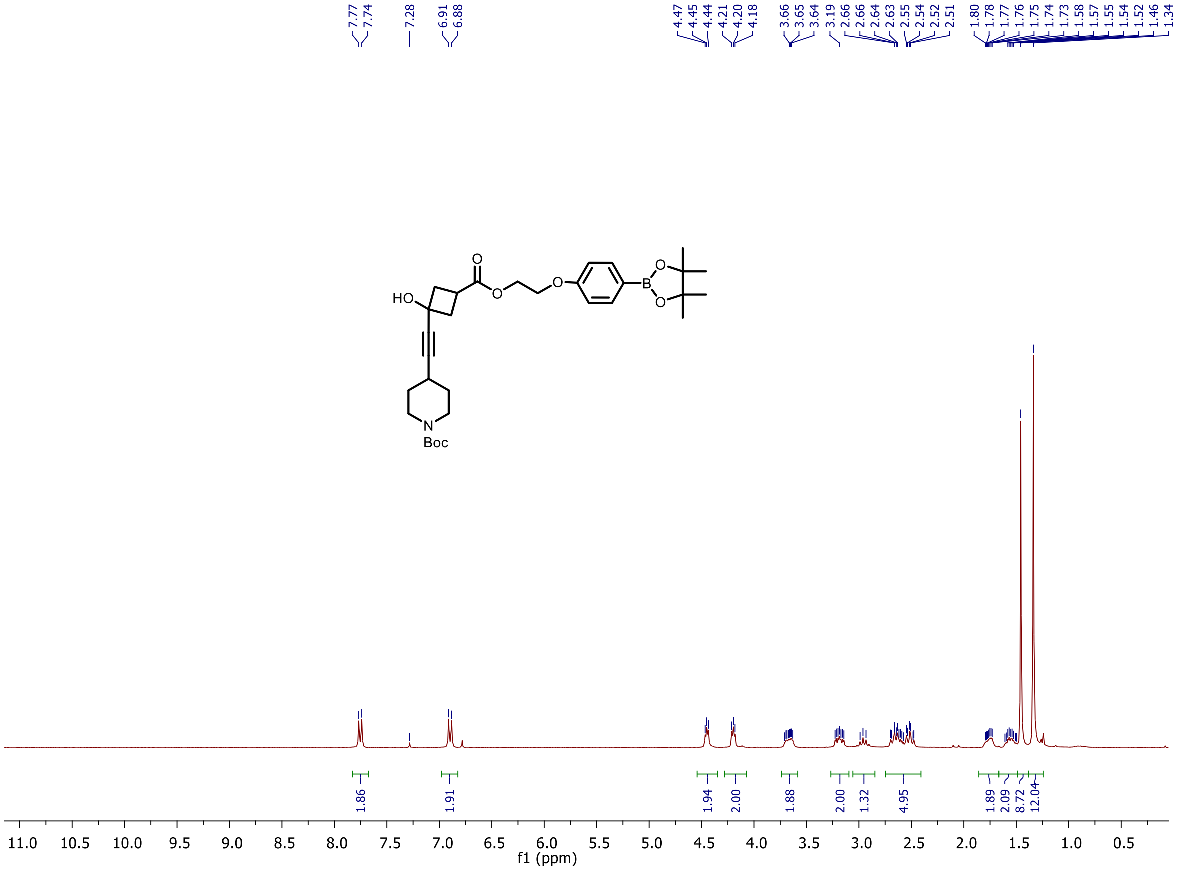

^{13}C-NMR (101 MHz, $CDCl_3$) of compound **S10**

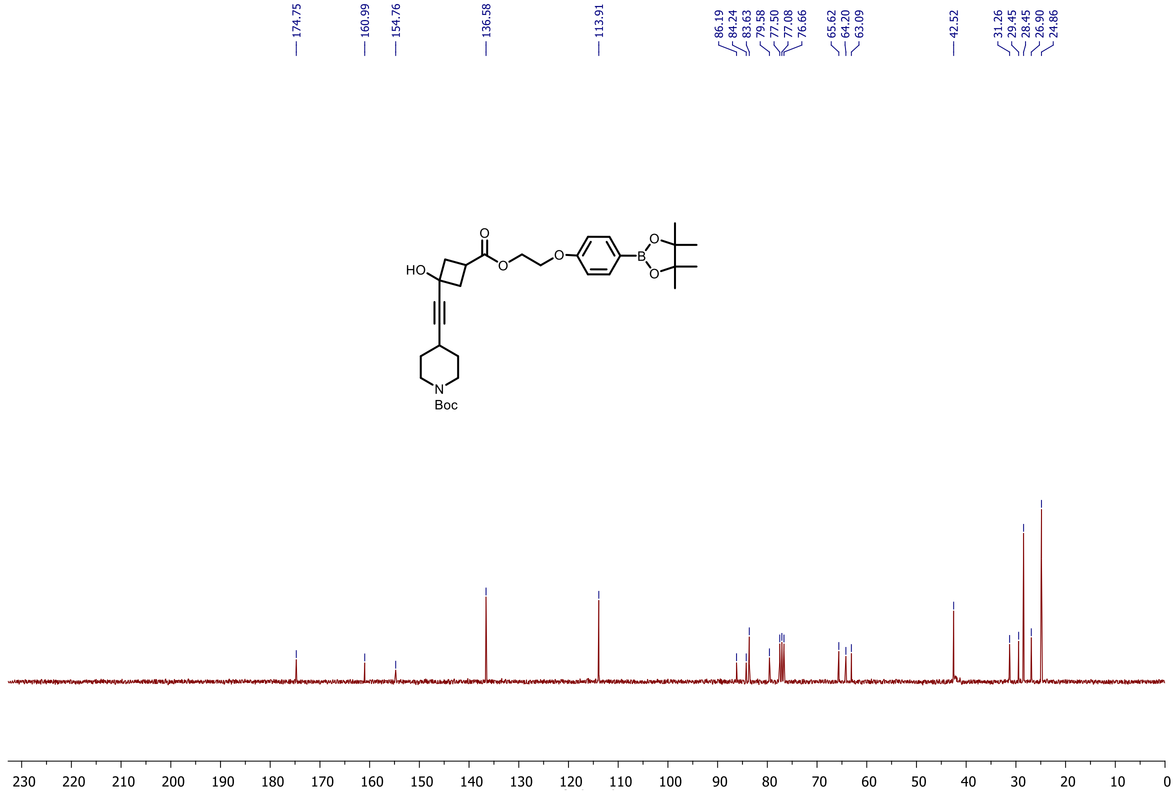

^{11}B NMR (96 MHz, $CDCl_3$) of compound **S10**

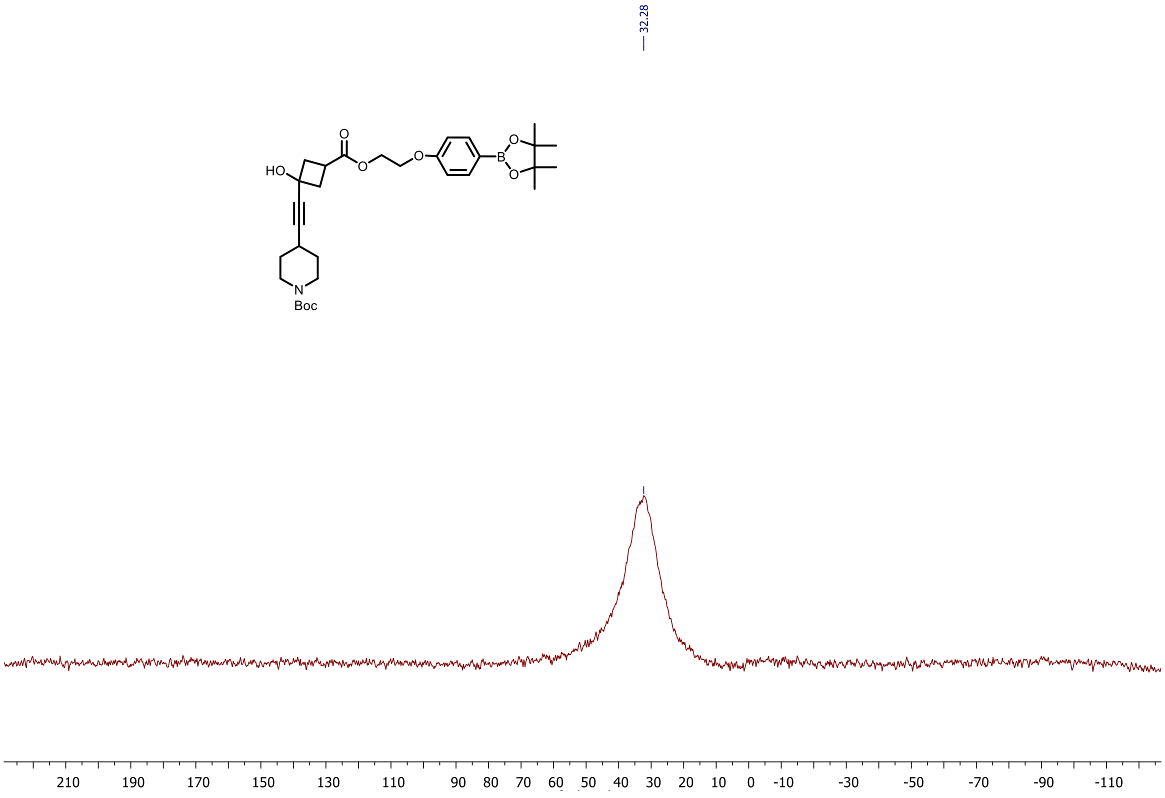

^{1}H-NMR (300 MHz, $CDCl_3$) of compound **S11**

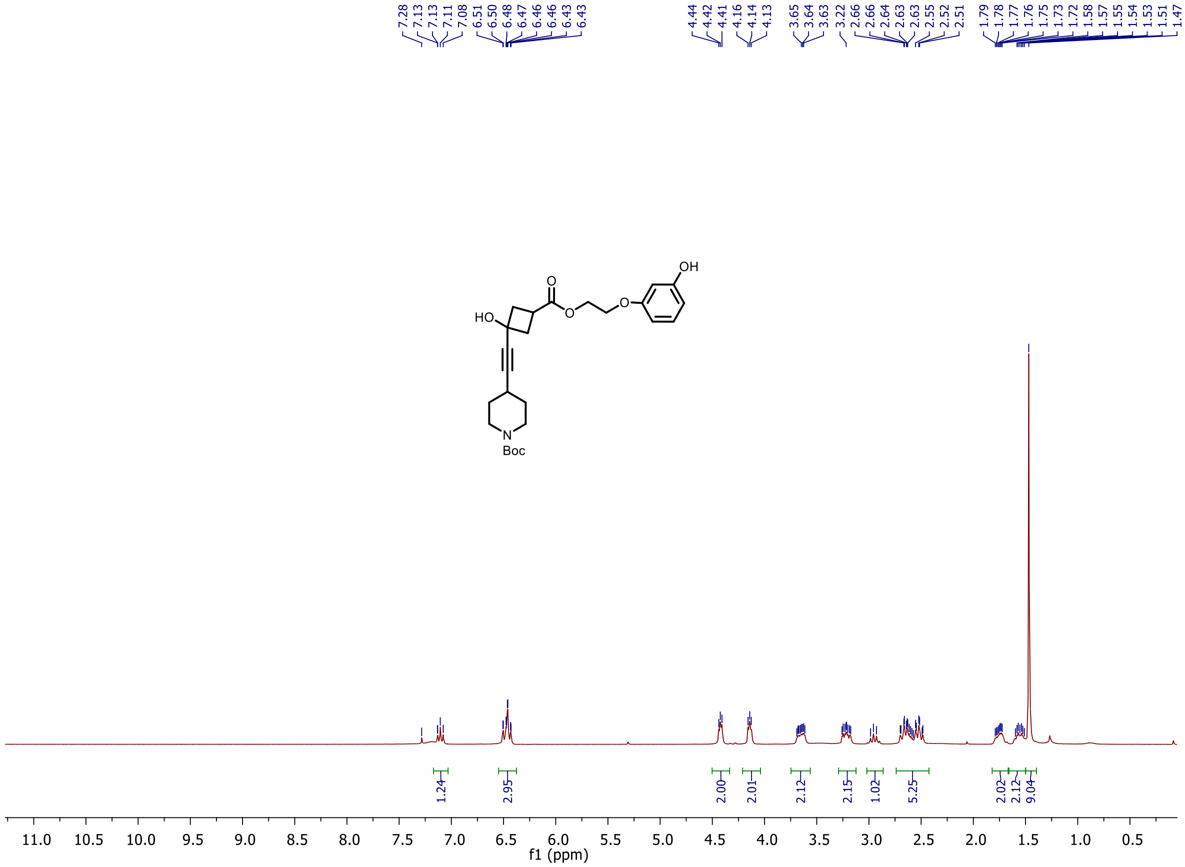

^{13}C-NMR (101 MHz, $CDCl_3$) of compound **S11**

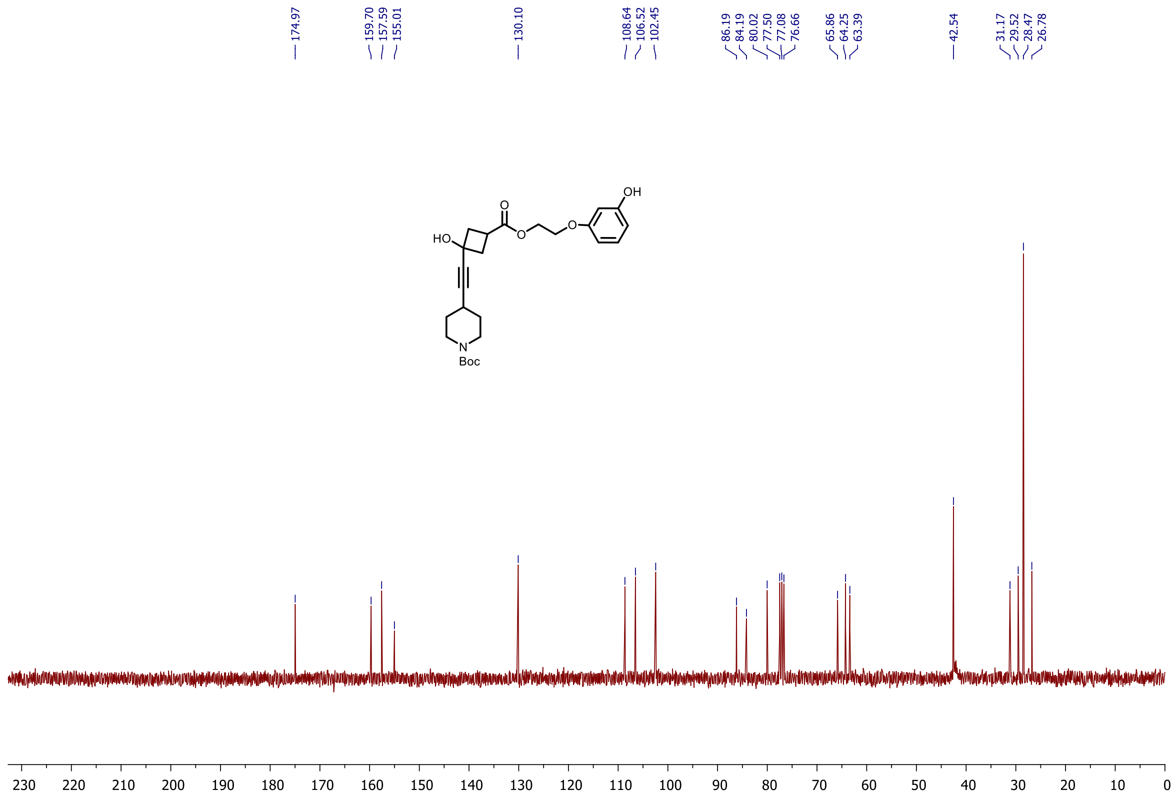

^{1}H-NMR (300 MHz, $CDCl_3$) of compound **S12**

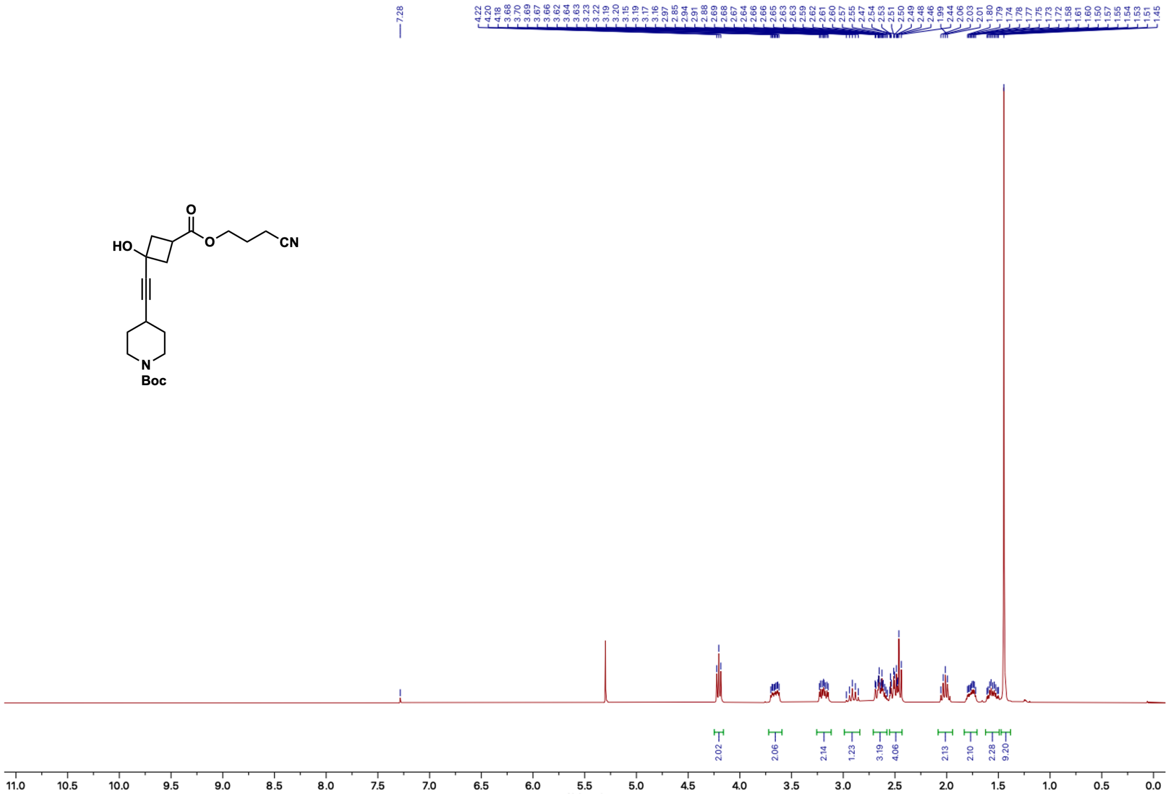

^{13}C-NMR (75 MHz, $CDCl_3$) of compound **S12**

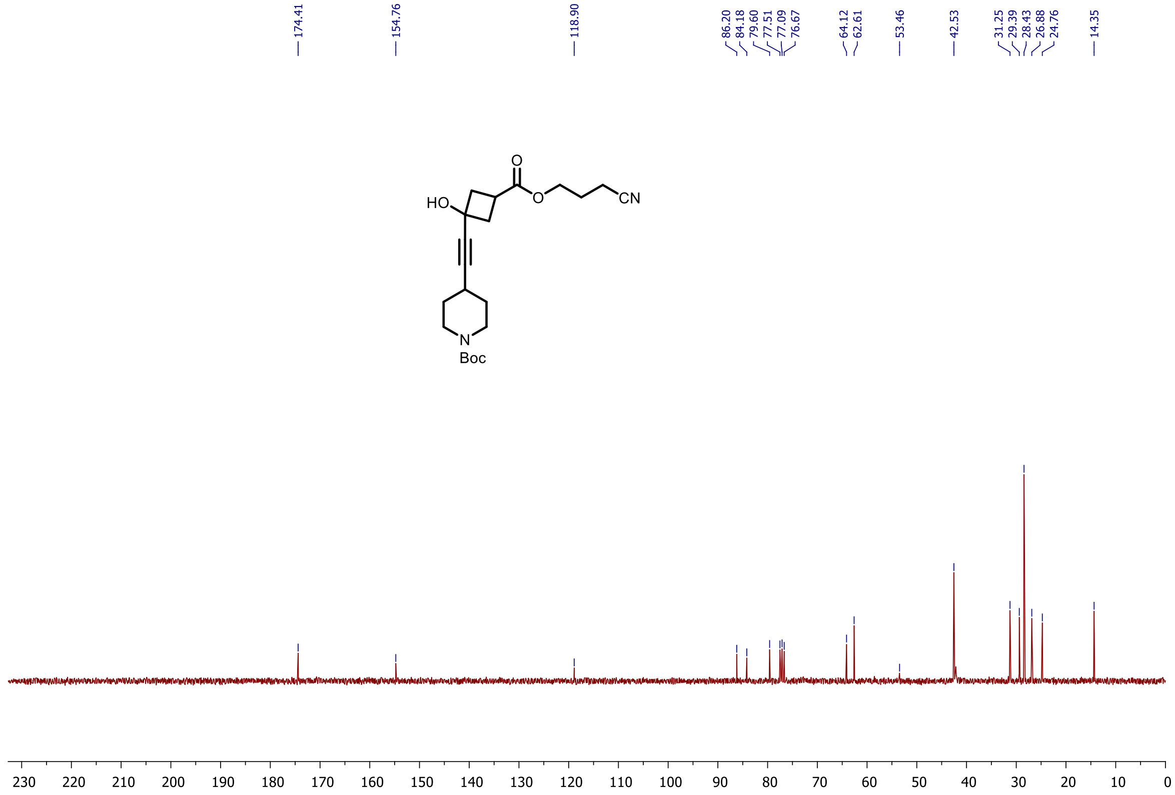

^{1}H-NMR (300 MHz, $CDCl_3$) of compound **S13**

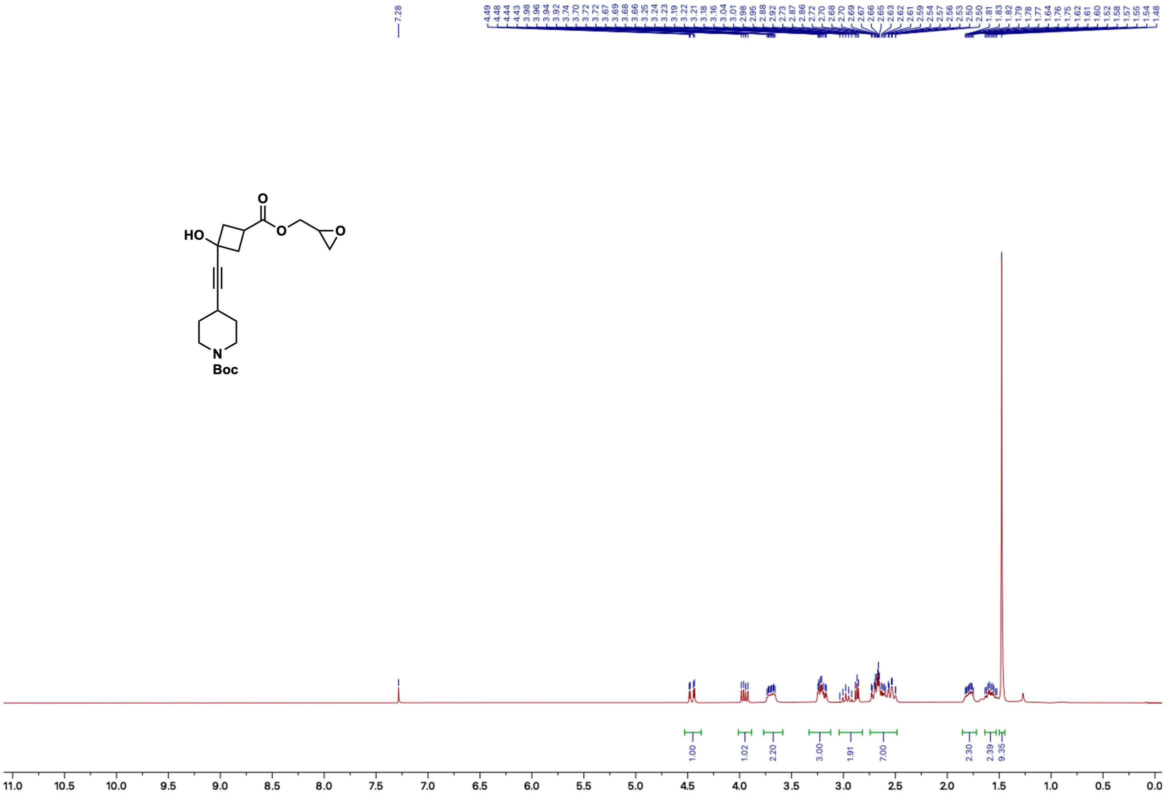

^{13}C-NMR (101 MHz, $CDCl_3$) of compound **S13**

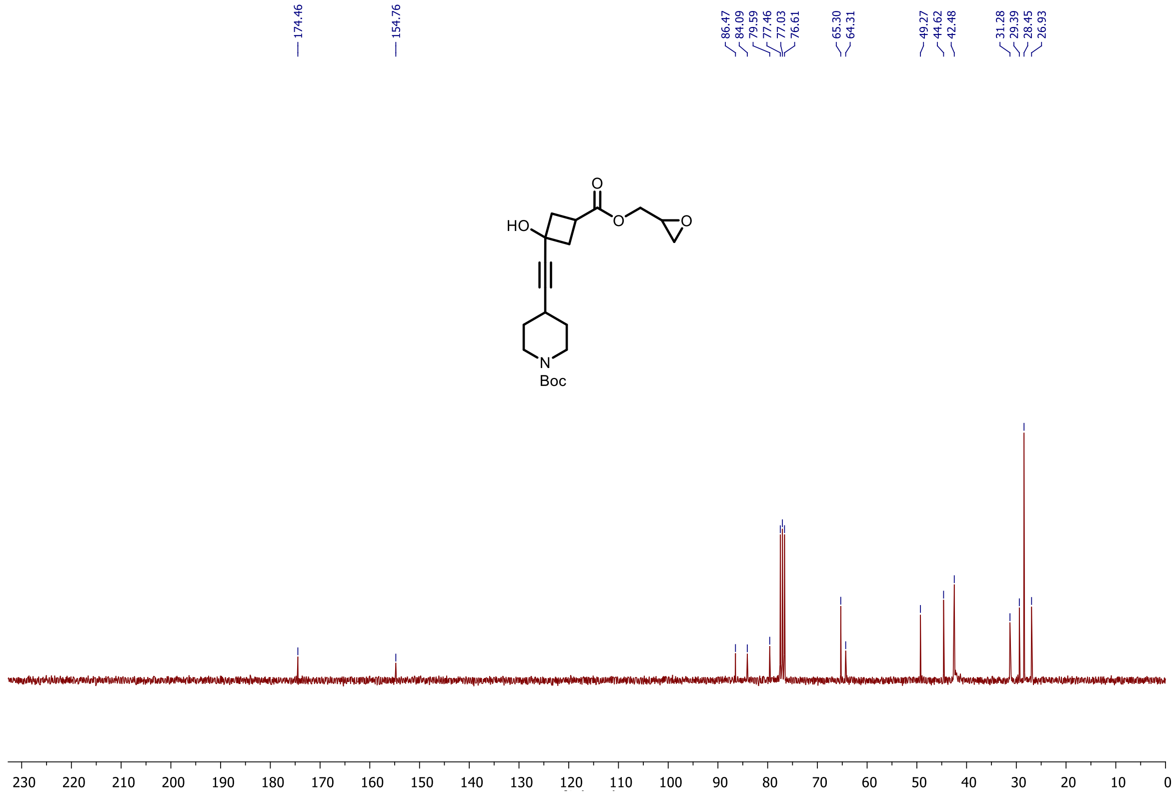

^{1}H-NMR (300 MHz, $CDCl_3$) of compound **S14**

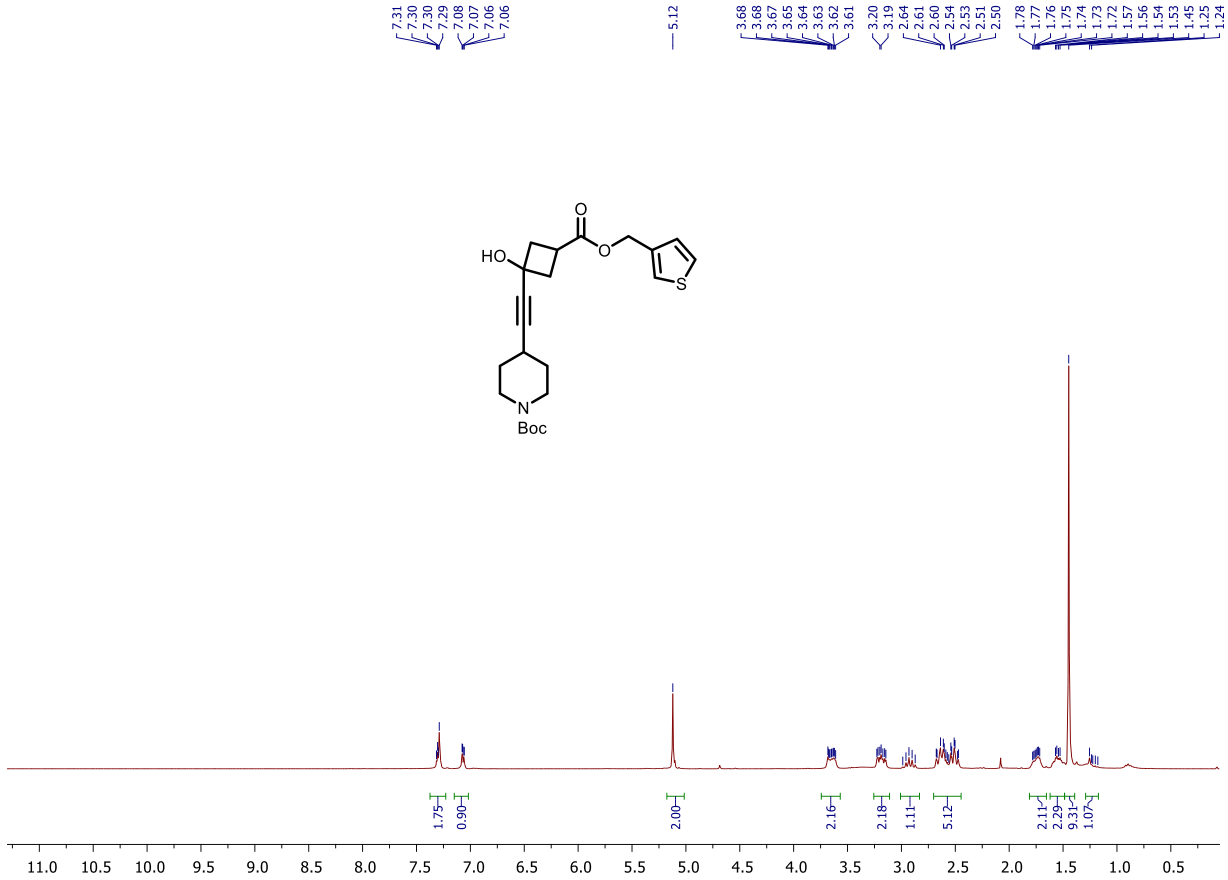

^{13}C-NMR (101 MHz, $CDCl_3$) of compound **S14**

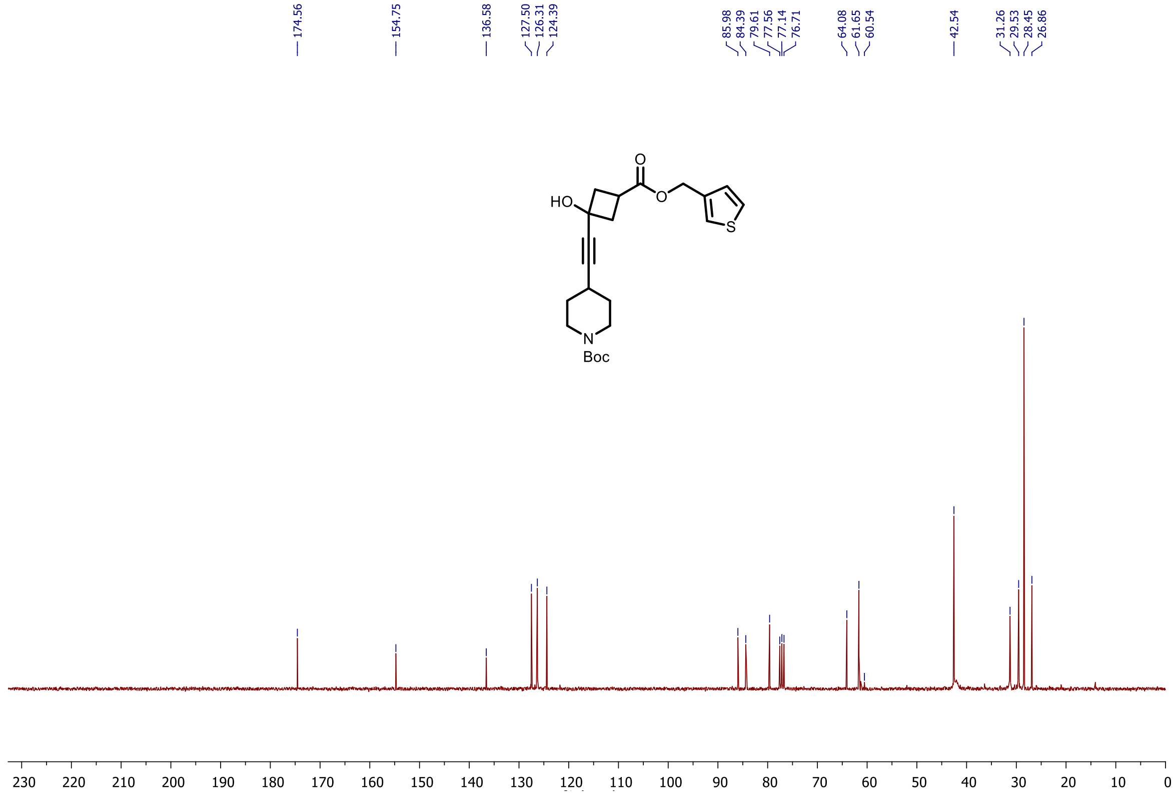

^{1}H-NMR (300 MHz, $CDCl_3$) of compound **S15**

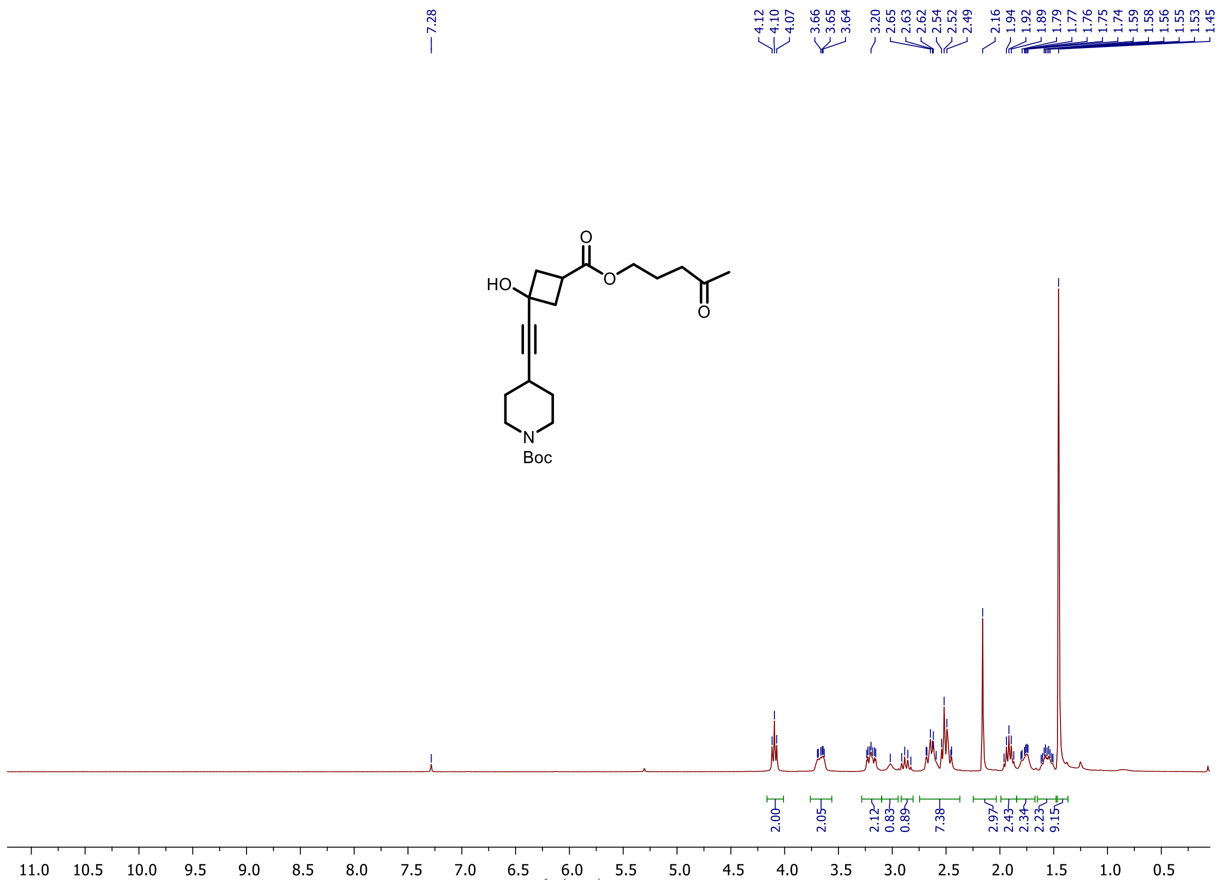

^{13}C-NMR (101 MHz, CDCl3) of compound **S15**

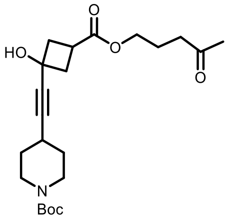

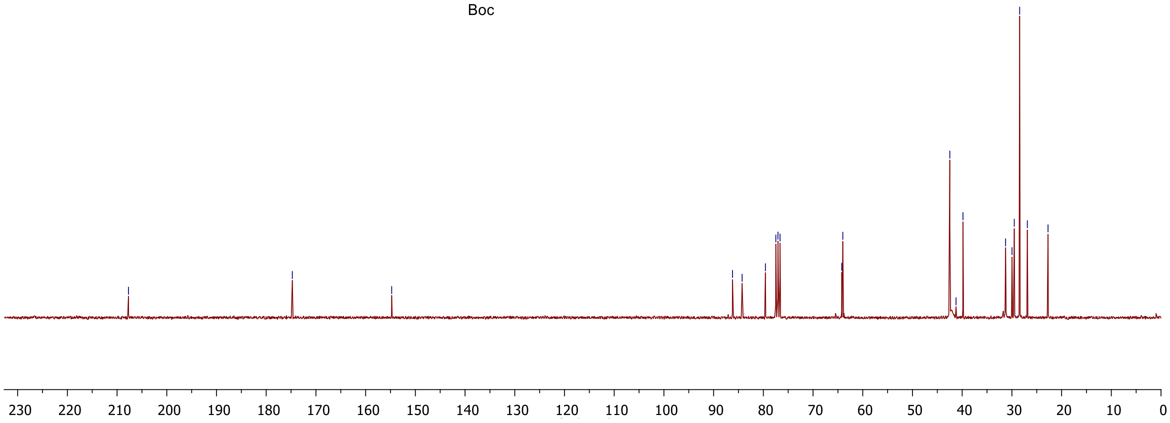

^{1}H-NMR (300 MHz, $CDCl_3$) of compound **S16**

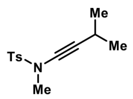

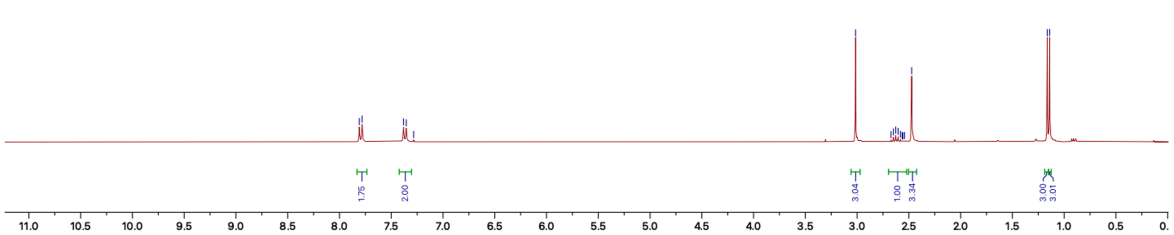

^{13}C-NMR (101 MHz, $CDCl_3$) of compound S**16**

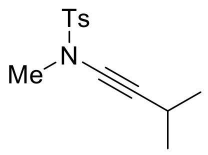

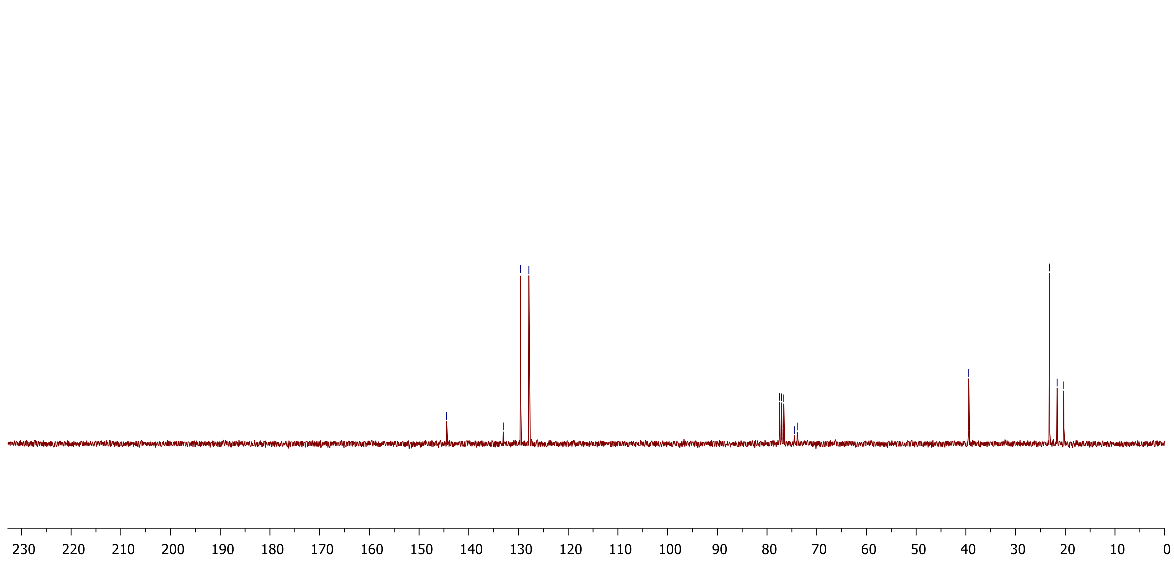

^{1}H-NMR (400 MHz, $CDCl_3$) of compound **S17**

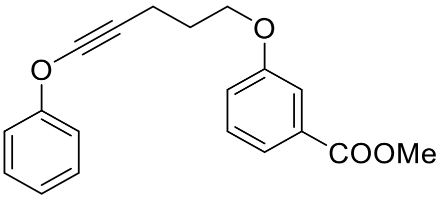

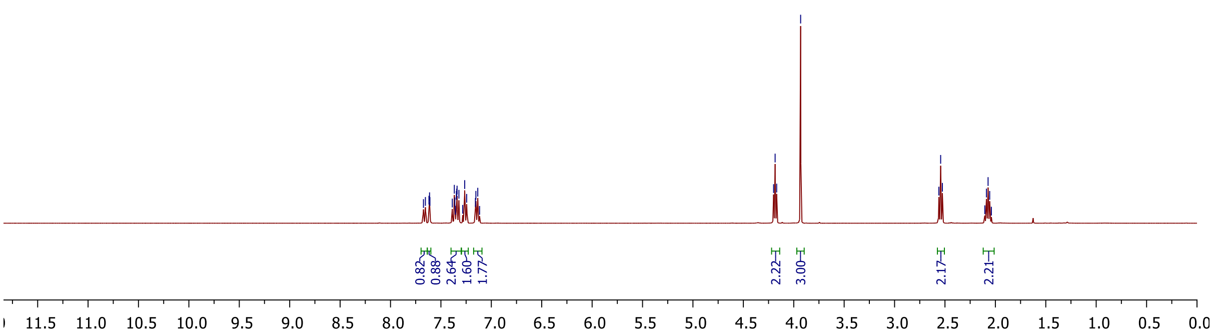

^{13}C-NMR (101 MHz, $CDCl_3$) of compound S**17**

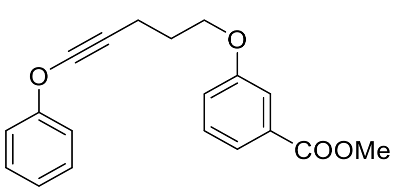

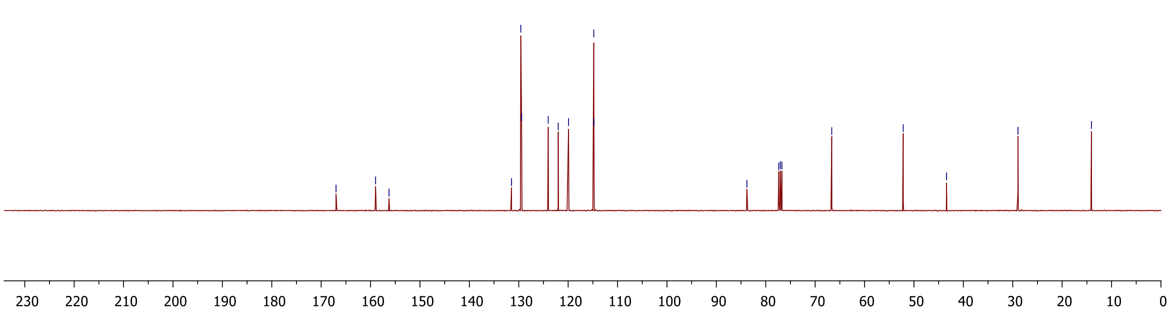

^{1}H NMR (400 MHz, $CDCl_3$) of compound **35**

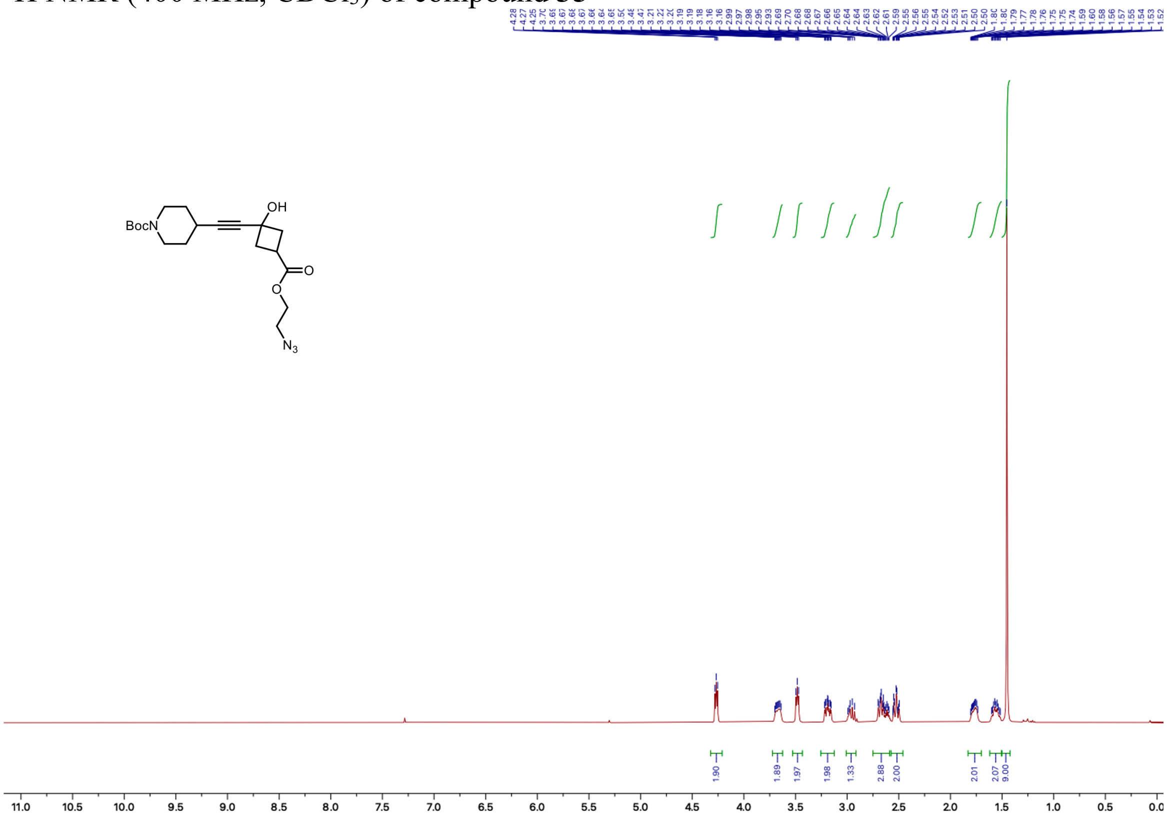

^{13}C NMR (75 MHz, $CDCl_3$) of compound **35**

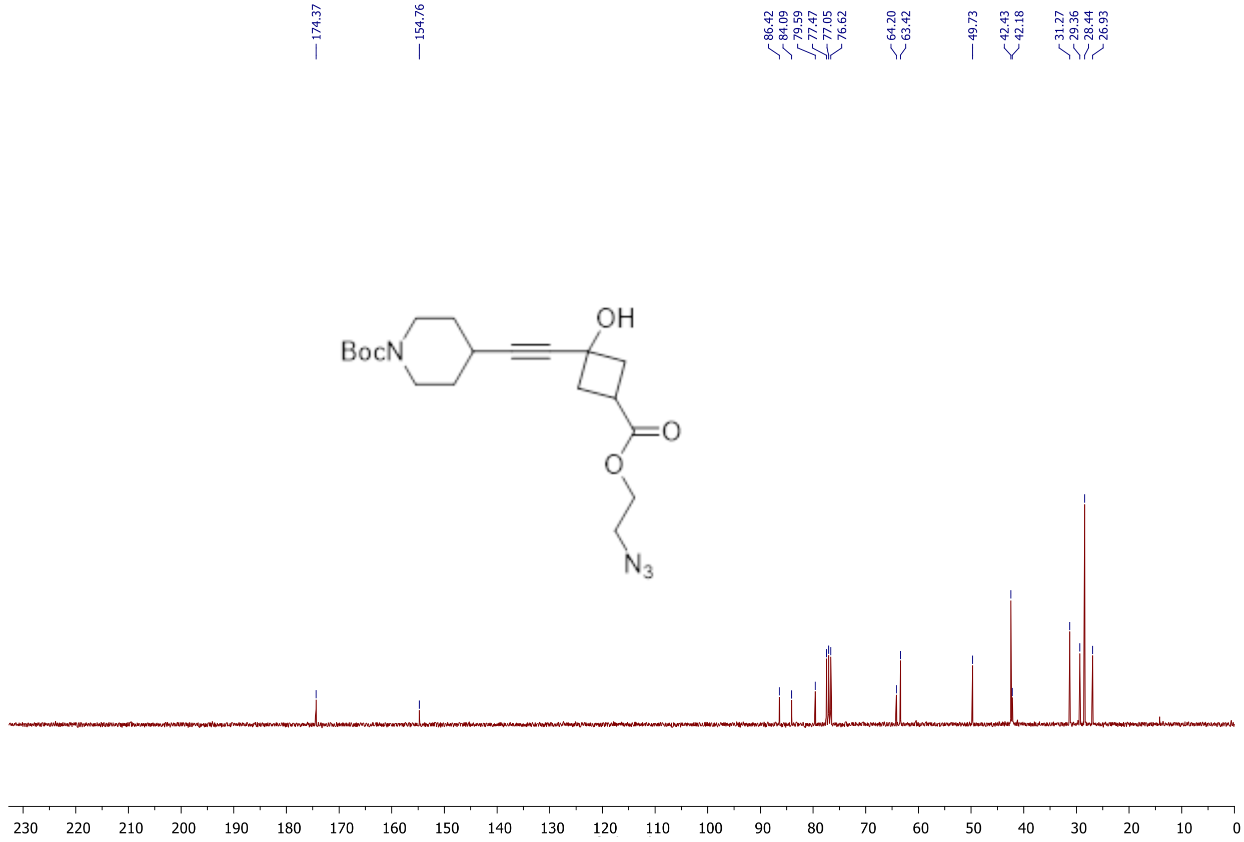

^{1}H NMR (400 MHz, $CDCl_3$) of compound **S18**

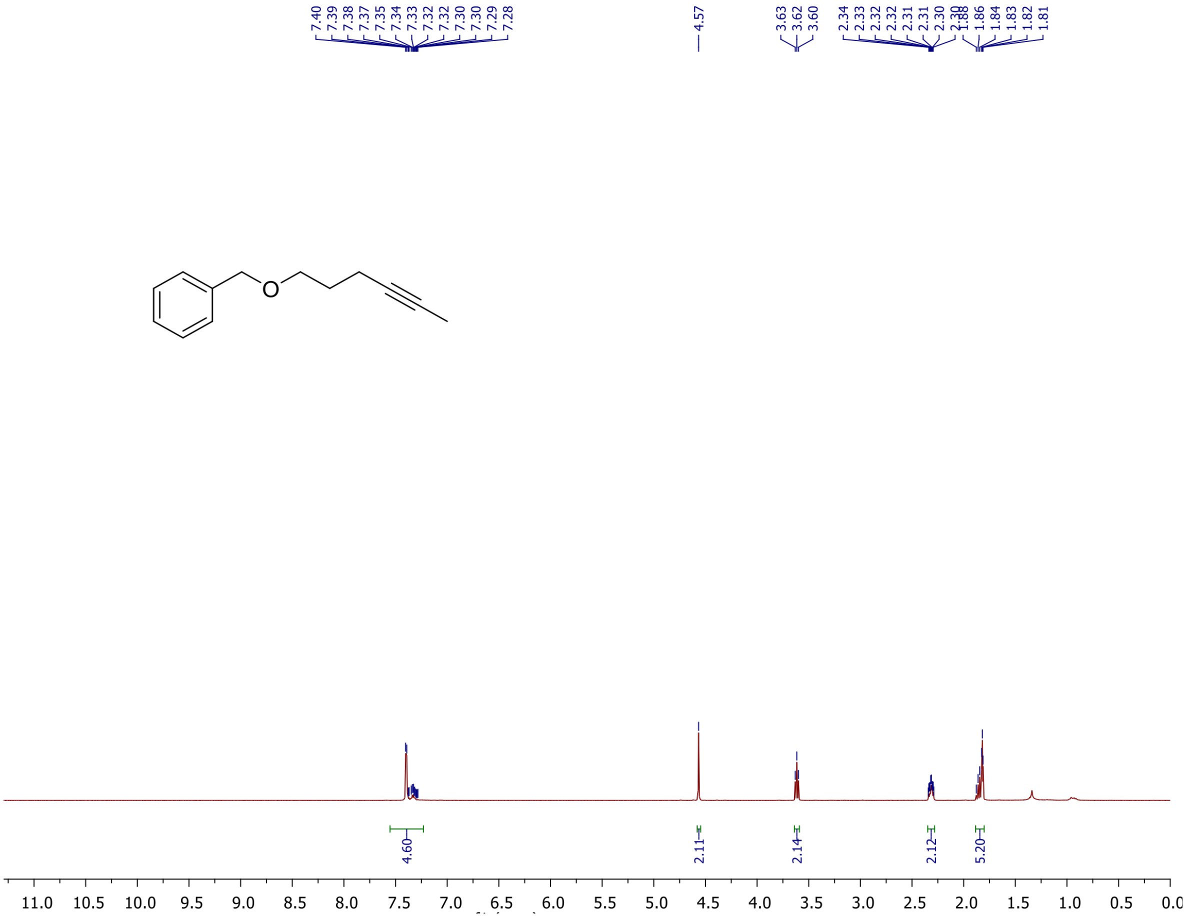

^{13}C NMR (101 MHz, $CDCl_3$) of compound **S18**

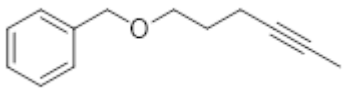

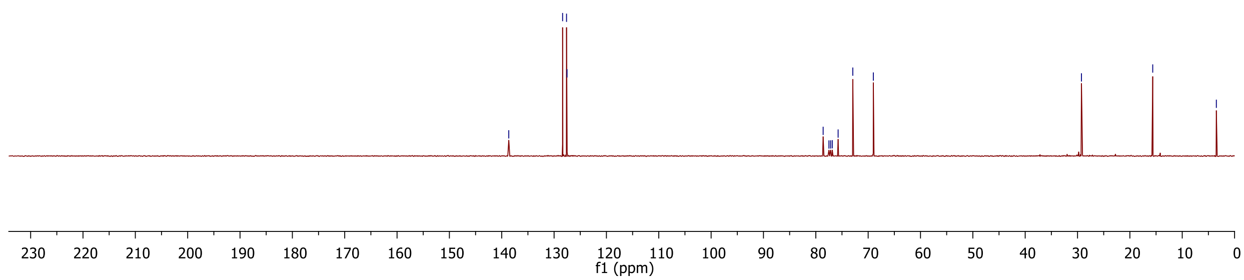

^{1}H NMR (400 MHz, $CDCl_3$) of compound **49**

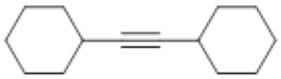

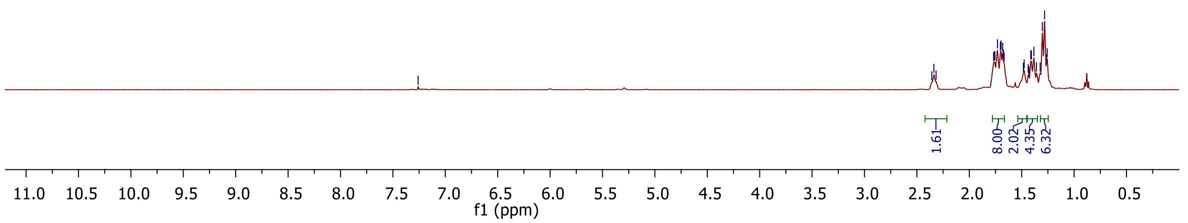

^{13}C NMR (101 MHz, $CDCl_3$) of compound **49**

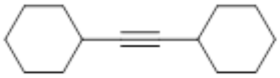

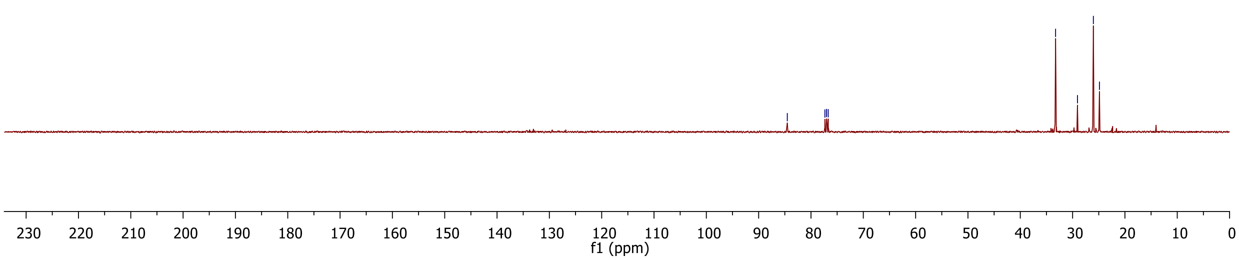

^{1}H NMR (300 MHz, $CDCl_3$) of compound **37**

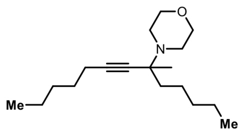

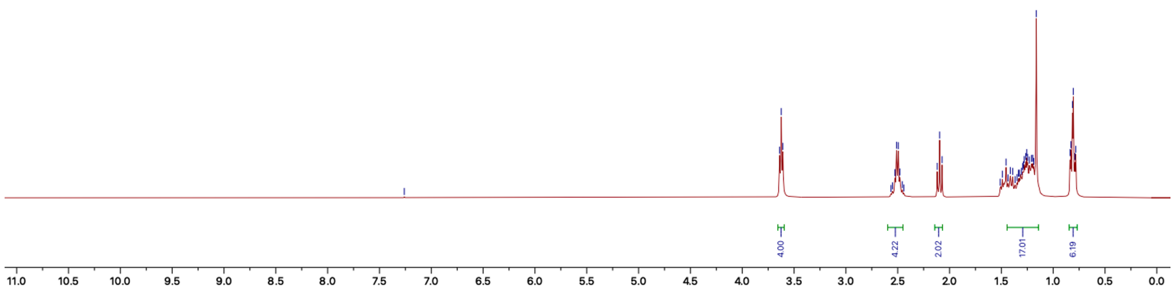

^{13}C NMR (101 MHz, $CDCl_3$) of compound **37**

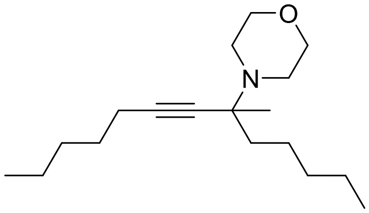

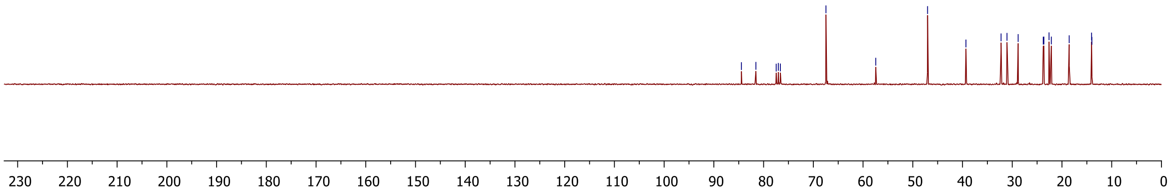

^{1}H NMR (800 MHz, $CDCl_3$) of compound **43**

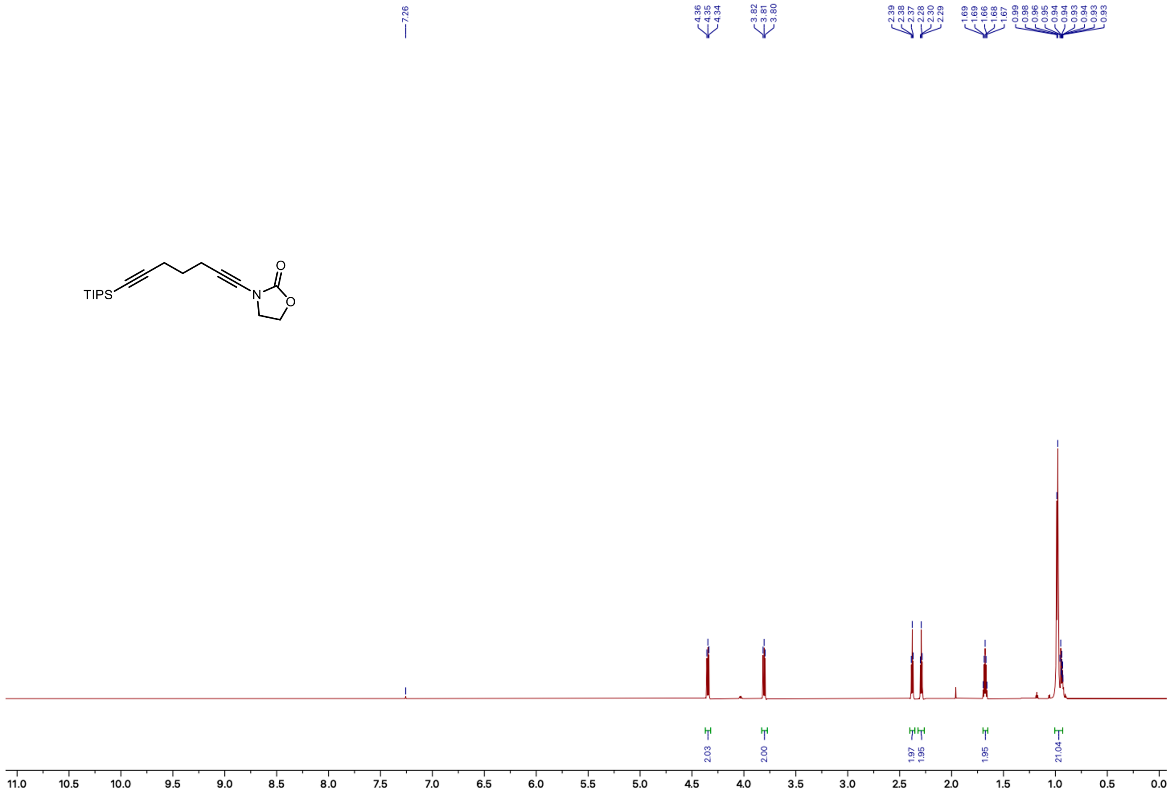

^{13}C NMR (201 MHz, $CDCl_3$) of compound **43**

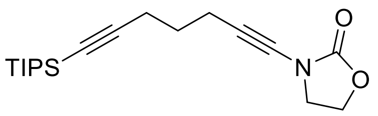

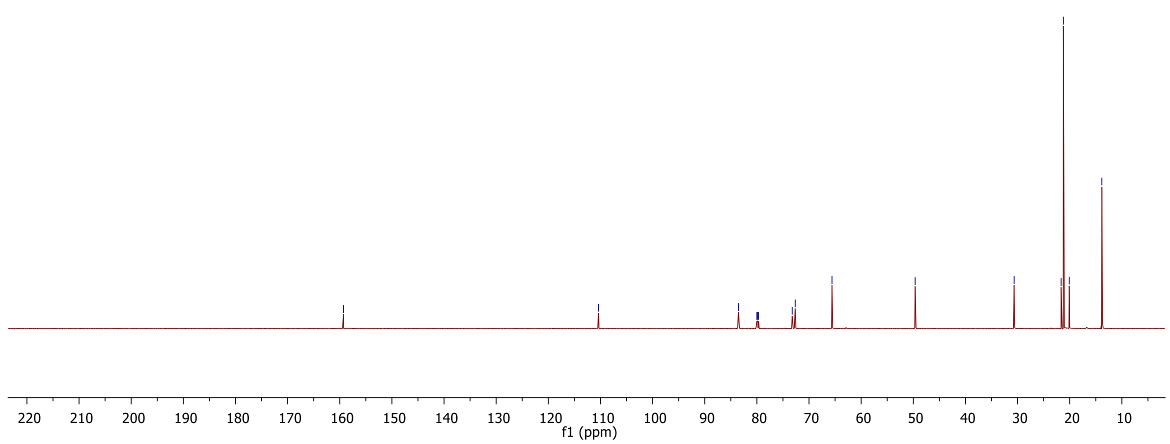

^{1}H NMR (400 MHz, $CDCl_3$) of compound **S19**

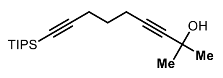

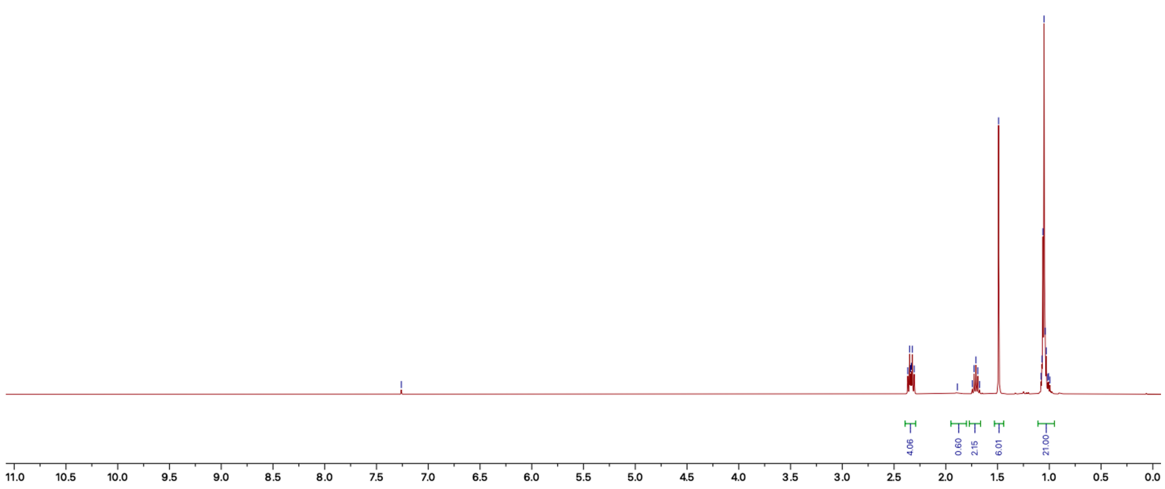

^{13}C NMR (101 MHz, $CDCl_3$) of compound **S19**

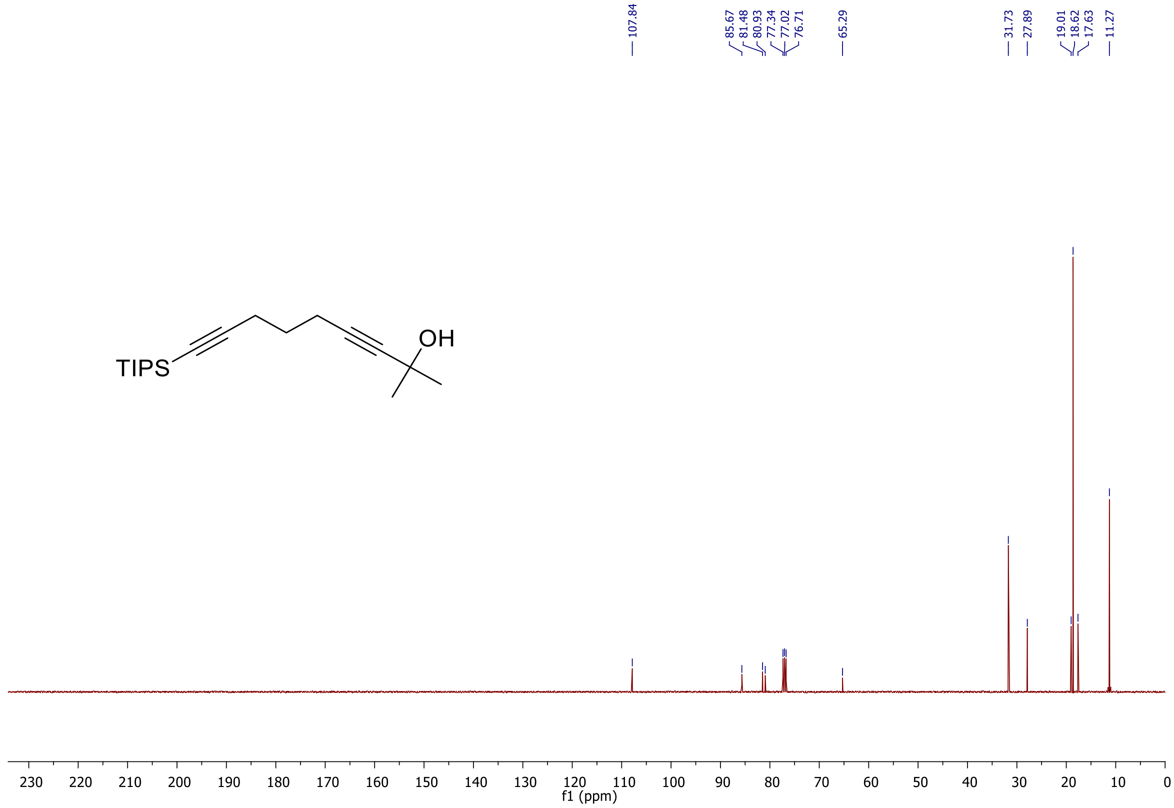

^{1}H NMR (300 MHz, $CDCl_3$) of compound **S20**

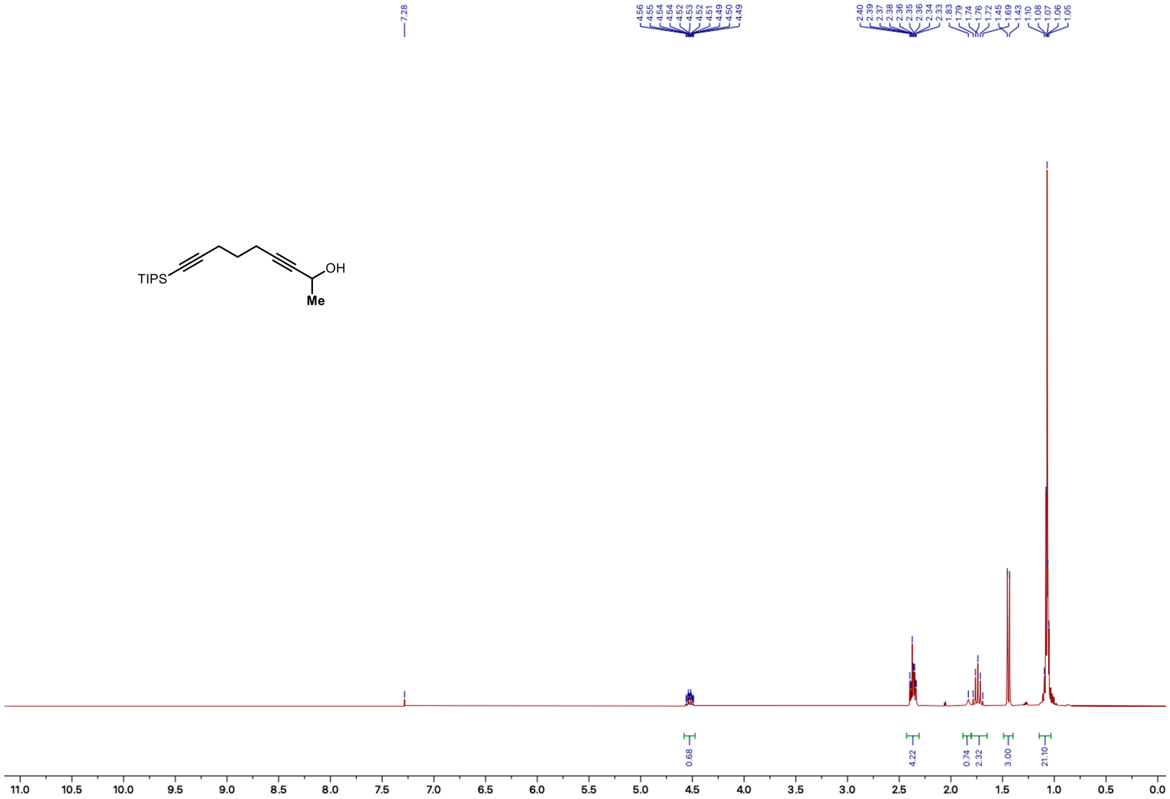

^{13}C NMR (75 MHz, $CDCl_3$) of compound **S20**

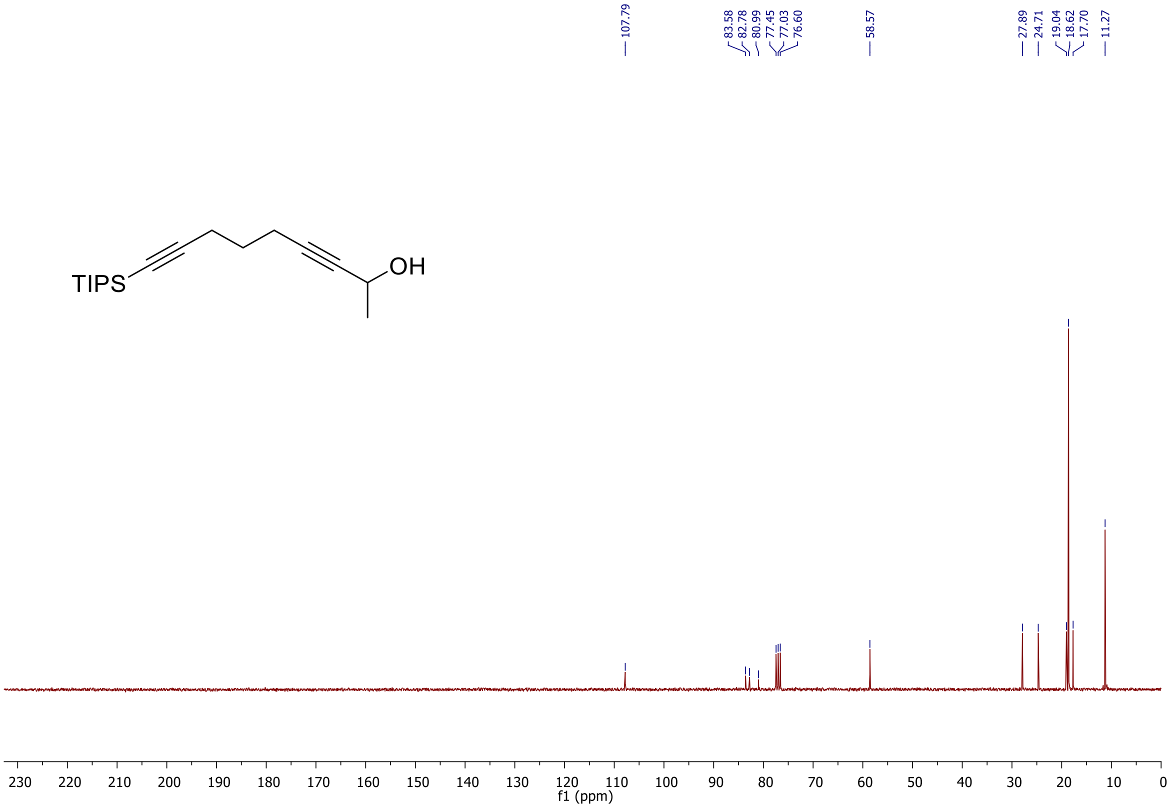

^{1}H NMR (300 MHz, $CDCl_3$) of compound **S21**

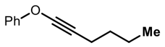

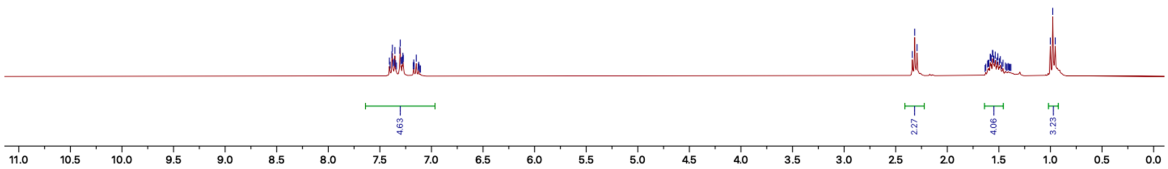

^{13}C NMR (75 MHz, $CDCl_3$) of compound **S21**

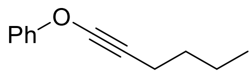

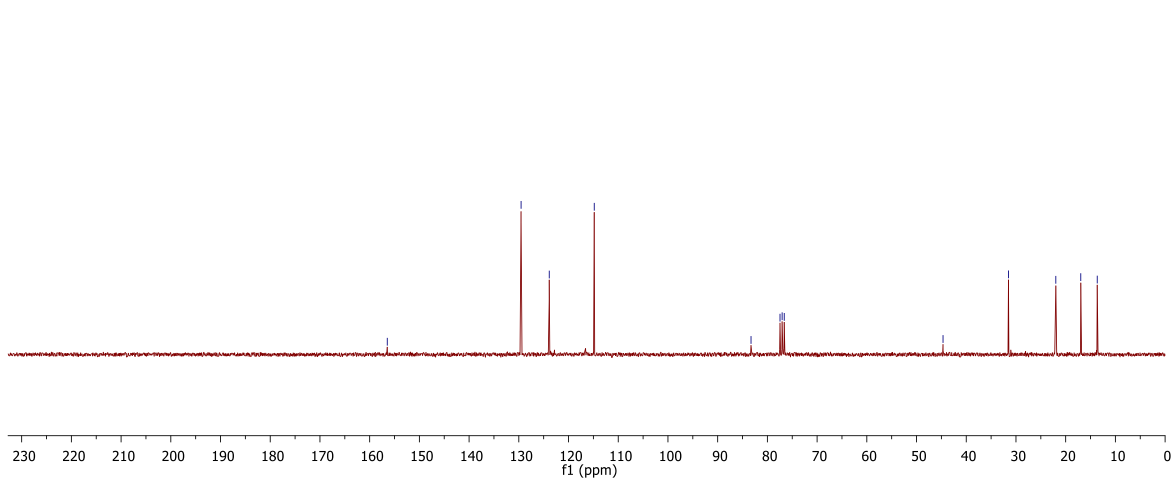

^{1}H NMR (300 MHz, $CDCl_3$) of compound **S22**

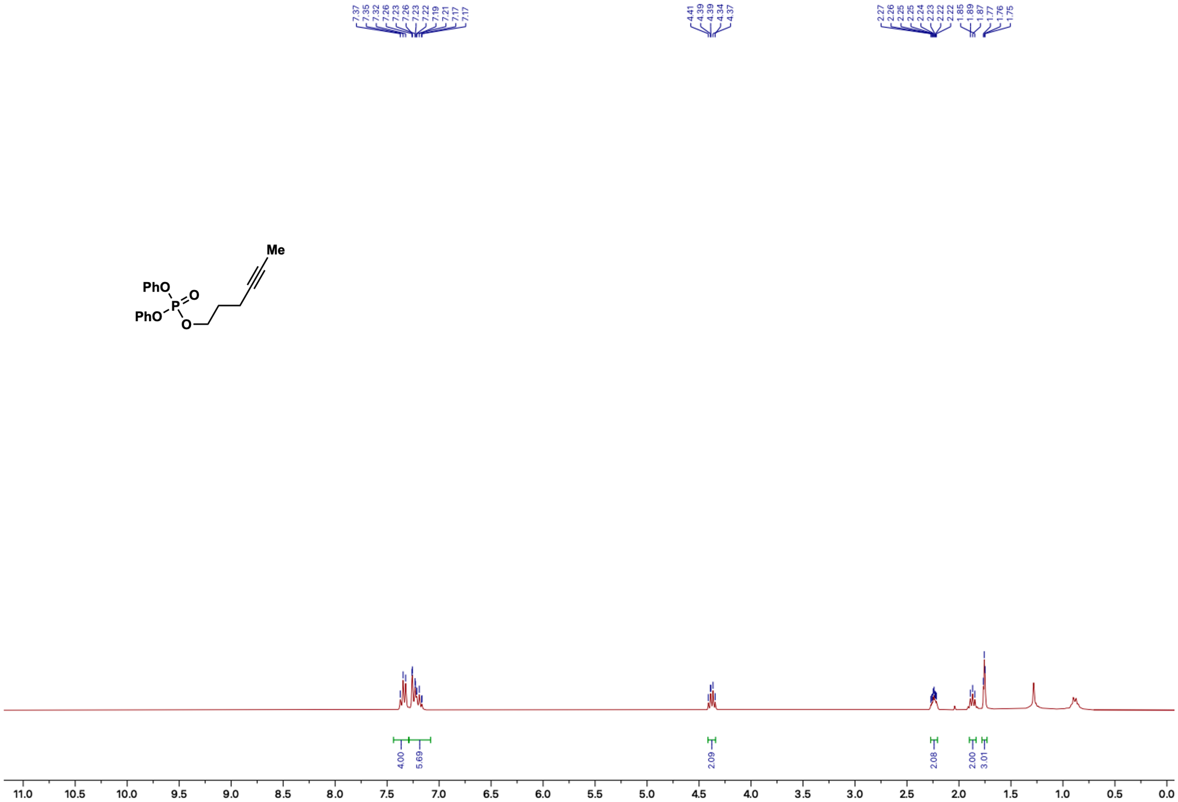

^{13}C NMR (75 MHz, $CDCl_3$) of compound **S22**

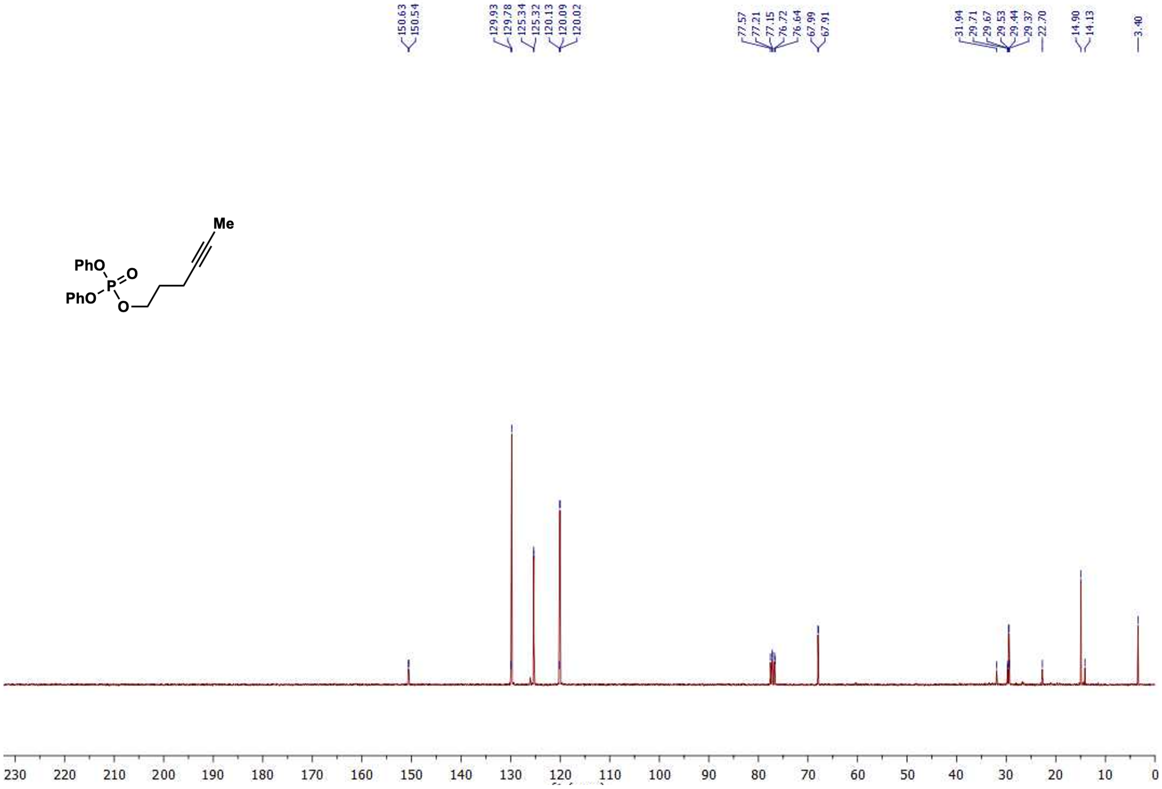

^{31}P NMR (75 MHz, $CDCl_3$) of compound **S22**

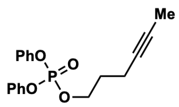

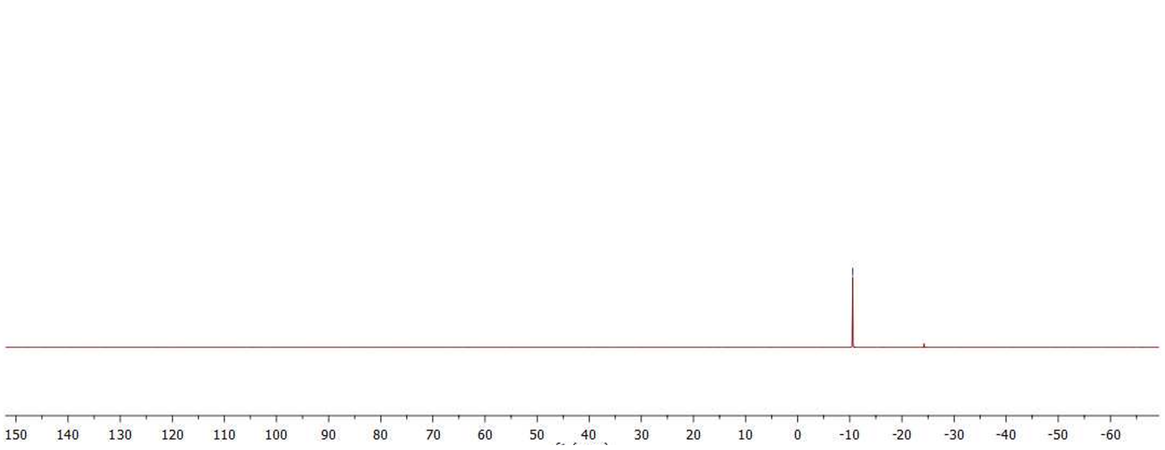

^{1}H NMR (300 MHz, $CDCl_3$) of compound **S23**

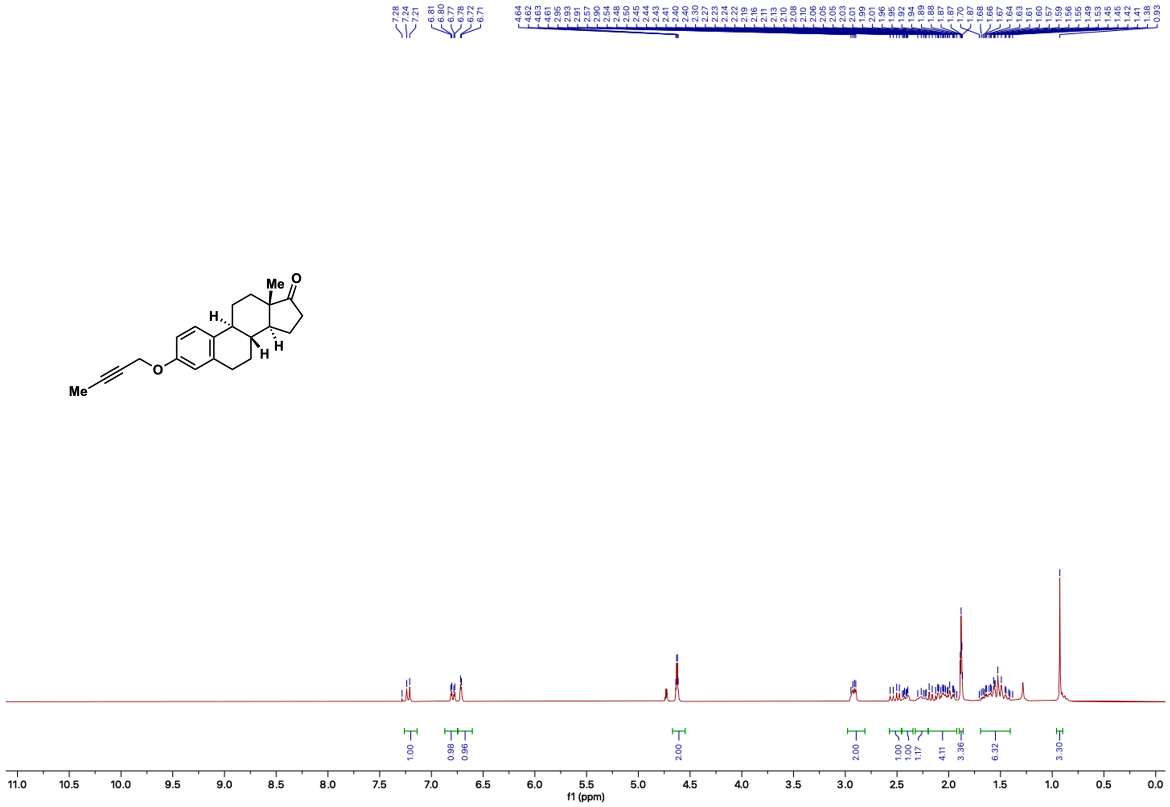

^{13}C NMR (75 MHz, $CDCl_3$) of compound **S23**

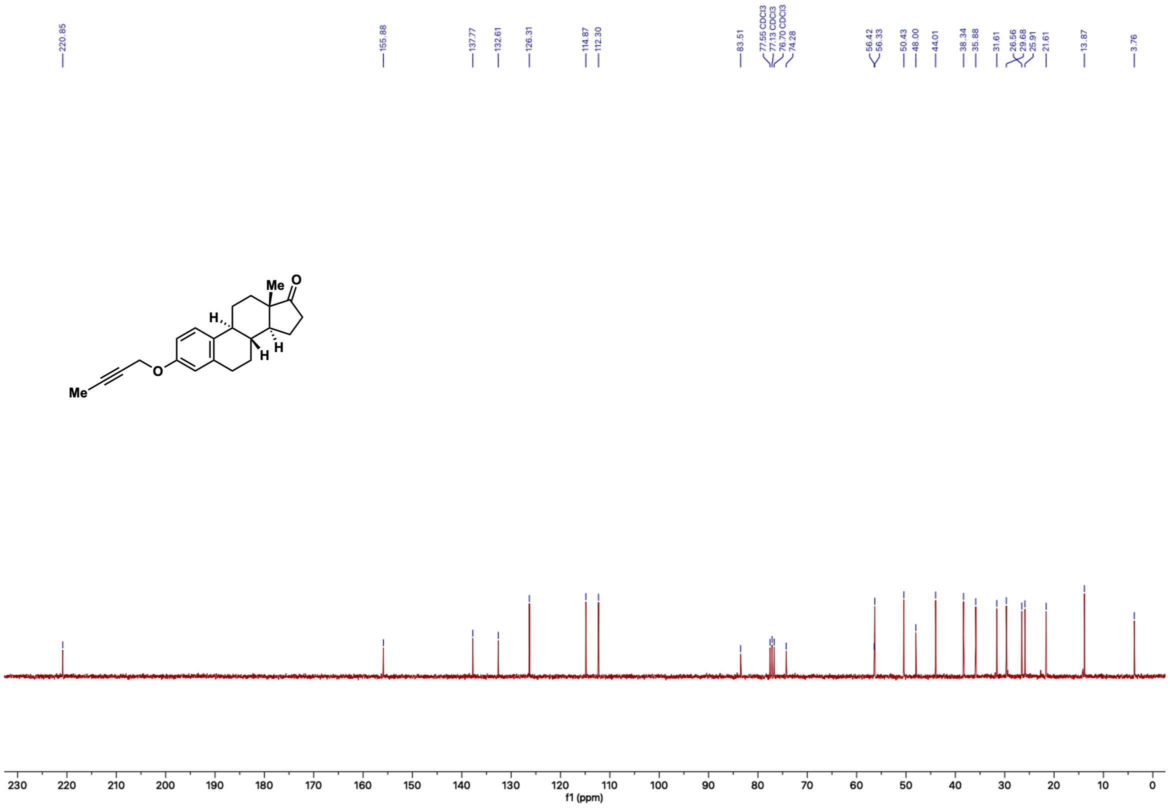

^{1}H NMR (300 MHz, $CDCl_3$) of compound **S24**

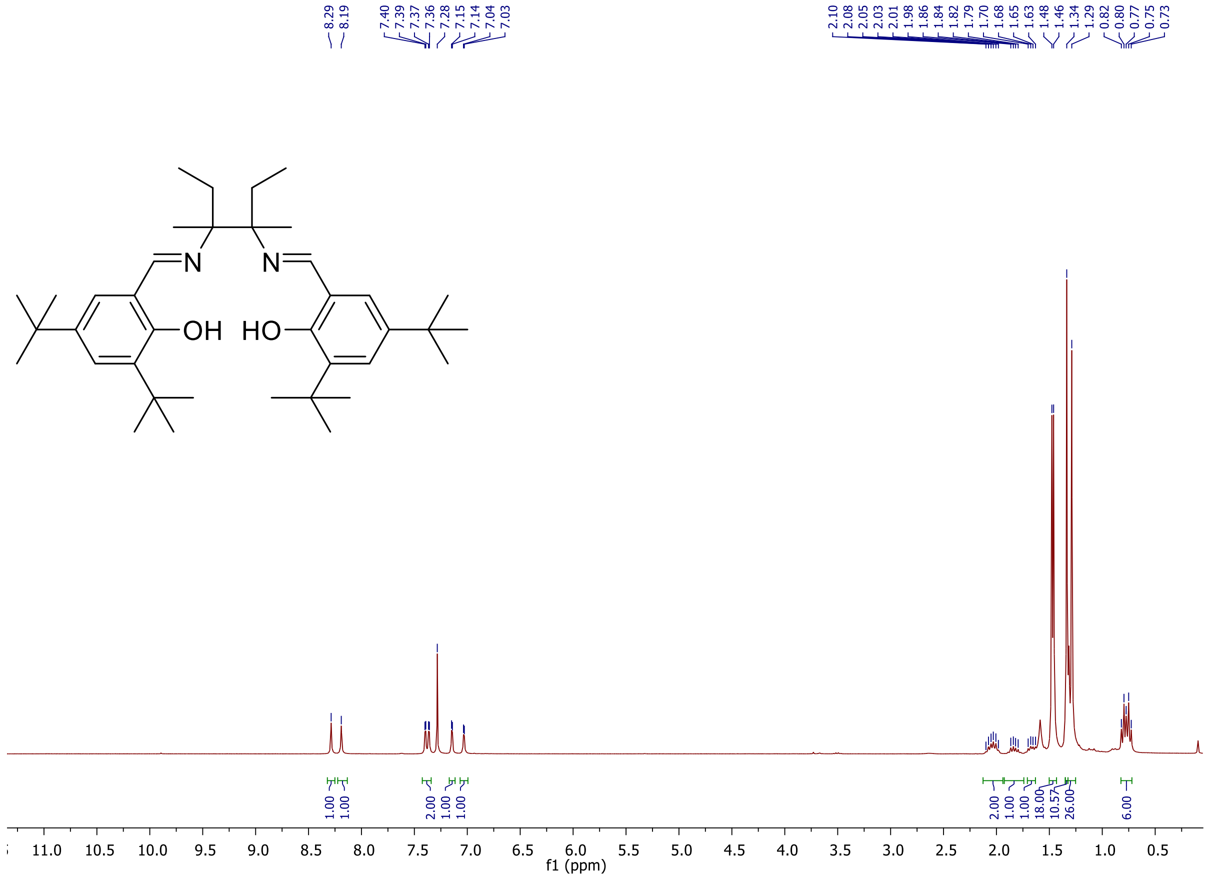

^{13}C NMR (201 MHz, $CDCl_3$) of compound **S24**

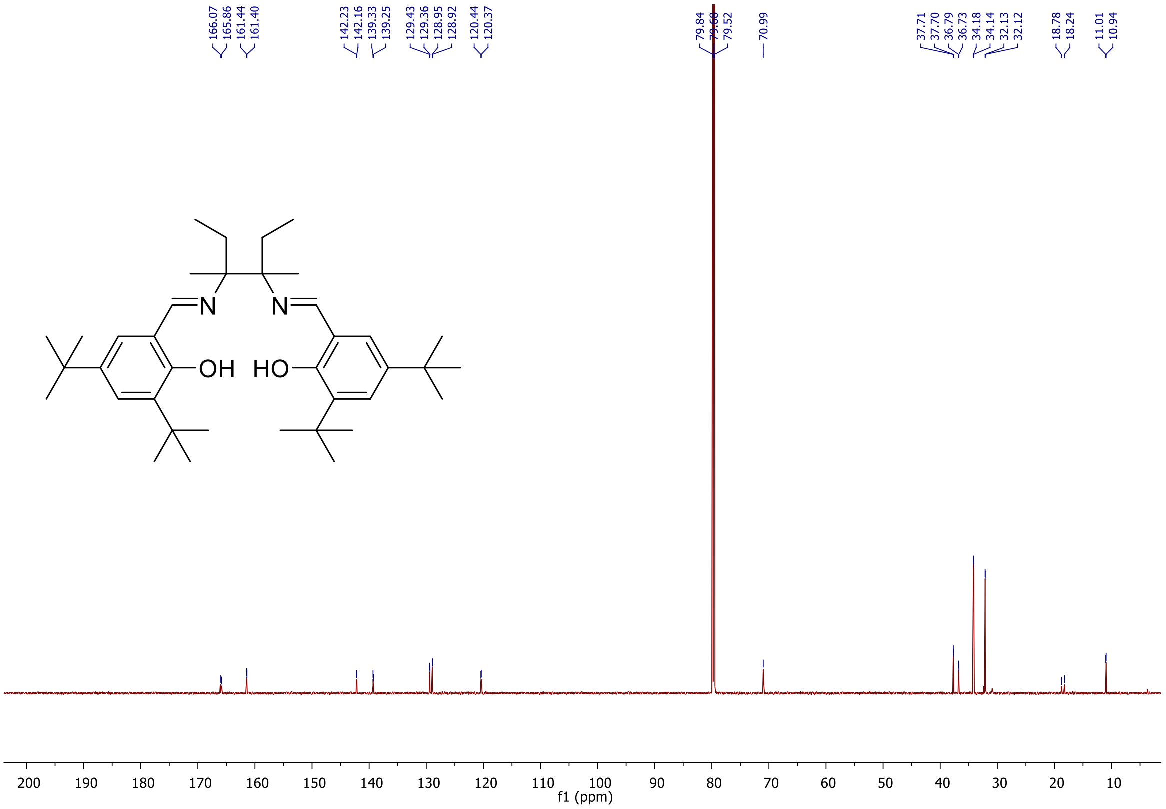

^{1}H-NMR (300 MHz, $CDCl_3$) of compound **2**

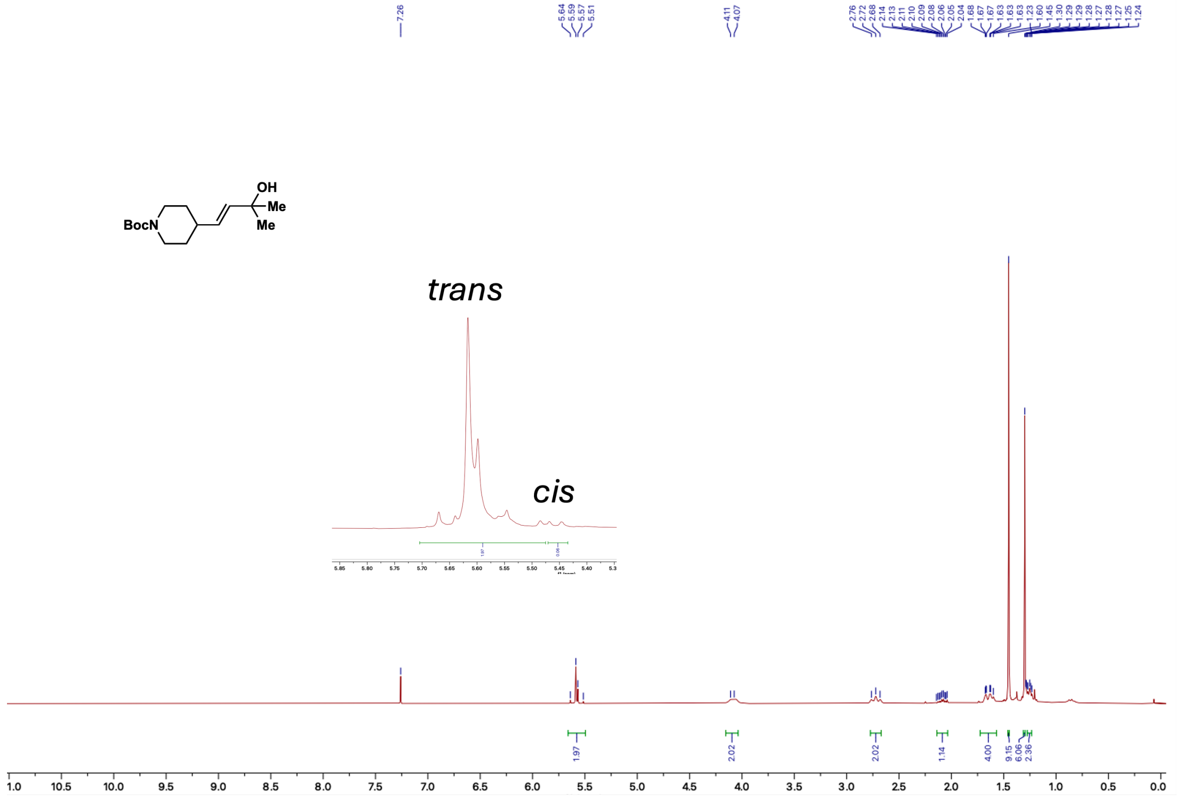

^{13}C-NMR (125 MHz, $CDCl_3$) of compound **2**

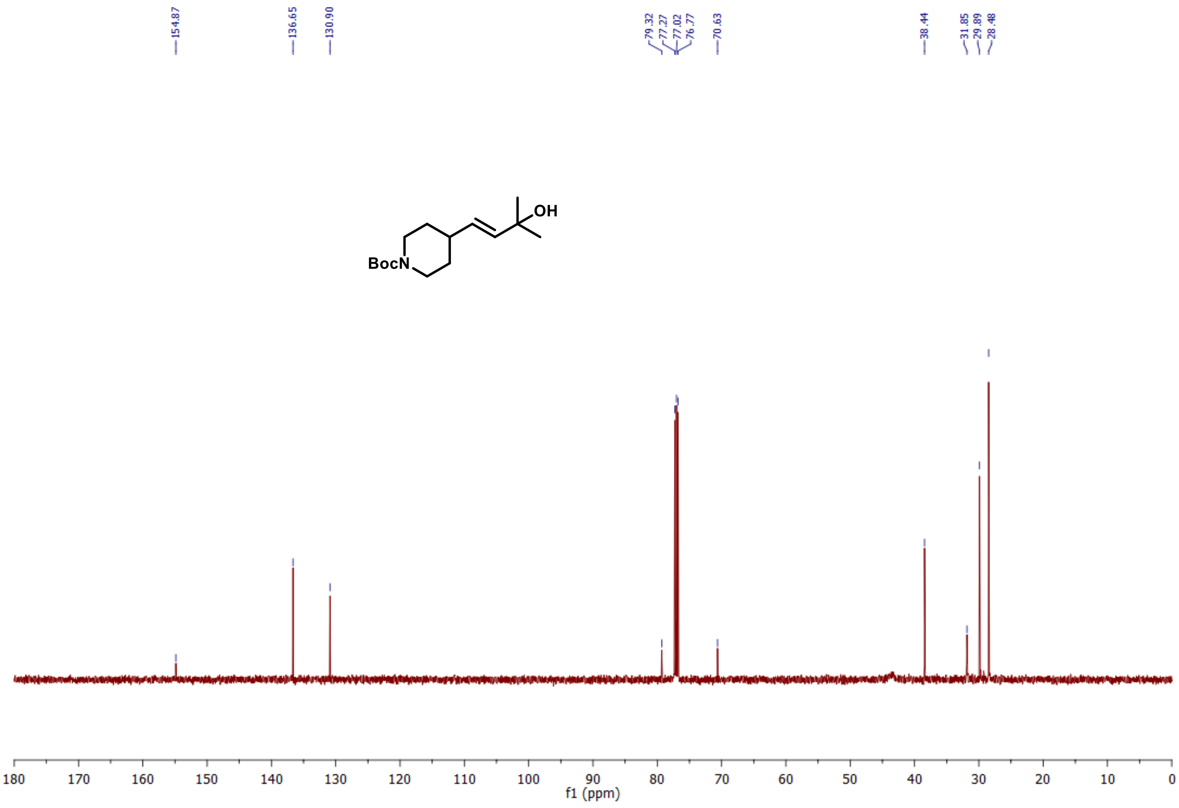

^{1}H-NMR (300 MHz, $CDCl_3$) of compound **3**

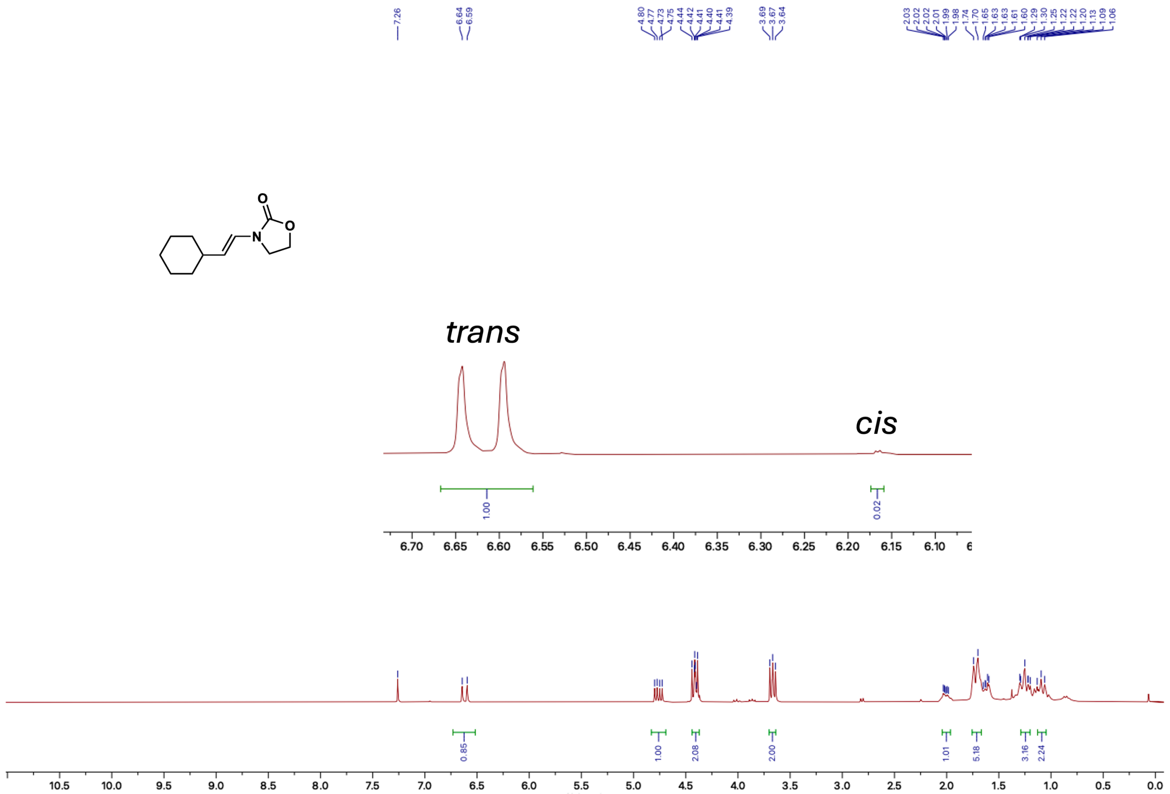

^{13}C-NMR (101 MHz, $CDCl_3$) of compound **3**

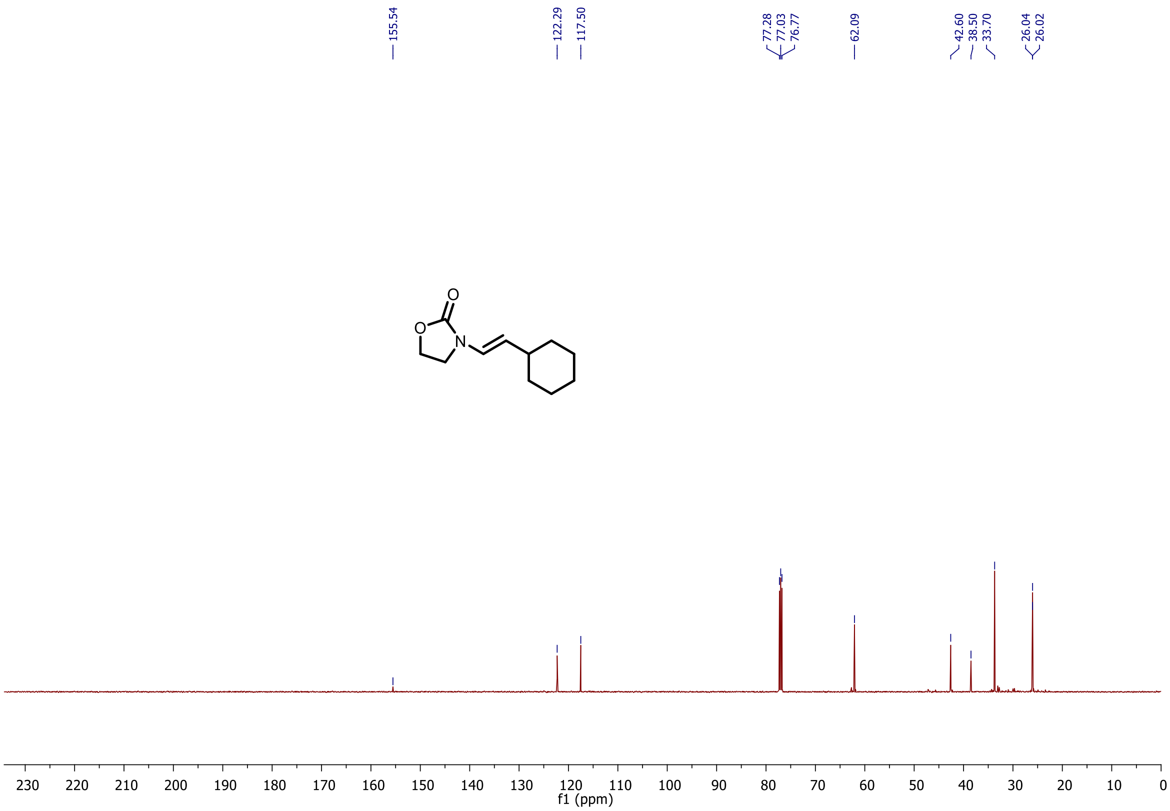

^{1}H-NMR (300 MHz, $CDCl_3$) of compound **4**

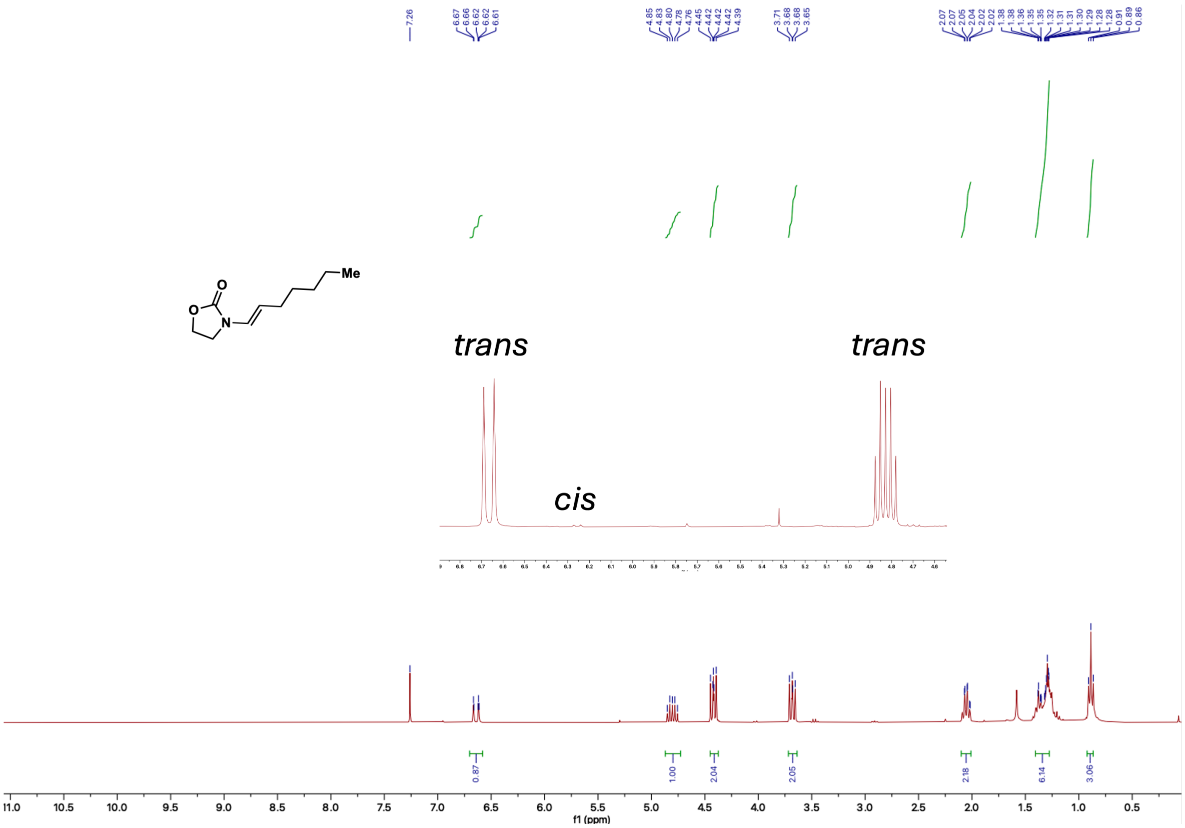

^{13}C-NMR (101 MHz, $CDCl_3$) of compound **4**

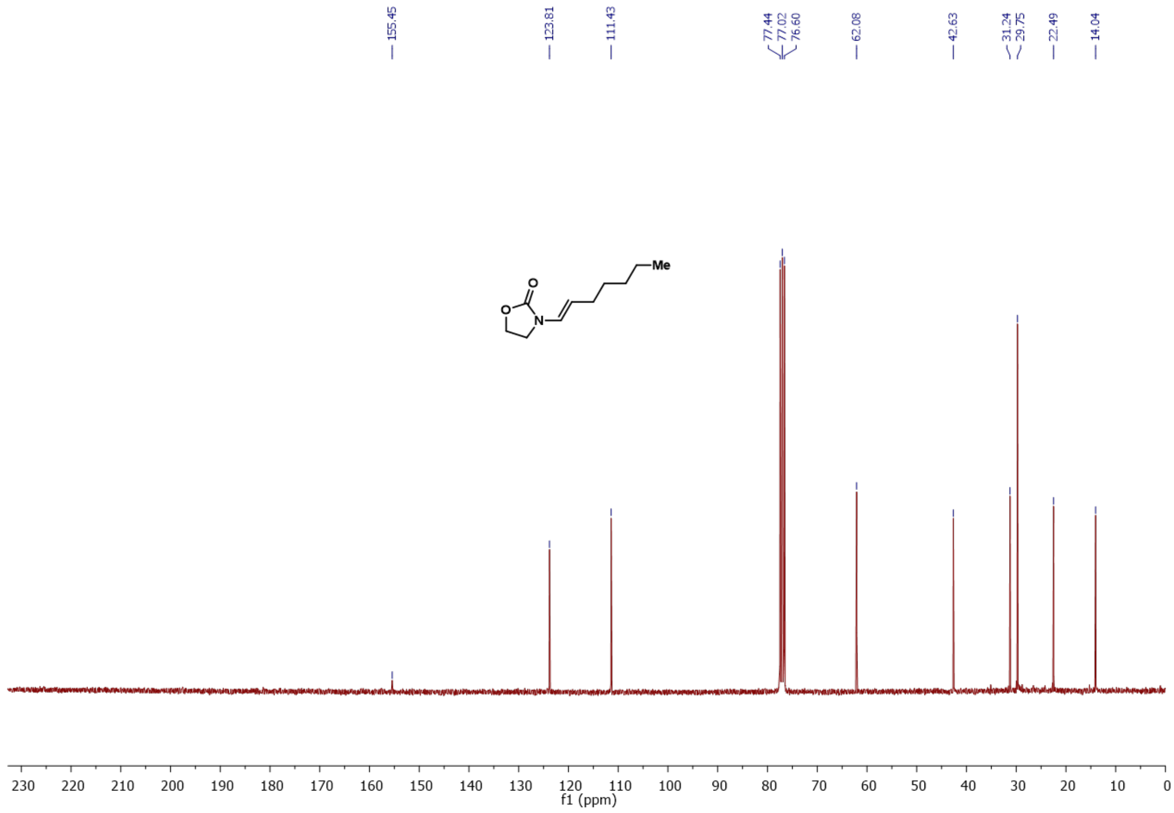

^{1}H-NMR (300 MHz, $CDCl_3$) of compound **5**

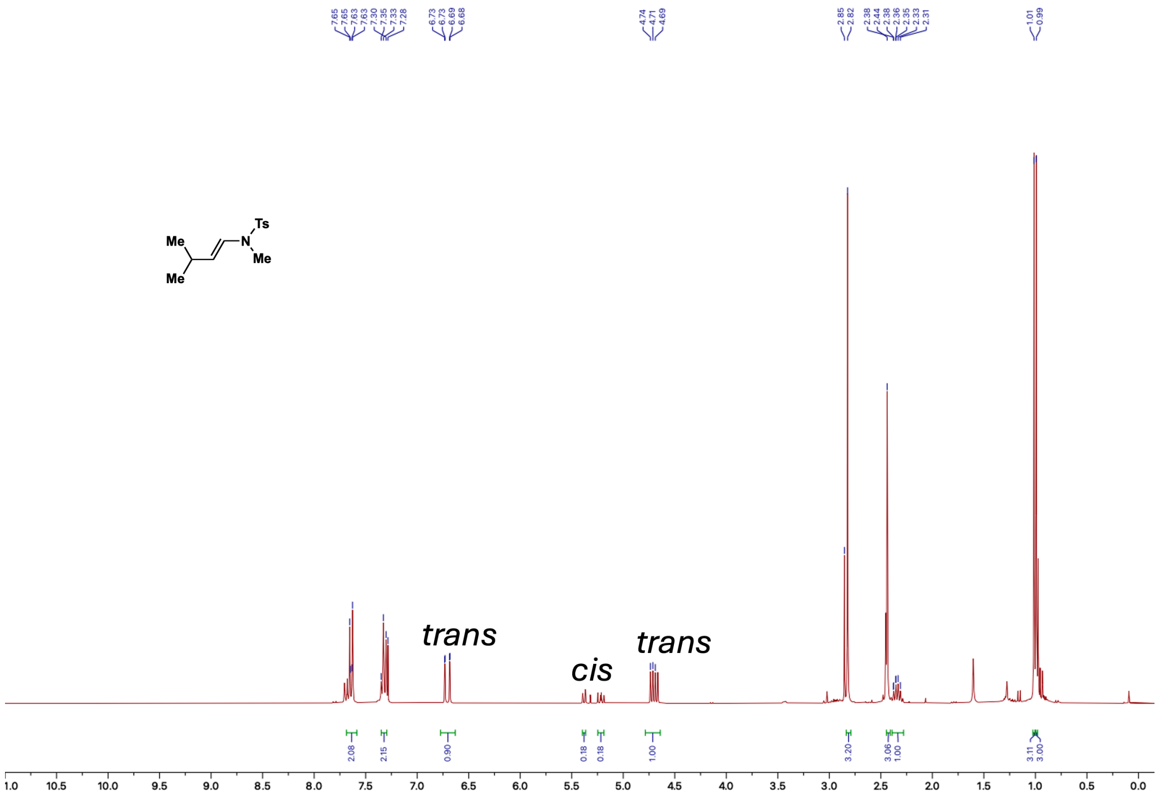

^{13}C-NMR (75MHz, $CDCl_3$) of compound **5**

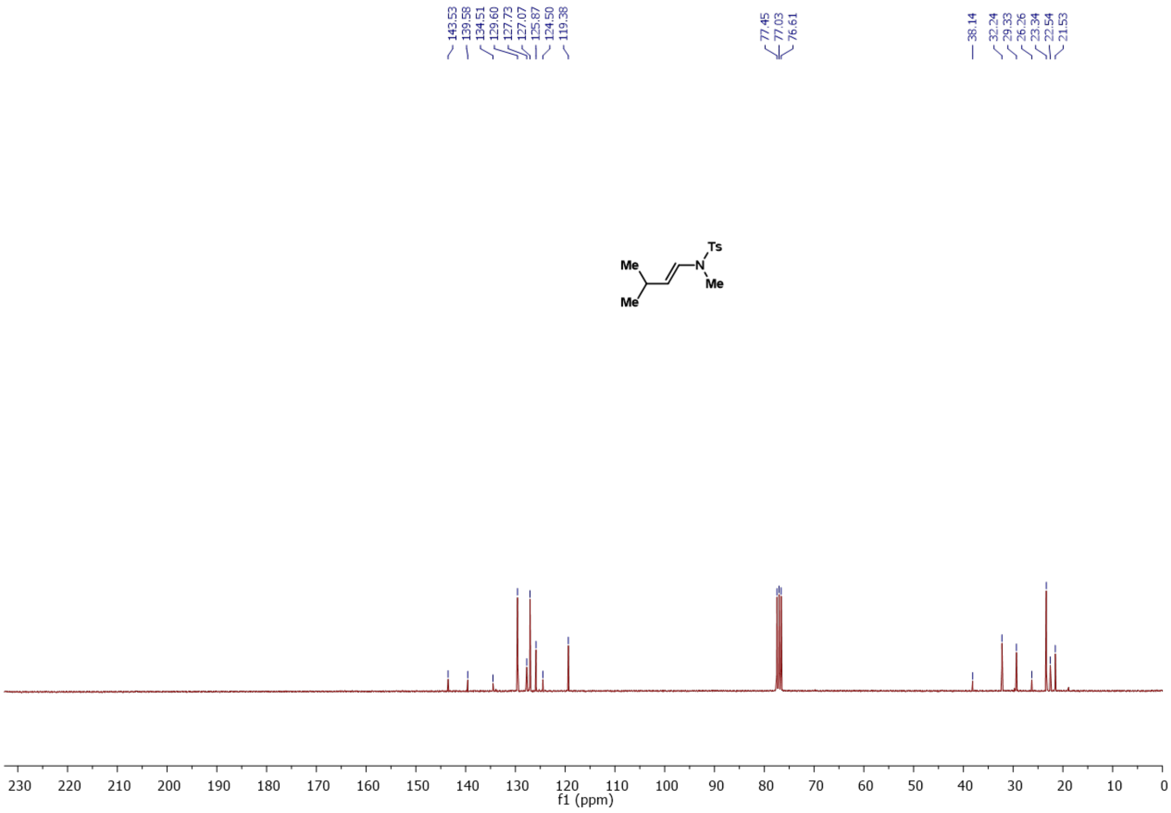

^{1}H-NMR (300 MHz, $CDCl_3$) of compound **6**

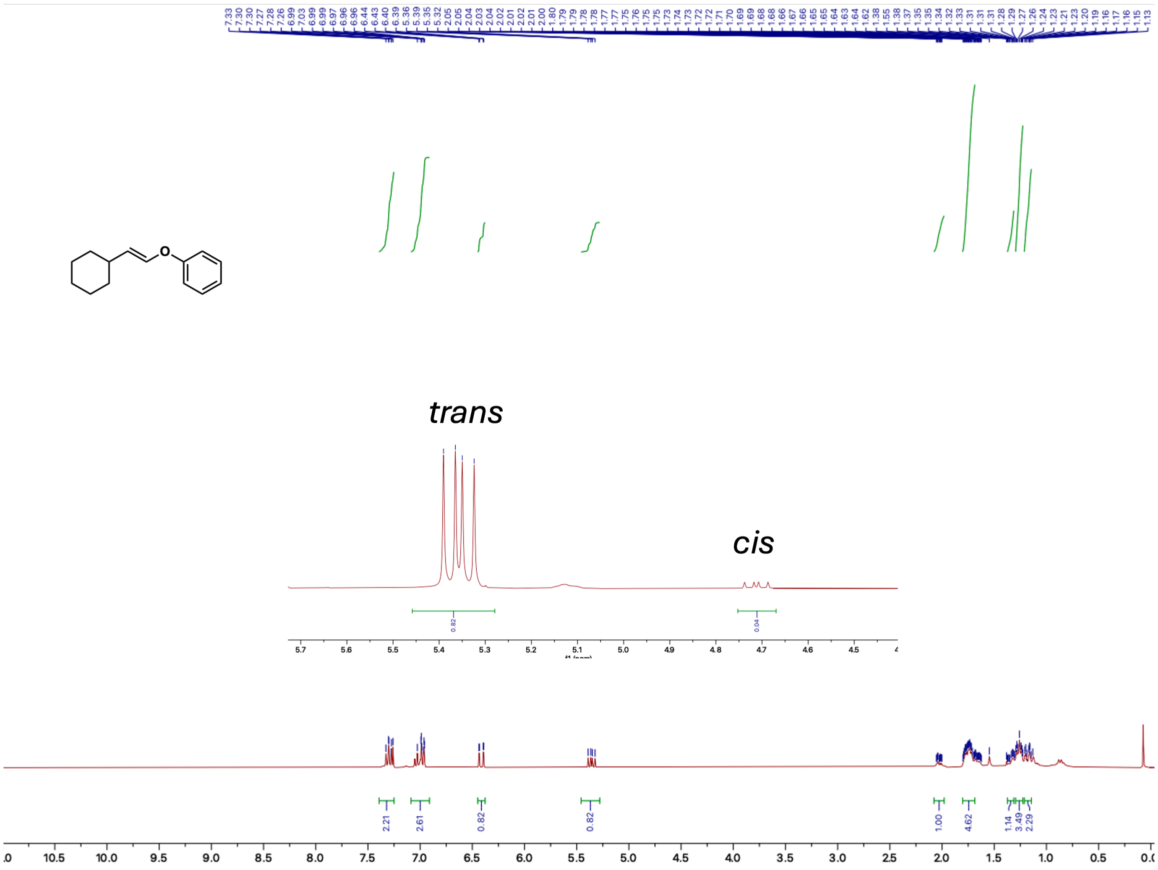

^{13}C-NMR (101 MHz, $CDCl_3$) of compound **6**

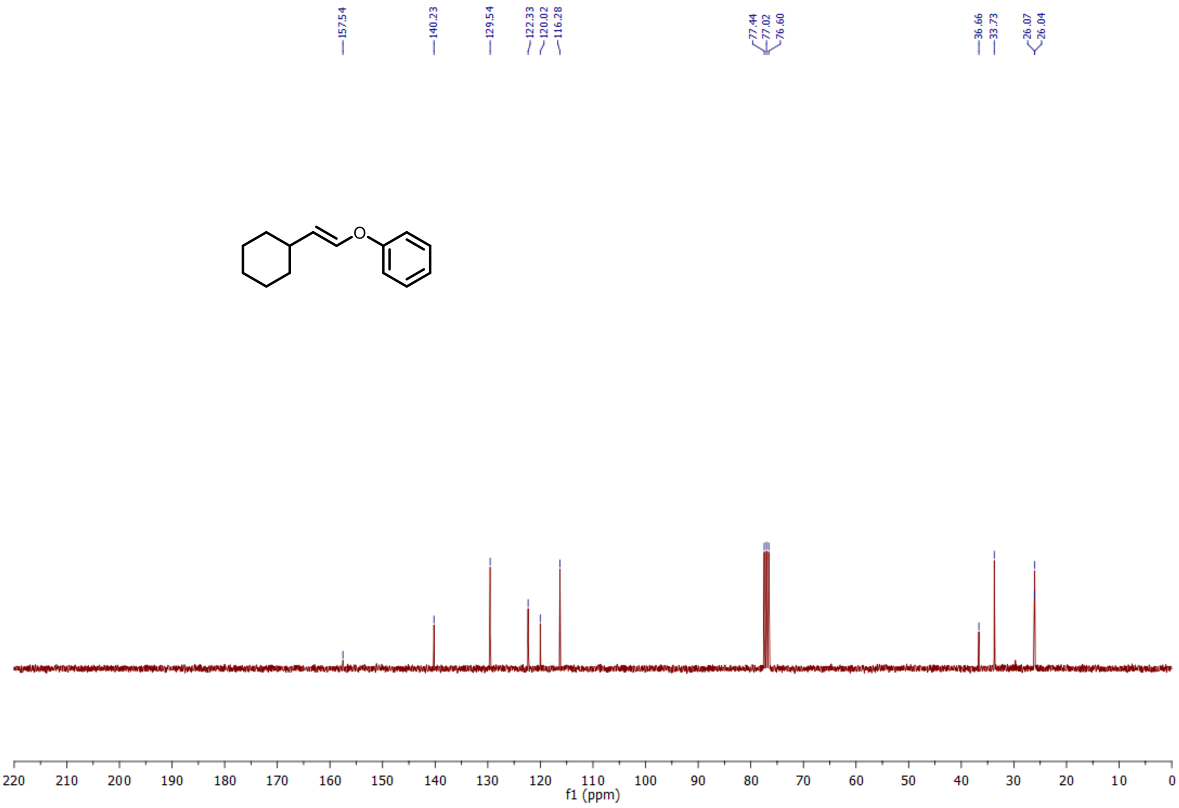

^{1}H-NMR (300 MHz, $CDCl_3$) of compound **7**

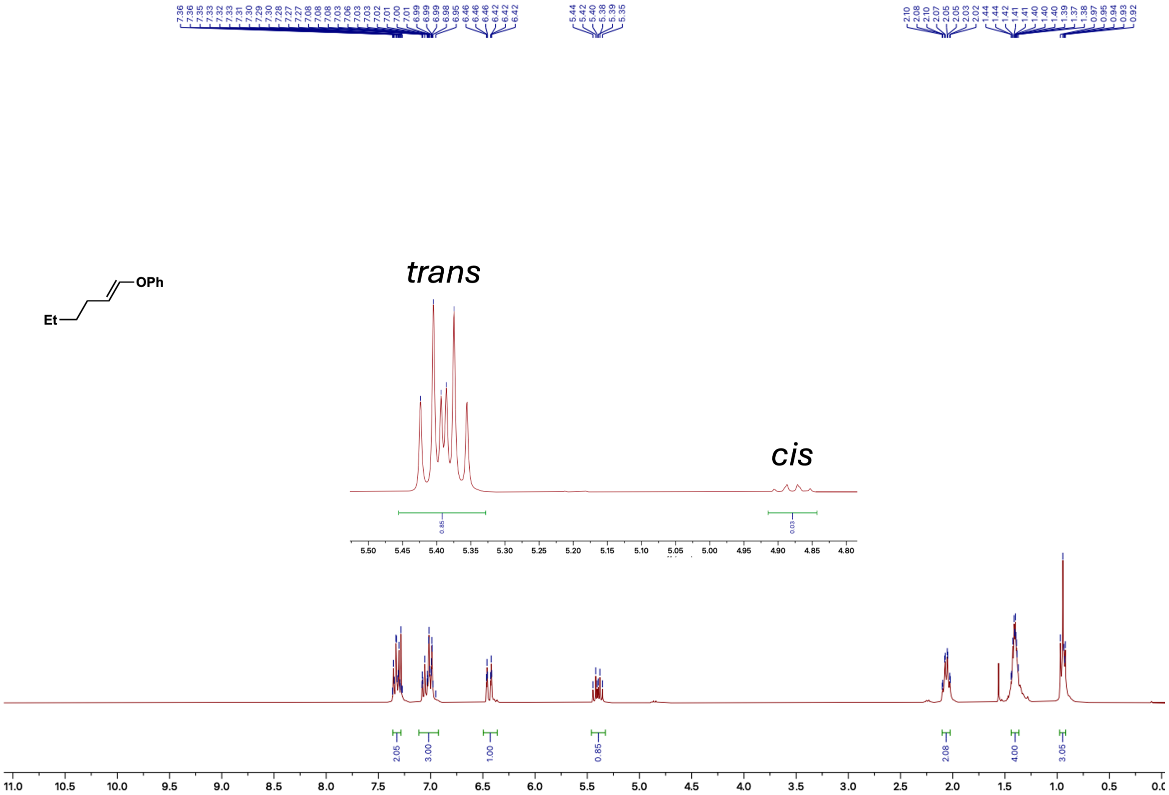

^{13}C-NMR (75MHz, $CDCl_3$) of compound **7**

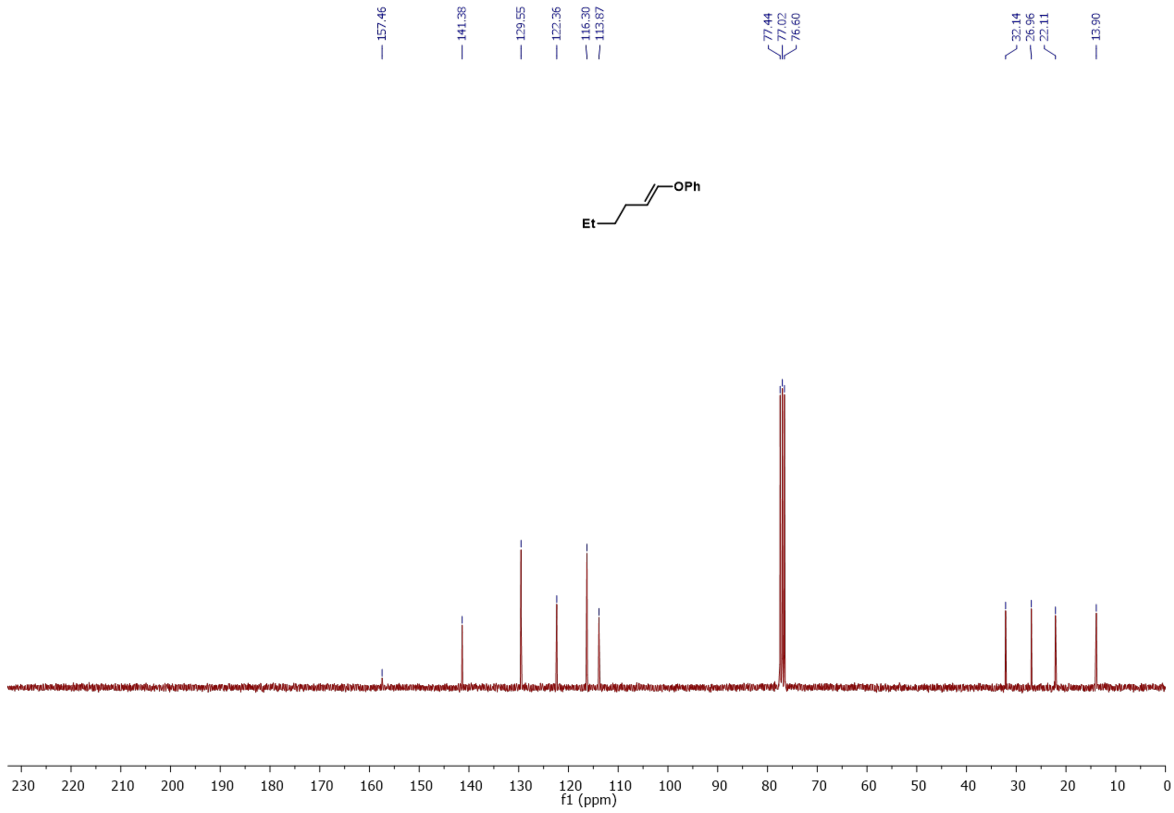

^{1}H NMR (400 MHz, $CDCl_3$) of compound **8**

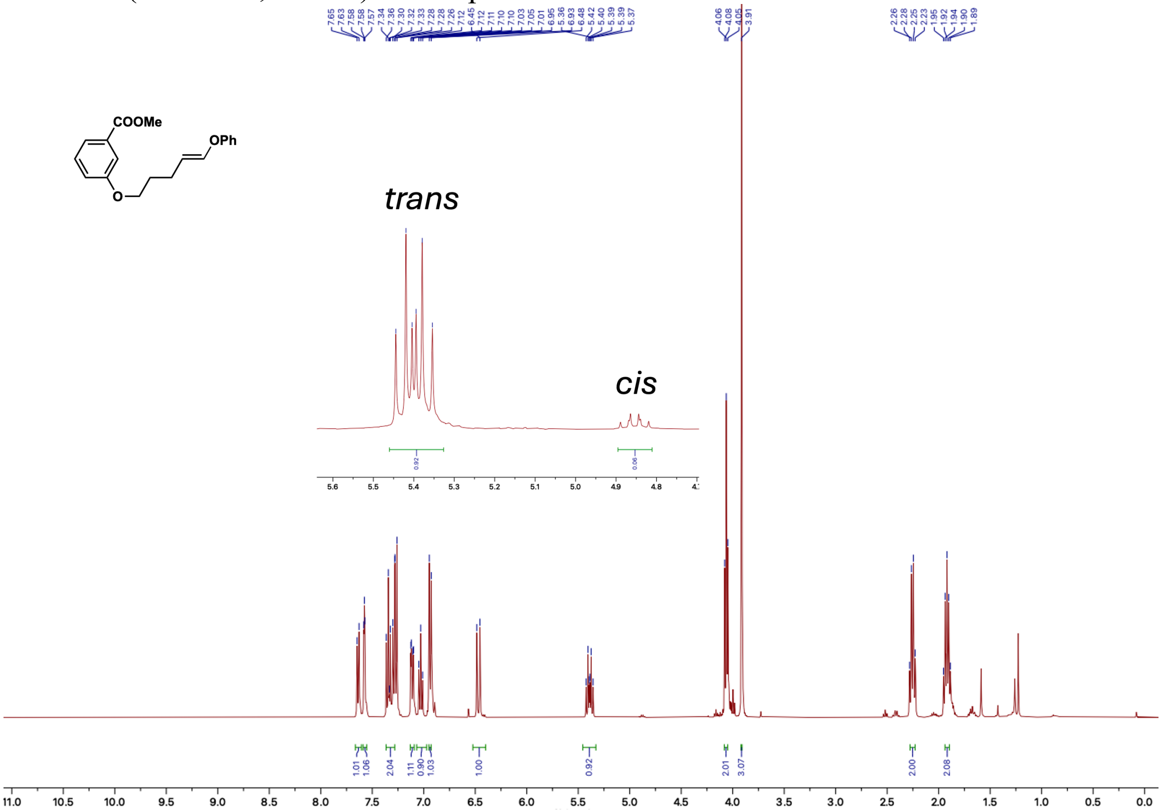

^{13}C NMR (101 MHz, $CDCl_3$) of compound **8**

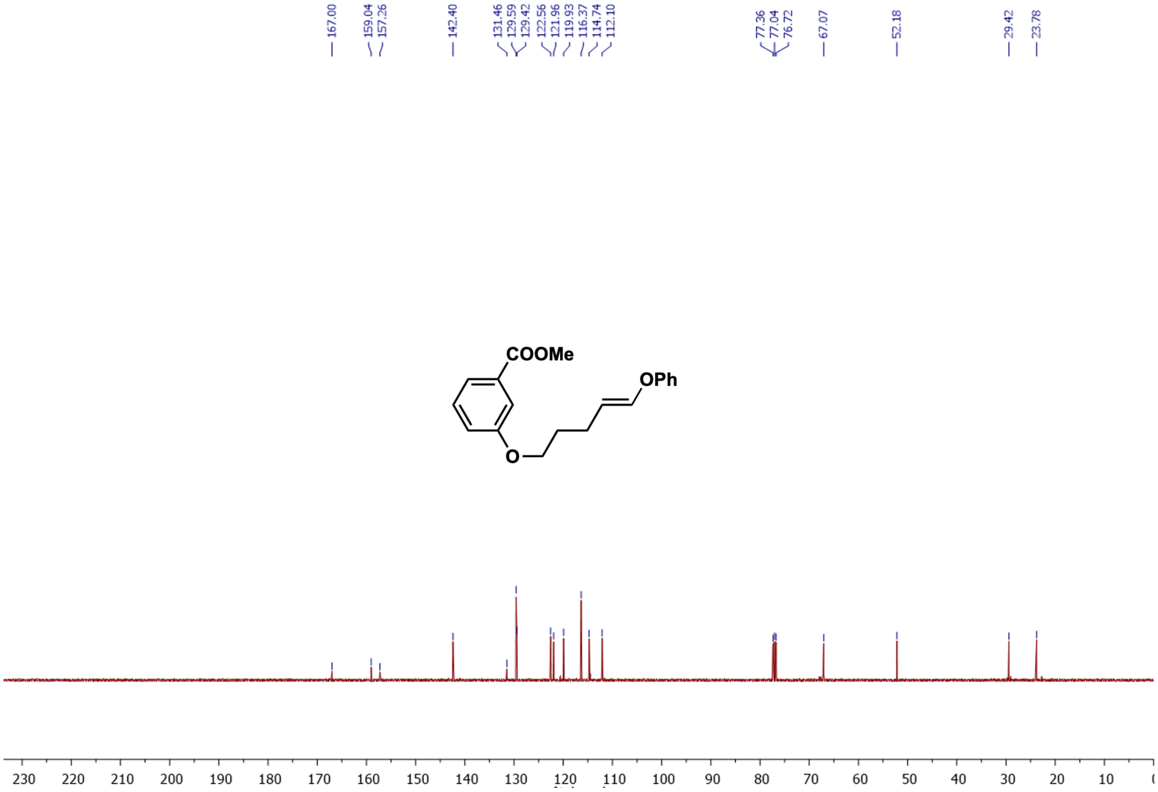

^{1}H-NMR (300 MHz, $CDCl_3$) of compound **9** (E/Z ratio was determined using GCMS)

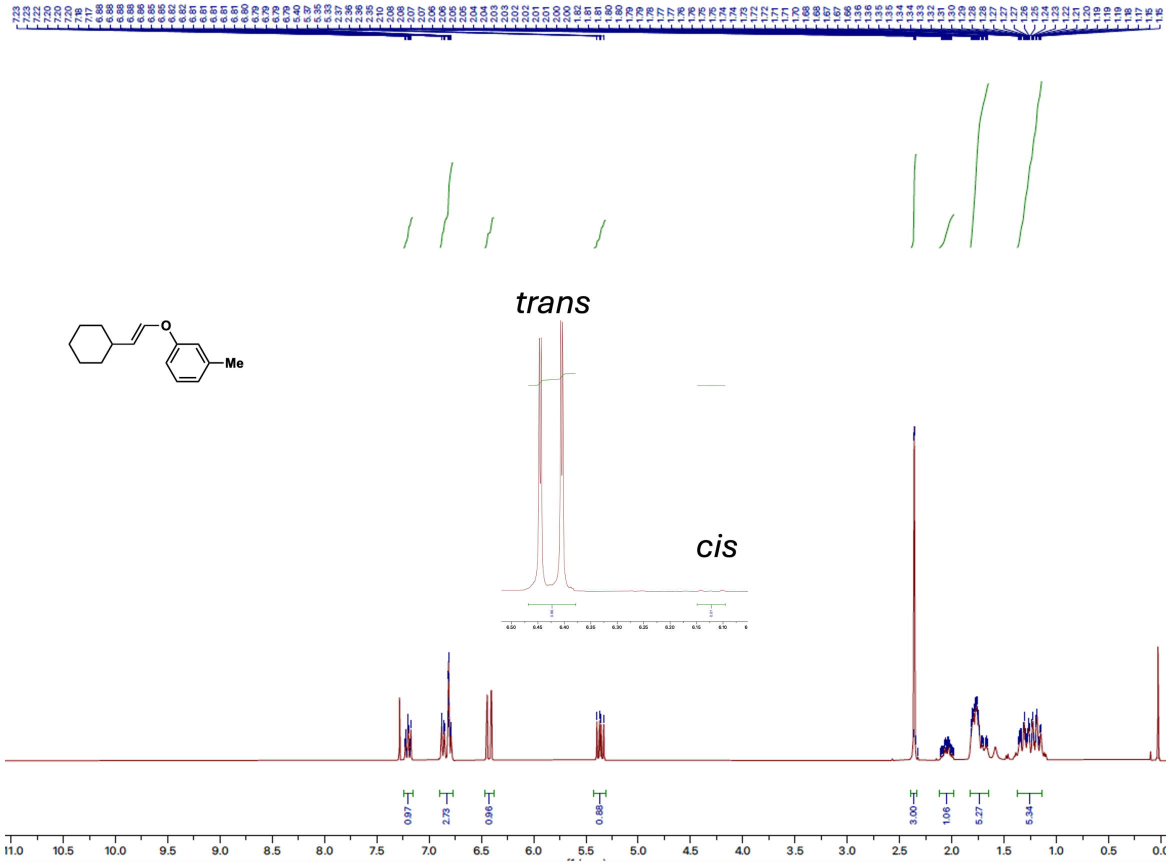

^{13}C-NMR (101 MHz, $CDCl_3$) of compound **9**

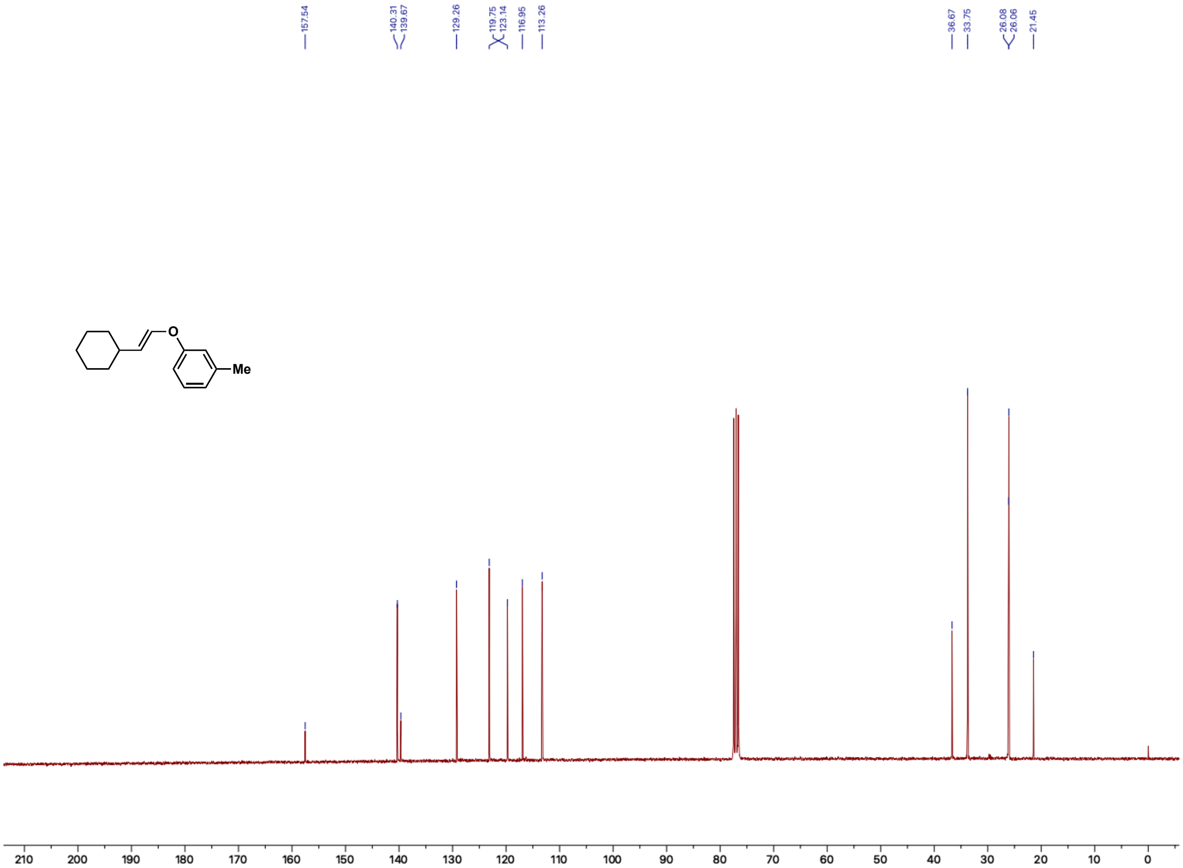

^{1}H-NMR (300 MHz, $CDCl_3$) of compound **10** (mixed with alkane product, E/Z ratio was determined using GCMS and ^{31}P-NMR)

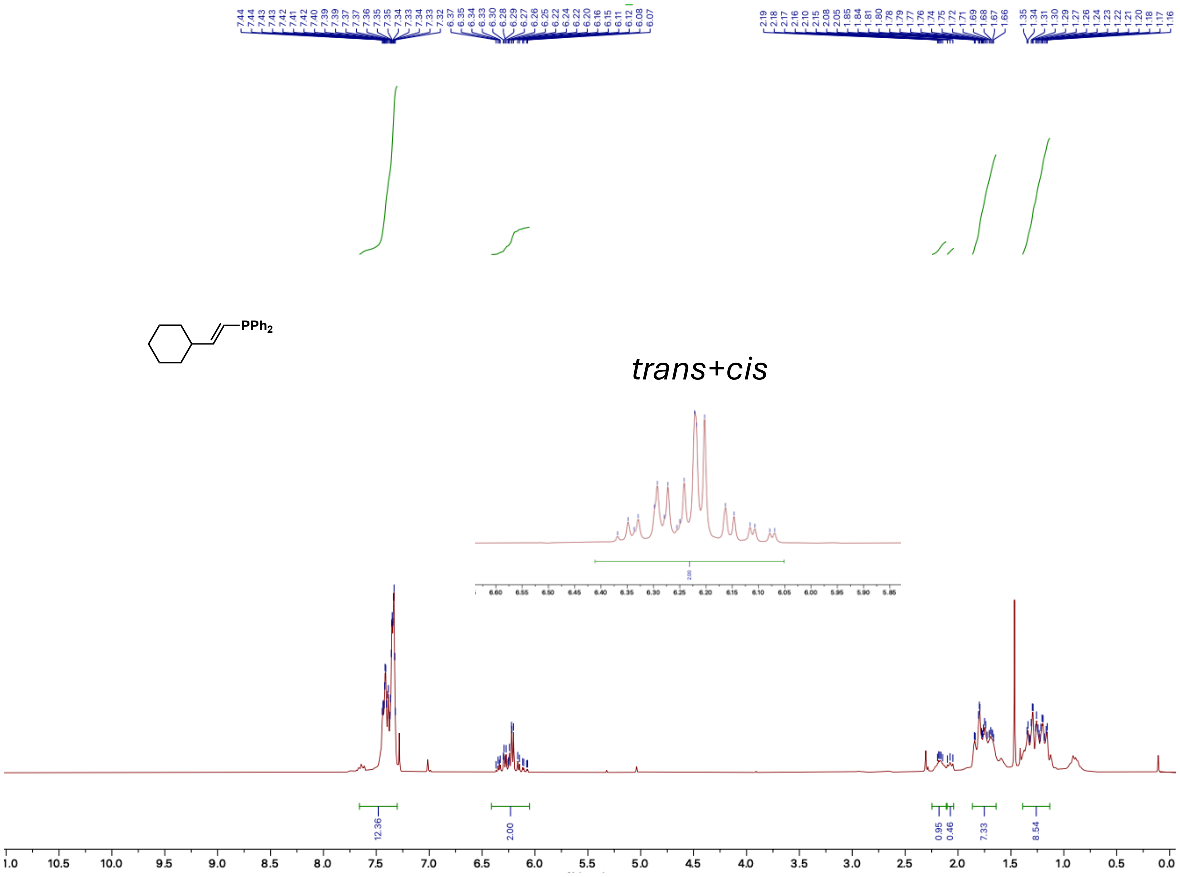

^{13}C-NMR (75 MHz, $CDCl_3$) of compound **10** (mixed with alkane product)

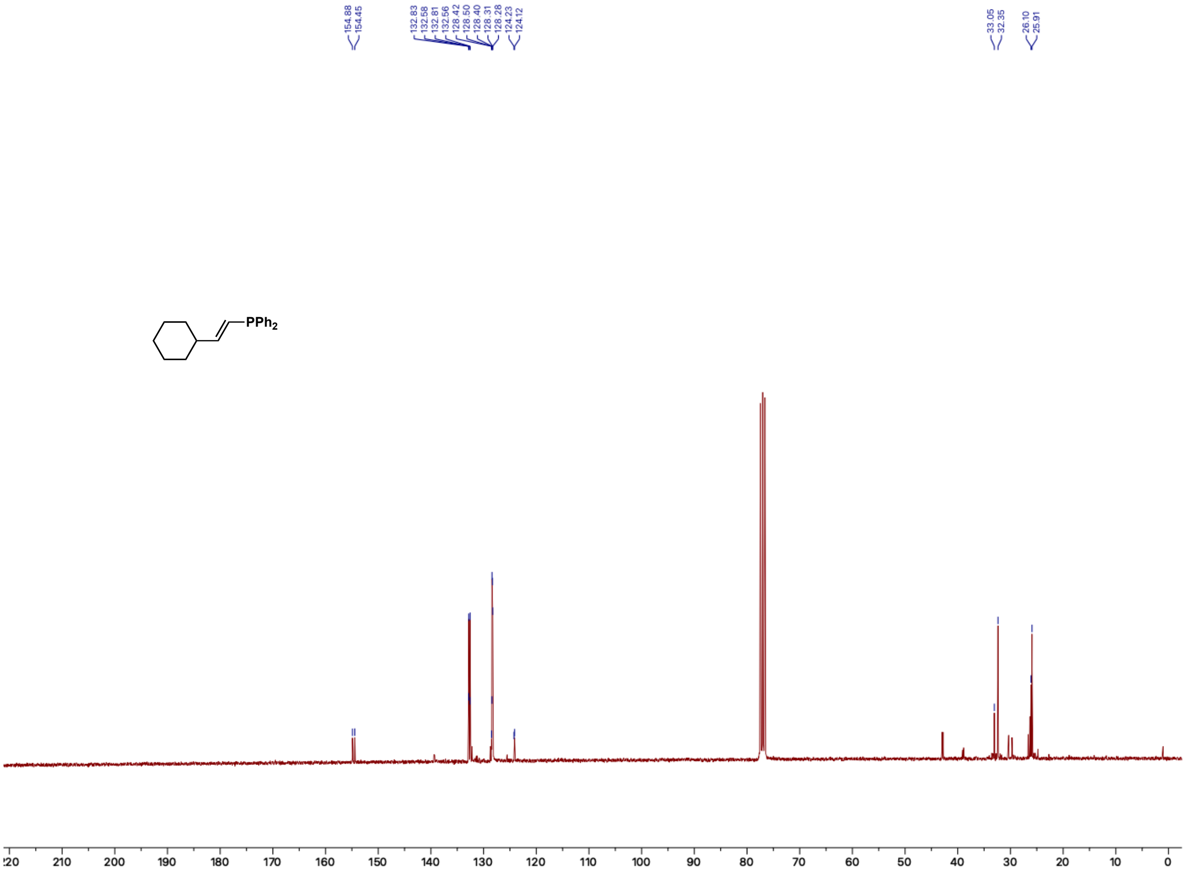

^{31}P-NMR (121 MHz, $CDCl_3$) of compound **10** (* alkane product)

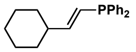

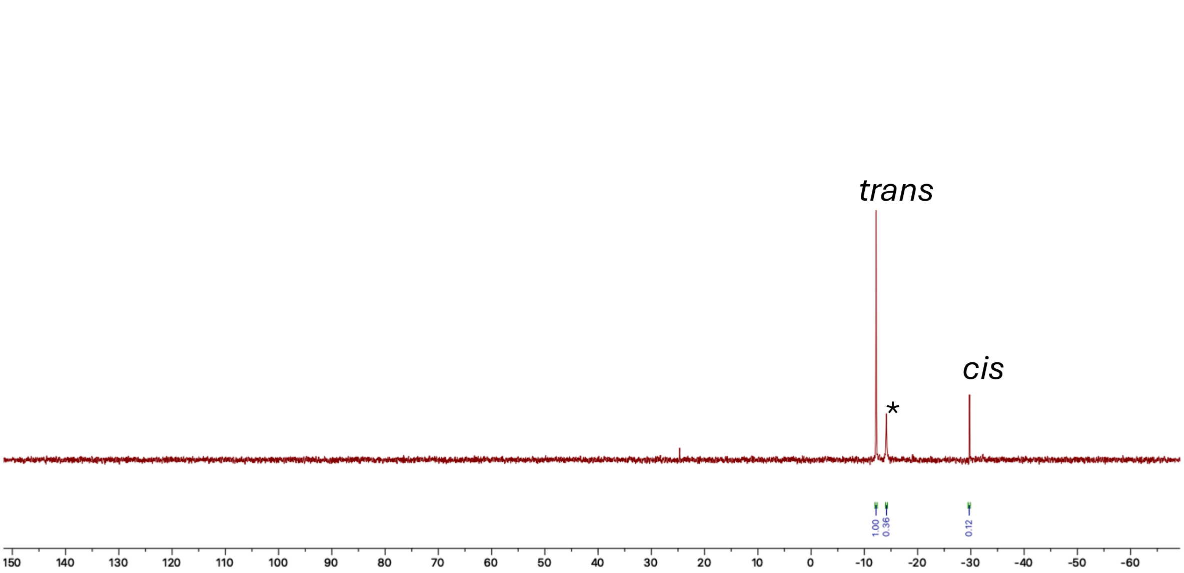

^{1}H-NMR (300 MHz, $CDCl_3$) of compound **11**

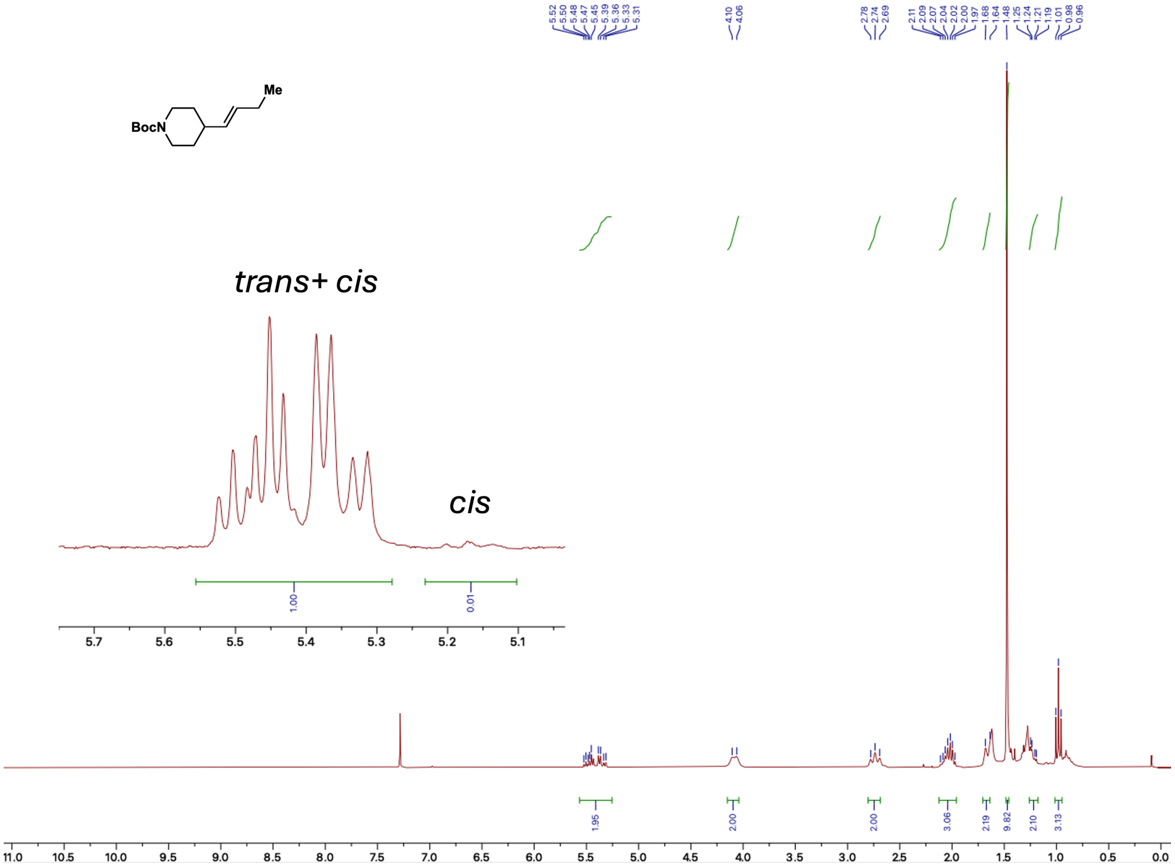

^{13}C-NMR (101 MHz, CDCl$_3$) of compound **11**

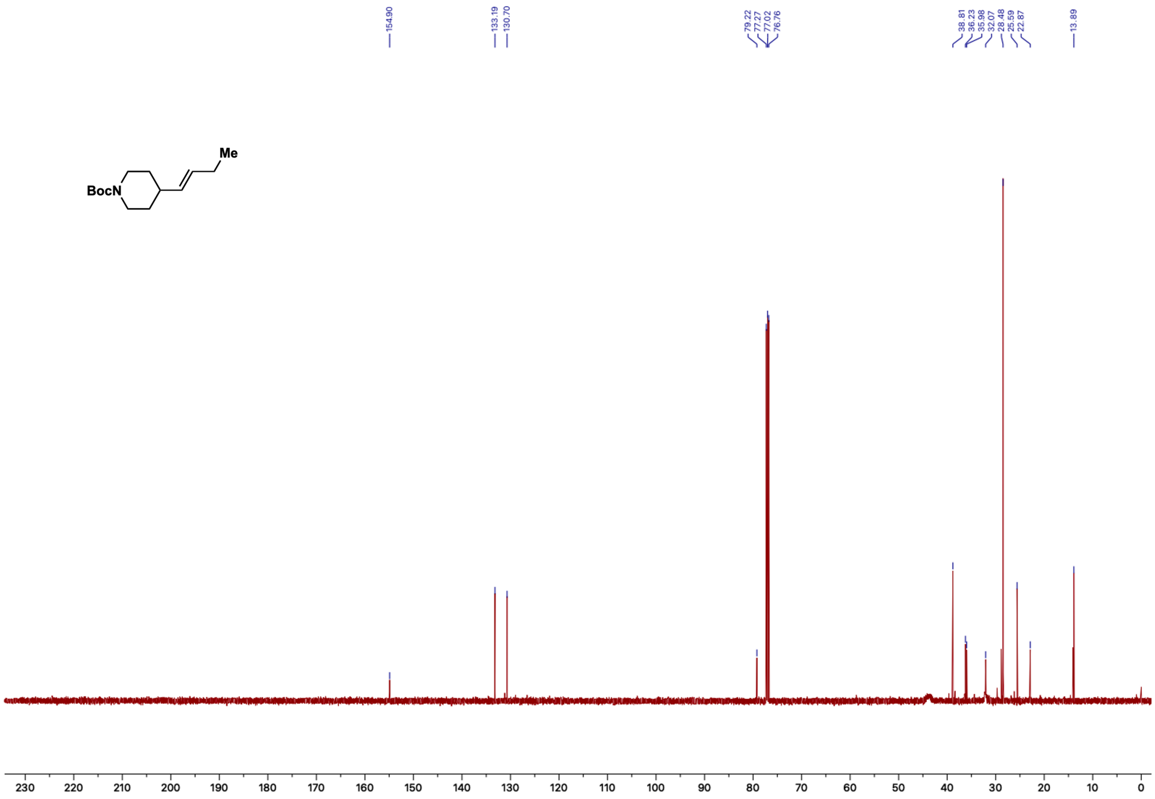

^{1}H-NMR (800 MHz, $CDCl_3$) of compound **12**

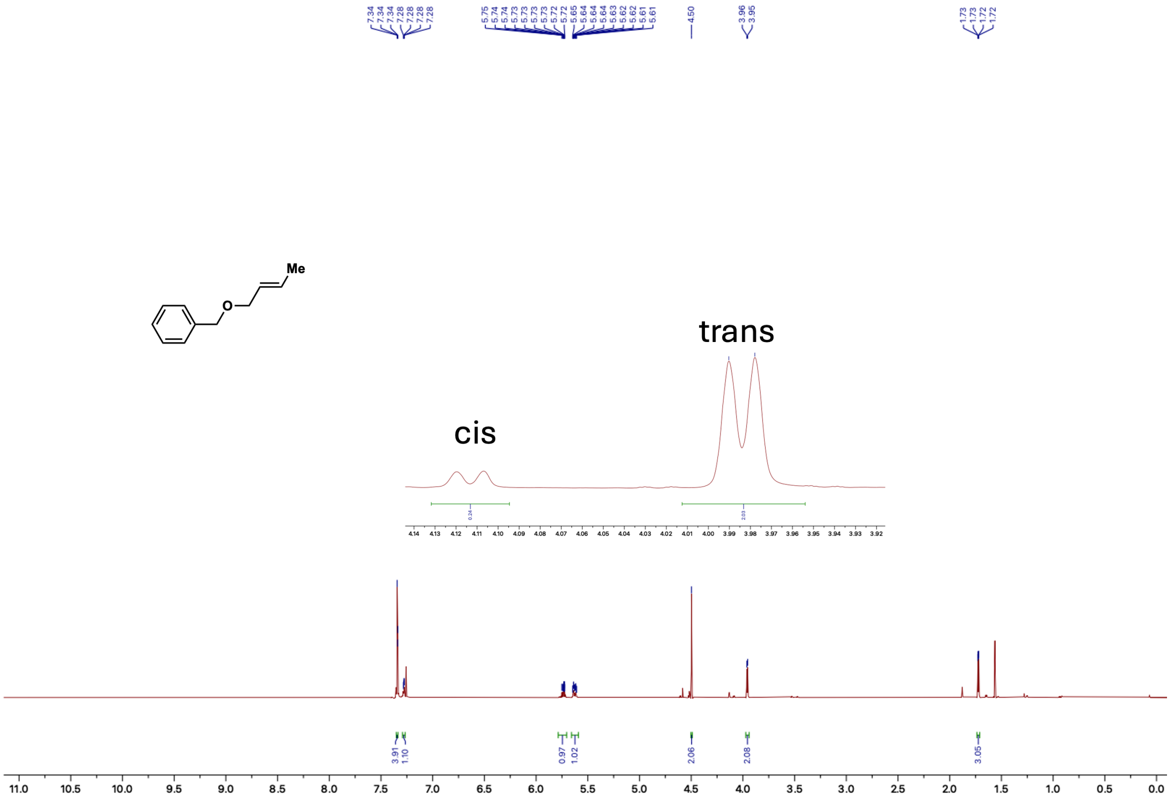

^{13}C-NMR (201 MHz, CDCl$_3$) of compound **12**

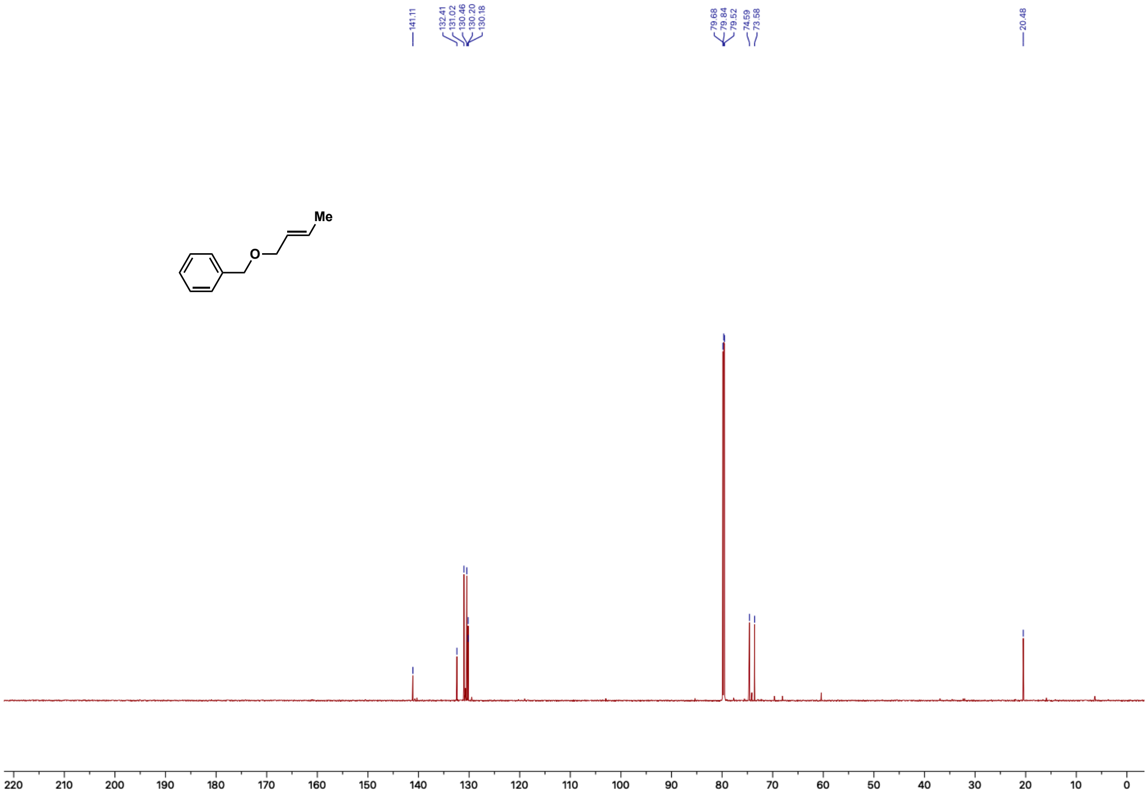

^{1}H-NMR (300 MHz, $CDCl_3$) of compound **13** (* alkane product)

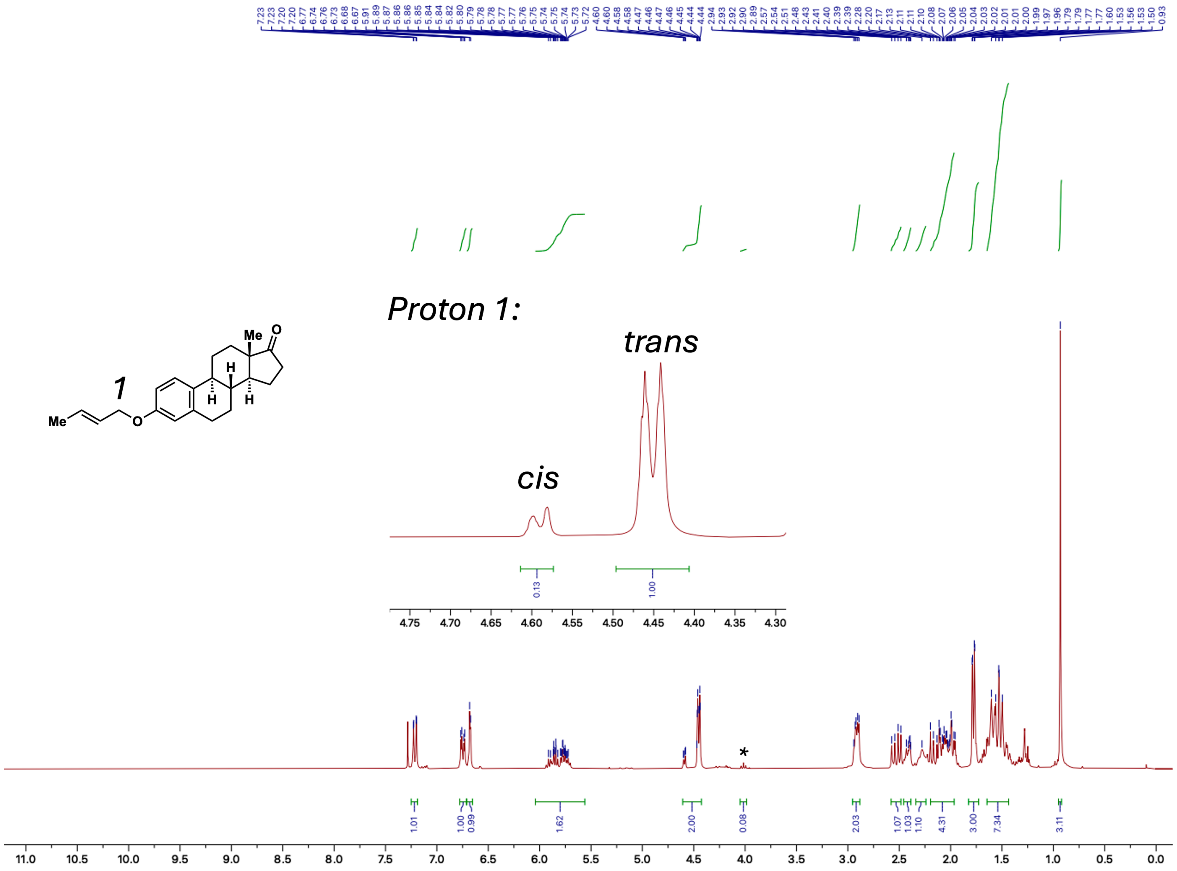

^{13}C-NMR (75 MHz, $CDCl_3$) of compound **13**

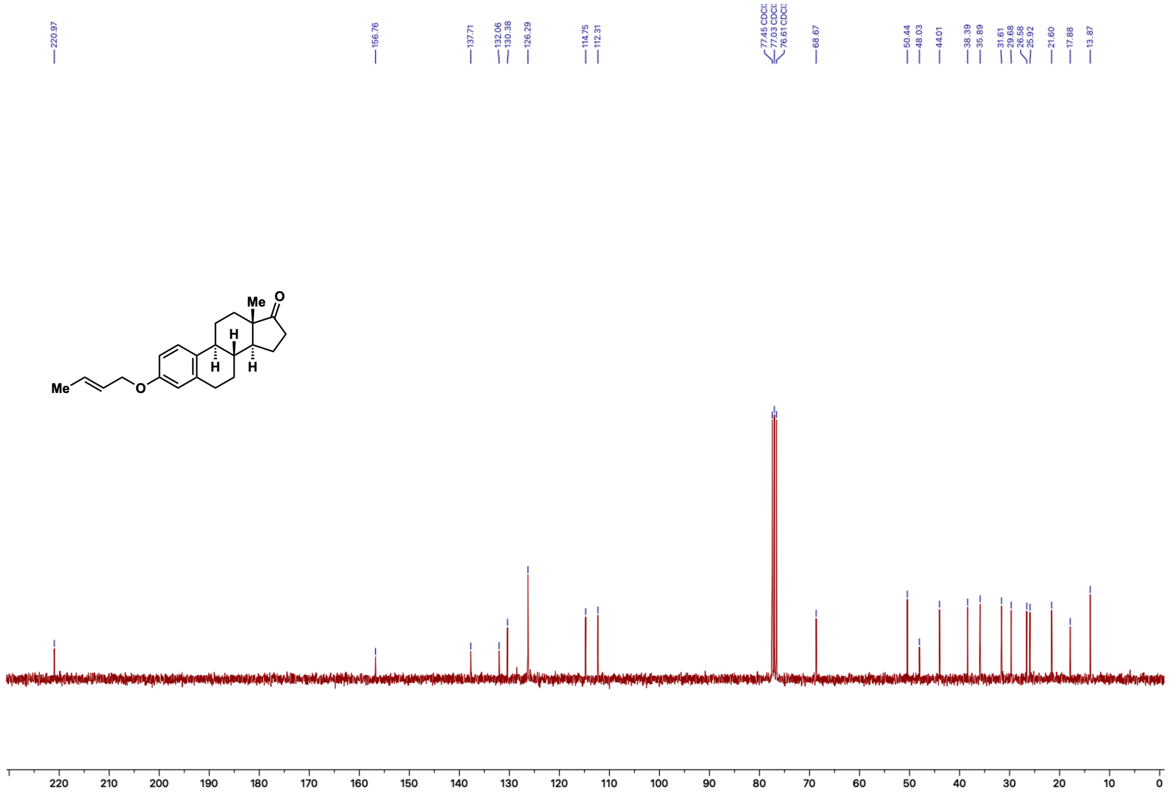

^{1}H-NMR (300 MHz, $CDCl_3$) of compound **14**

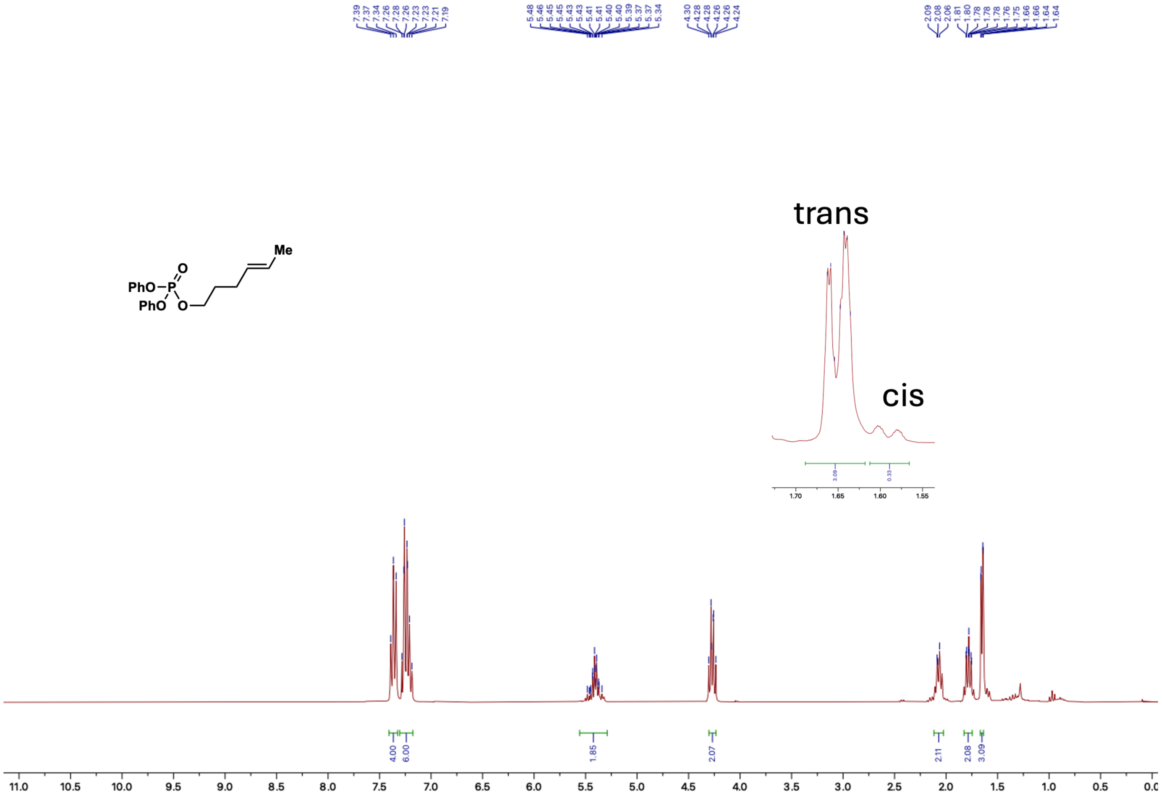

^{13}C-NMR (101 MHz, $CDCl_3$) of compound **14**

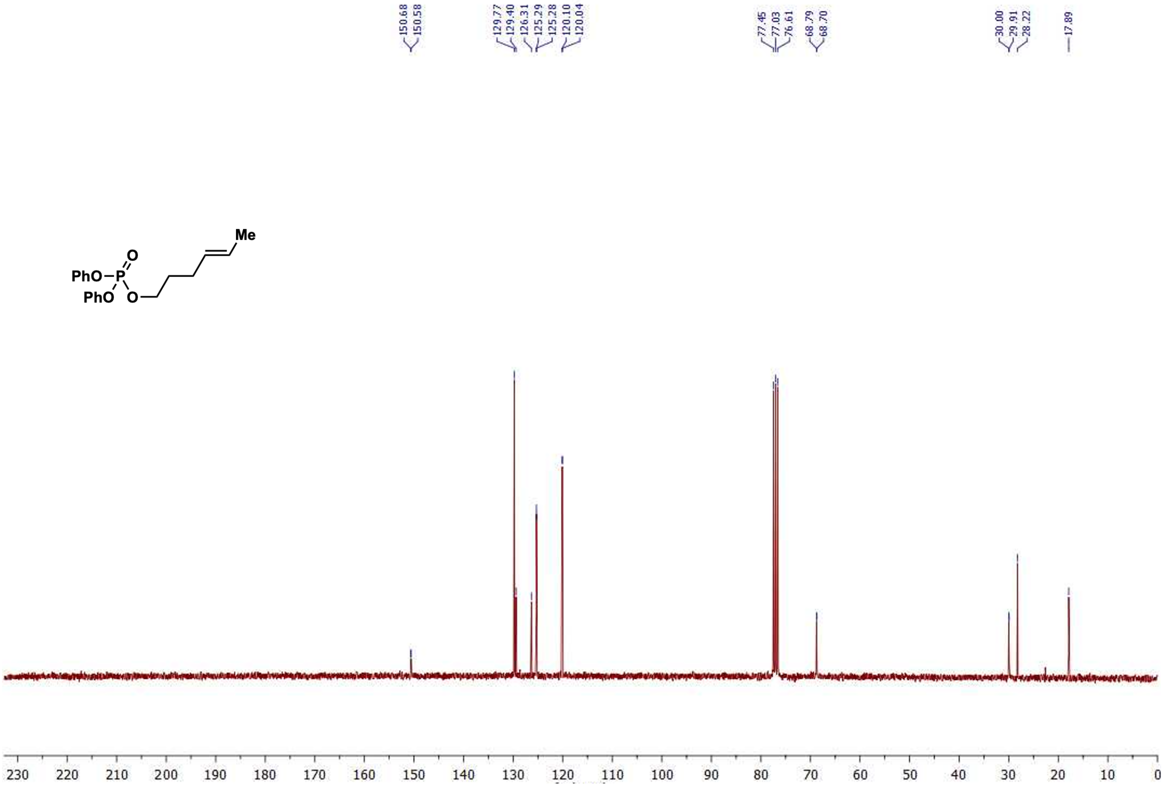

^{31}P-NMR (101 MHz, $CDCl_3$) of compound **14**

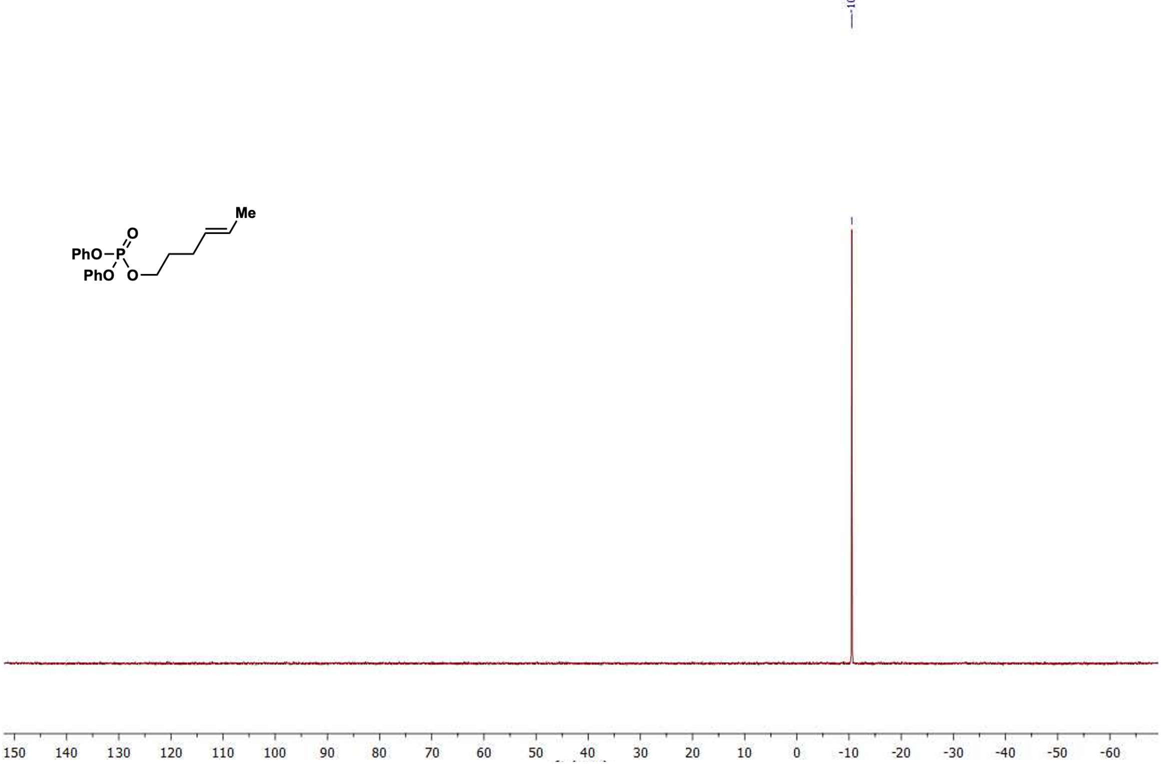

^{1}H-NMR (400 MHz, $CDCl_3$) of compound **15** (ratio determined using GC-MS)

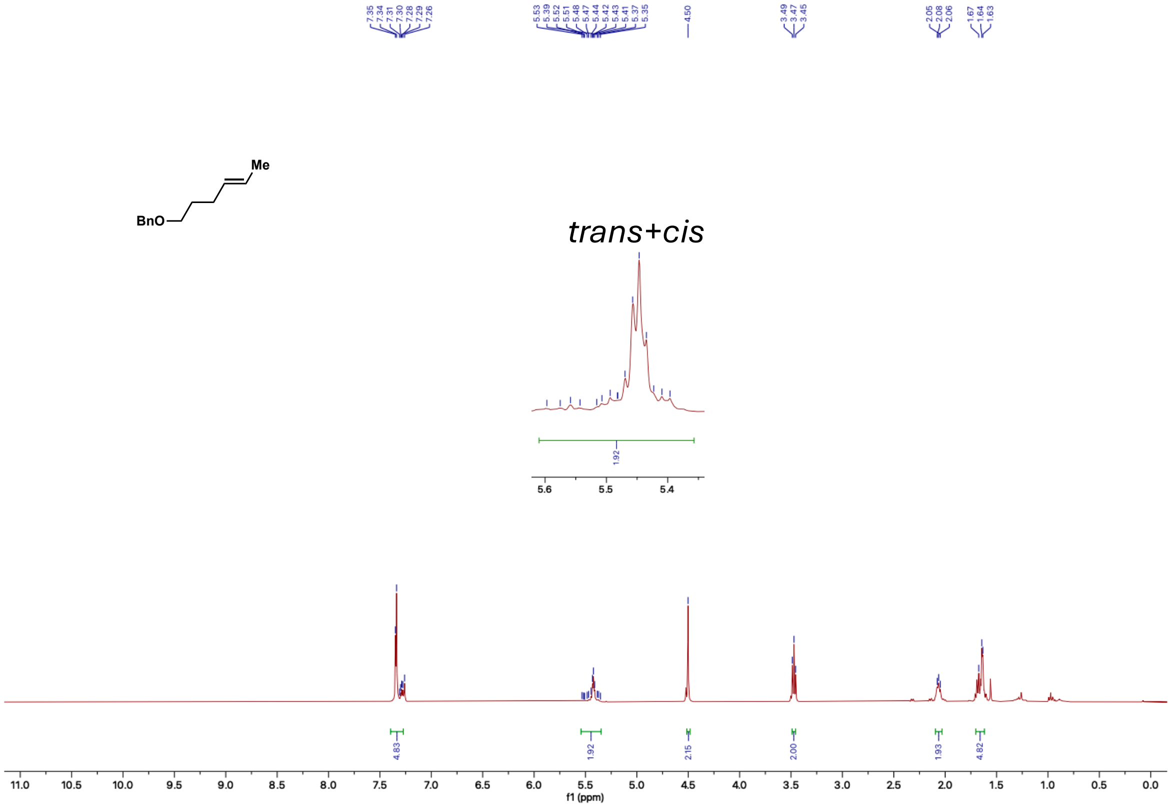

^{13}C-NMR (75 MHz, $CDCl_3$) of compound **15**

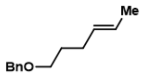

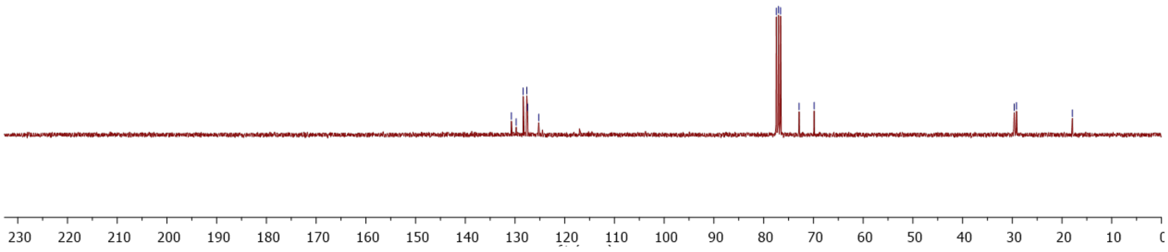

^{1}H-NMR (500 MHz, $CDCl_3$) of compound **16** (mixed with alkane product)

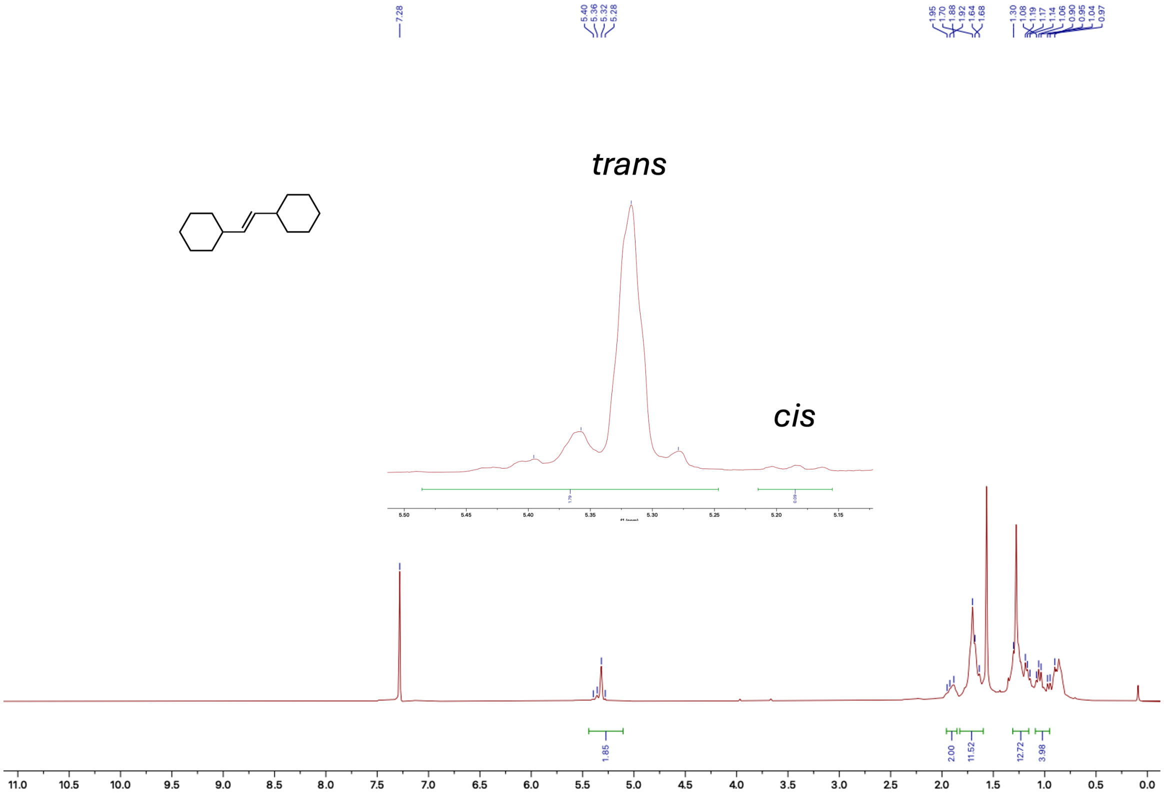

^{13}C-NMR (75 MHz, $CDCl_3$) of compound **16** (mixed with alkane product)

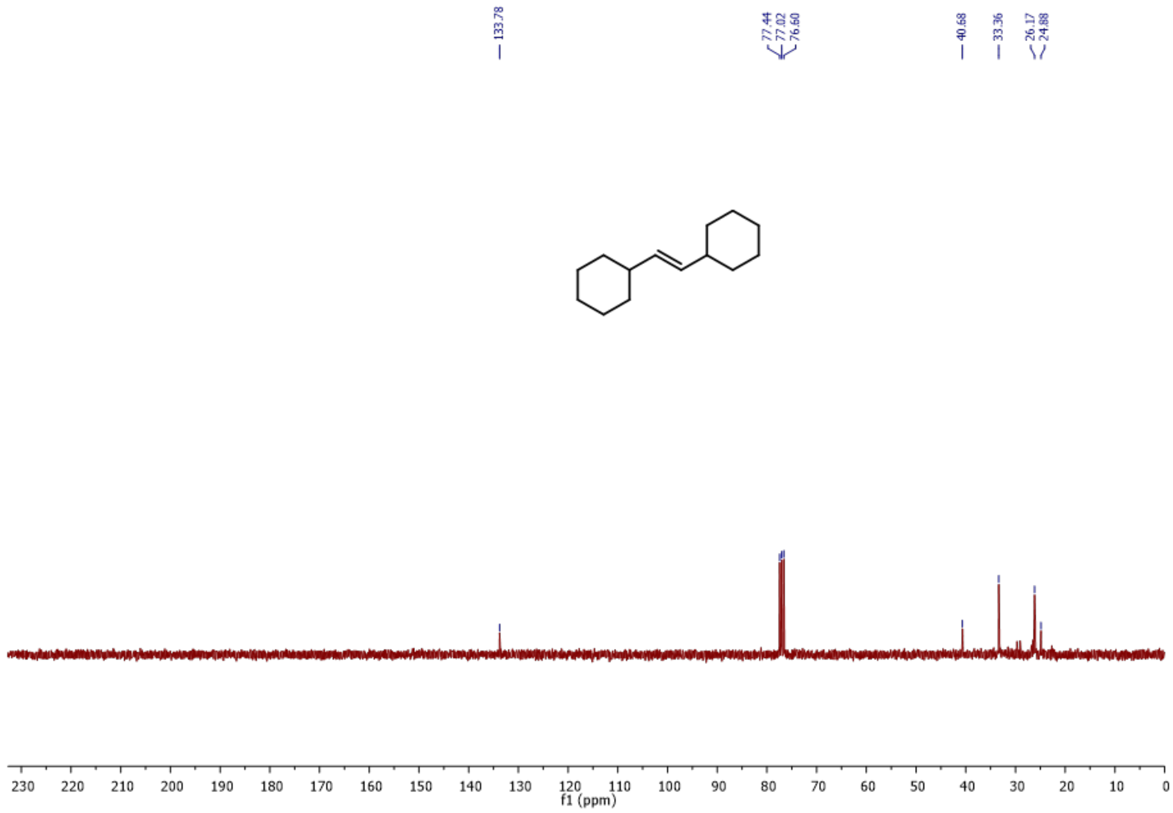

^{1}H-NMR (400 MHz, $CDCl_3$) of compound **17** (* alkane product)

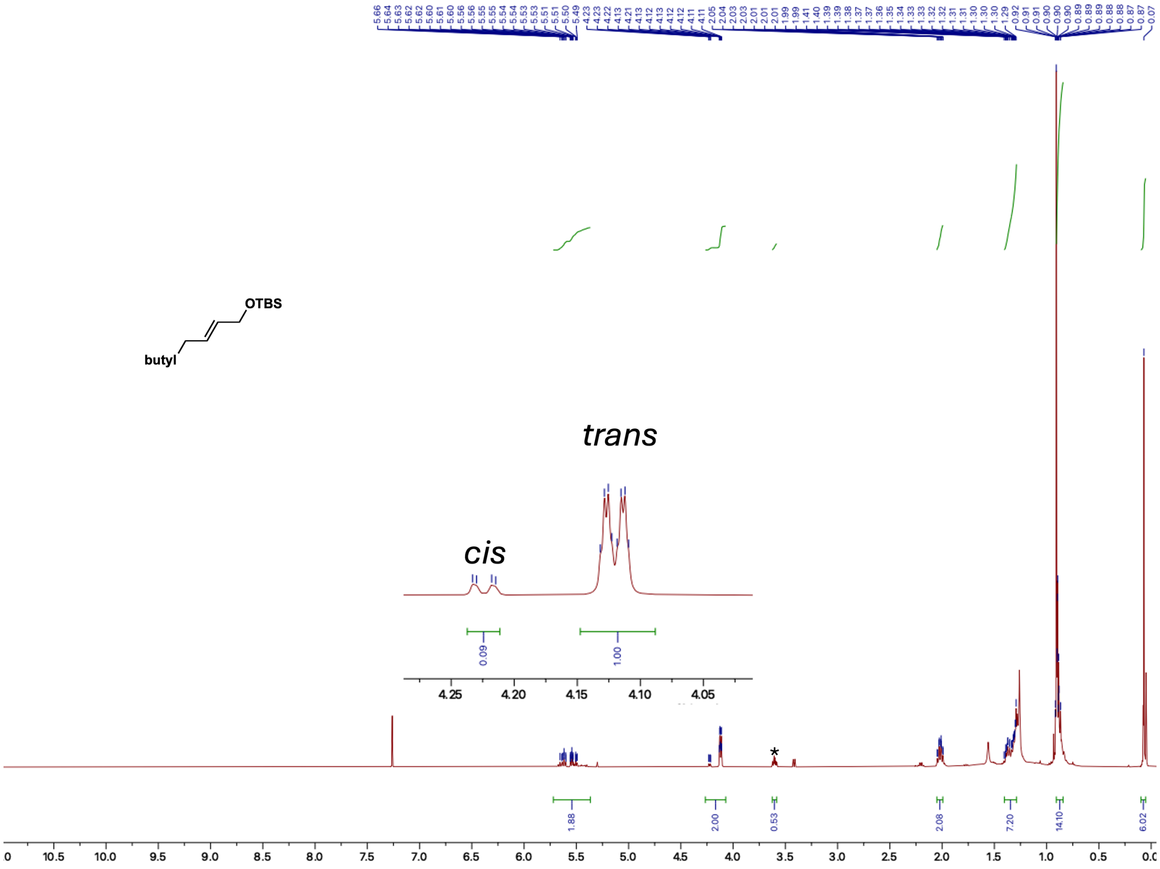

^{13}C-NMR (101 MHz, $CDCl_3$) of compound **17** (*mixed with alkane product*)

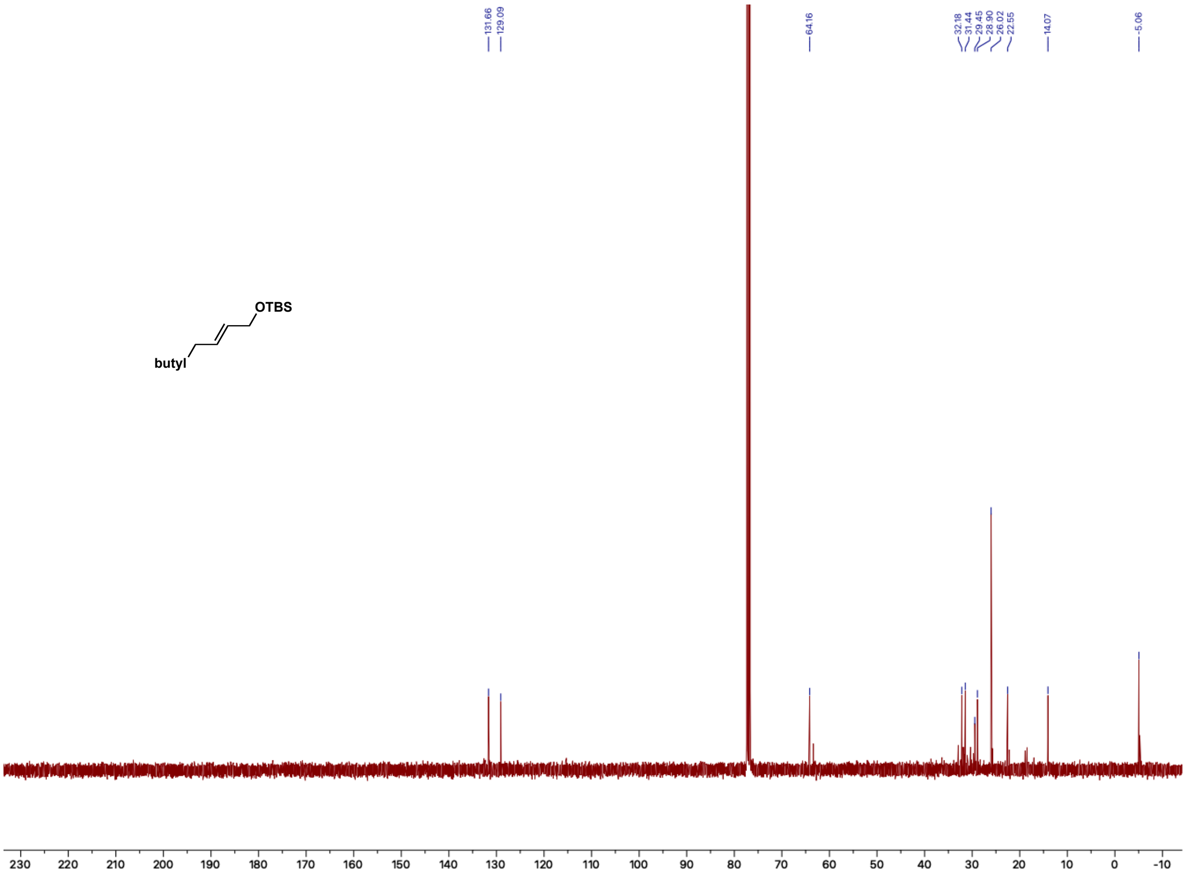

^{1}H-NMR (300 MHz, $CDCl_3$) of compound **18**

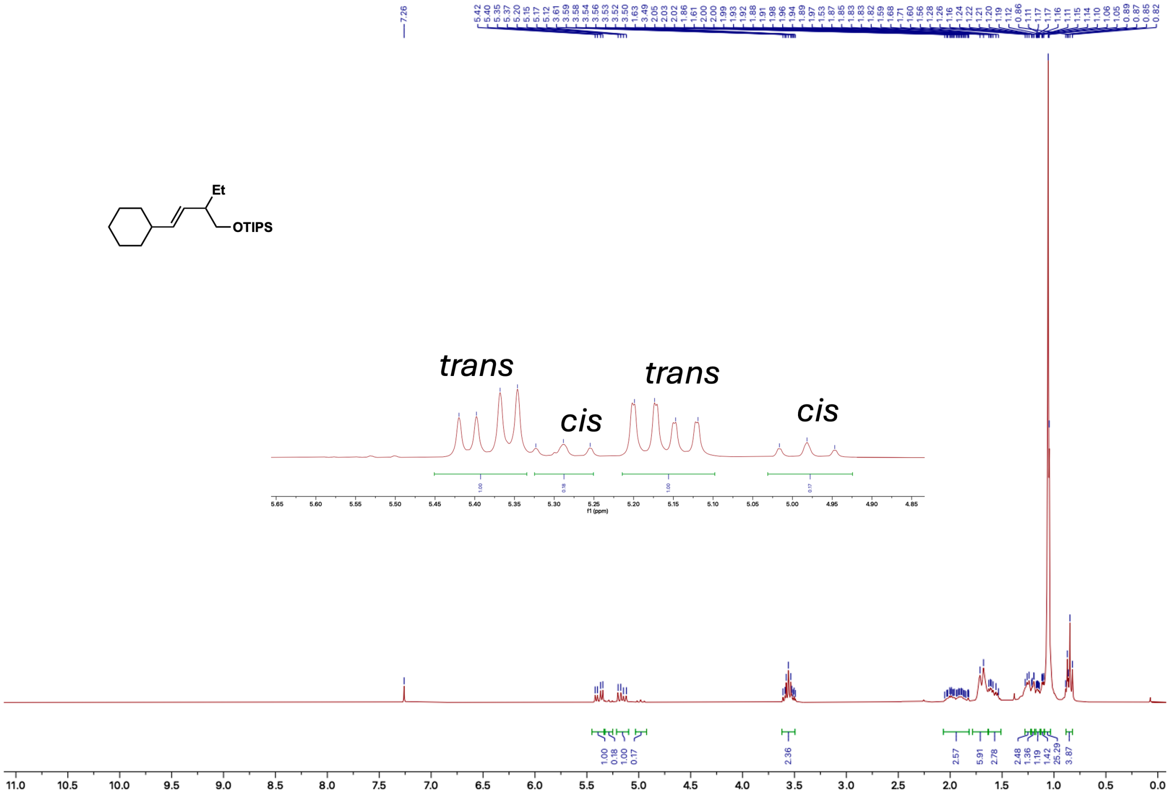

^{13}C-NMR (75 MHz, $CDCl_3$) of compound **18**

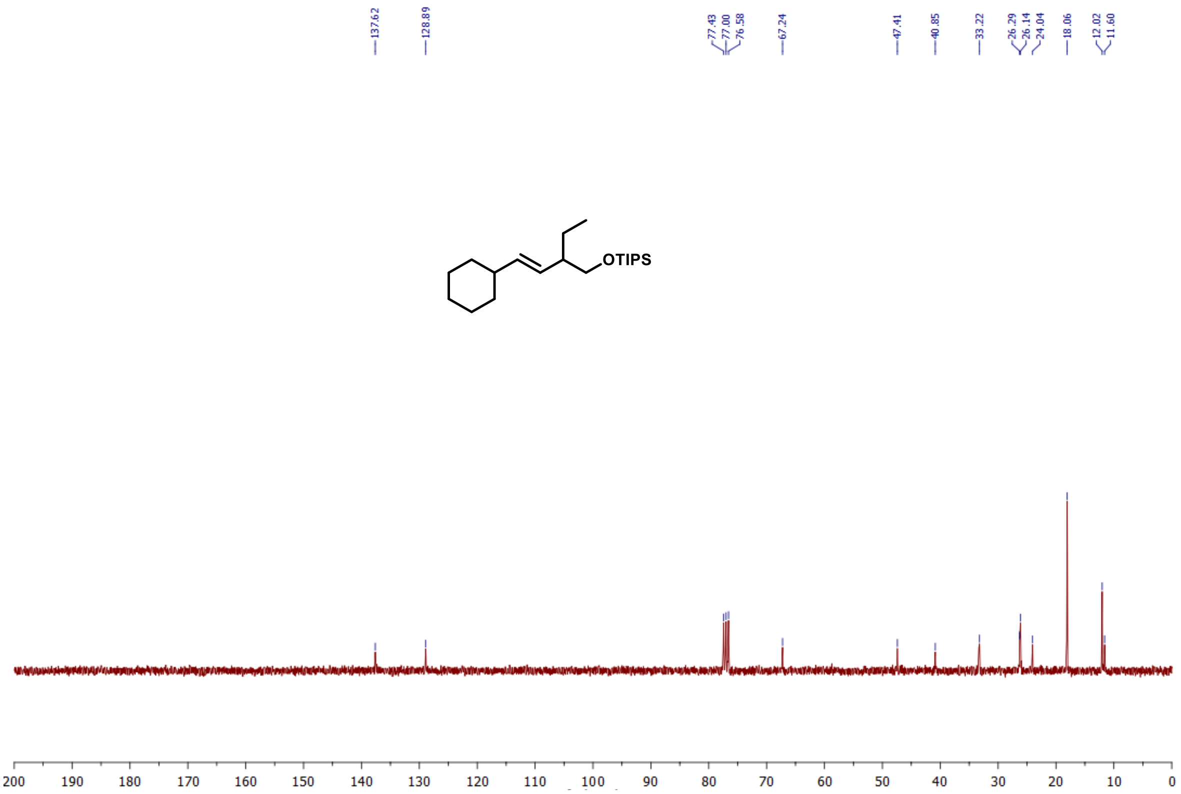

^{1}H-NMR (300 MHz, $CDCl_3$) of compound **19**

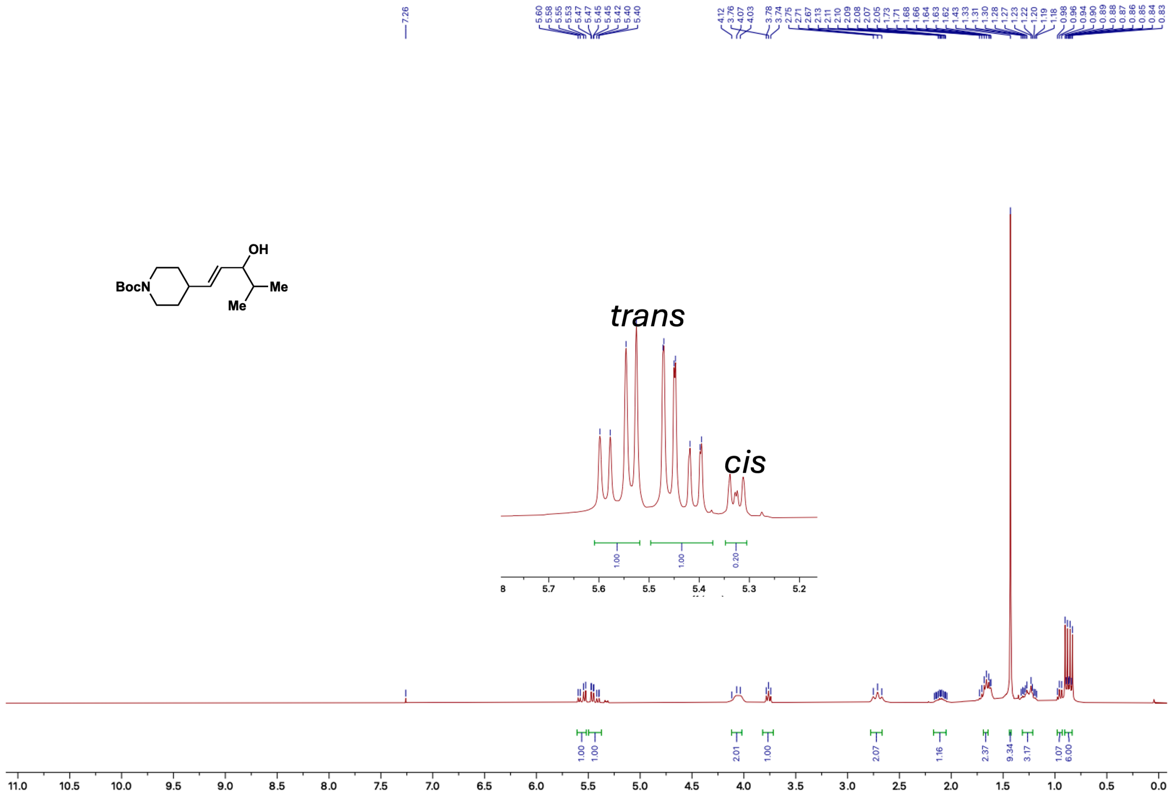

^{13}C-NMR (75 MHz, $CDCl_3$) of compound **19**

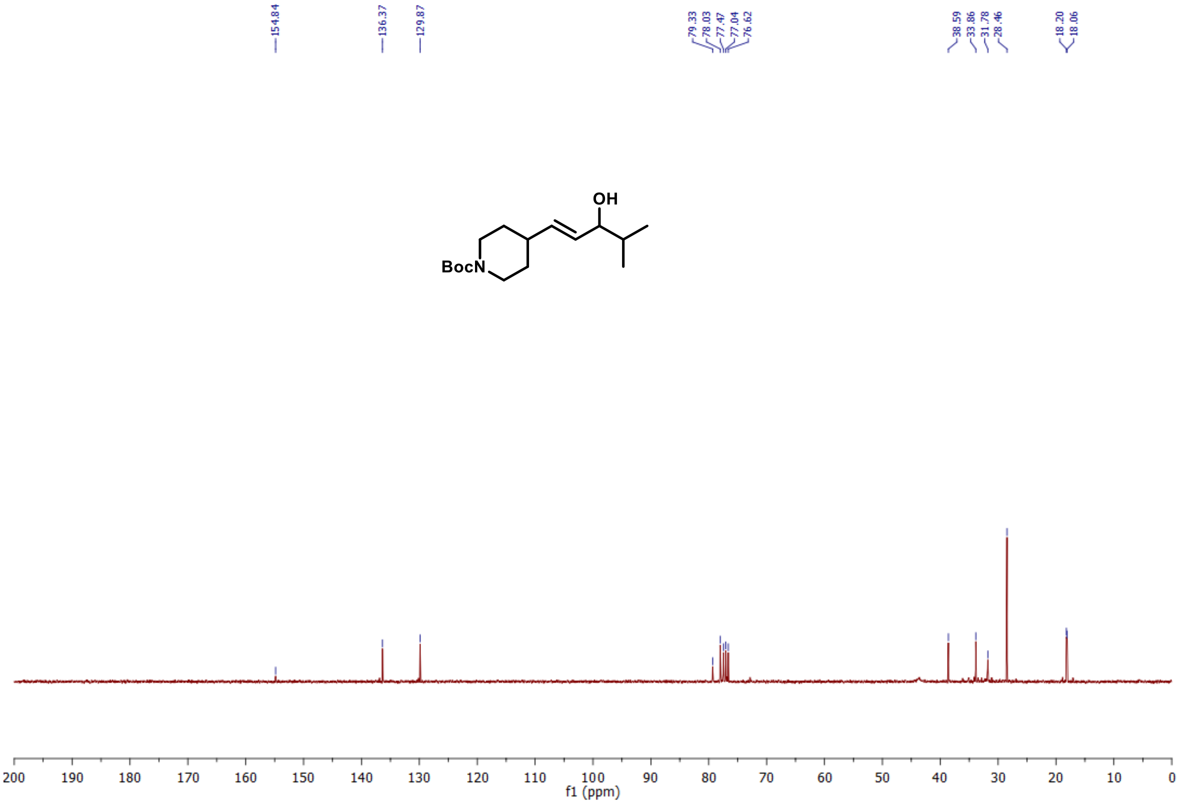

^{1}H-NMR (300 MHz, $CDCl_3$) of compound **20**

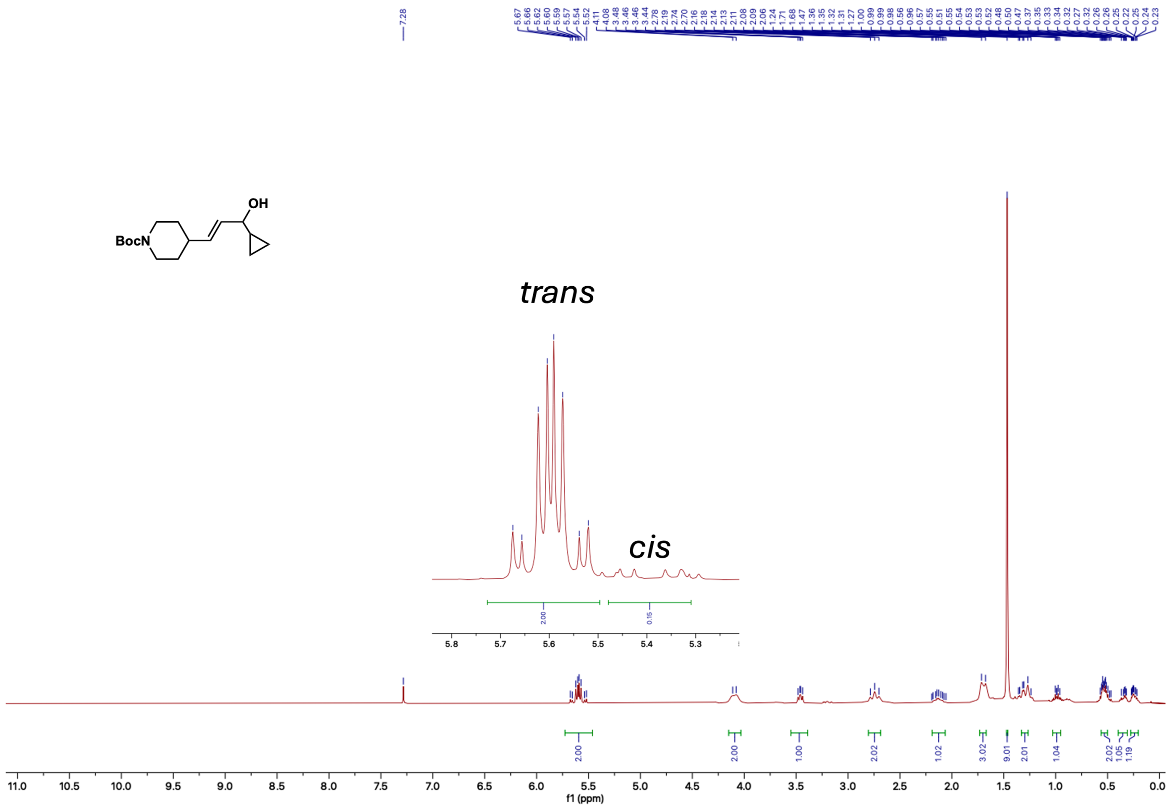

^{13}C-NMR (126 MHz, $CDCl_3$) of compound **20**

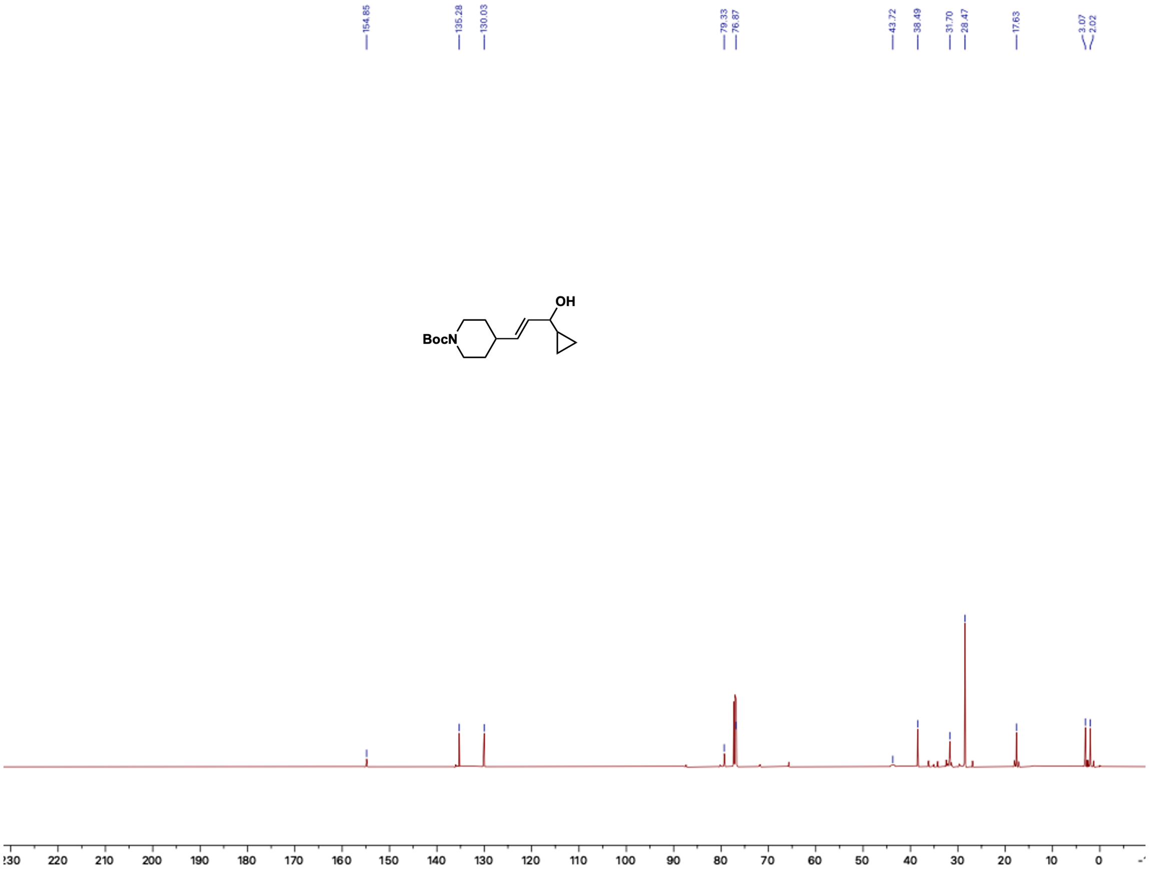

^{1}H-NMR (400 MHz, $CDCl_3$) of compound **21** (* residue of alkyne)

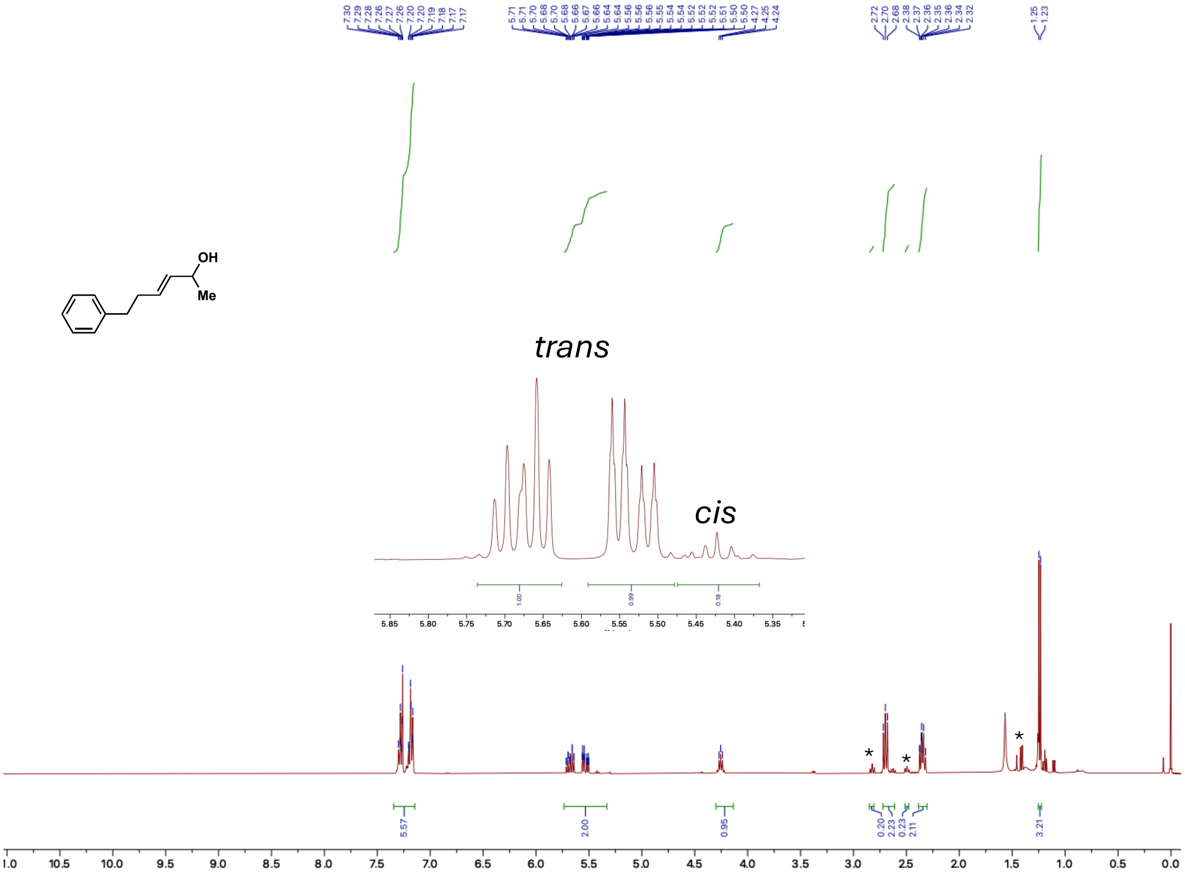

^{13}C-NMR (126 MHz, $CDCl_3$) of compound **21**

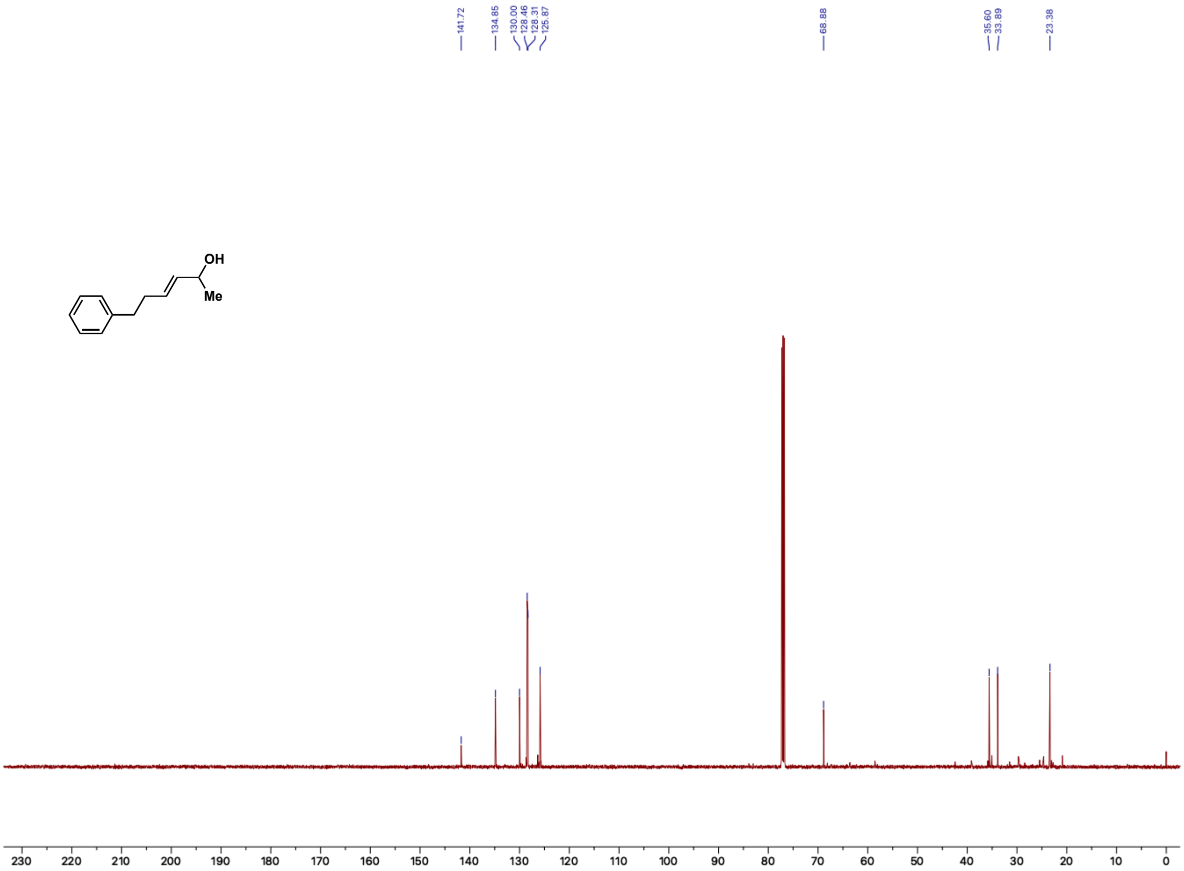

^{1}H-NMR (400 MHz, $CDCl_3$) of compound **22**

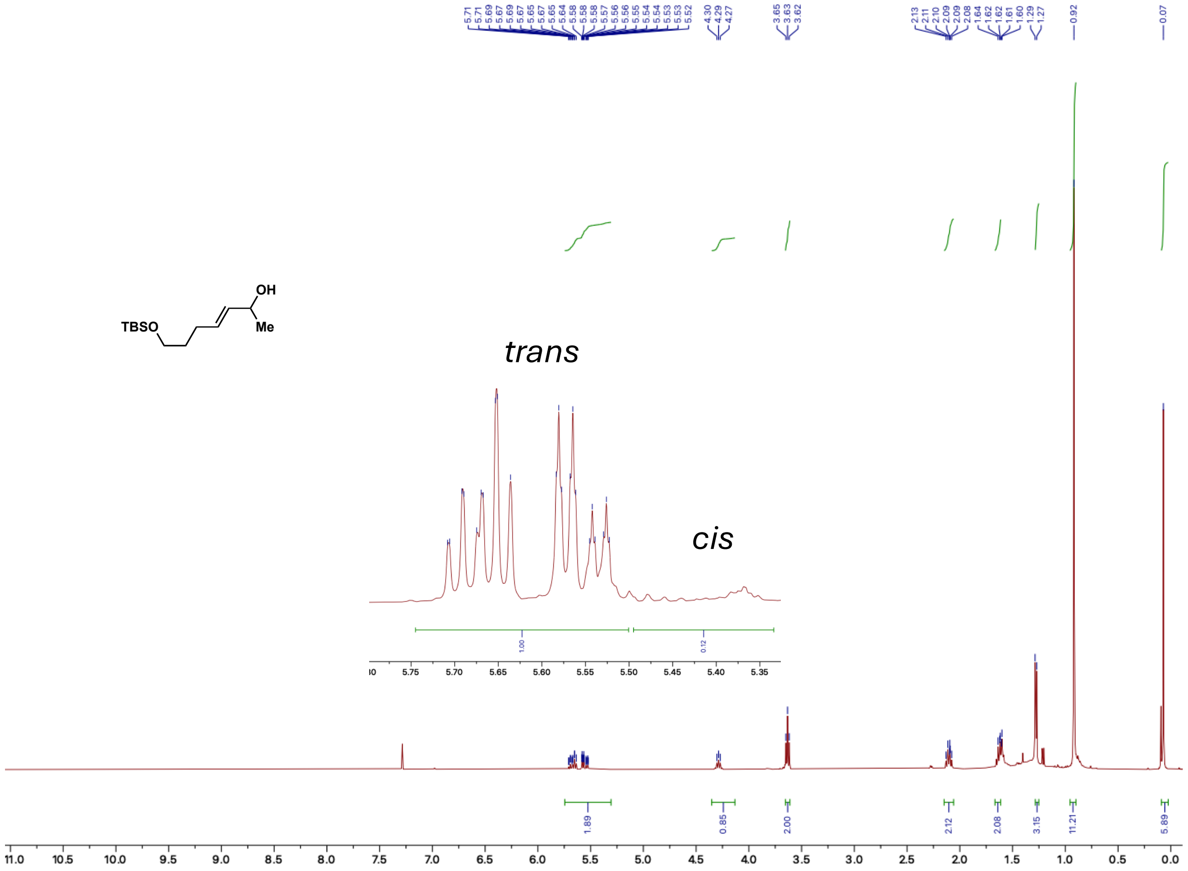

^{13}C-NMR (201 MHz, $CDCl_3$) of compound **22**

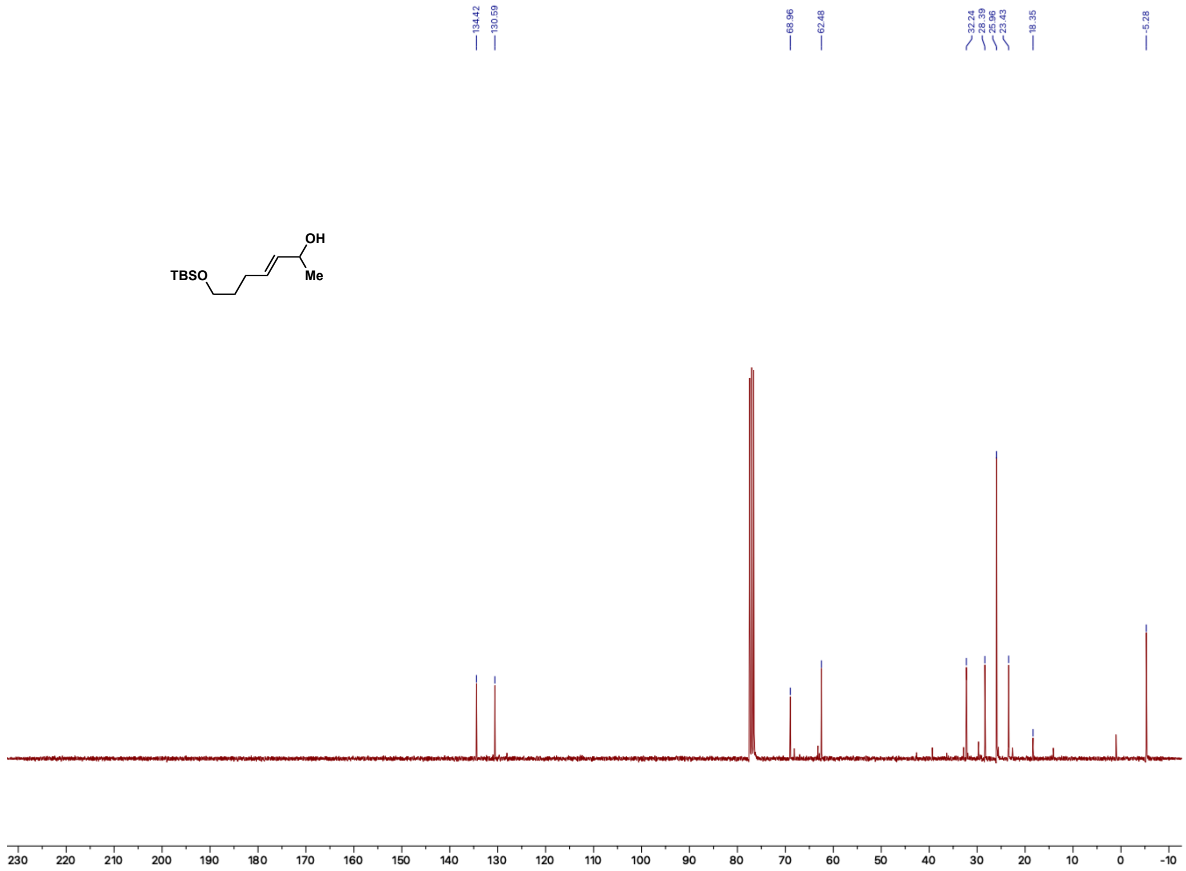

^{1}H-NMR (400 MHz, $CDCl_3$) of compound **23**

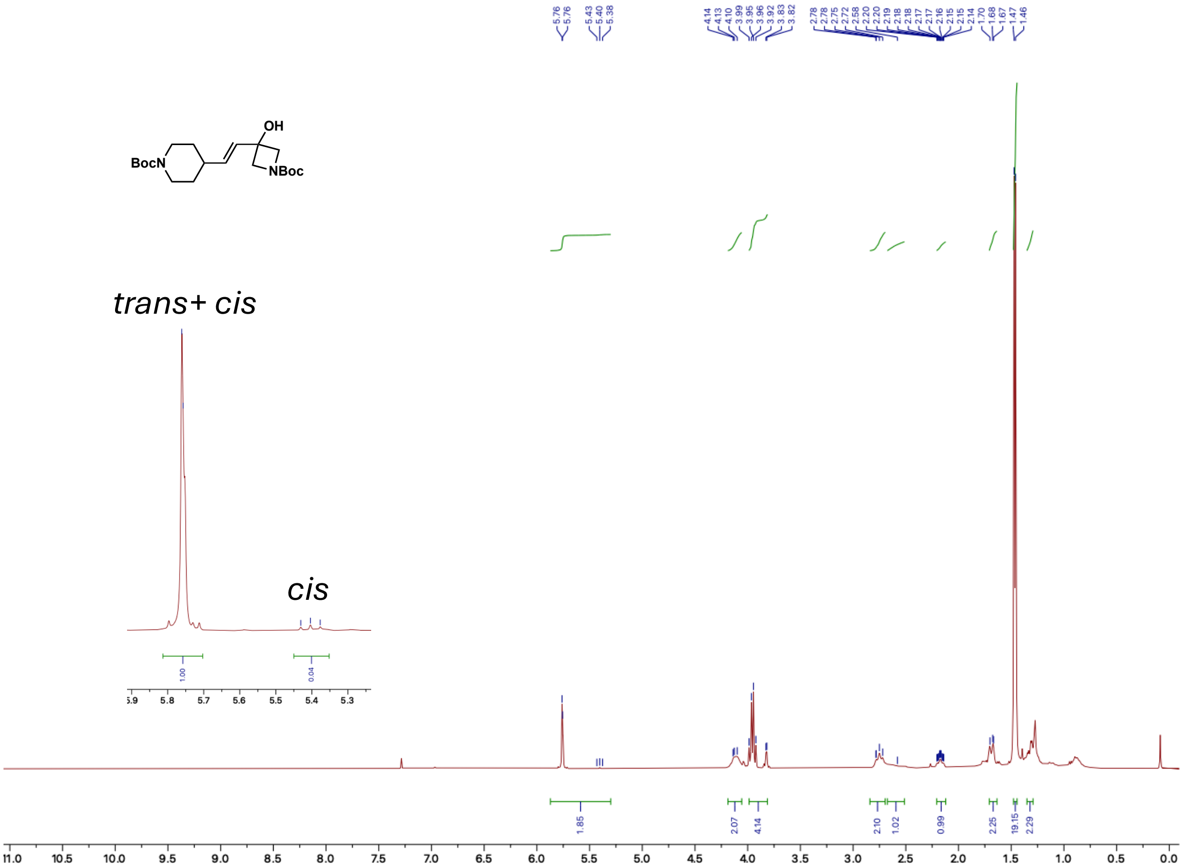

^{13}C-NMR (126 MHz, $CDCl_3$) of compound **23**

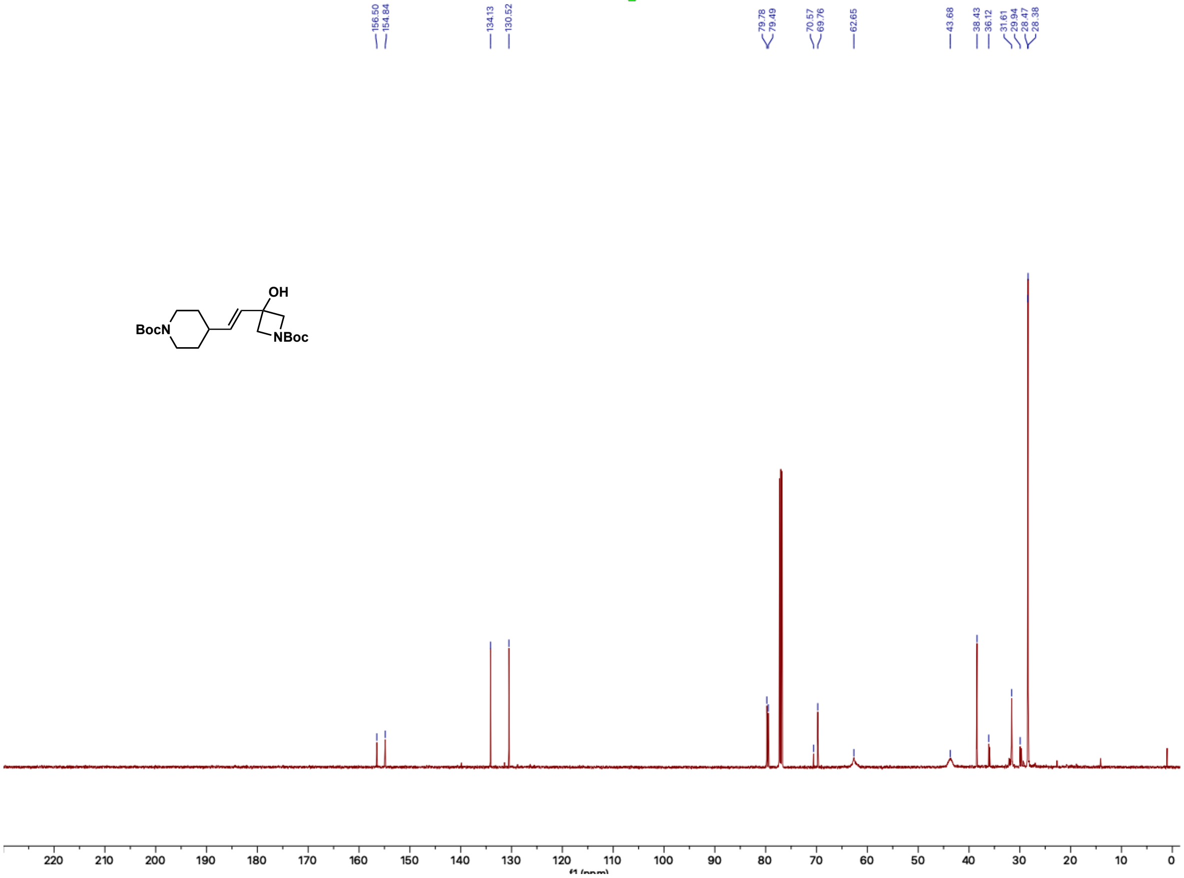

^{1}H-NMR (400 MHz, $CDCl_3$) of compound **24**

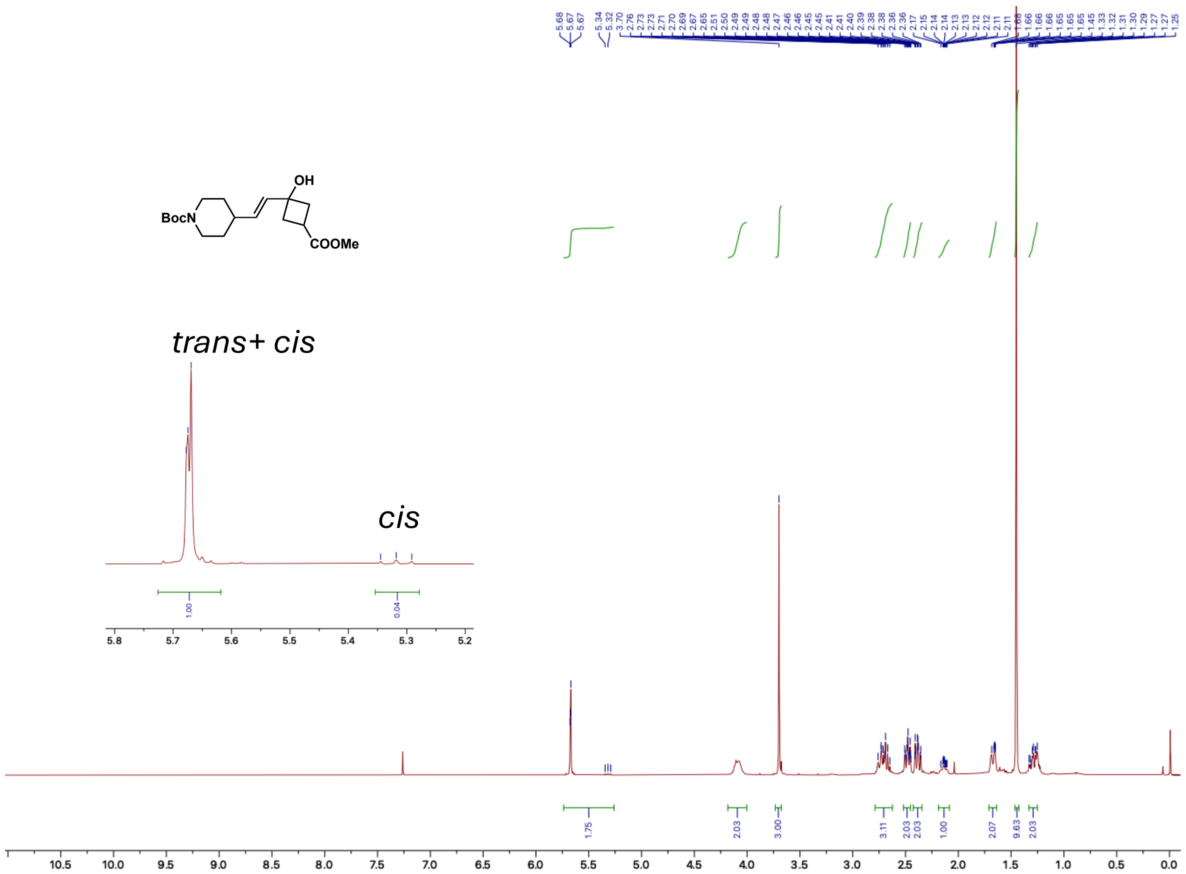

^{13}C-NMR (101 MHz, $CDCl_3$) of compound **24**

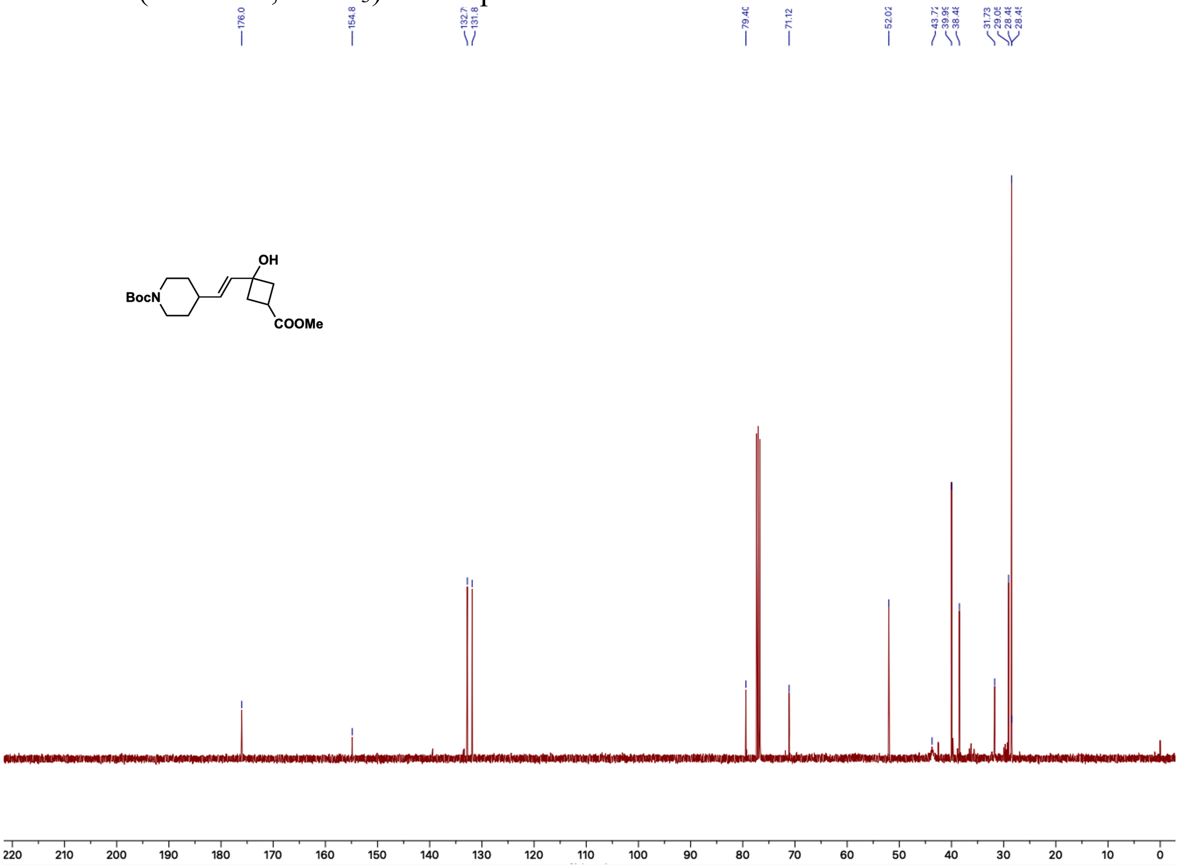

^{1}H-NMR (300 MHz, $CDCl_3$) of compound **25**

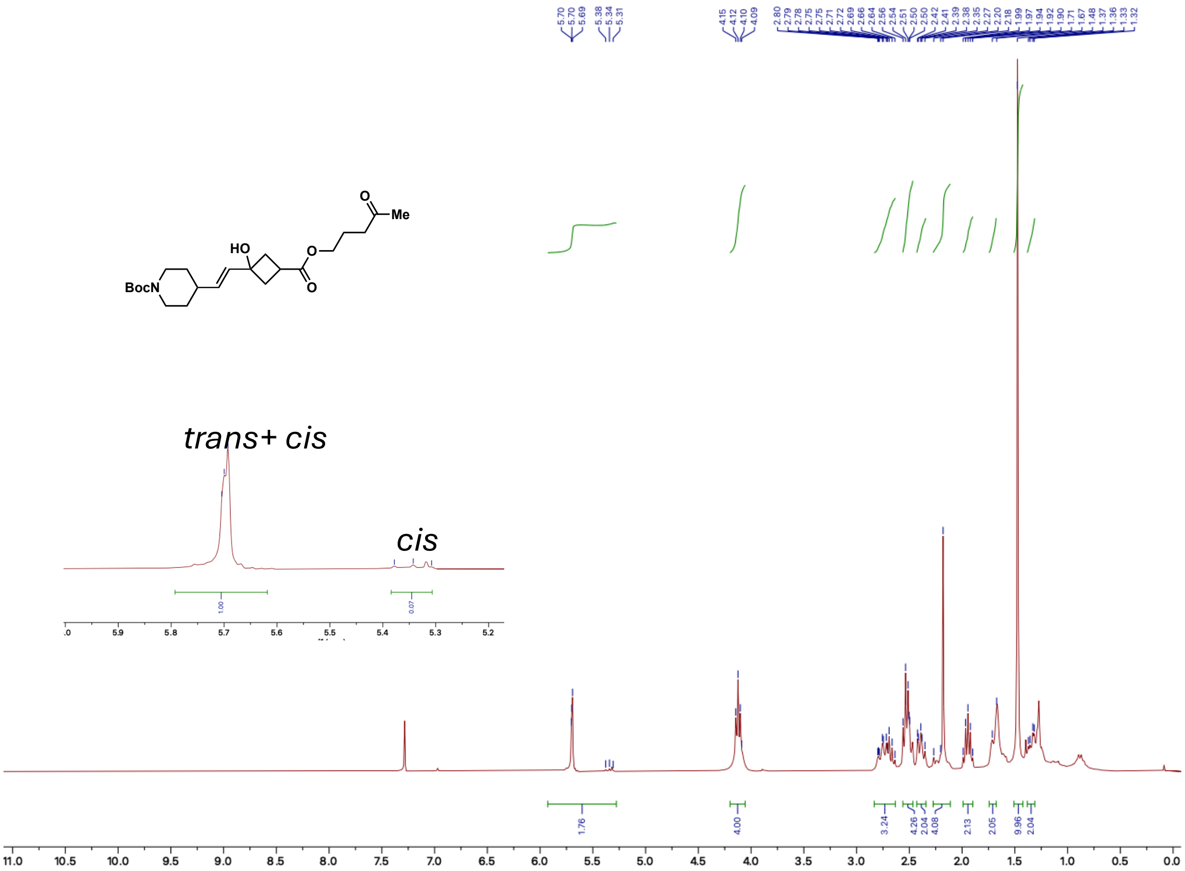

^{13}C-NMR (101 MHz, $CDCl_3$) of compound **25**

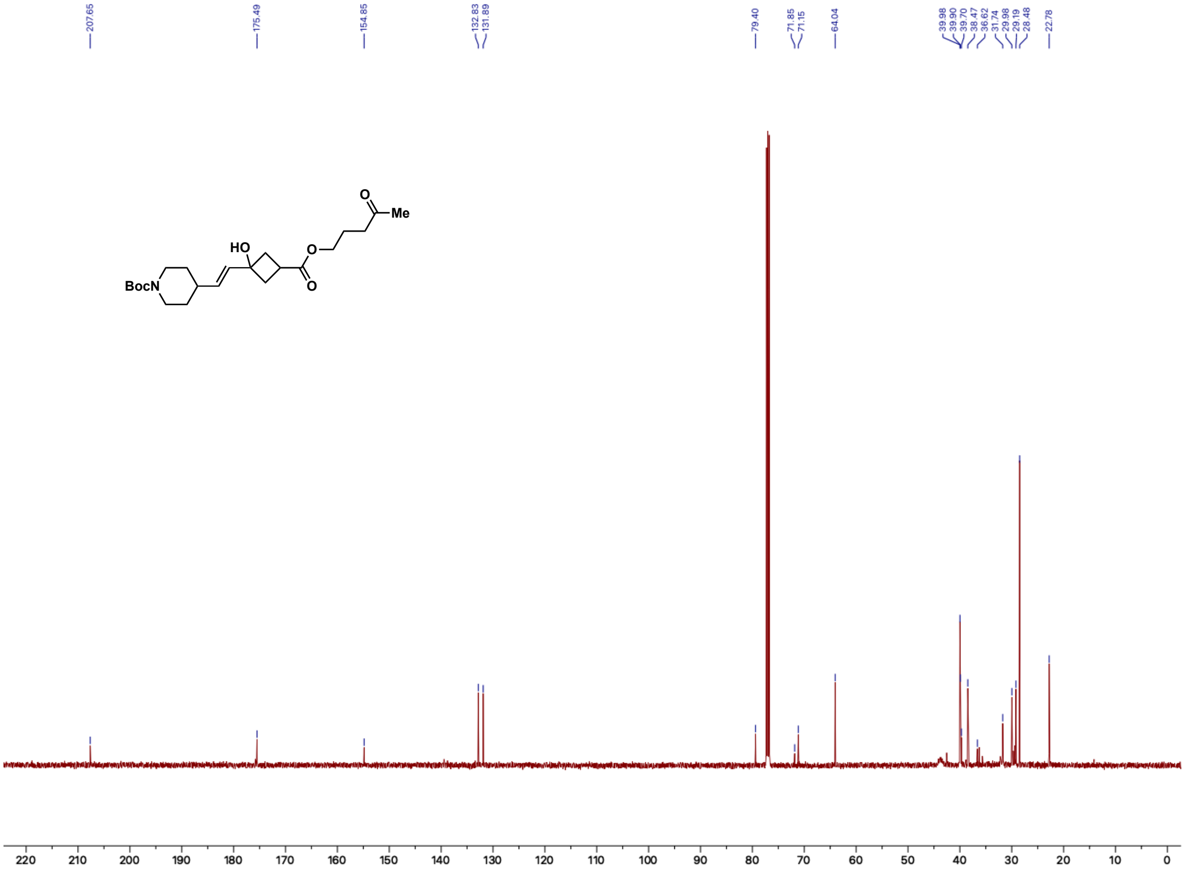

^{1}H-NMR (300 MHz, $CDCl_3$) of compound **26**

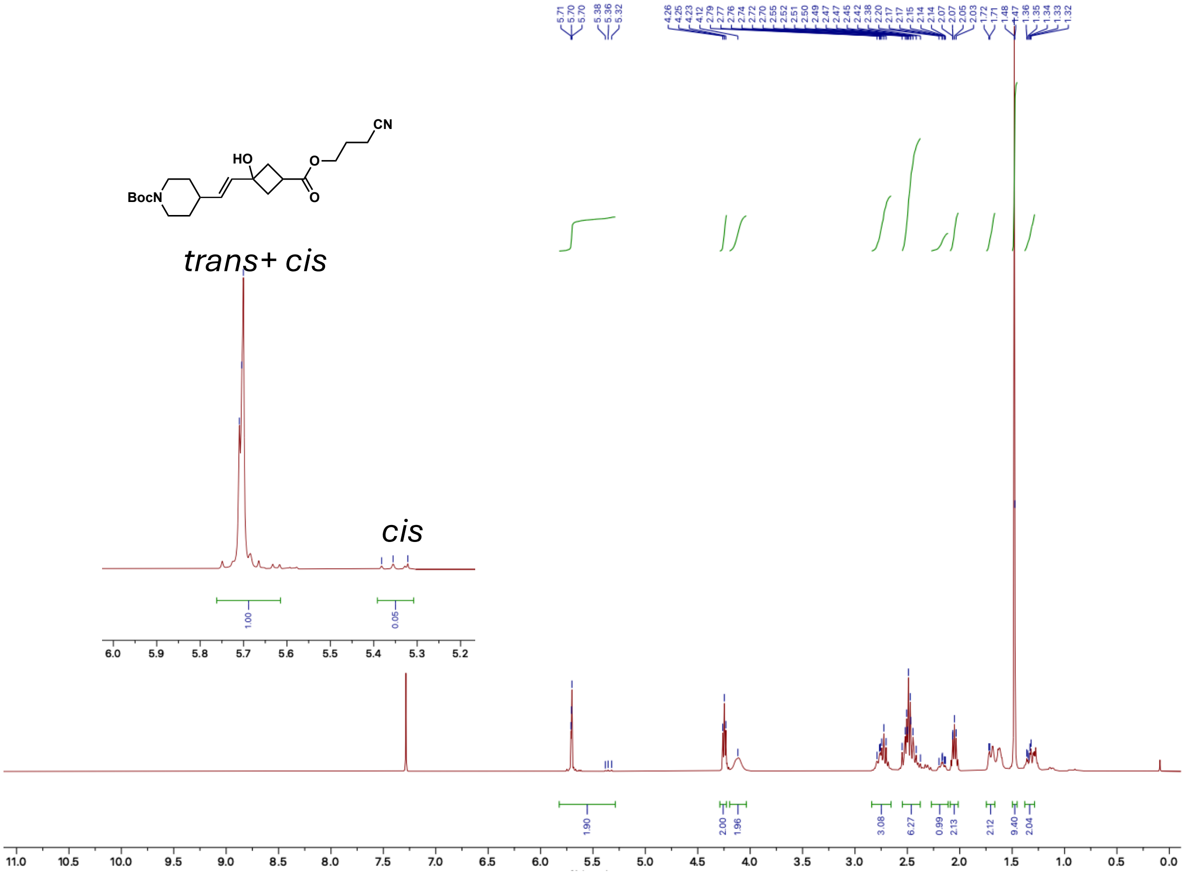

^{13}C-NMR (101 MHz, $CDCl_3$) of compound **26**

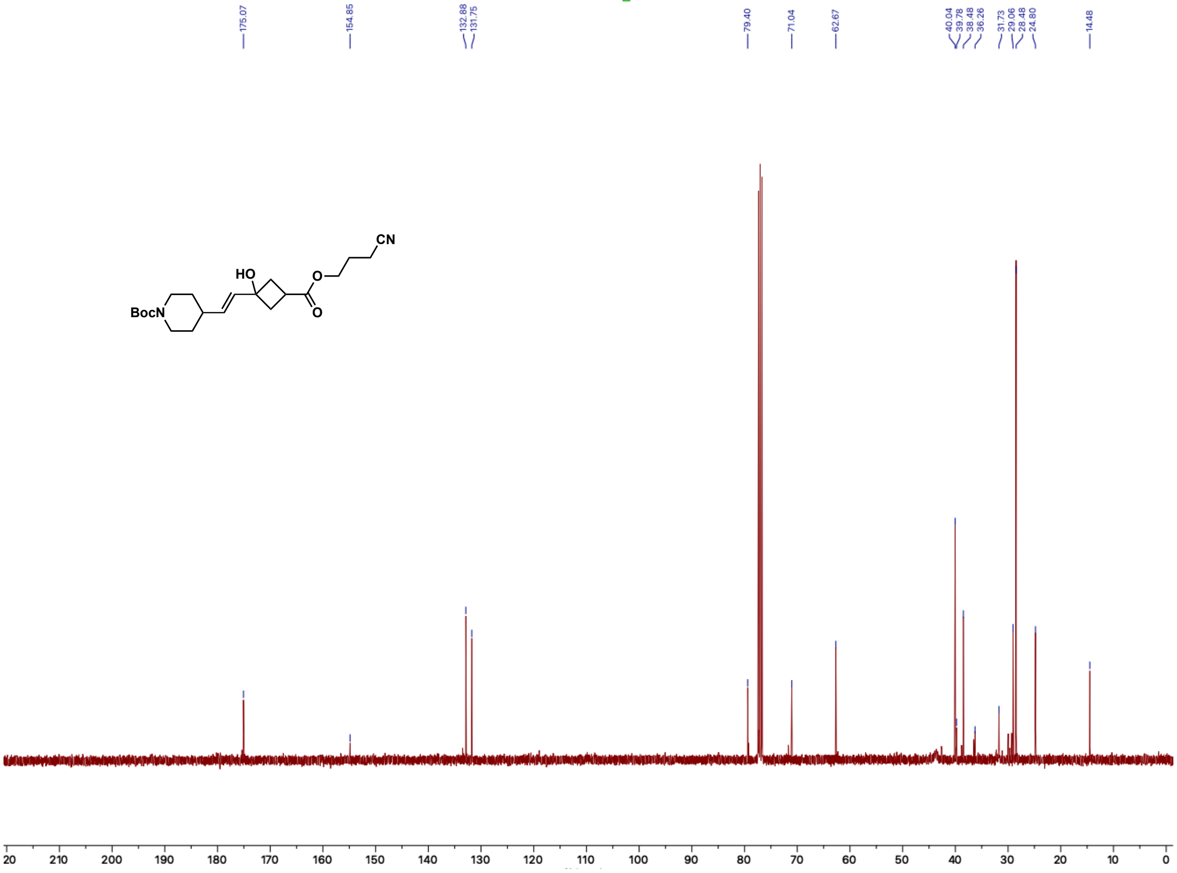

^{1}H-NMR (300 MHz, $CDCl_3$) of compound **27**

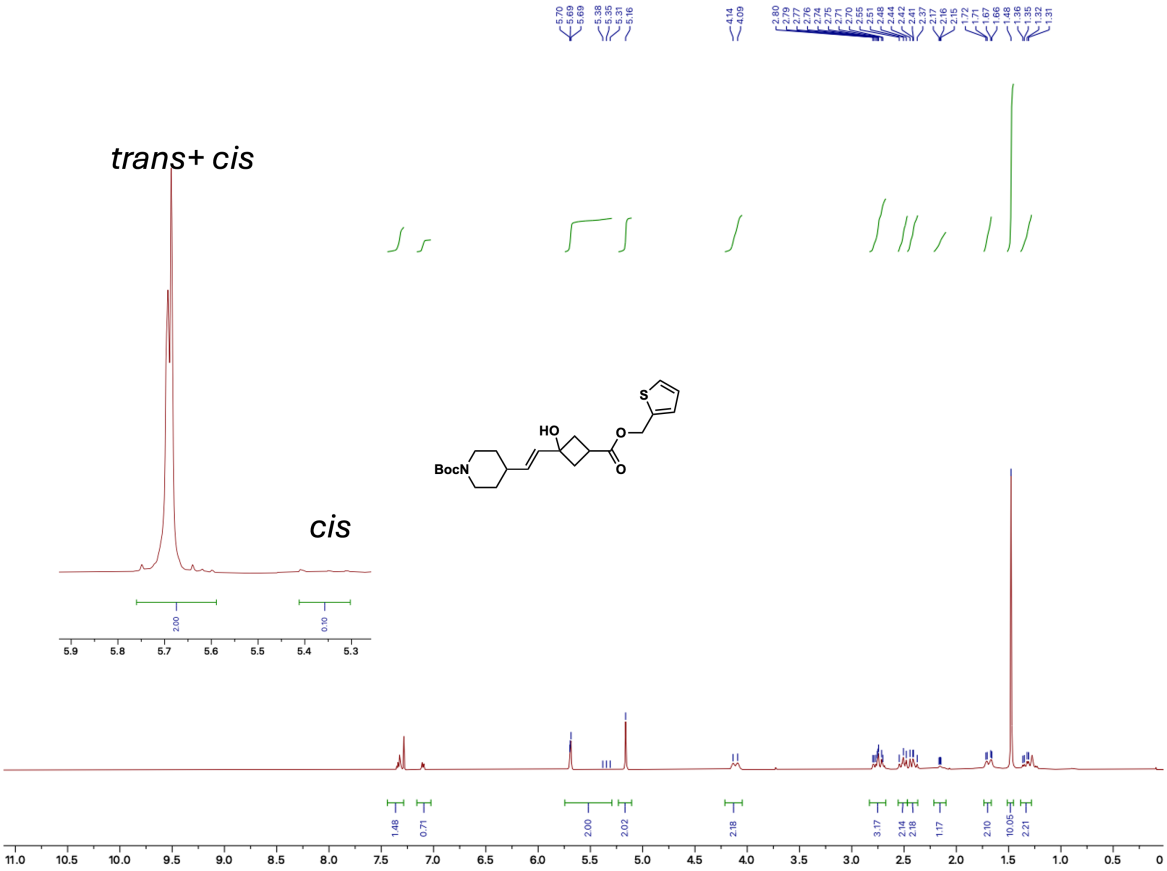

^{13}C-NMR (75 MHz, $CDCl_3$) of compound **27**

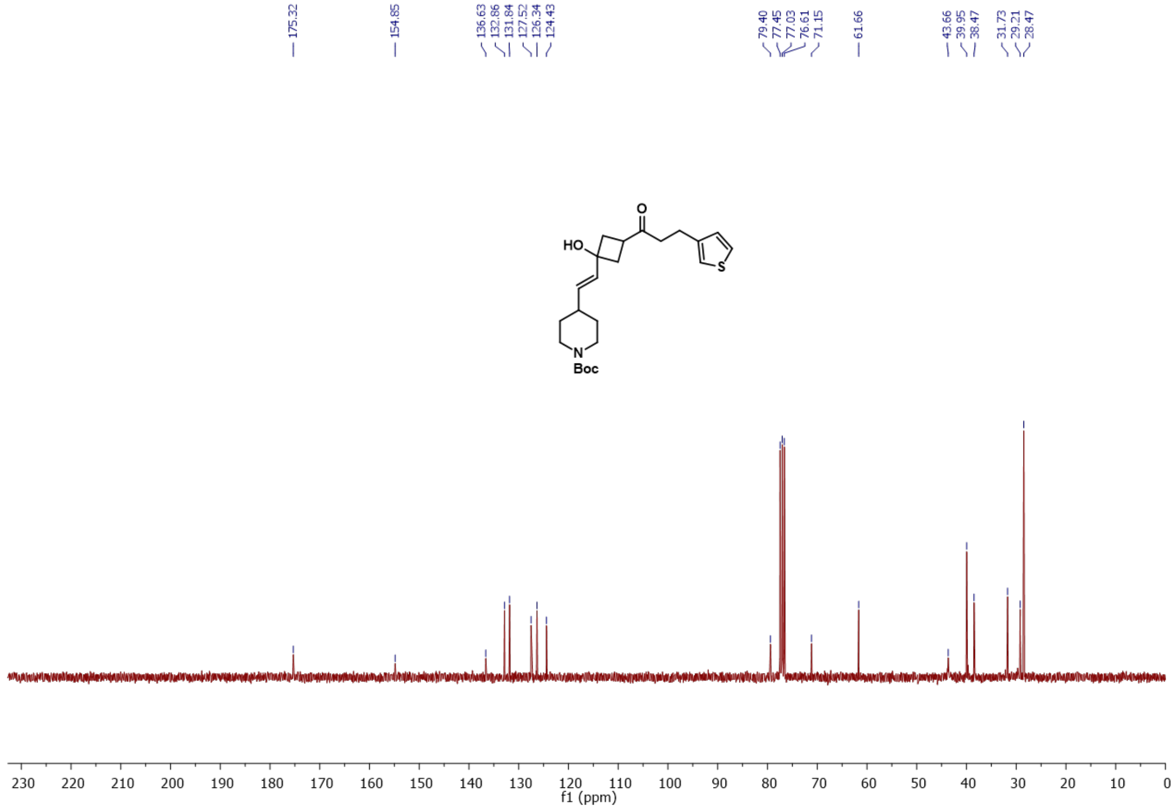

^{1}H-NMR (300 MHz, $CDCl_3$) of compound **28**

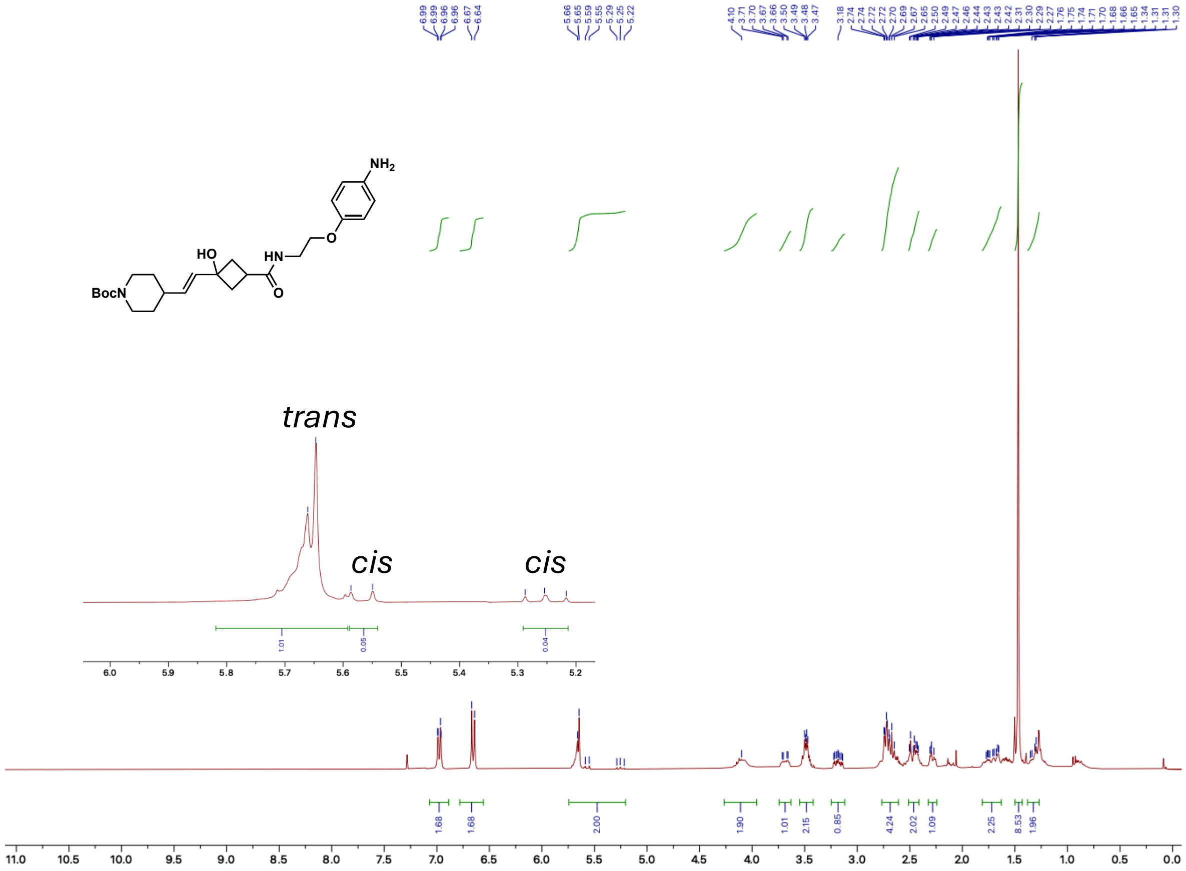

^{13}C-NMR (101 MHz, $CDCl_3$) of compound **28**

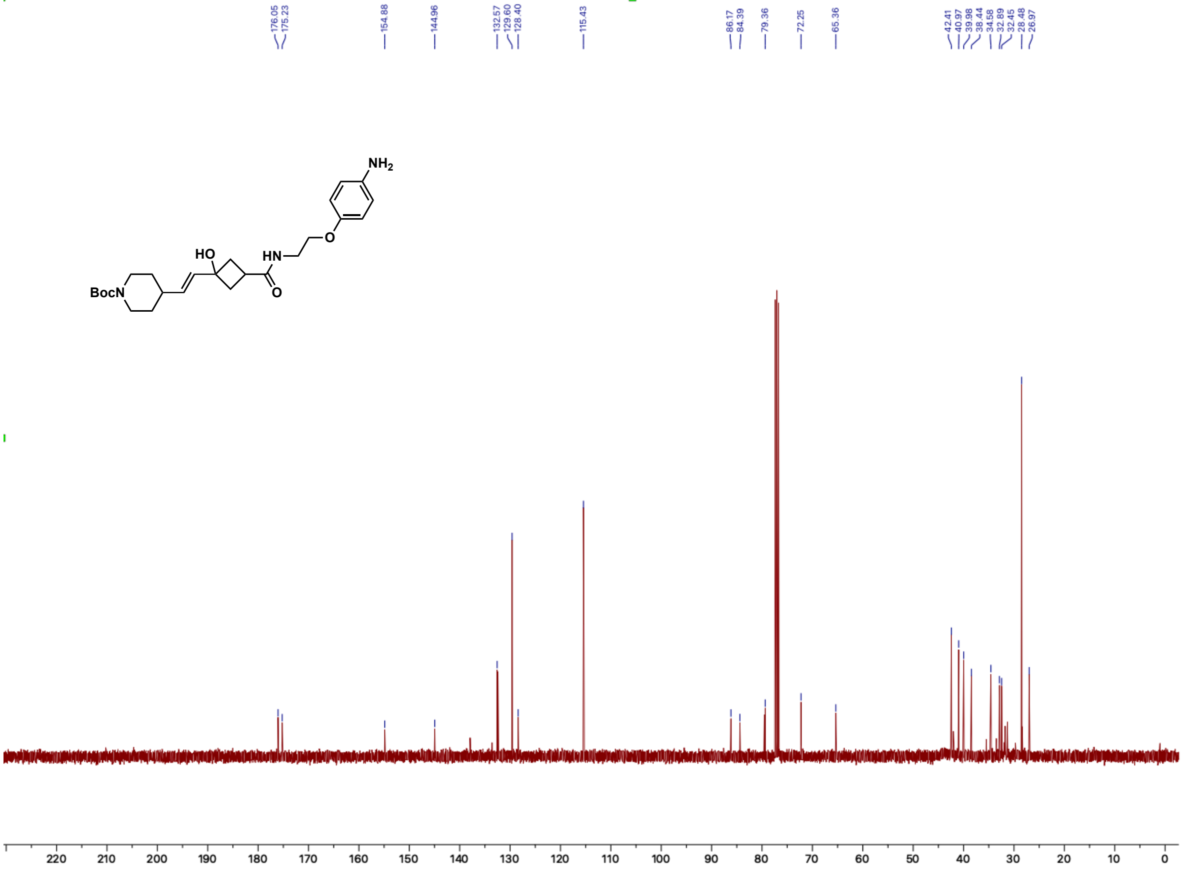

^{1}H-NMR (300 MHz, $CDCl_3$) of compound **29**

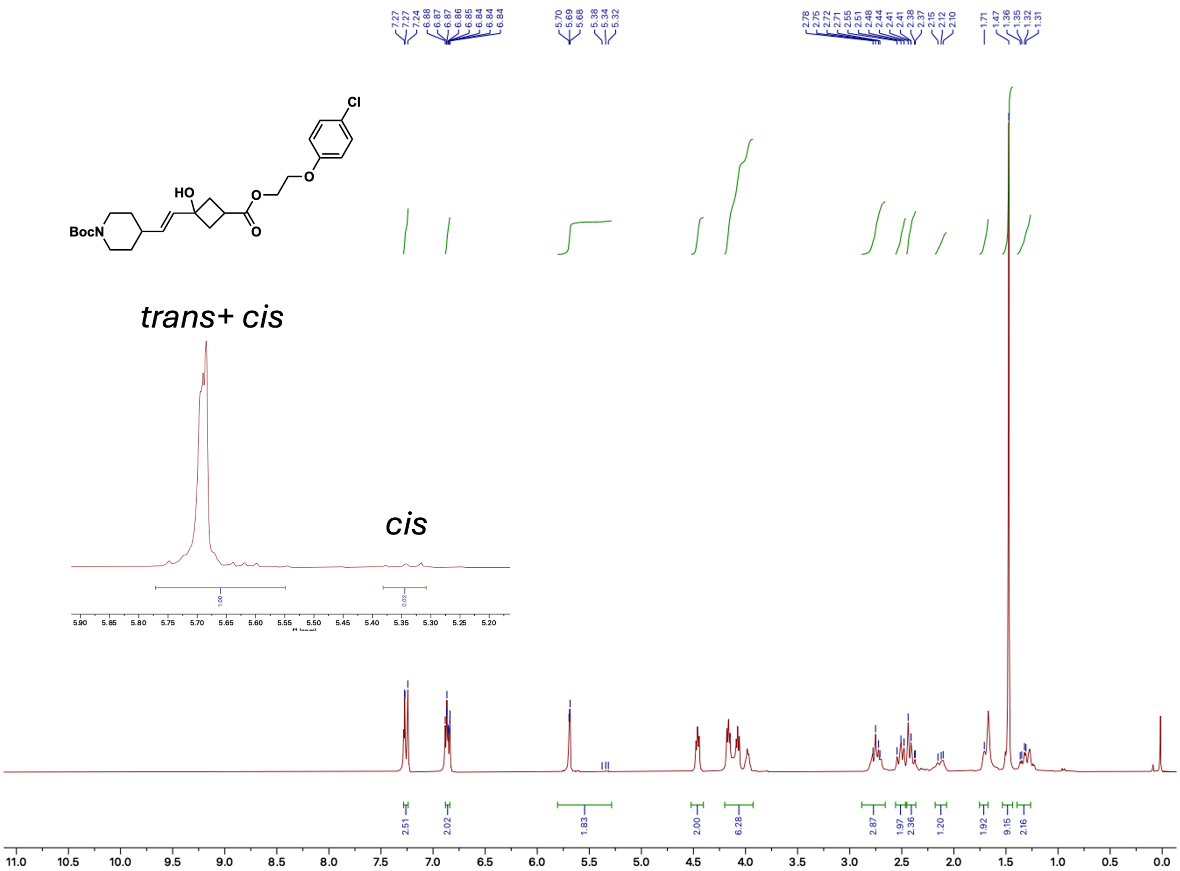

^{13}C-NMR (75 MHz, $CDCl_3$) of compound **29**

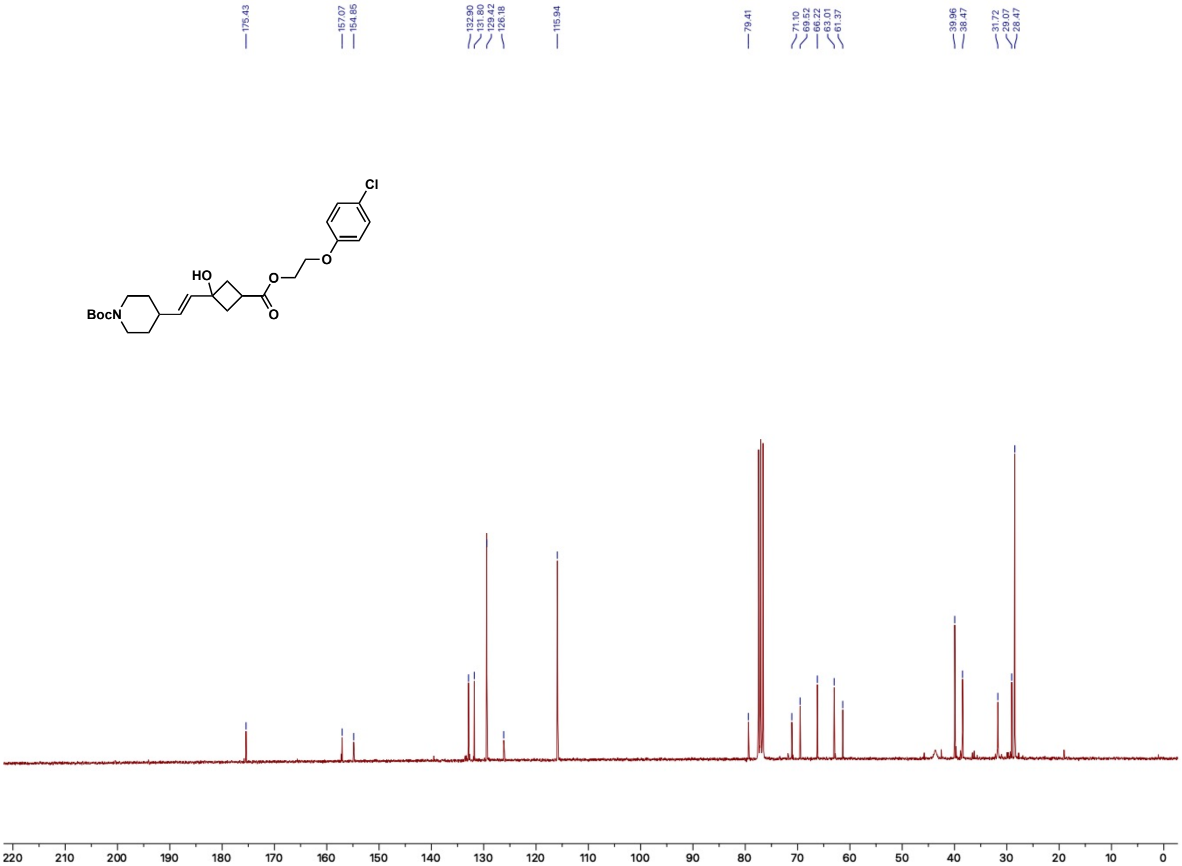

^{1}H-NMR (300 MHz, $CDCl_3$) of compound **30**

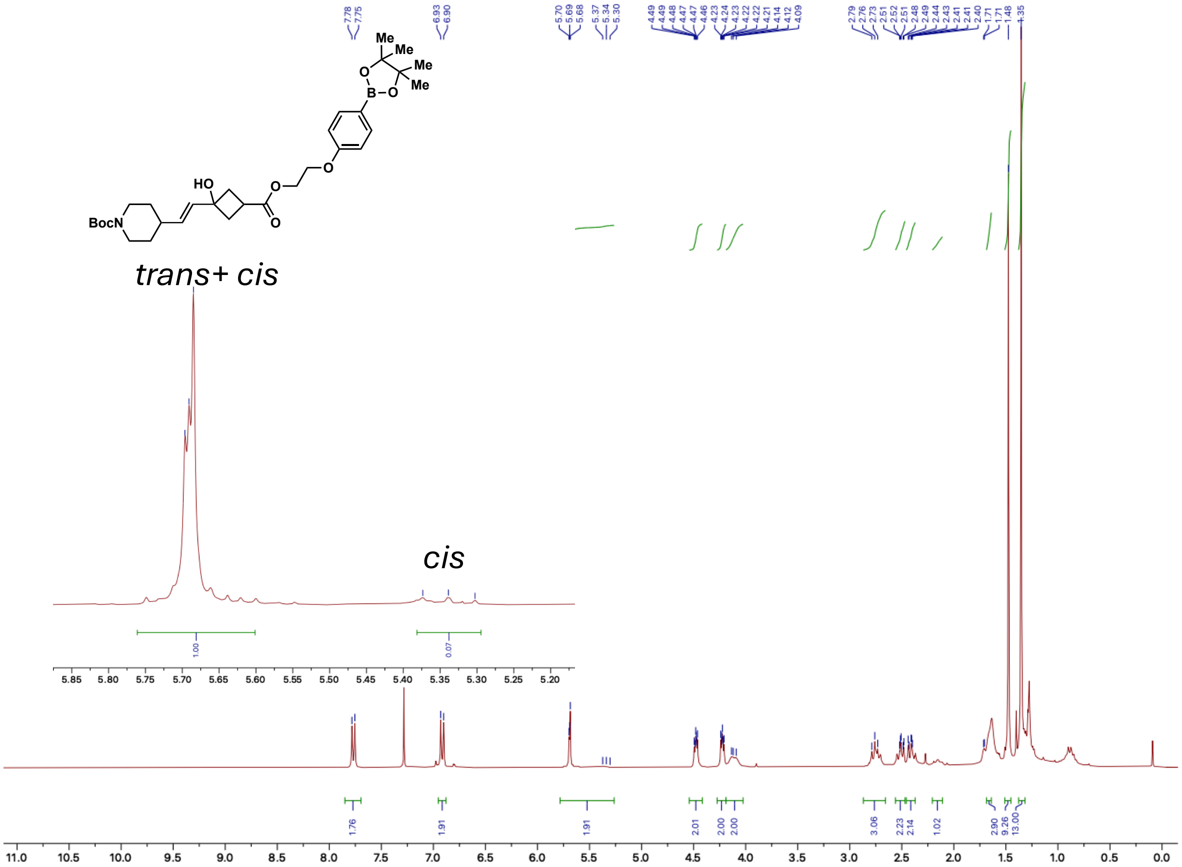

^{13}C-NMR (101 MHz, $CDCl_3$) of compound **30**

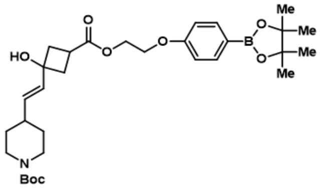

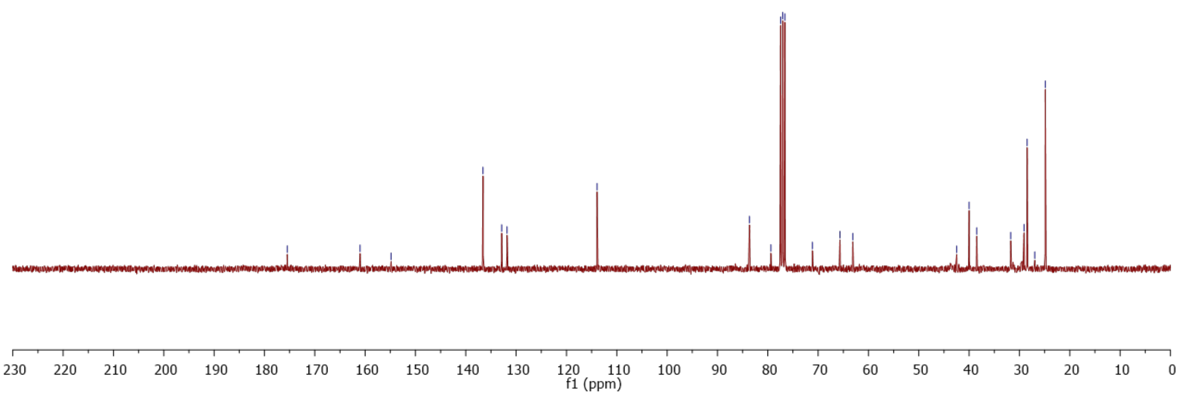

^{11}B NMR (96 MHz, $CDCl_3$) of compound **30**

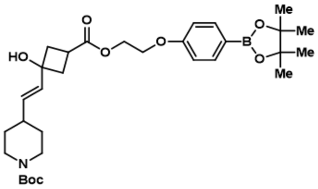

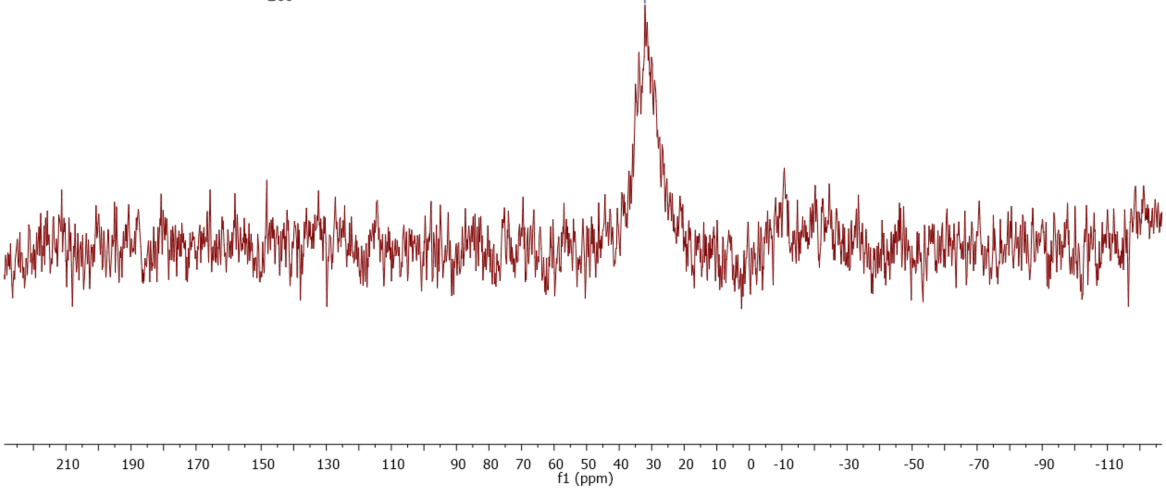

^{1}H-NMR (300 MHz, $CDCl_3$) of compound **31**

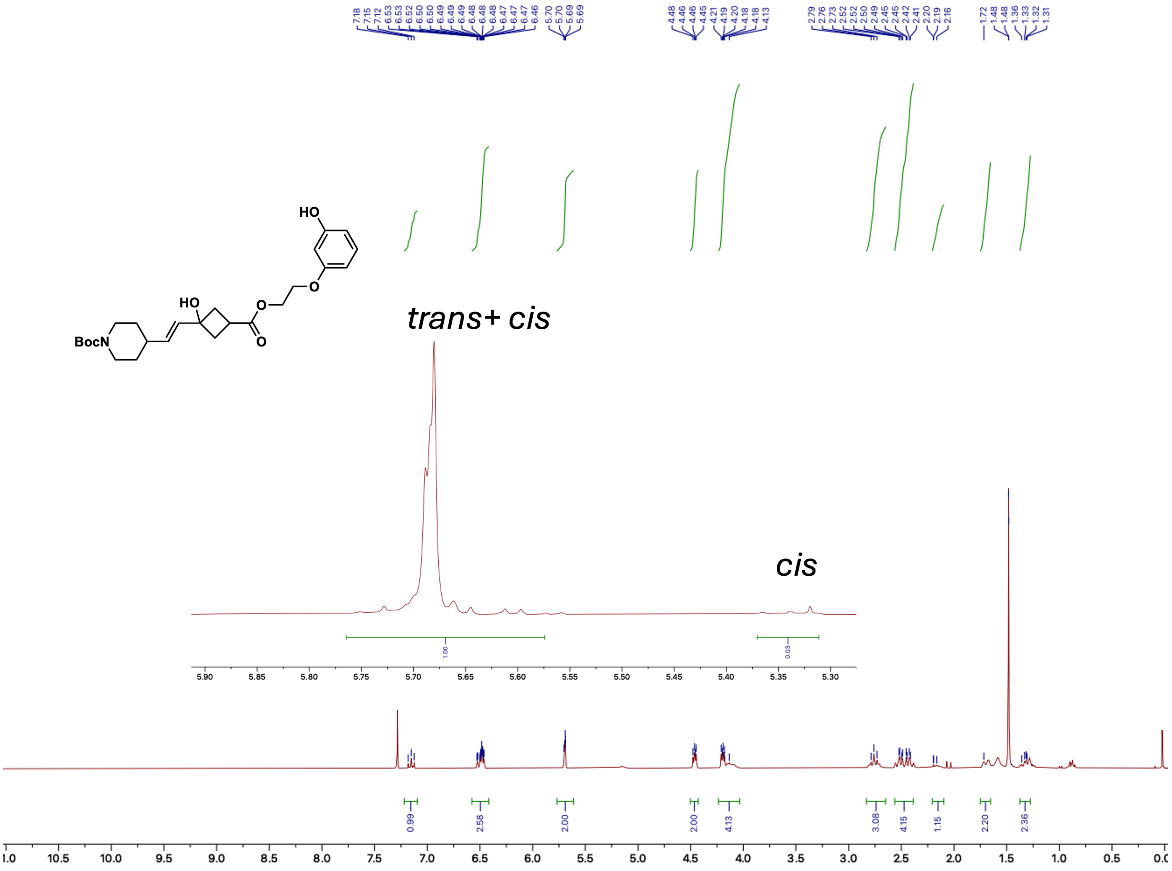

^{13}C-NMR (101 MHz, $CDCl_3$) of compound **31**

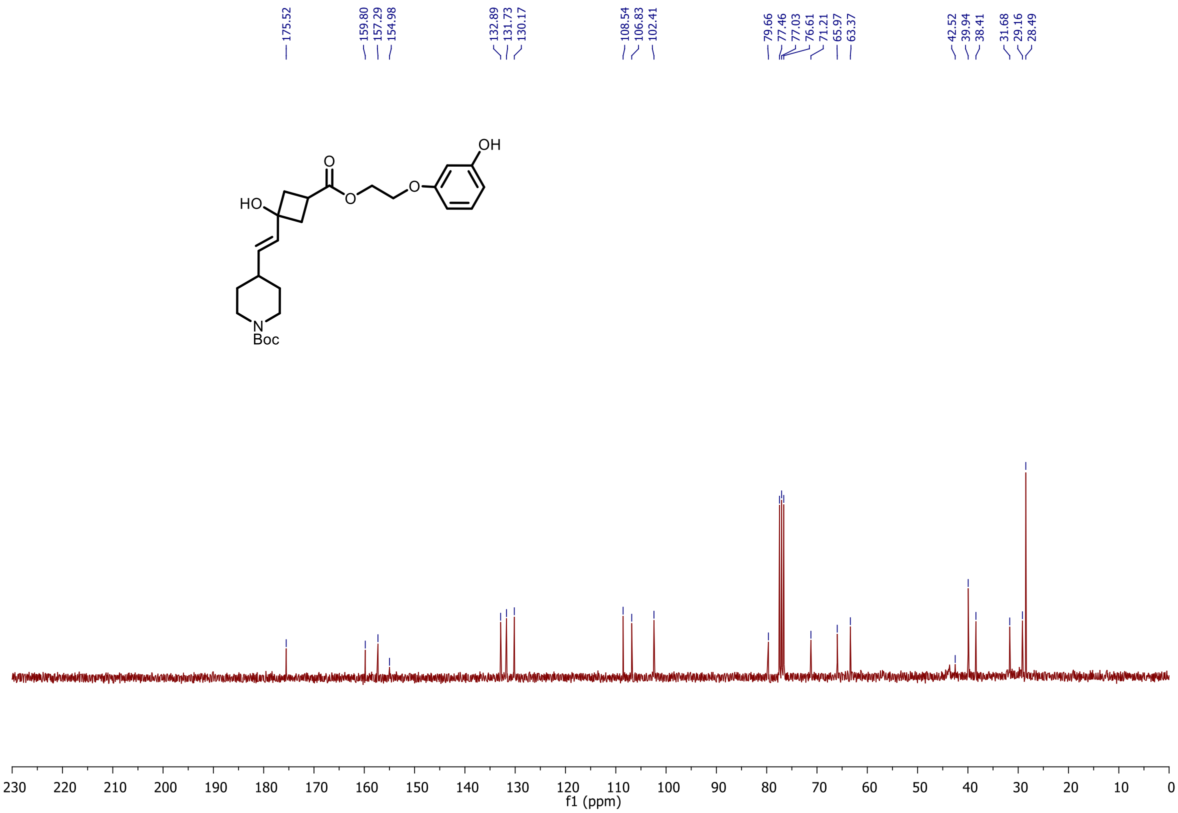

^{1}H-NMR (300 MHz, $CDCl_3$) of compound **32**

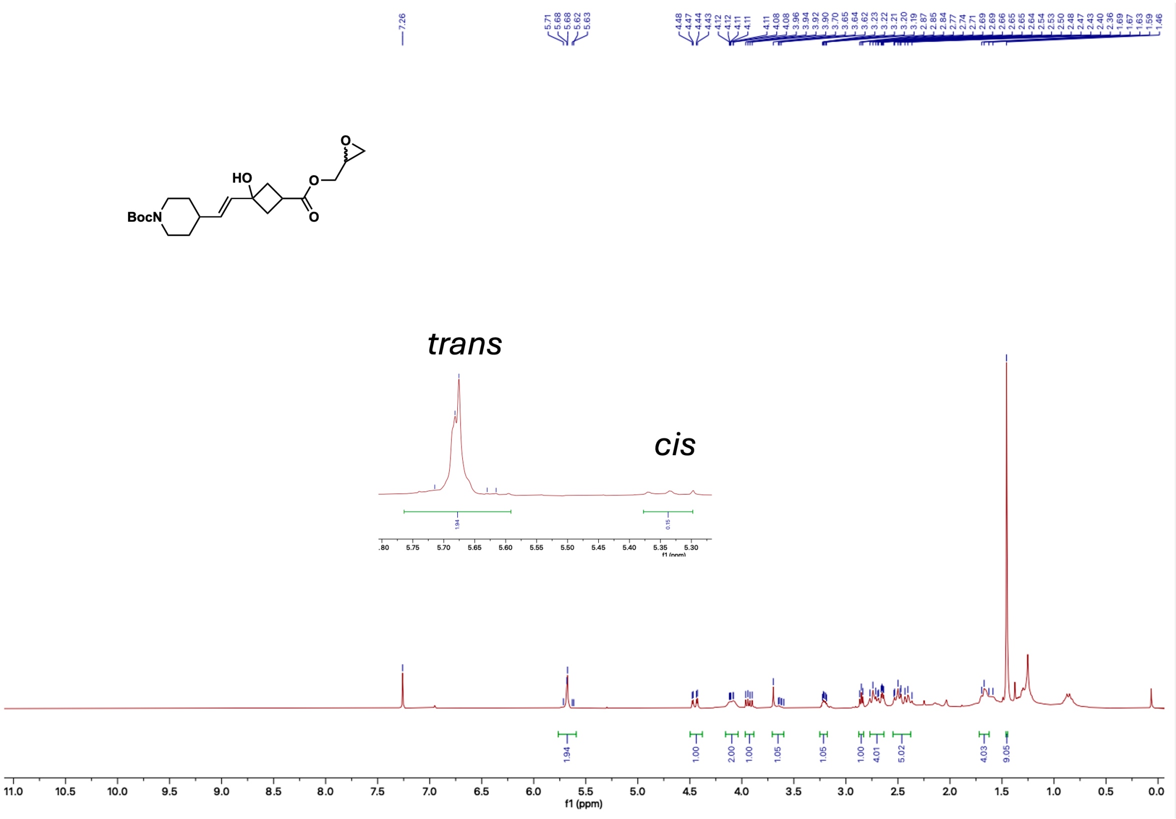

^{13}C-NMR (75 MHz, $CDCl_3$) of compound **32**

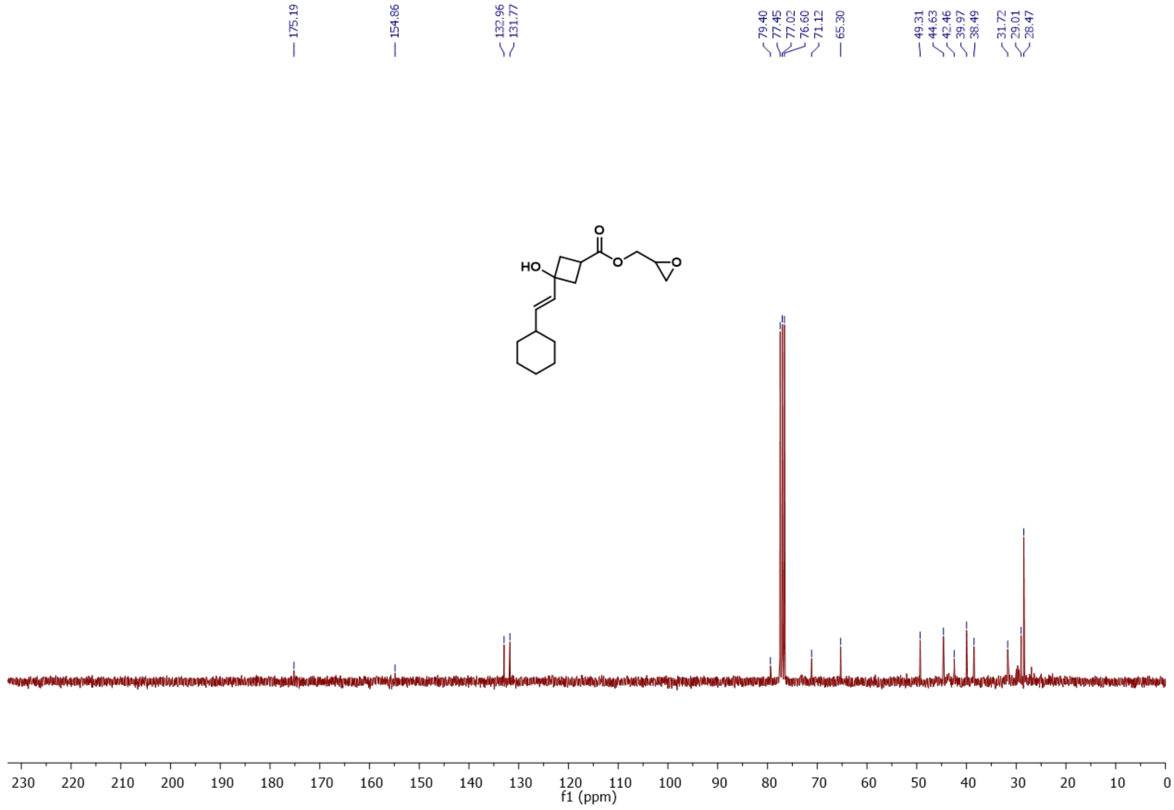

^{1}H-NMR (400 MHz, $CDCl_3$) of compound **38**

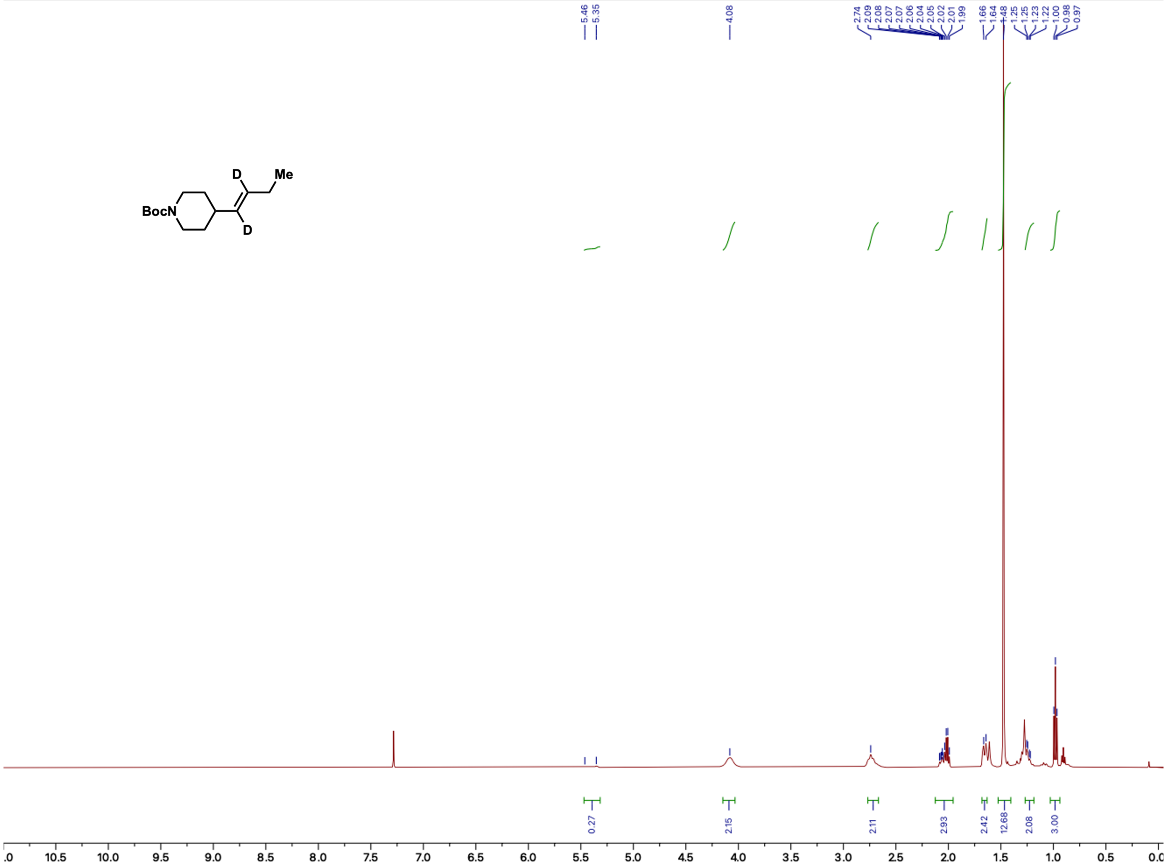

^{13}C-NMR (101 MHz, $CDCl_3$) of compound **38**

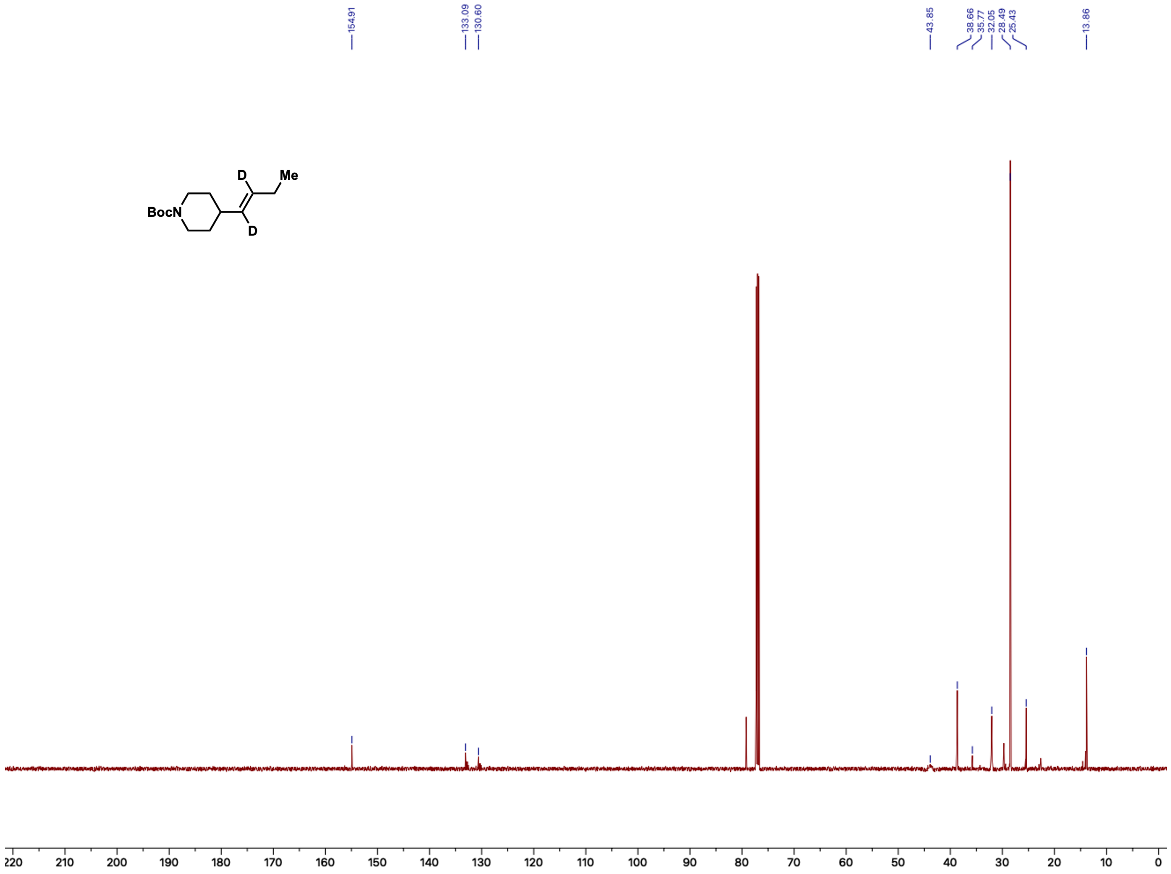

^{2}H-NMR (61 MHz, $CDCl_3$) of compound **38**

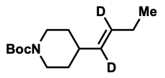

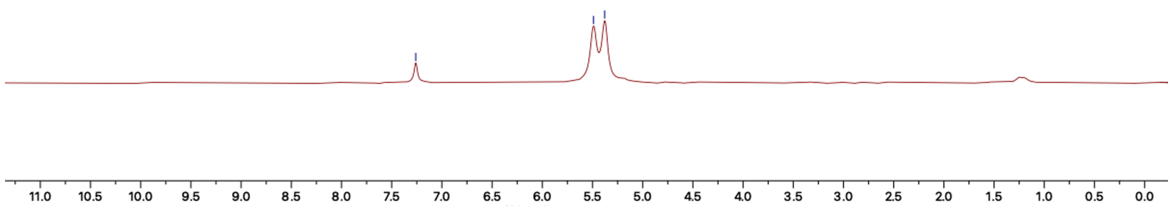

^{1}H-NMR (400 MHz, $CDCl_3$) of compound **39**

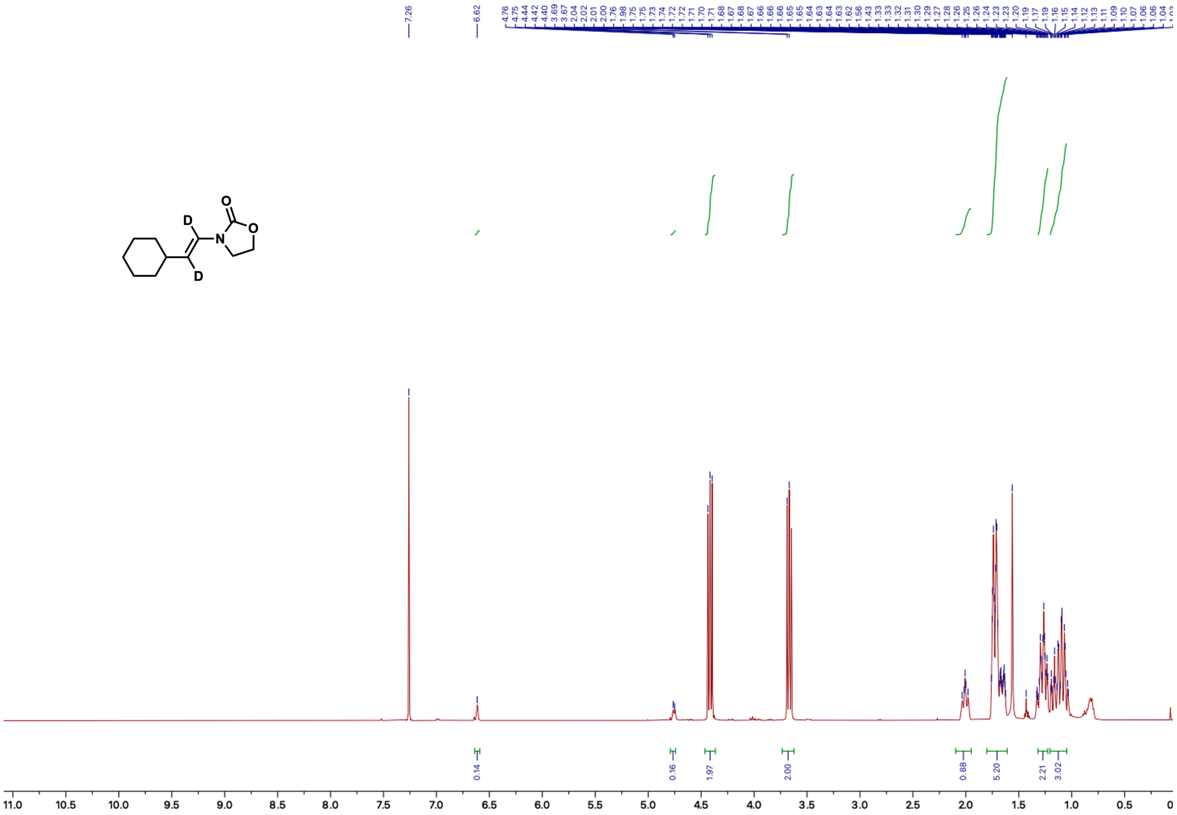

^{13}C-NMR (101 MHz, $CDCl_3$) of compound **39**

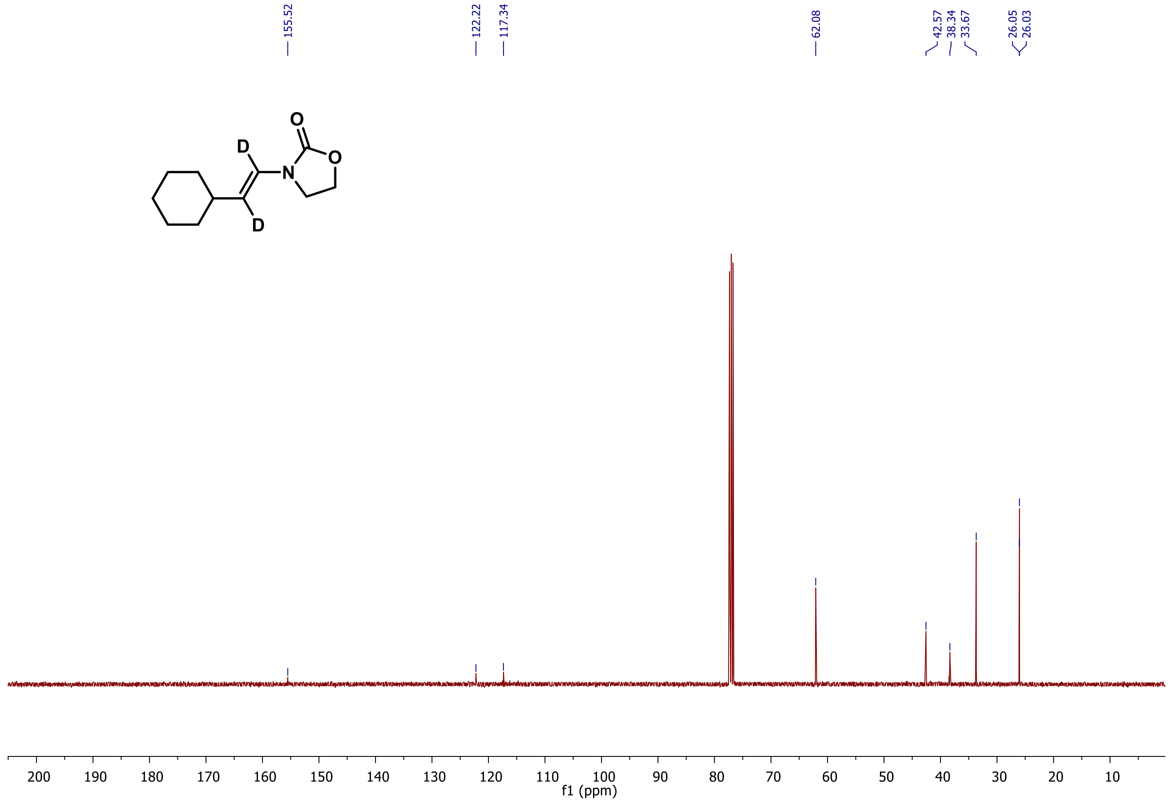

^{2}H-NMR (61 MHz, $CDCl_3$) of compound **39**

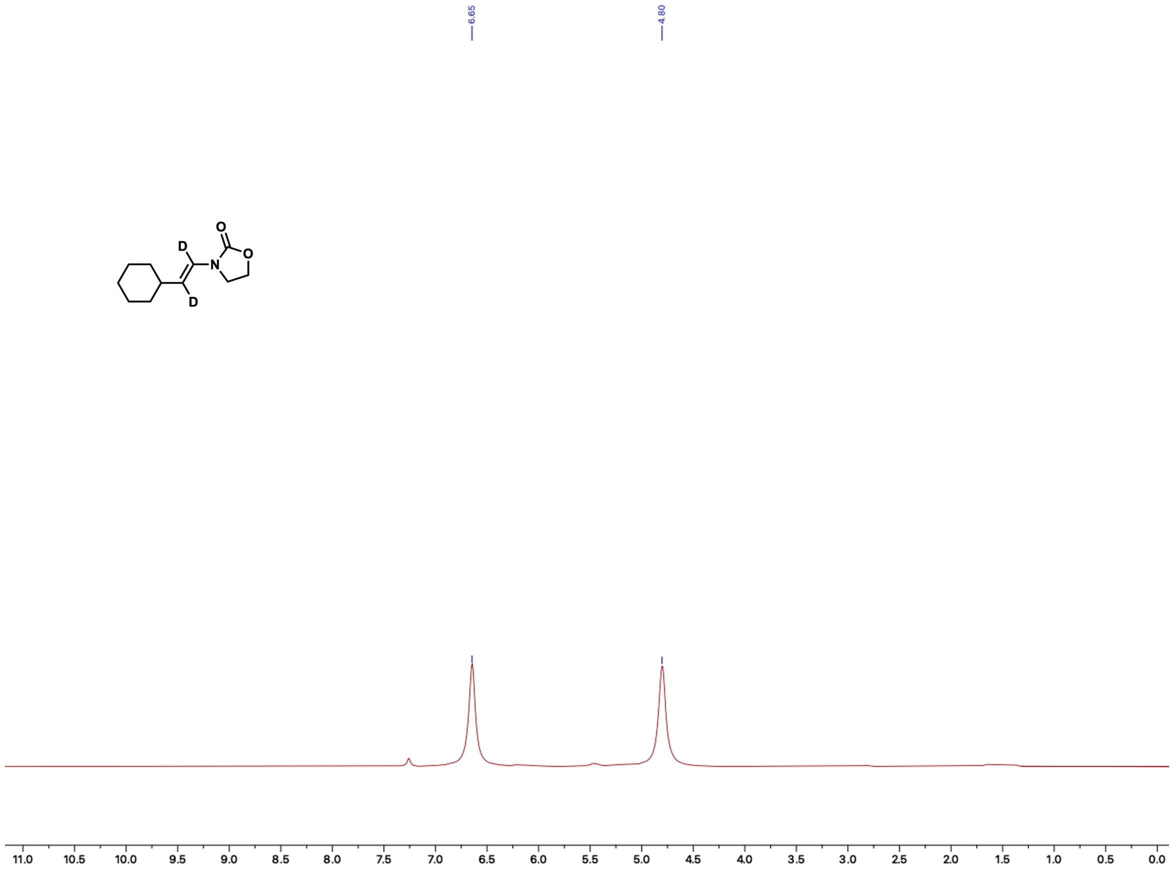

^{1}H-NMR (400 MHz, $CDCl_3$) of compound **40**

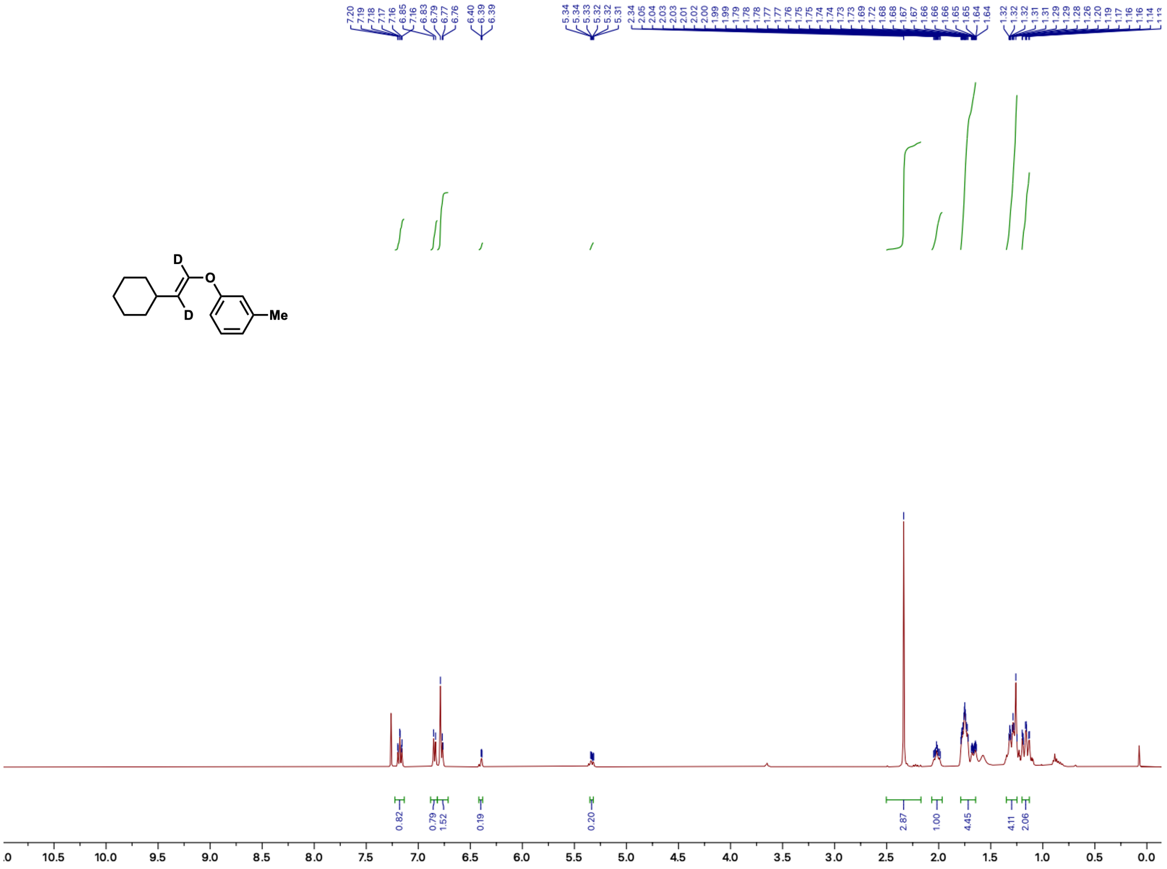

^{13}C-NMR (101 MHz, CDCl3) of compound **40**

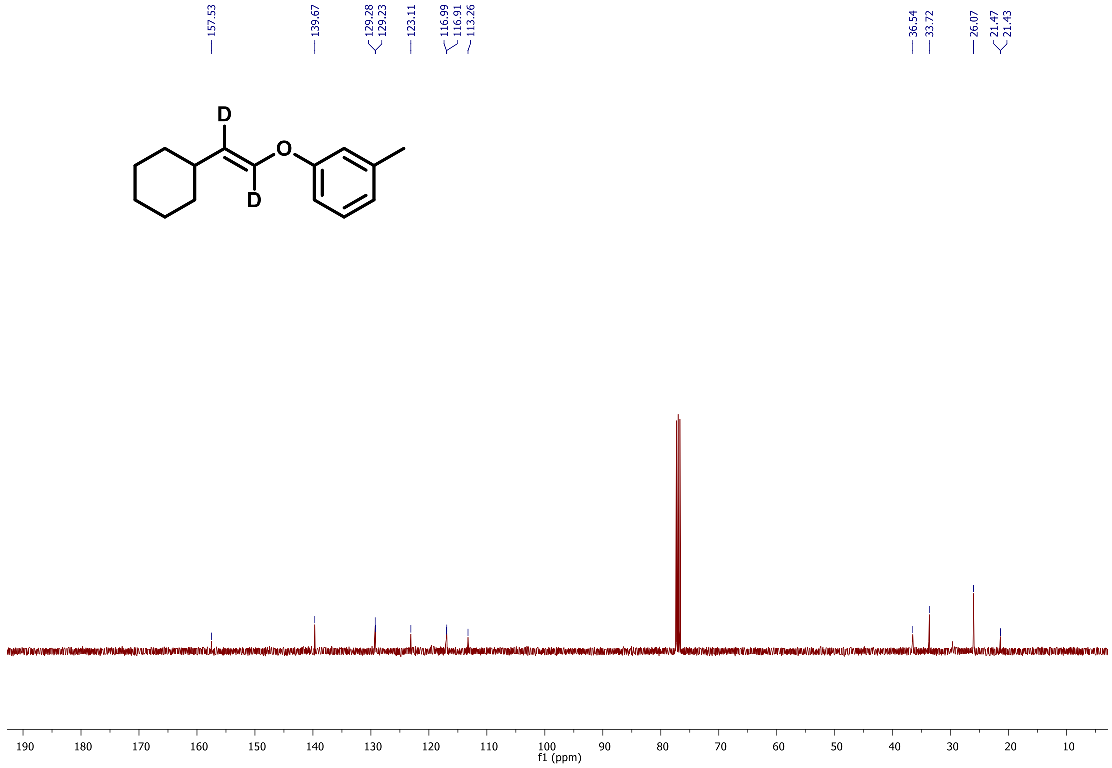

^{2}H-NMR (61 MHz, $CDCl_3$) of compound **40**

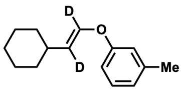

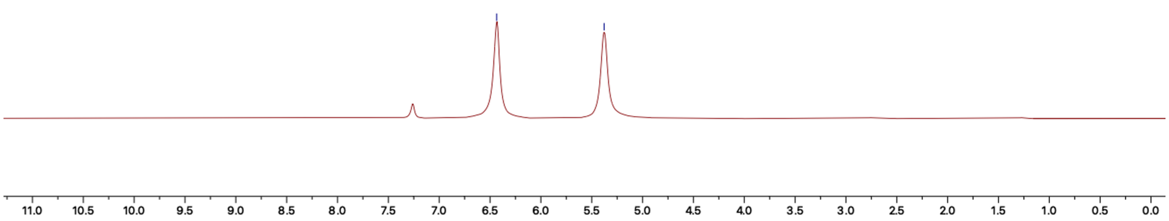

^{1}H-NMR (400 MHz, $CDCl_3$) of compound **41**

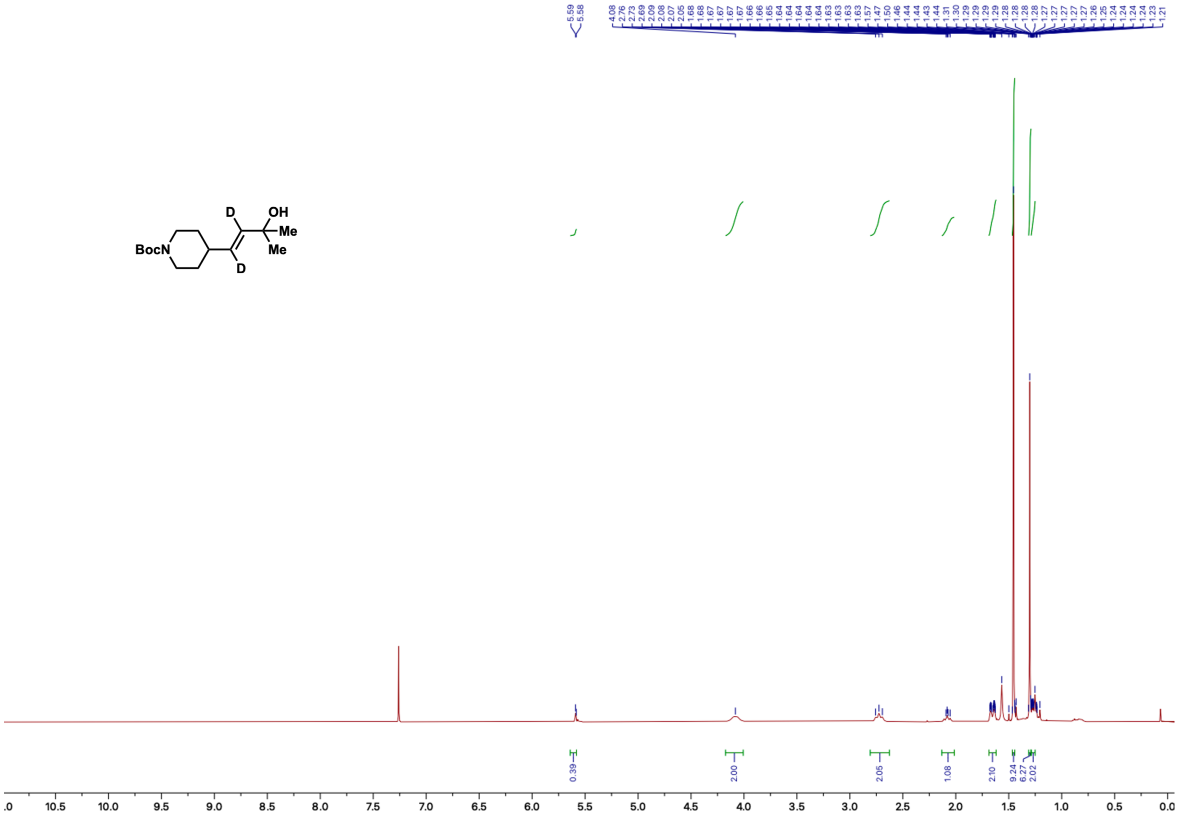

^{13}C-NMR (126 MHz, $CDCl_3$) of compound **41**

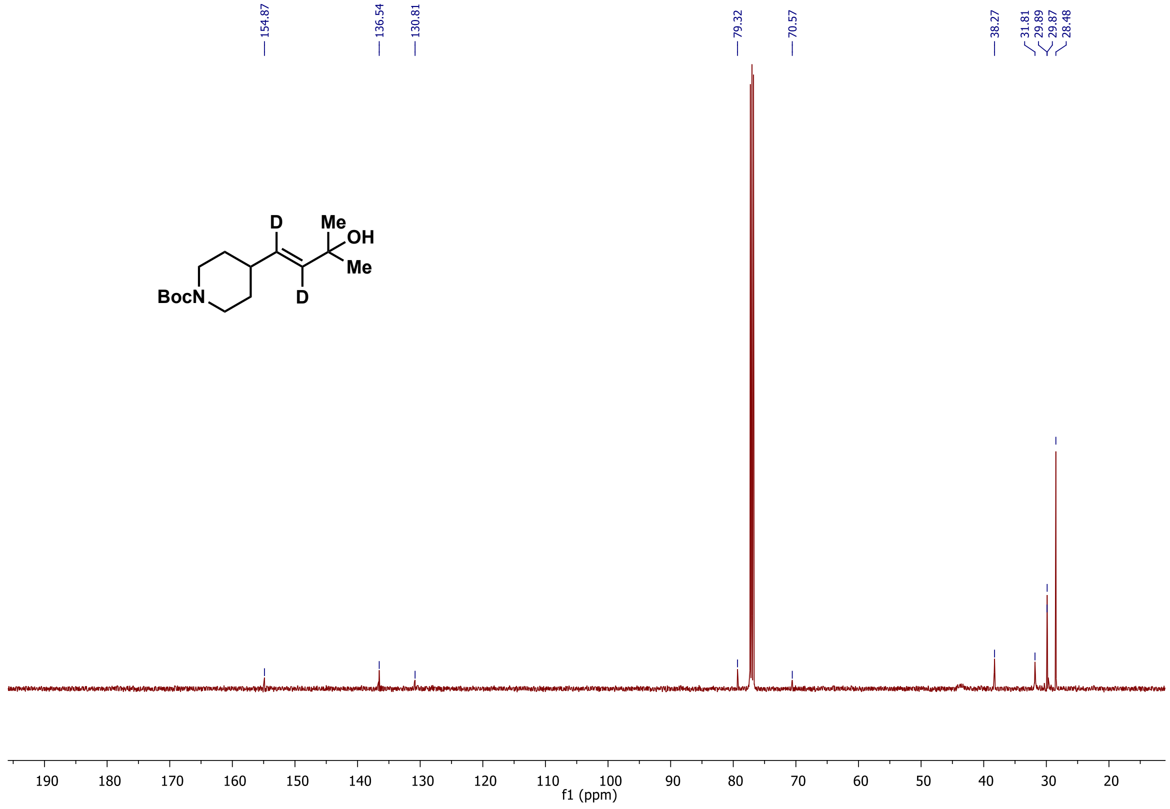

^{2}H-NMR (61 MHz, $CDCl_3$) of compound **41**

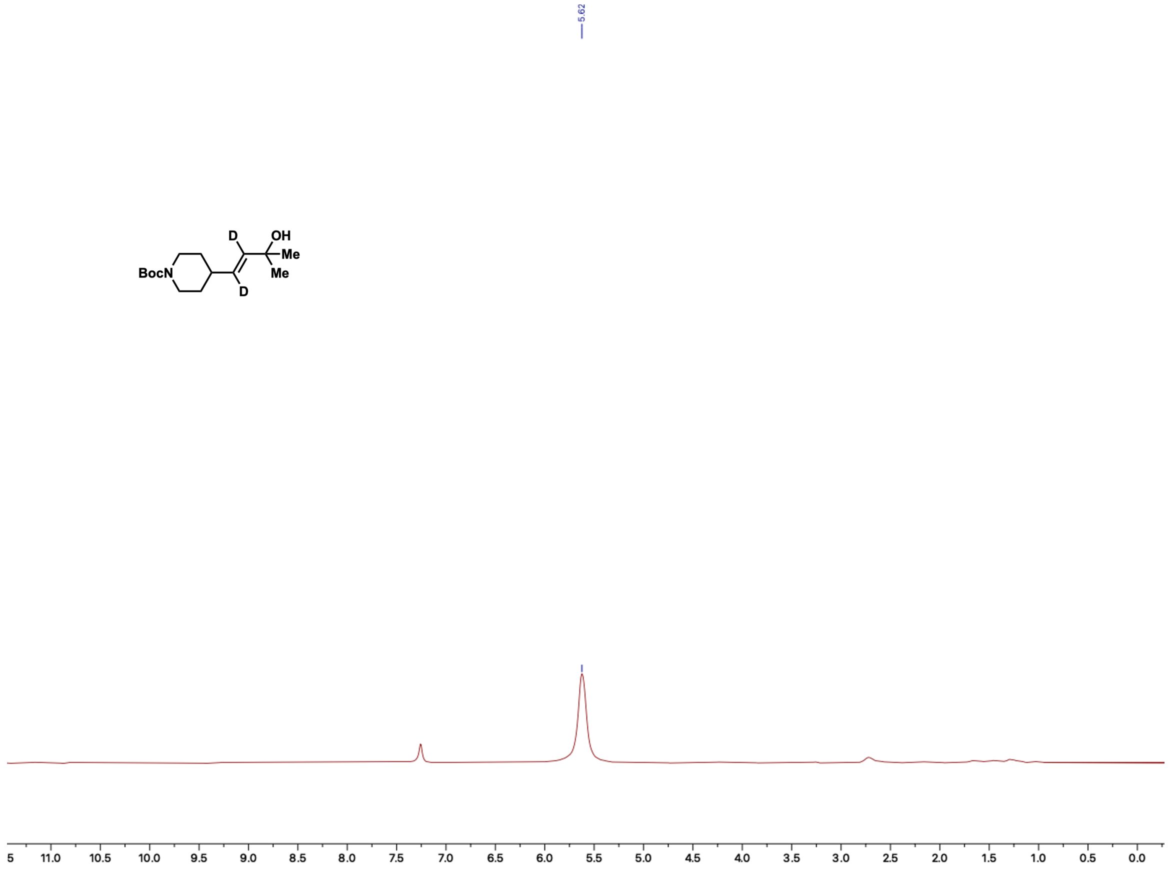

^{1}H-NMR (300 MHz, $CDCl_3$) of compound **42**

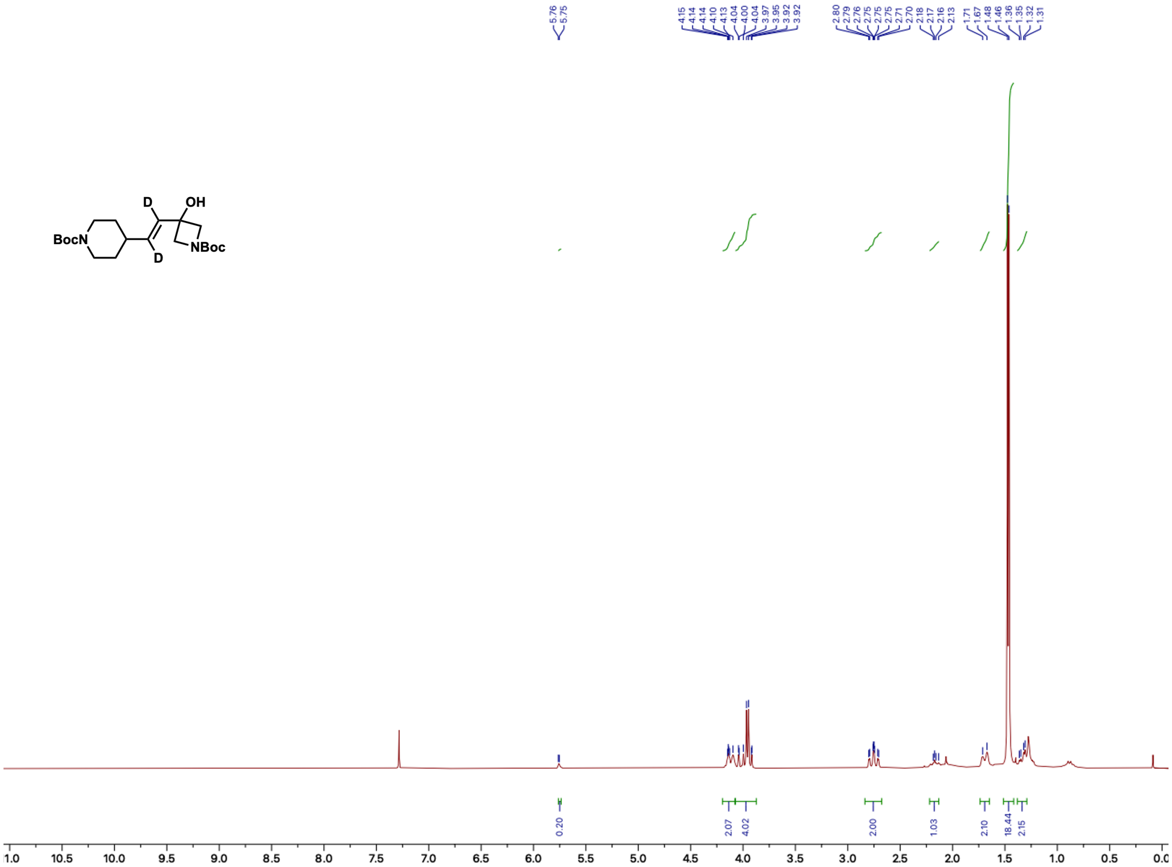

^{13}C-NMR (201 MHz, $CDCl_3$) of compound **42**

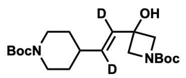

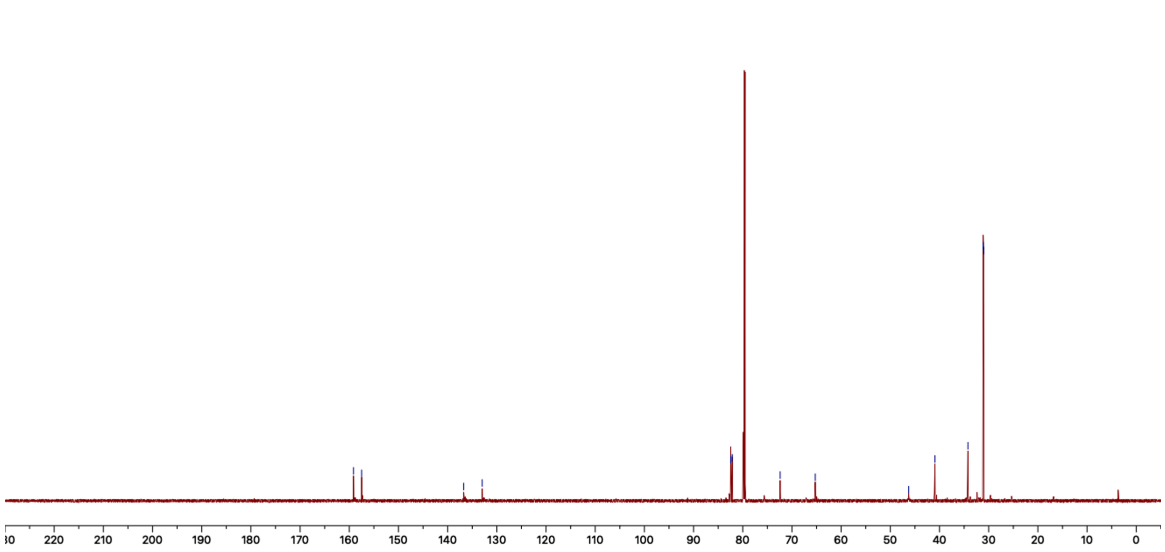

^{2}H-NMR (61 MHz, $CDCl_3$) of compound **42**

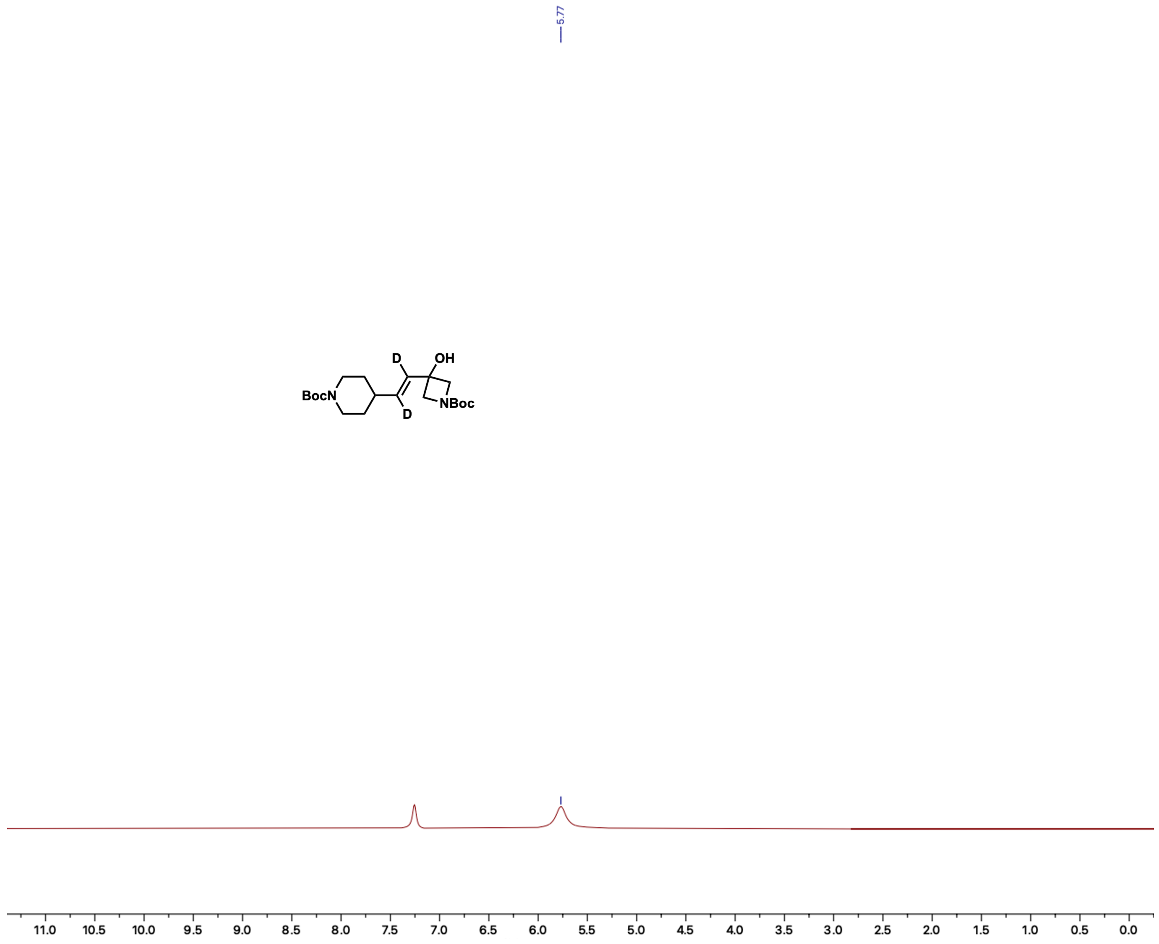

^{1}H NMR (800 MHz, $CDCl_3$) of compound **44**

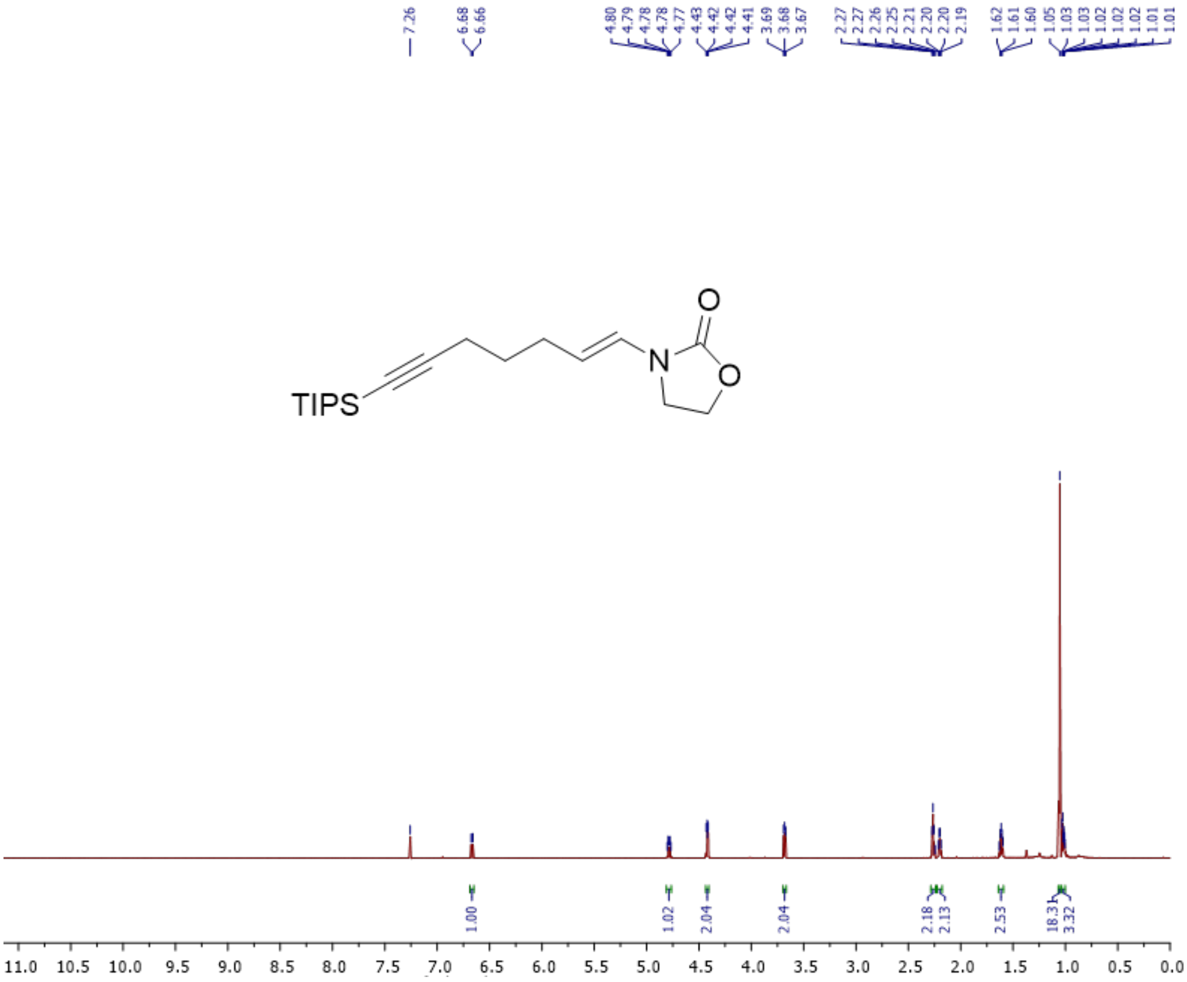

^{13}C NMR (201 MHz, $CDCl_3$) of compound **44**

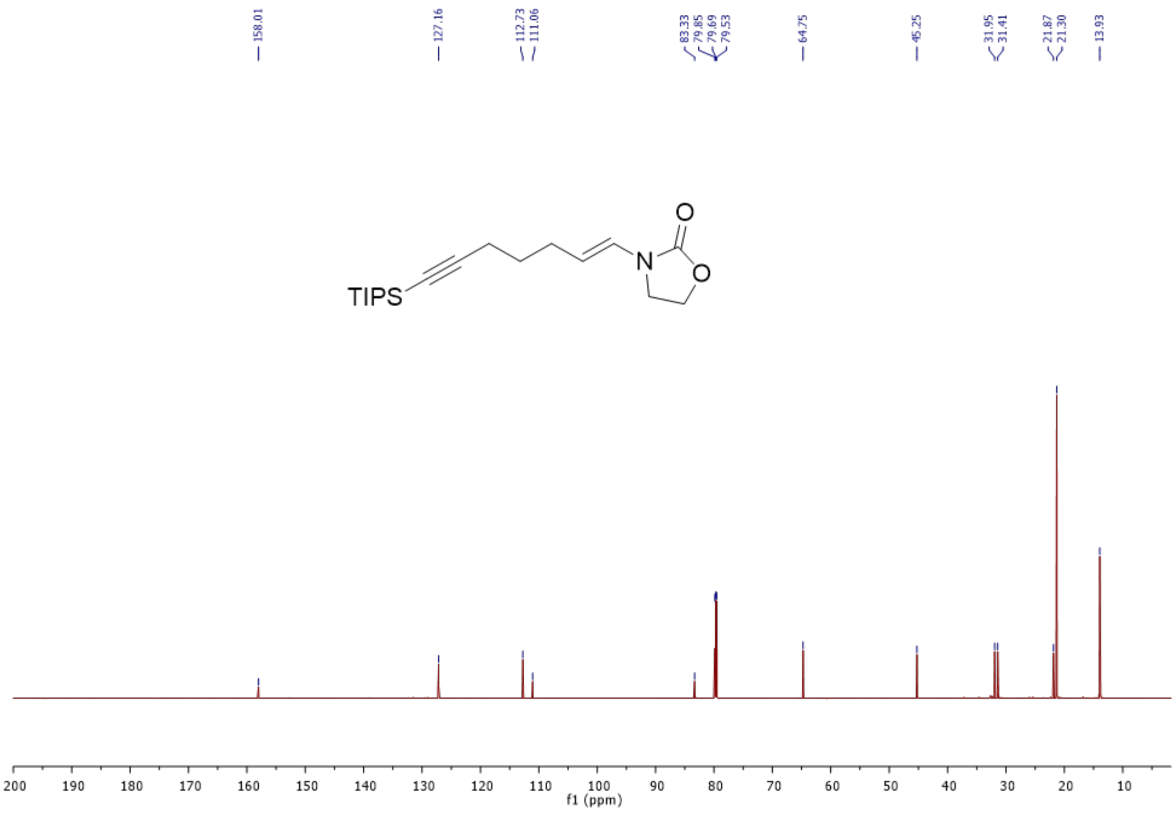

^{1}H NMR (400 MHz, $CDCl_3$) of compound **45**

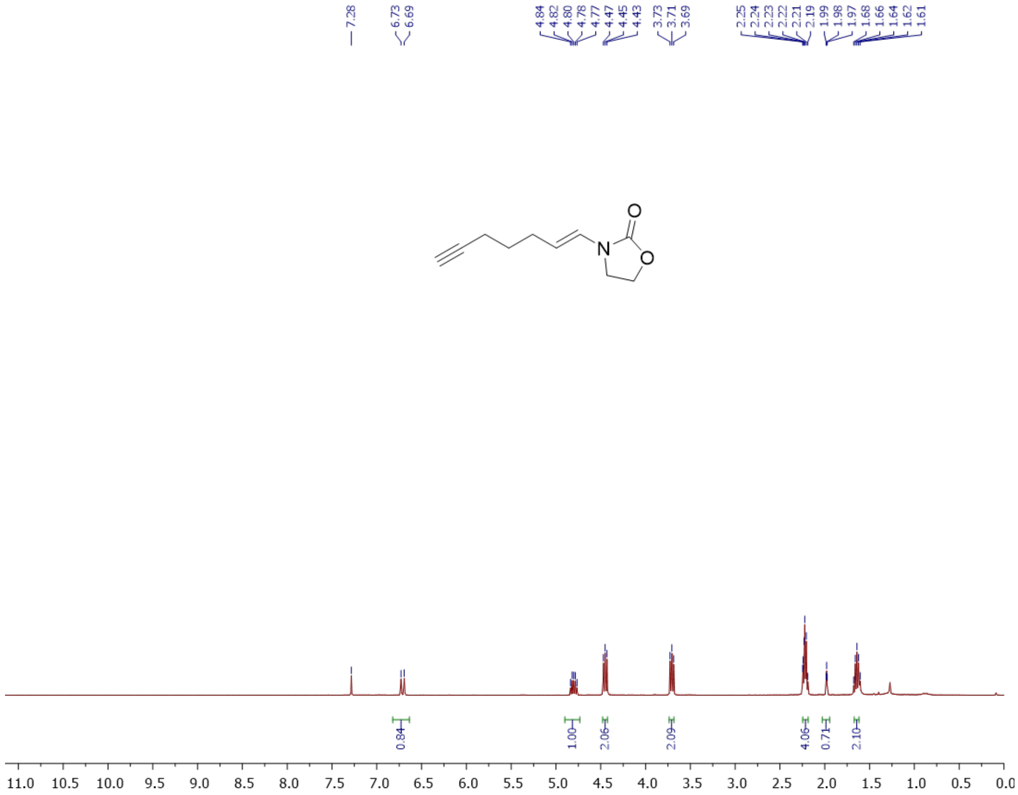

^{13}C NMR (101 MHz, $CDCl_3$) of compound **45**

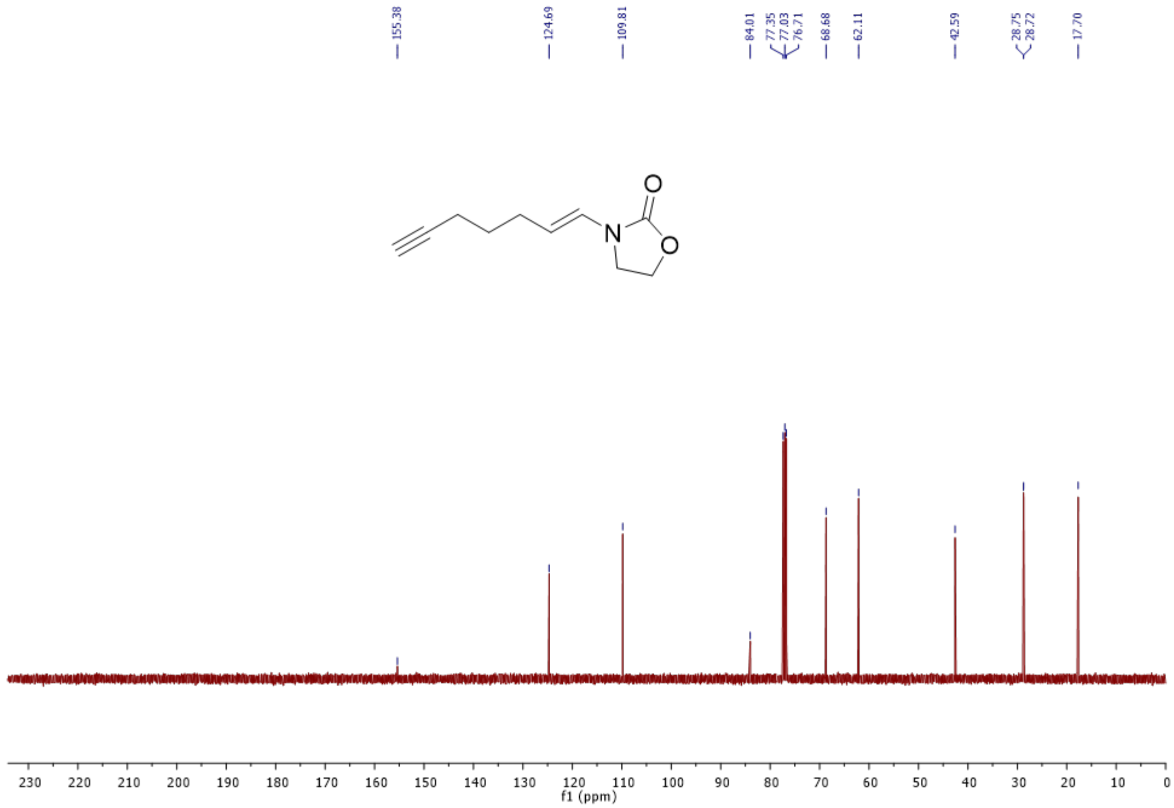

^{1}H NMR (400 MHz, $CDCl_3$) of compound **46**

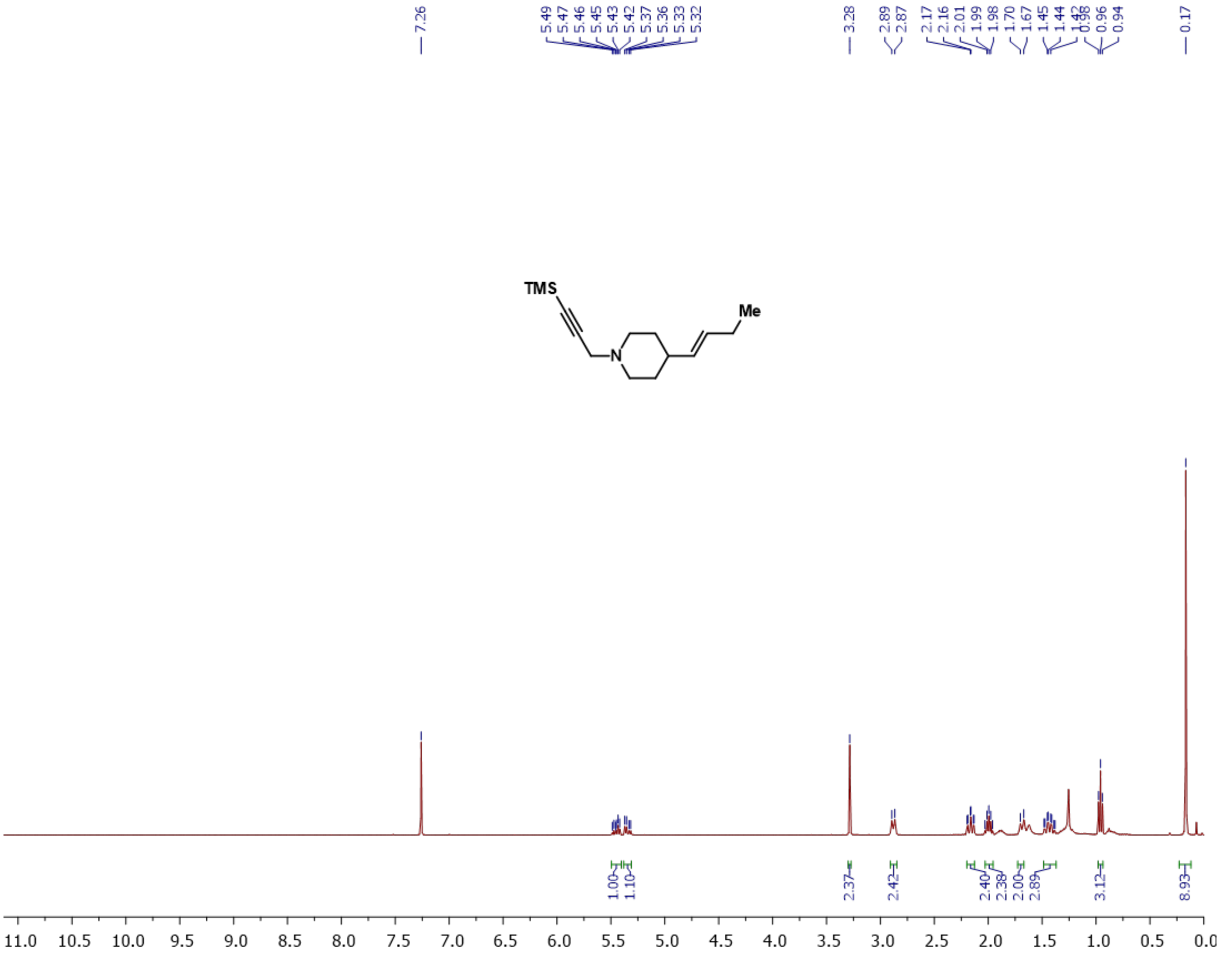

^{13}C NMR (101 MHz, $CDCl_3$) of compound **46**

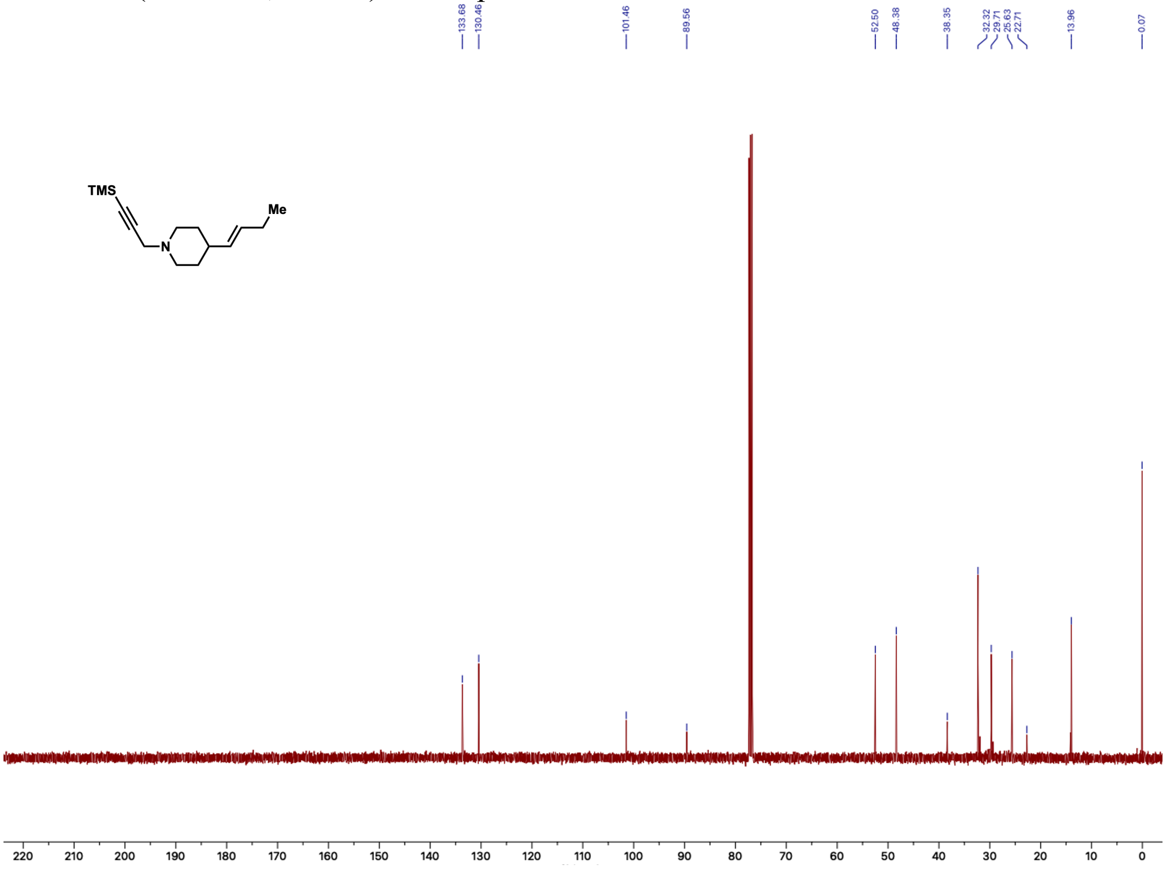

^{1}H NMR (300 MHz, $CDCl_3$) of compound **47**

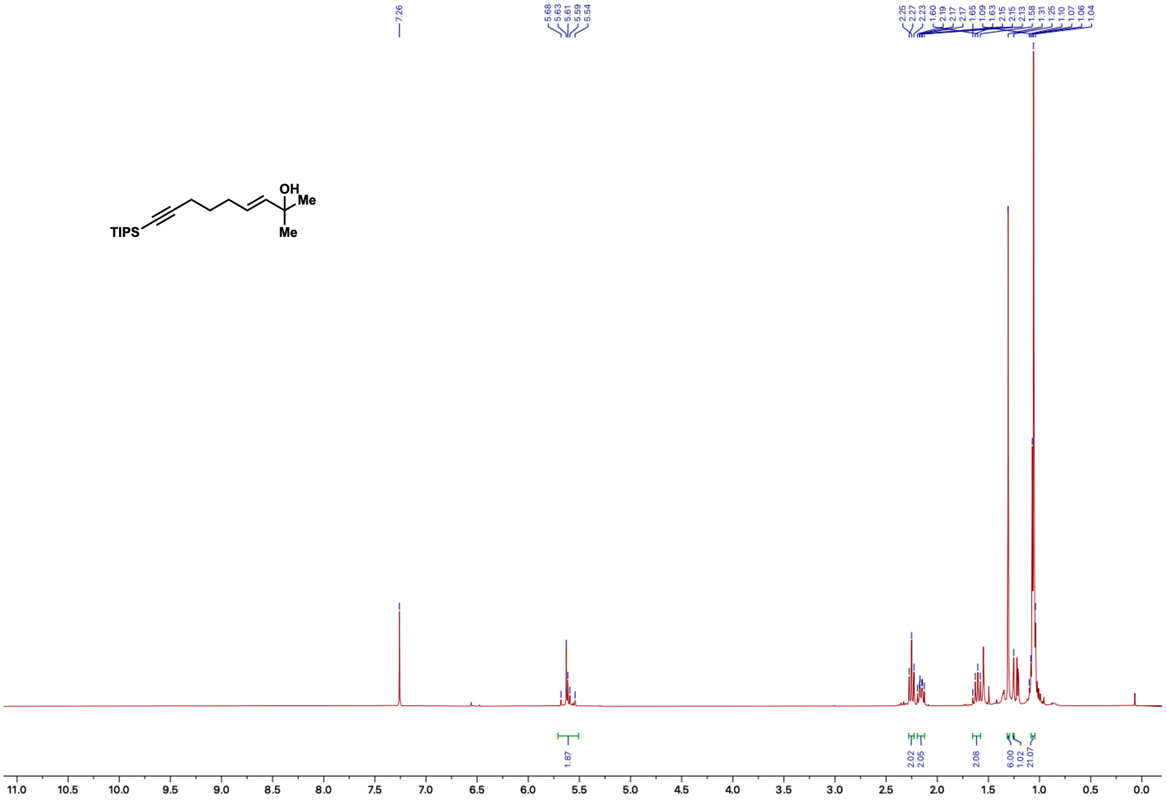

^{13}C NMR (126 MHz, $CDCl_3$) of compound **47**

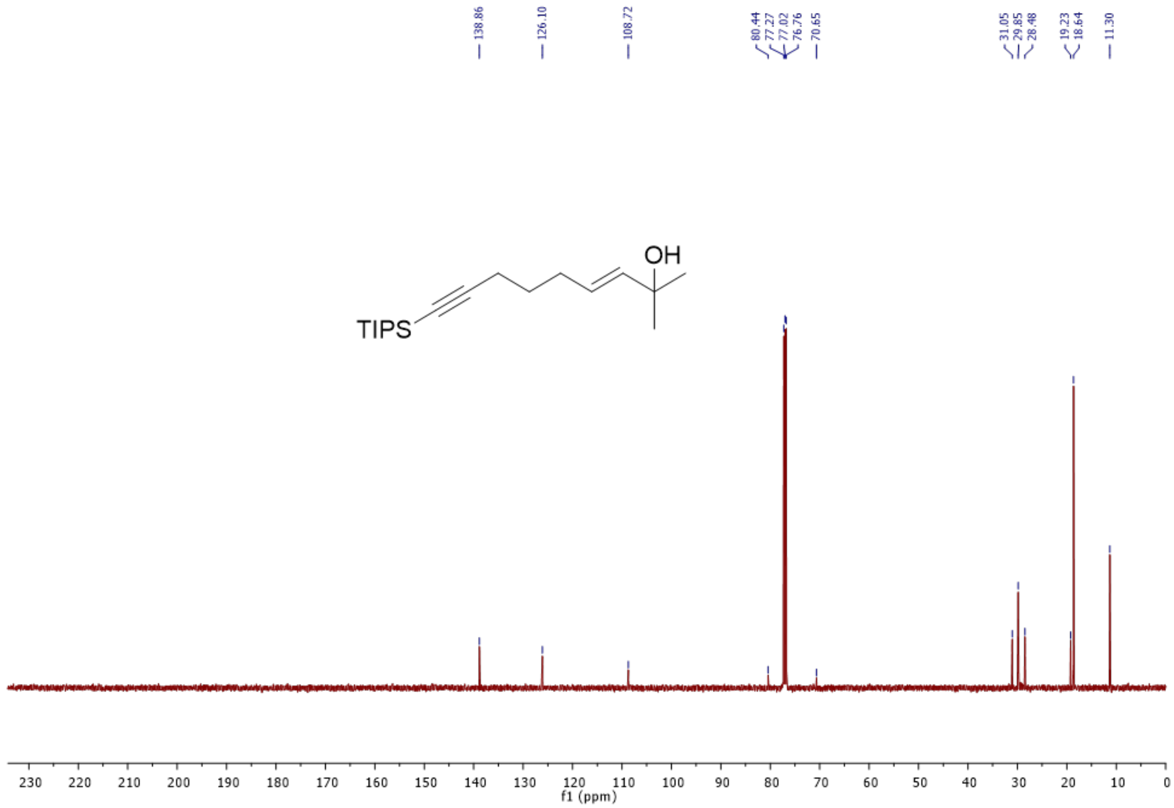

^1H NMR (300 MHz, $CDCl_3$) of compound **48**

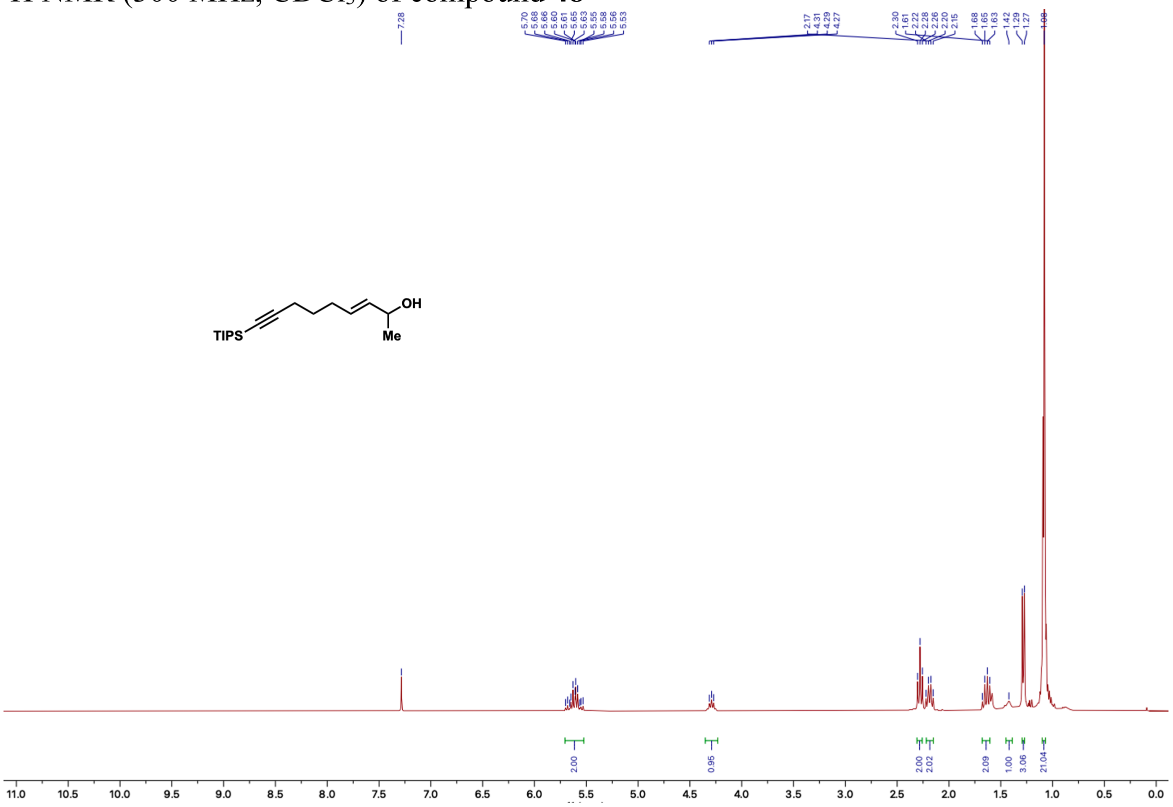

^{13}C NMR (75 MHz, $CDCl_3$) of compound **48**

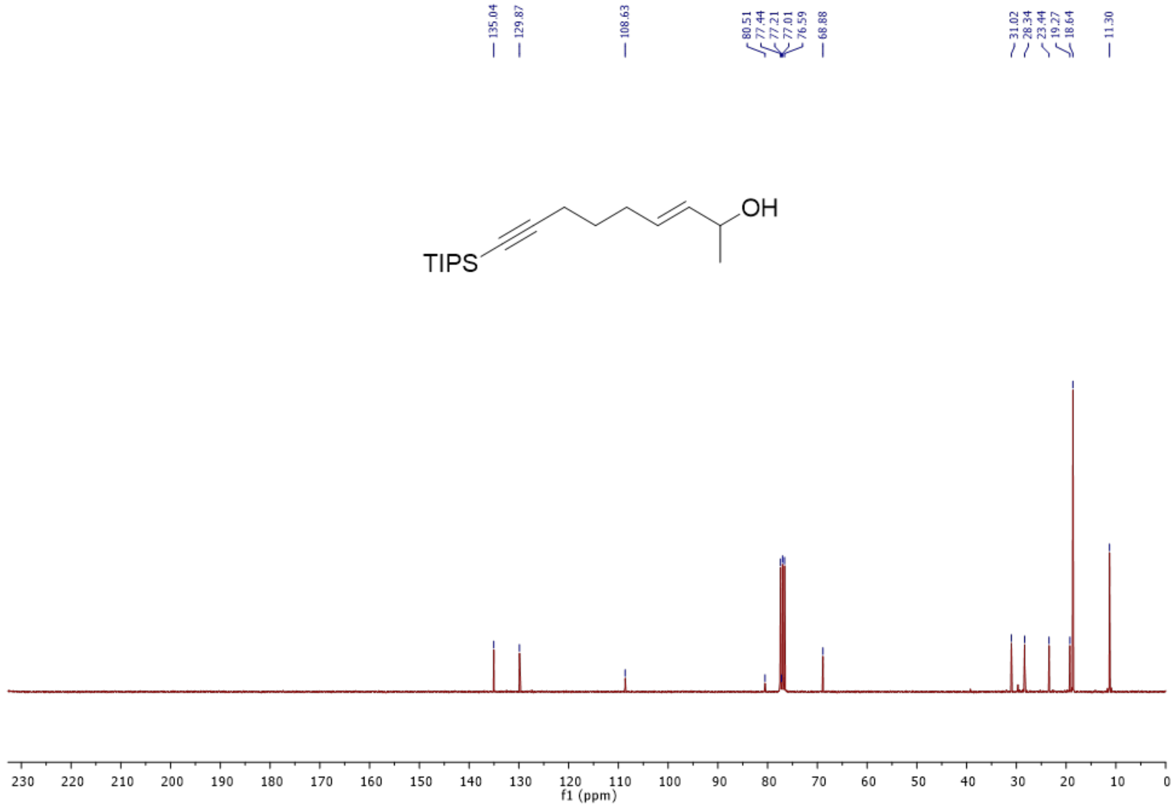

^{1}H NMR (500 MHz, $CDCl_3$) of compound **53**

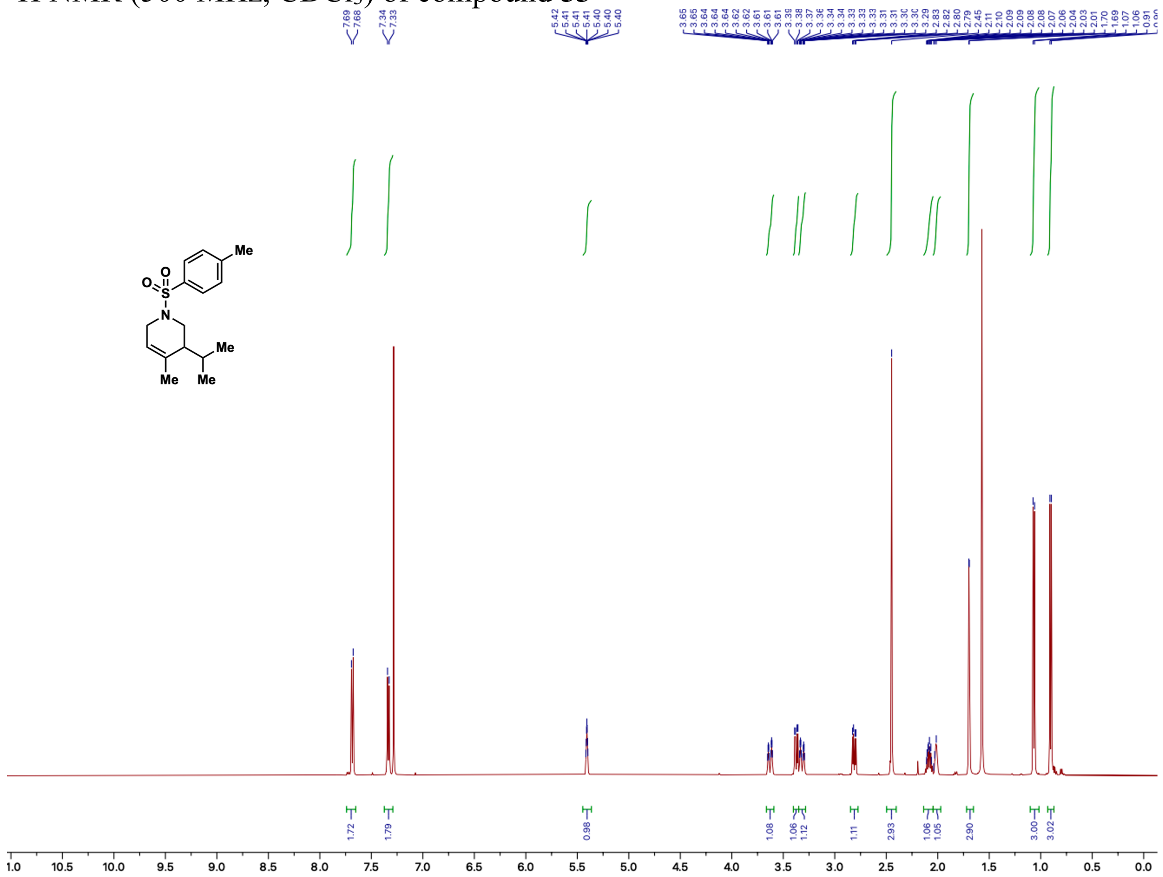

^{13}C NMR (126 MHz, CDCl$_3$) of compound **53**

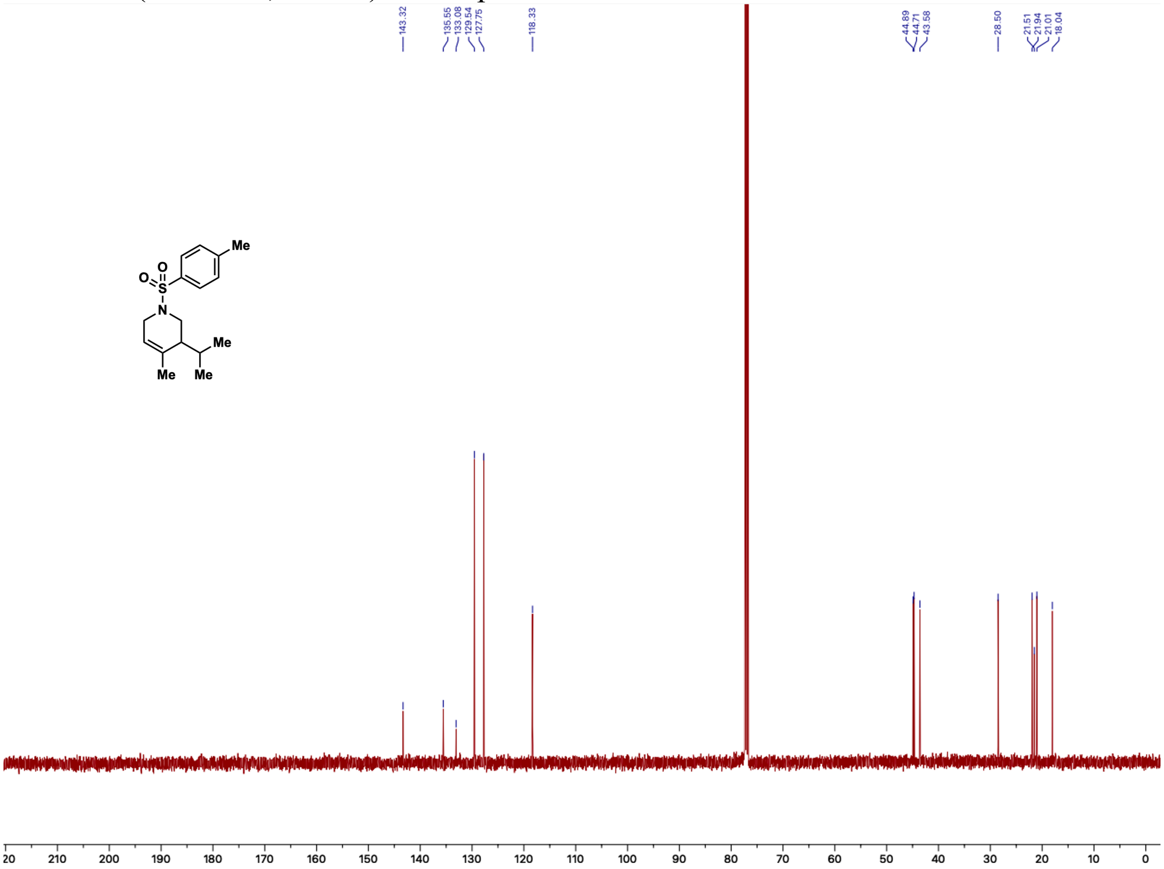

^{1}H-COSY of compound **53**

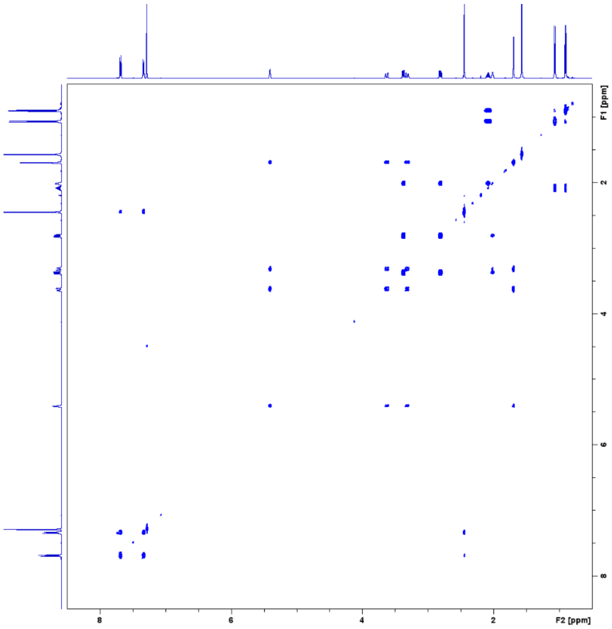

Expansion of the ^{1}H COSY experiment, including relevant correlations of H7.

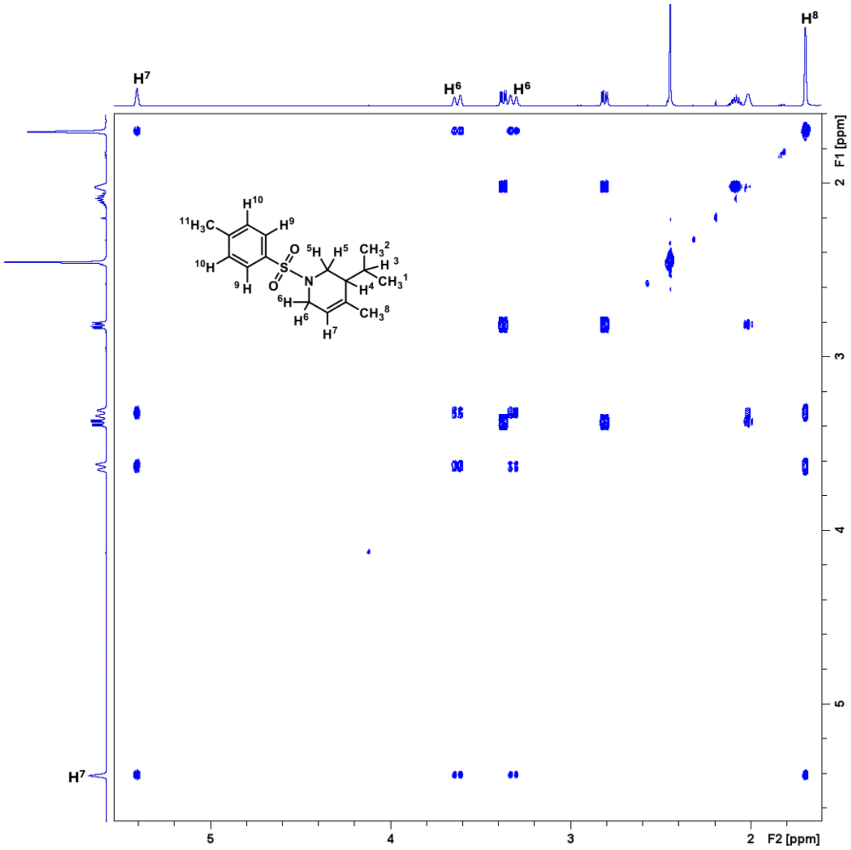

^{1}H-NOESY of compound **53**

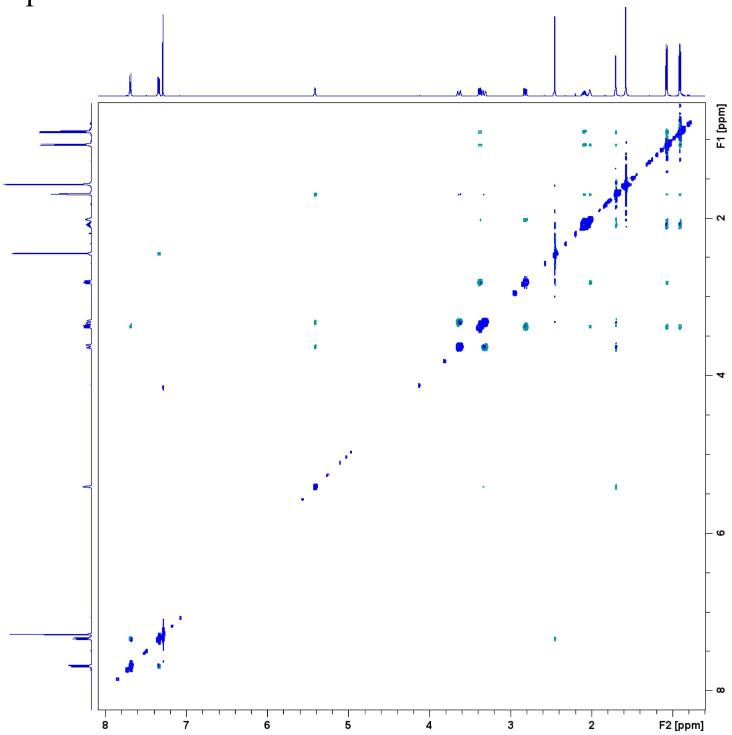

Expansion of ^{1}H NOESY experiment, including relevant correlations.

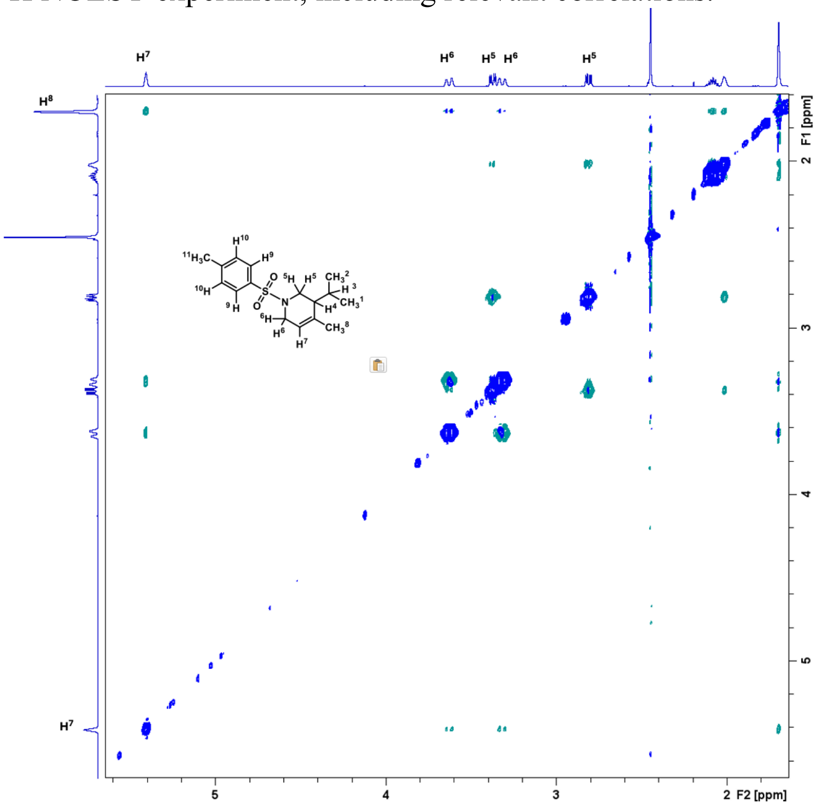

$^{1}H\{^{13}C\}$ HSQC of compound **53**

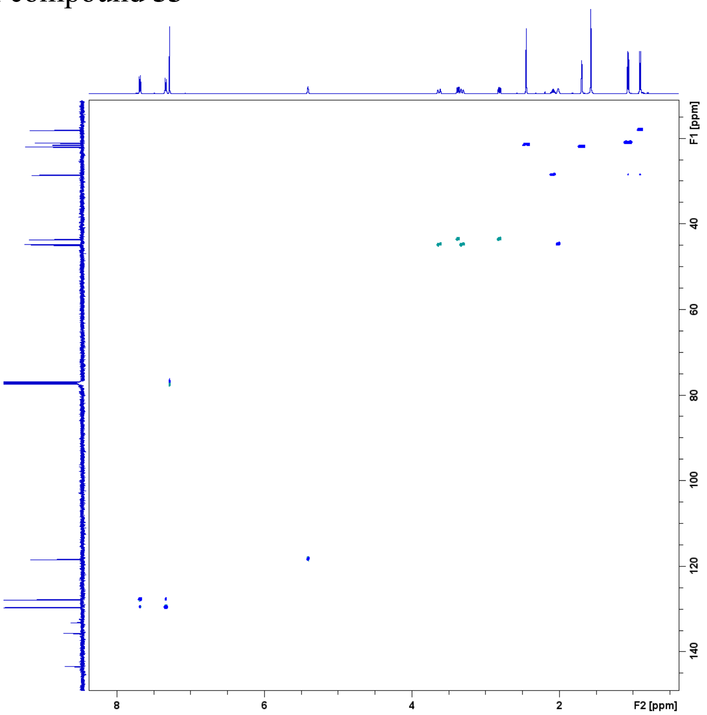

Expansion of $^{1}H\{^{13}C\}$ HSQC experiment, including relevant correlations.

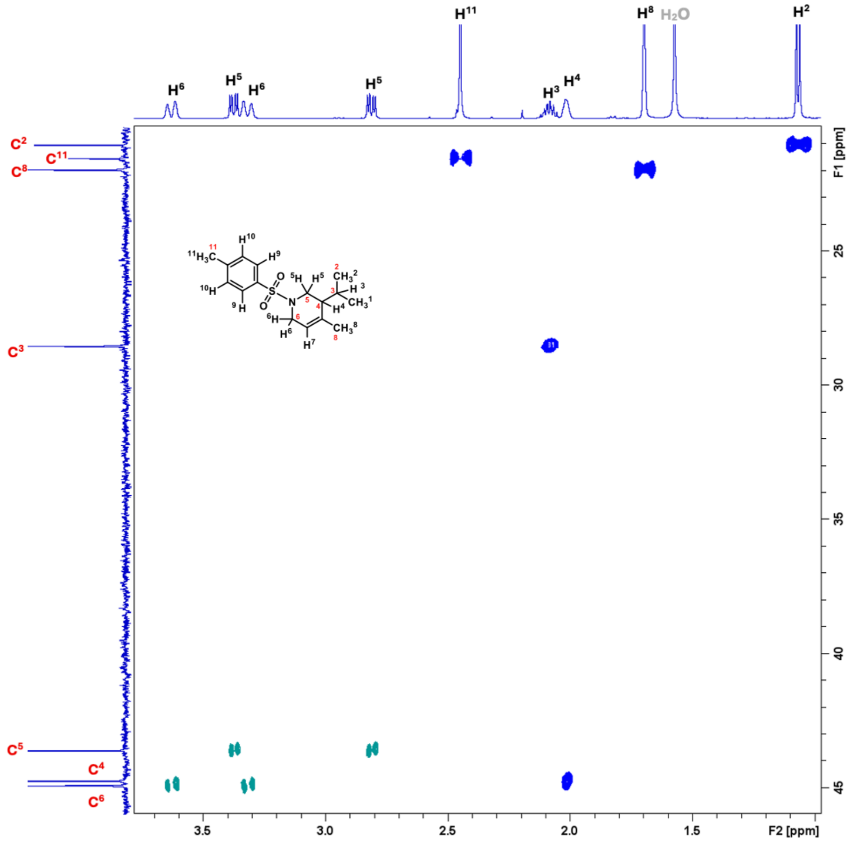

$^1H\{^{13}C\}$ HMBC of compound **53**

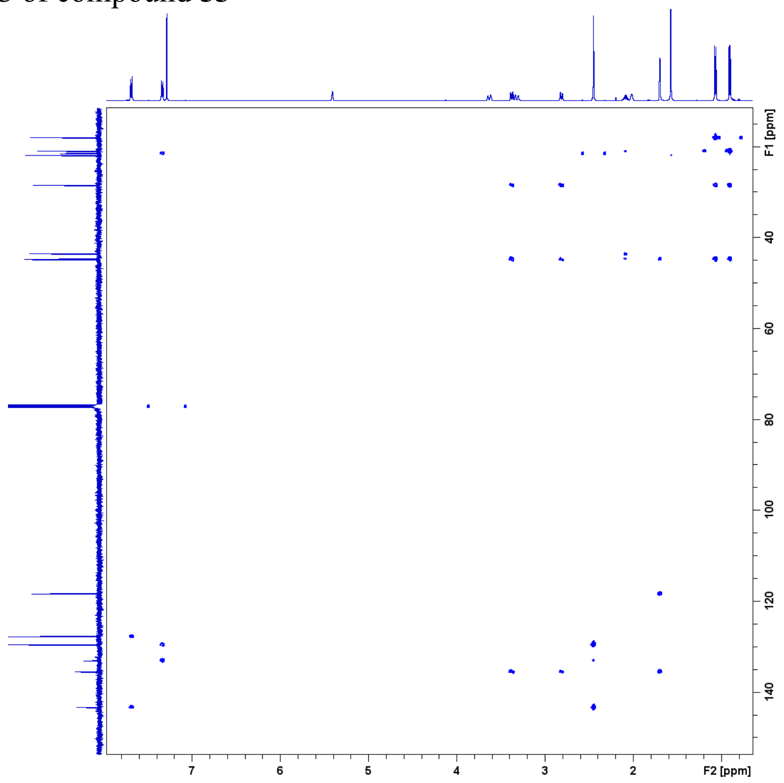

Expansion of $^1H\{^{13}C\}$ HMBC experiment, including relevant correlations.

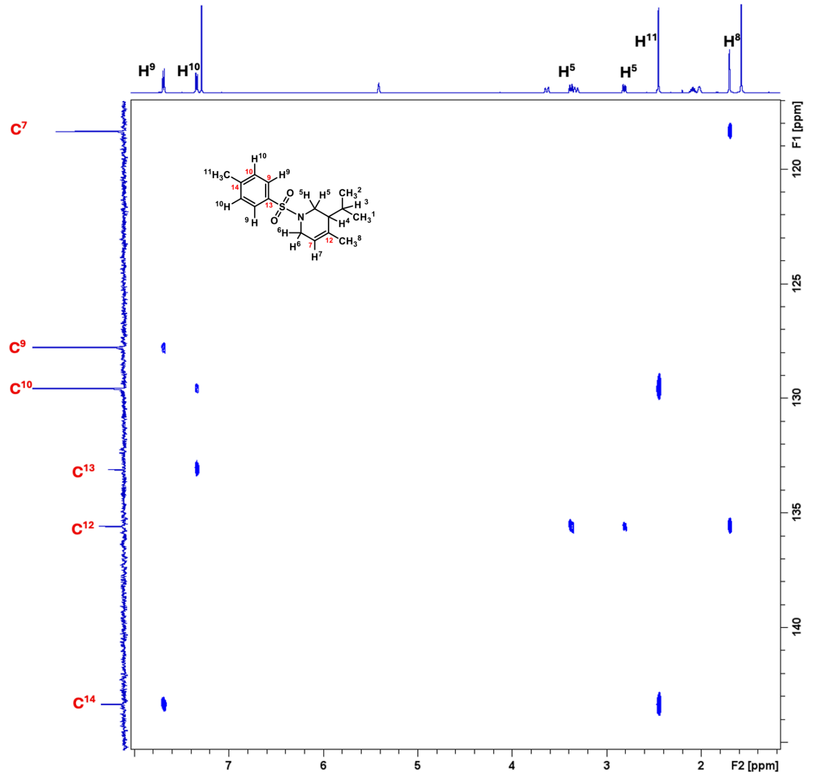

^{1}H NMR (400 MHz, $CDCl_3$) of compound **54**

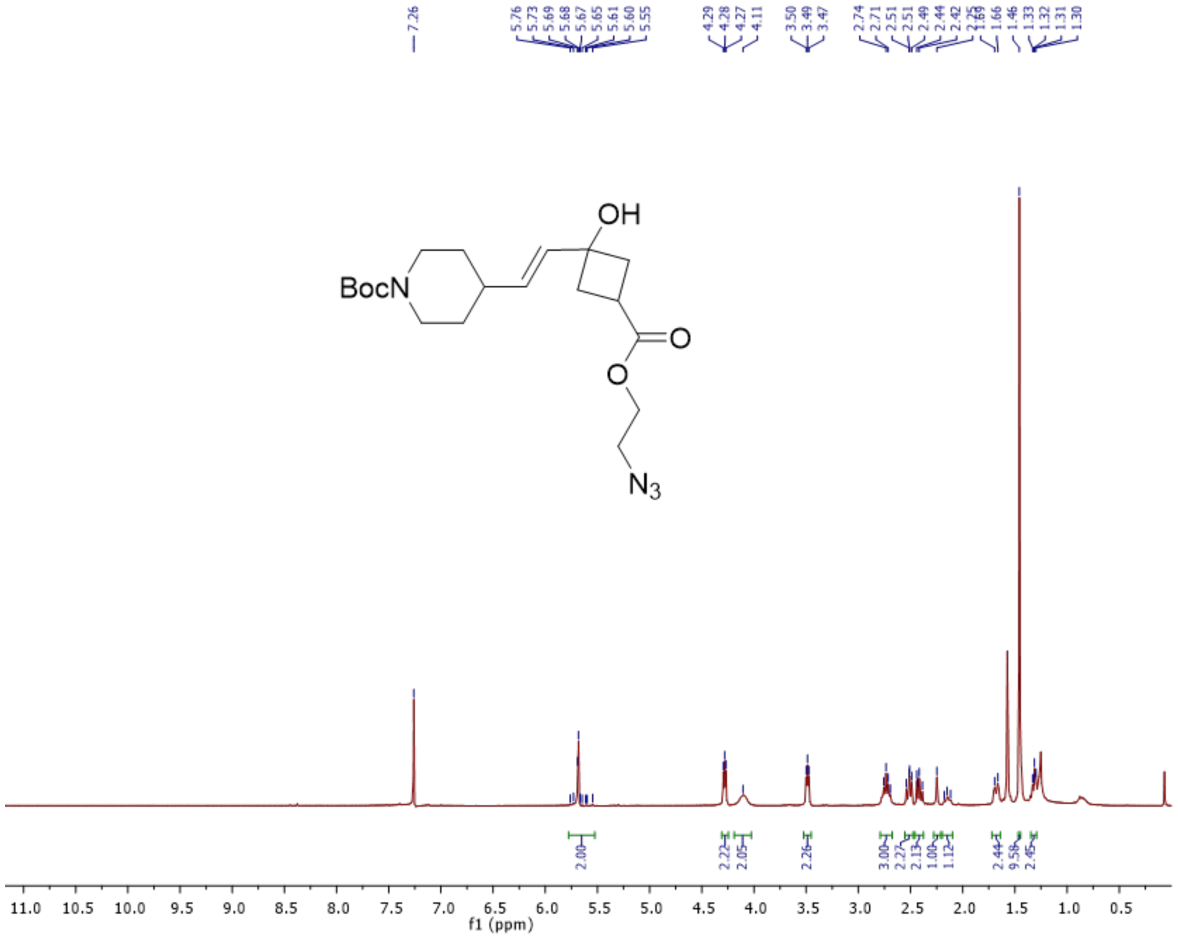

^{13}C NMR (101 MHz, $CDCl_3$) of compound **54**

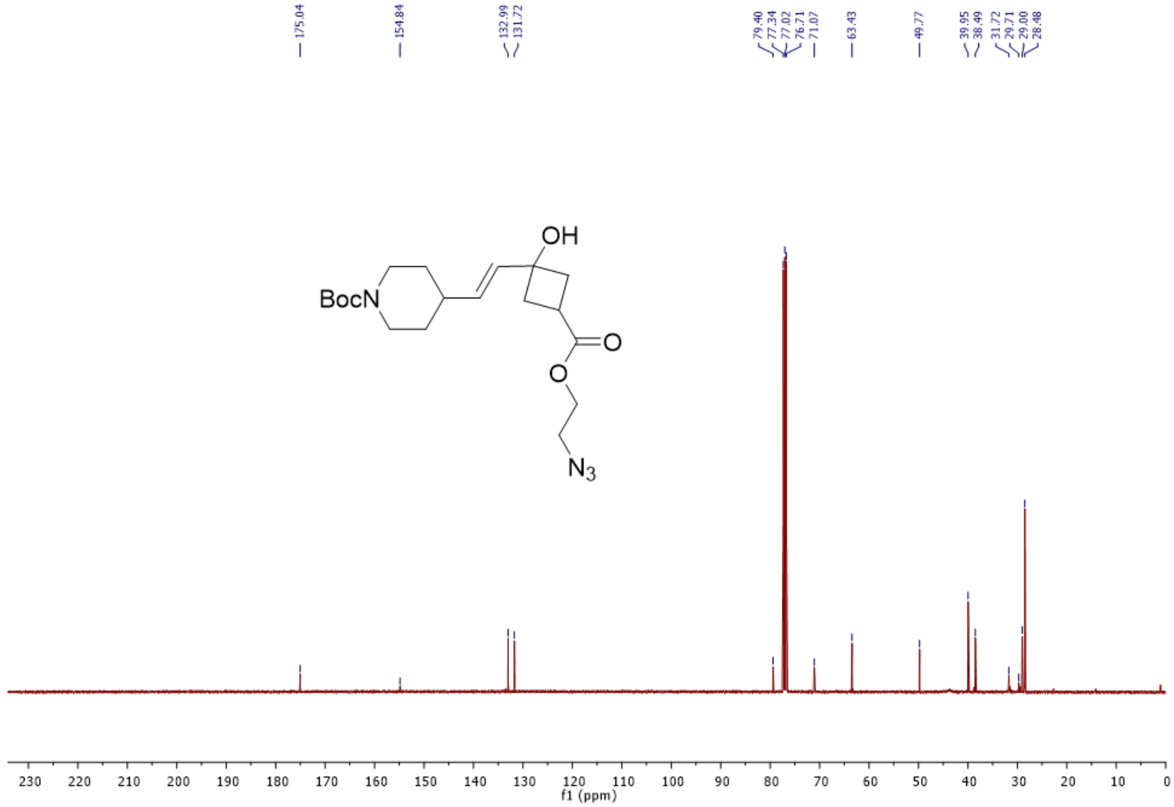

^{1}H NMR (800 MHz, $CDCl_3$) of compound **55** (with residue of unreacted alkyne)

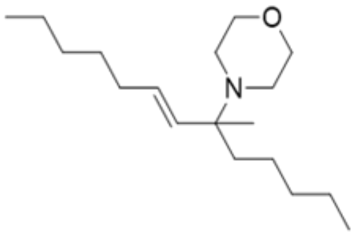

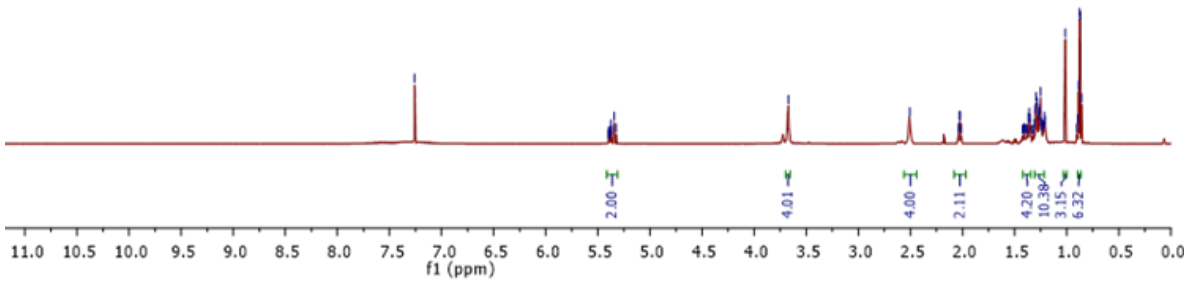

^{13}C NMR (201 MHz, $CDCl_3$) of compound **55** (with residue of unreacted alkyne)

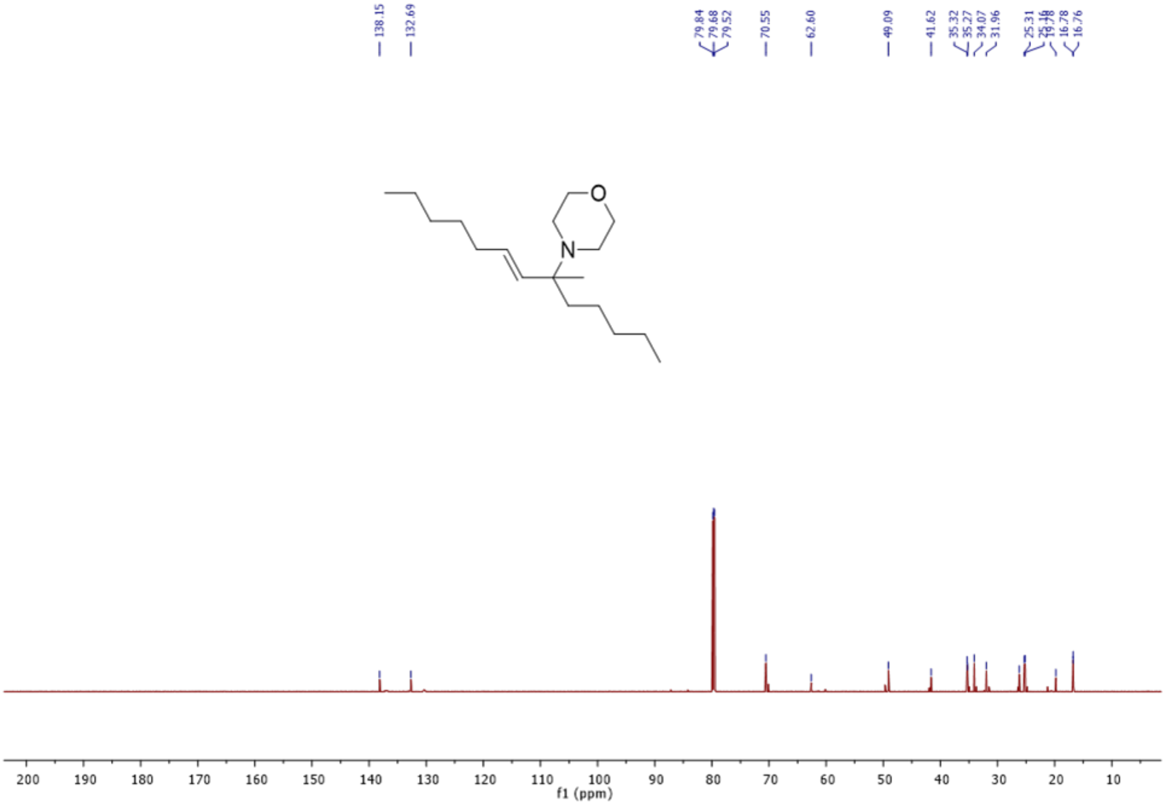

5. References

1. Kim, Y. B.; Kim, D.; Dighe, S. U.; Chang, S.; Park, J.-W. Cobalt-Hydride-Catalyzed Hydrosilylation of 3-Alkynes Accompanying π-Bond Migration. *ACS Catal.* **2021**, *11*(3), 1548–1553.
2. Saito, N.; Sun, Z.; Sato, Y. Nickel-Promoted Highly Regioselective Carboxylation of Aryl Ynol Ether and Its Application to the Synthesis of Chiral β-Aryloxypropionic Acid Derivatives. *Chem. Asian J.* **2015**, *10*(6), 1170–1176.
3. Huang, H.; Tang, L.; Liu, Q.; Xi, Y.; He, G.; Zhu, H. Formation of α-Chalcogenyl Acrylamides through Unprecedented Chalcogen-Mediated Metal-Free Oxyfunctionalization of Ynamides with DMSO as an Oxidant. *Chem. Commun.* **2016**, *52*(35), 5605–5608.
4. Li, C.; Zhang, L. Gold-Catalyzed Nitrene Transfer to Activated Alkynes: Formation of α,β-Unsaturated Amidines. *Org. Lett.* **2011**, *13*(7), 1738–1741.
5. Doi, R.; Abdullah, I.; Taniguchi, T.; Saito, N.; Sato, Y. Nickel-Catalyzed Hydrocarboxylation of Ynamides with CO_2 and H_2O: Observation of Unexpected Regioselectivity. *Chem. Commun.* **2017**, *53*(54), 7720–7723.
6. Hrubý, S.; Ulč, J.; Císařová, I.; Kotora, M. Synthesis of Cationic [4]-, [5]-, and [6]Azahelicenes with Extended π-Conjugated Systems. *Adv. Synth. Catal.* **2023**, *365*(7), 965–970.
7. Tang, S.; Wang, P.; Li, H.; Lei, A. Multimetallic Catalysed Radical Oxidative C(sp^3)–H/C(sp)–H Cross-Coupling between Unactivated Alkanes and Terminal Alkynes. *Nat. Commun.* **2016**, *7*, 11676.
8. Periasamy, M.; Reddy, P. O.; Satyanarayana, I.; Mohan, L.; Edukondalu, A. Diastereoselective Synthesis of Tetrasubstituted Propargylamines via Hydroamination and Metalation of 1-Alkynes and Their Enantioselective Conversion to Trisubstituted Chiral Allenes. *J. Org. Chem.* **2016**, *81*(3), 987–999.
9. Alcaide, B.; Almendros, P.; Lázaro-Milla, C. Regioselective Synthesis of Heteroatom-Functionalized Cyclobutene-Triflones and Cyclobutenones. *Adv. Synth. Catal.* **2017**, *359*(15), 2630–2639.

10. Asaro, M. F.; Nakayama, I.; Wilson Jr, R. B. Formation of sterically hindered primary vicinal diamines from vicinal and geminal dinitro compounds. *J. Org. Chem.,* 1992, *57*(2), 778-782.
11. Liu, J.; Rong, J.; Wood, D. P.; Wang, Y.; Liang, S. H.; Lin, S. Co-catalyzed hydrofluorination of alkenes: photocatalytic method development and electroanalytical mechanistic investigation. *J. Am. Chem. Soc.* **2024**, *146*(7), 4380-4392.
12. Motoyama, Y.; Abe, M., Kamo, K.; Kosako, Y.; Nagashima, H. Encapsulated molecular catalysts in polysiloxane gels: ruthenium cluster-catalyzed isomerization of alkenes. *Chem. Commun.* 2008, (42), 5321-5323.
13. Li, D. R.; He, A.; Falck, J. R. Enantioselective, organocatalytic reduction of ketones using bifunctional thiourea-amine catalysts. *Org. Lett.*, 2010, 12(8), 1756-1759.
14. House, H. O.; Kinloch, E. F. Reactions Involving Electron Transfer. V. Reduction of Nonconjugated Acetylenes. *J. Org. Chem.* **1974**, *39*(6), 747–755.
15. Birch, A. J. Reduction by Dissolving Metals. Part I. *J. Chem. Soc.* **1944**, *1944*, 430–436.
16. Grant, B.; Djerassi, C. Mechanism of hydride reduction of 1-alkyn-3-ols. *The J. Org. Chem.* **1974**, *39*(7), 968-970.
17. Ashby, E. C.; Lin, J. J. Selective Reduction of Alkenes and Alkynes by the Reagent Lithium Aluminum Hydride–Transition-Metal Halide. *J. Org. Chem.* **1978**, *43*(13), 2567–2572.
18. Gaussian 16, Revision C.01; Frisch, M. J.; Trucks, G. W.; Schlegel, H. B.; Scuseria, G. E.; Robb, M. A.; Cheeseman, J. R.; Scalmani, G.; Barone, V.; Petersson, G. A.; Nakatsuji, H.; Li, X.; Caricato, M.; Marenich, A. V.; Bloino, J.; Janesko, B. G.; Gomperts, R.; Mennucci, B.; Hratchian, H. P.; Ortiz, J. V.; Izmaylov, A. F.; Sonnenberg, J. L.; Williams-Young, D.; Ding, F.; Lipparini, F.; Egidi, F.; Goings, J.; Peng, B.; Petrone, A.; Henderson, T.; Ranasinghe, D.; Zakrzewski, V. G.; Gao, J.; Rega, N.; Zheng, G.; Liang, W.; Hada, M.; Ehara, M.; Toyota, K.; Fukuda, R.; Hasegawa, J.; Ishida, M.; Nakajima, T.; Honda, Y.; Kitao, O.; Nakai, H.; Vreven, T.; Throssell, K.; Montgomery, J. A., Jr.; Peralta, J. E.; Ogliaro, F.; Bearpark, M. J.; Heyd, J. J.; Brothers, E. N.; Kudin, K. N.; Staroverov, V. N.; Keith, T. A.; Kobayashi, R.; Normand, J.; Raghavachari, K.; Rendell, A. P.; Burant, J. C.; Iyengar, S. S.; Tomasi, J.; Cossi, M.;

Millam, J. M.; Klene, M.; Adamo, C.; Cammi, R.; Ochterski, J. W.; Martin, R. L.; Morokuma, K.; Farkas, O.; Foresman, J. B.; Fox, D. J. Gaussian, Inc., Wallingford CT, 2016.

19. Neese, F. Software Update: The ORCA Program System—Version 6.0. *WIREs Comput. Mol. Sci.* **2025**, *15* (2), e70019.
20. Perdew, J. P.; Burke, K.; Ernzerhof, M. Generalized Gradient Approximation Made Simple. *Phys. Rev. Lett.* **1996**, *77*, 3865–3868.
21. Perdew, J. P.; Burke, K.; Ernzerhof, M. Generalized Gradient Approximation Made Simple [Phys. Rev. Lett. 77, 3865 (1996)]. *Phys. Rev. Lett.* **1997**, *78*, 1396.
22. Grimme, S.; Antony, J.; Ehrlich, S.; Krieg, H. A Consistent and Accurate *Ab Initio* Parametrization of Density Functional Dispersion Correction (DFT-D) for the 94 Elements H–Pu. *J. Chem. Phys.* **2010**, *132* (15), 154104.
23. Grimme, S.; Ehrlich, S.; Goerigk, L. Effect of the Damping Function in Dispersion Corrected Density Functional Theory. *J. Comput. Chem.* **2011**, *32* (7), 1456–1465.
24. Weigend, F.; Ahlrichs, R. Balanced Basis Sets of Split Valence, Triple Zeta Valence and Quadruple Zeta Valence Quality for H to Rn: Design and Assessment of Accuracy. *Phys. Chem. Chem. Phys.* **2005**, *7*, 3297–3305.
25. Weigend, F. Accurate Coulomb-Fitting Basis Sets for H to Rn. *Phys. Chem. Chem. Phys.* **2006**, *8*, 1057–1065.
26. Santra, G.; Sylvetsky, N.; Martin, J. M. L. Minimally Empirical Double Hybrid Functionals Trained Against the GMTKN55 Database: revDSD-PBEP86-D4, revDOD-PBE-D4, and DOD-SCAN-D4. *J. Phys. Chem. A* **2019**, *123* (24), 5129–5143.
27. Caldeweyher, E.; Bannwarth, C.; Grimme, S. Extension of the D3 Dispersion Coefficient Model. *J. Chem. Phys.* **2017**, *147* (3), 034112.
28. Caldeweyher, E.; Ehlert, S.; Hansen, A.; Neugebauer, H.; Spicher, S.; Bannwarth, C.; Grimme, S. A Generally Applicable Atomic-Charge Dependent London Dispersion Correction. *J. Chem. Phys.* **2019**, *150* (15), 154122.
29. Field-Theodore, T. E.; Olejniczak, M.; Jaszuński, M.; Wilson, D. J. D. NMR Shielding Constants for Group 15 Trifluorides. *Phys. Chem. Chem. Phys.* **2018**, *20* (35), 23025–23033.

30. Weigend, F.; Kattannek, M.; Ahlrichs, R. Approximated Electron Repulsion Integrals: Cholesky Decomposition versus Resolution of the Identity Methods. *J. Chem. Phys.* **2009**, *130* (16), 164106.
31. Weigend, F. Hartree–Fock Exchange Fitting Basis Sets for H to Rn. *J. Comput. Chem.* **2008**, *29* (2), 161–175.
32. Hellweg, A.; Hättig, C.; Höfener, S.; Klopper, W. Optimized Accurate Auxiliary Basis Sets for RI-MP2 and RI-CC2 Calculations for the Atoms Rb to Rn. *Theor. Chem. Acc.* **2007**, *117* (4), 587–597.
33. Marenich, A. V.; Cramer, C. J.; Truhlar, D. G. Universal Solvation Model Based on Solute Electron Density and on a Continuum Model of the Solvent Defined by the Bulk Dielectric Constant and Atomic Surface Tensions. *J. Phys. Chem. B* **2009**, *113* (18), 6378–6396.
34. Cramer, C. J. *Essentials of Computational Chemistry: Theories and Models*; 2nd ed.; John Wiley & Sons: Chichester, UK, 2007.
35. Besora, M.; Vidossich, P.; Lledós, A.; Ujaque, G.; Maseras, F. Calculation of Reaction Free Energies in Solution: A Comparison of Current Approaches. *J. Phys. Chem. A* **2018**, *122* (5), 1392–1399.
